# Supplementary material for: Chromosome-associated RNA–protein complexes promote pairing of homologous chromosomes during meiosis in Schizosaccharomyces pombe
Source: Nat Commun. 2019 Dec 6;10:5598. doi: 10.1038/s41467-019-13609-0 (PMC6898681; doi:10.1038/s41467-019-13609-0)
Supplement: Supplementary file 1 — Supplementary Information [file 41467_2019_13609_MOESM1_ESM.pdf]

Chromosome-associated RNA-protein complexes promote pairing of homologous chromosomes during meiosis in *Schizosaccharomyces pombe*

Ding *et al.*

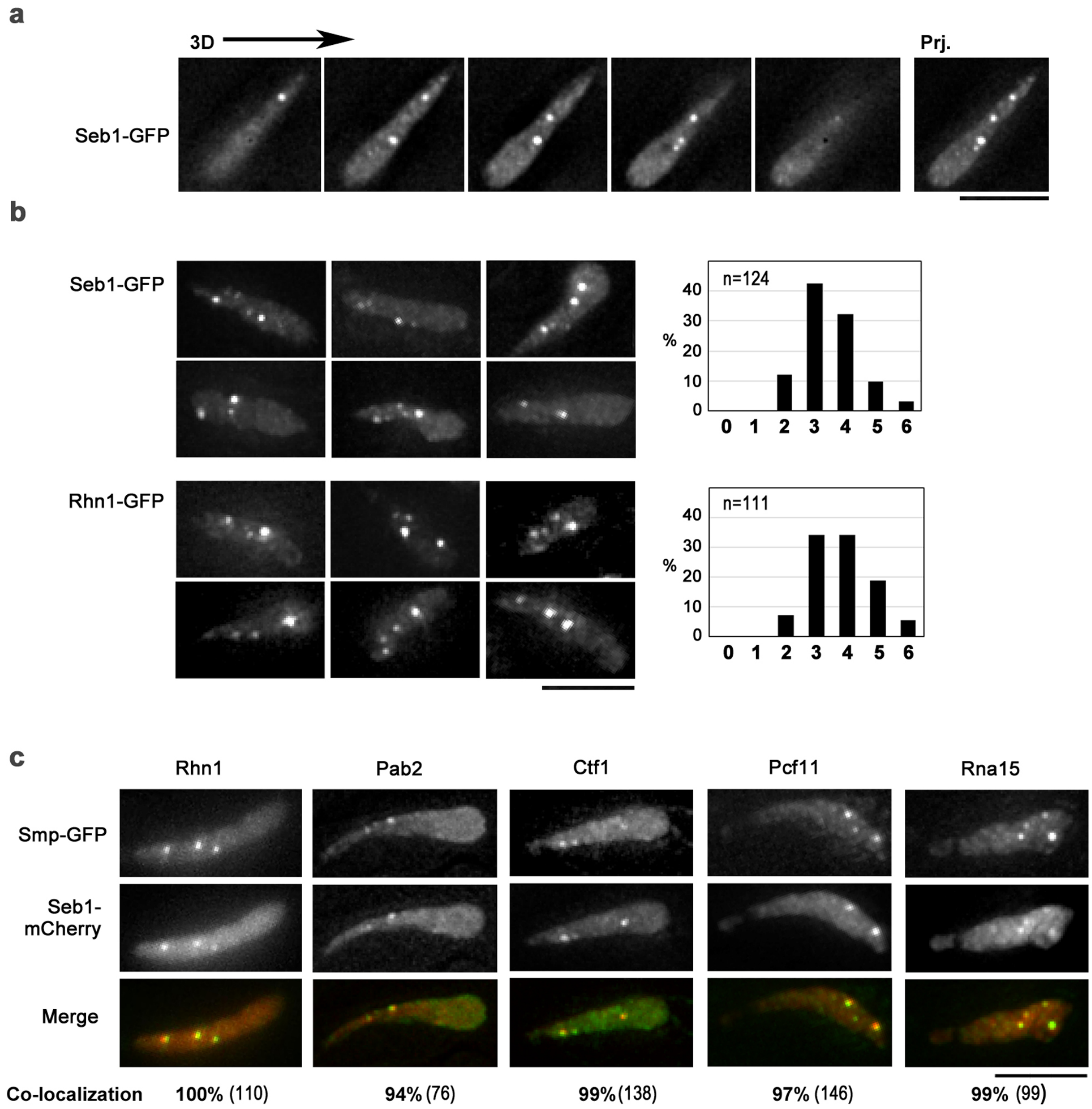

Supplementary Figure 1. Chromosomal accumulation of Smp proteins
(a) Optical section images of Seb1-GFP in the horsetail nucleus were acquired at a 0.35 μm focus interval. Optical section images were projected to produce the projection image (Prj). **(b)** Examples of projection images for Seb1-GFP and Rhn1-GFP (left). The number distribution of Seb1-GFP and Rhn1-GFP dots (right). The "dots" were identified using the 2D polygon finder function of softWoRx software (GE Healthcare); the fluorescence intensity threshold of a "dot" was set as 1.5 times higher than the average intensity in the nucleus. "n" in the bar graphs represents the number of horsetail nuclei examined. Source data are provided as a Source Data 6. **(c)** Co-localization of GFP-fused Smp proteins with Seb1-mCherry in the horsetail nucleus. The number below indicates percentage of Smp dots colocalized with Seb1 dots; the number in parentheses indicates the number of cells examined. Scale bars represent 5 μm (**a**-**c**).

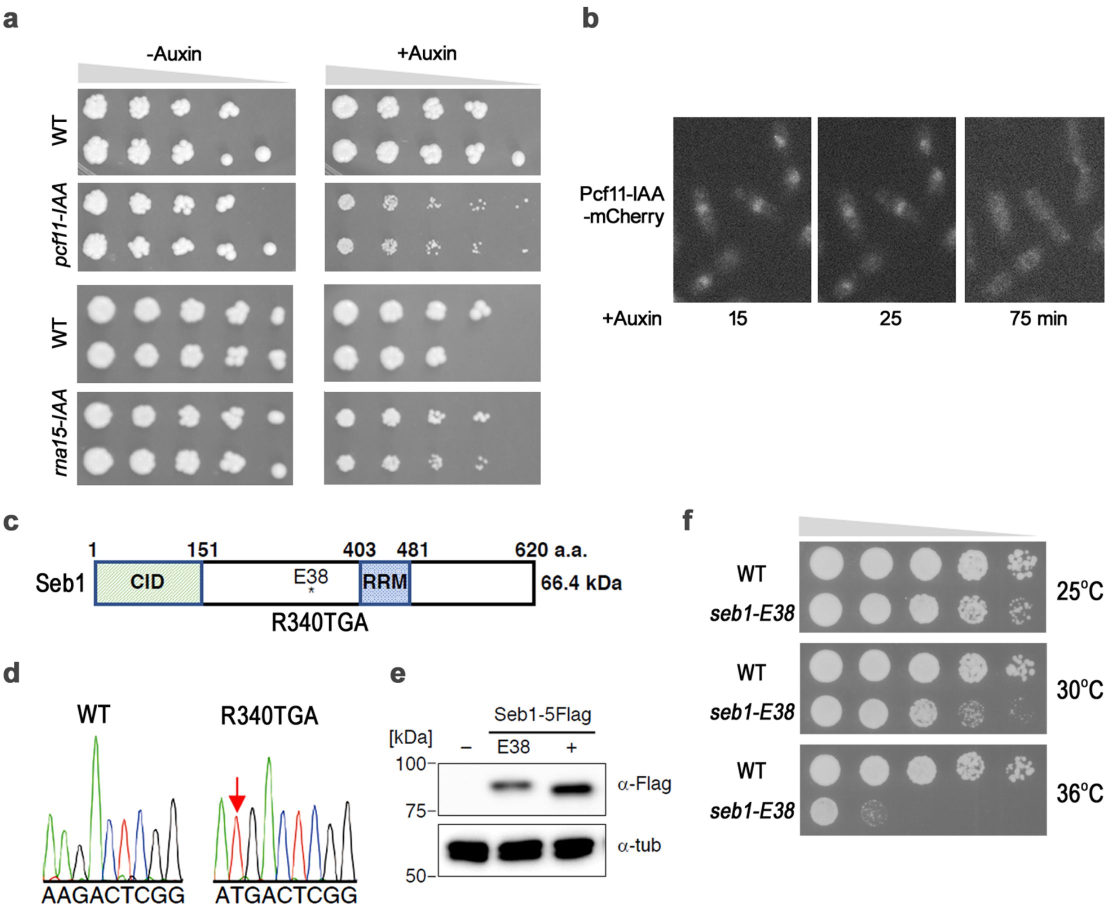

g

	Normal Segregation	No.	Normal Spore Formation	No.	Spore Viability	No.
WT	93.7%	111	95.0%	221	88.5%	312
seb1^{-}	77.6%	156	81.7%	223	84.8%	312
Δrhn1	84.9%	126	76.4%	246	20.5%	312
Δpab2	85.2%	81	80.0%	202	83.7%	312
seb1^{-}*Δrhn1*	54.0%	89	30.6%	829	30.2%	312

Supplementary Figure 2. Mutant characterization

(a) Growth assay of Pcf11 and Rna15 degron strains (*pcf11-IAA*, *rna15-IAA*) in the culture medium with or without Auxin. The plates were incubated for 3 days at 30°C. **(b)** An example of live cell imaging of Pcf11-IAA-mCherry degradation after adding Auxin and incubation at 26°C in EMM2-N medium. The fluorescent signals disappeared from the nuclei at 75 min after incubation in the medium containing Auxin. **(c)** Diagram of Seb1 protein feature. CID: CTD-interacting domain. RRM: RNA recognition motif. *: E38 (R340TGA) mutation.

(d) Sequencing of *seb1-E38* mutation. The *E38* allele bears a nonsense mutation converting R340 to a termination codon (TGA). **(e)** Immunoblot analysis of the Seb1-

E38 mutant. α-Tubulin was used as a loading control. (-) No tag strain (FY648), (E38) Seb1-E38-5Flag (EON125), (+) Seb1-5Flag (EON06). Primary antibody: (α-Flag) anti-Flag M2 antibody (1/1000 dilution; A8592, Merck), (α-tub) anti-α-tubulin (1/1000 dilution; T5168, Merck). Secondary antibody: Peroxidase-conjugated AffiPure anti-mouse IgG (1/1000 dilution; 515-035-072, Jackson). The Seb1-E38 mutant strain expresses a reduced amount of the full-length Seb1 protein. Source data are provided as a Source Data file S2. **(f)** Seb1-E38 growth assay. Five-fold-diluted cultures of the indicated strains were plated on YEA medium plates and incubated for 2–3 days at the indicated temperature. **(g)** Meiotic chromosome segregation (after meiotic division II, using cen2-*lacO*/lacI-GFP as marker), spore formation and spore viability in wild type cells and Smp mutants.

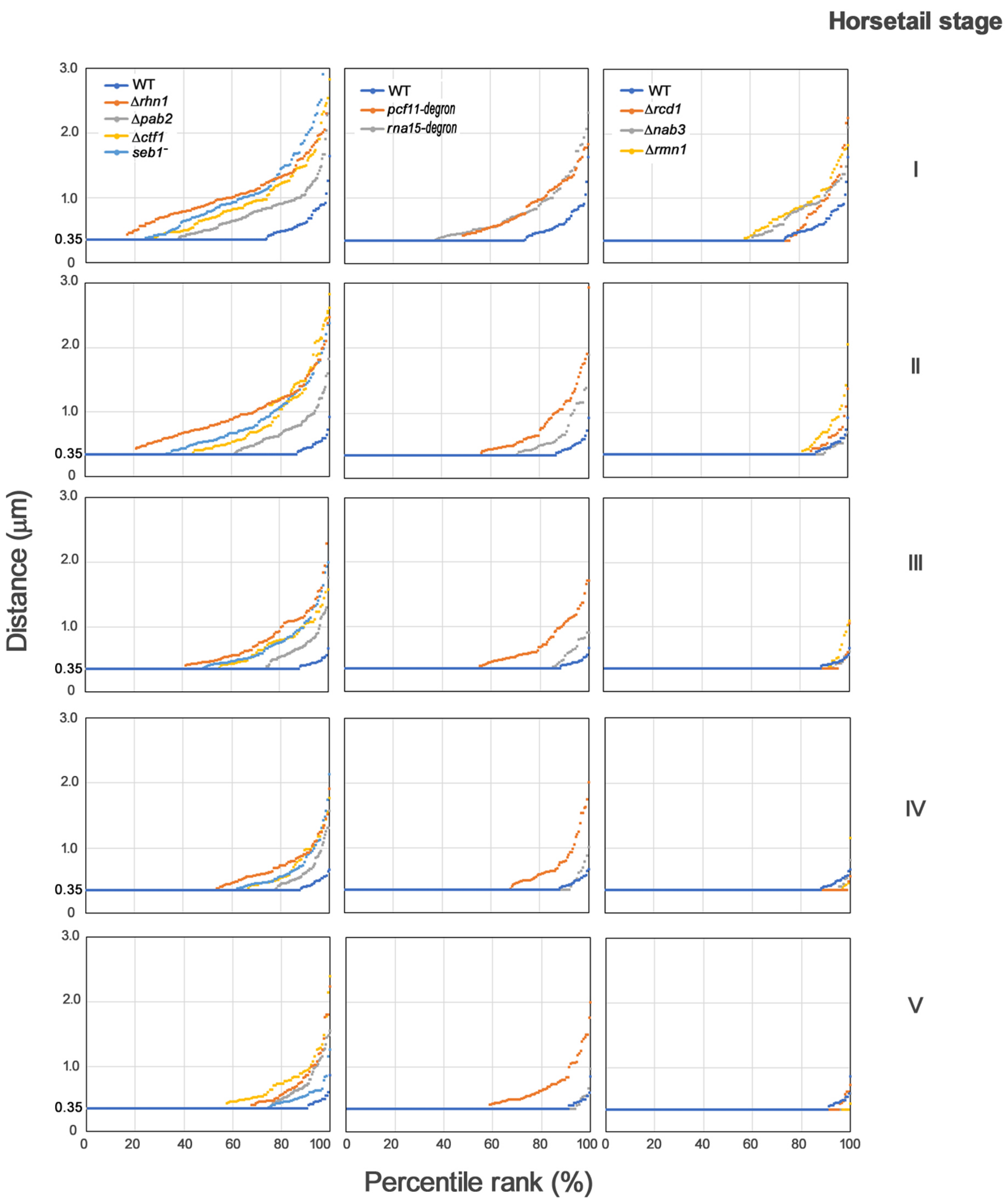

Supplementary Figure 3. Percentile plot of the distance

Percentile rank plots of the distance between homologous *sme2* loci in every horsetail stage shown in Fig. 1c. The threshold of pairing is 0.35 µm as labeled on the Y axis. Source data are provided as a Source Data file 1b.

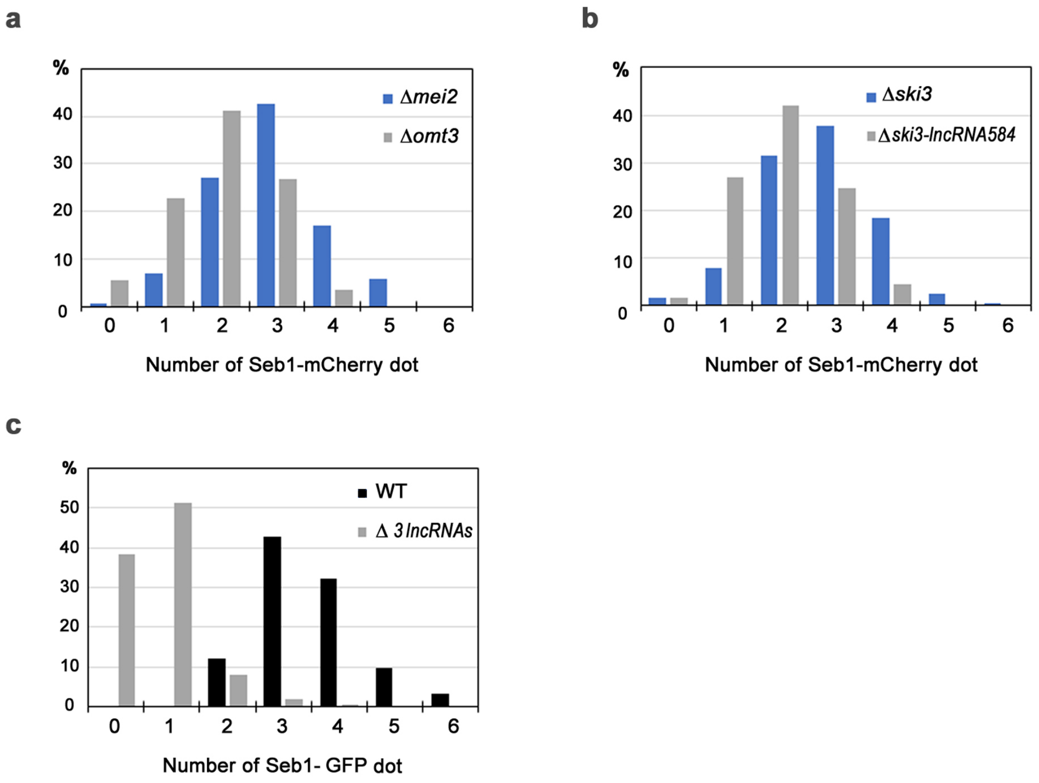

Supplementary Figure 4. Quantification of the Smp protein dots
(a) Number of Seb1-mCherry dots in the horsetail nuclei of Δ*mei2* and Δ*omt3* cells. **(b)** Number of Seb1-mCherry dots in the horsetail nuclei of Δ*ski3* and Δ*ski3-lncRNA584* cells. **(c)** Number of Seb1-GFP dots in the horsetail nuclei of wild type and in a strain lacking all the three lncRNAs, *sme2* RNA, *omt3* RNA, and lncRNA584. Source data are provided as a Source Data file S4.

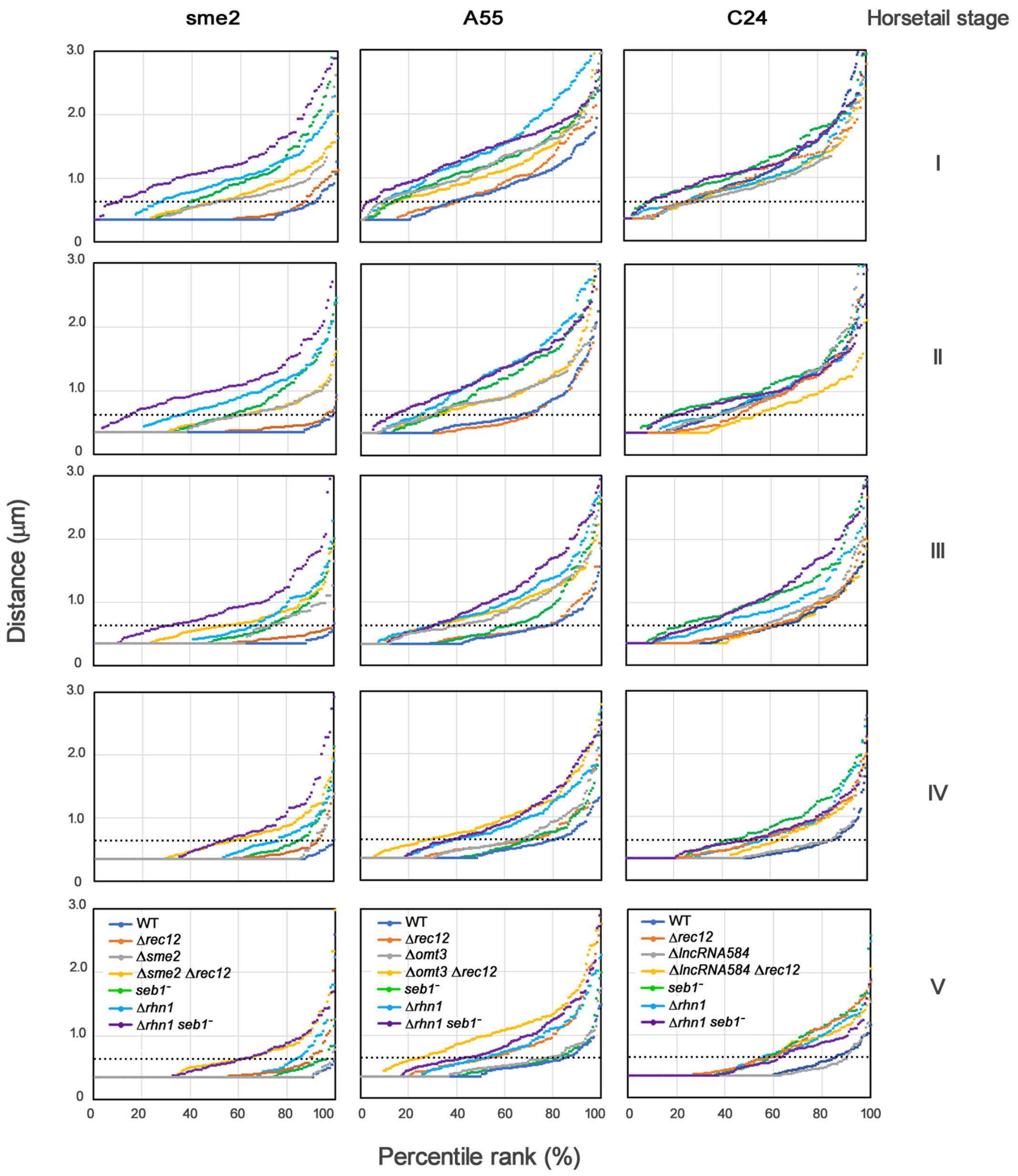

Supplementary Figure 5. Percentile plot of the distance

Percentile rank plots of the distance between homologous *sme2*, A55 and C24 loci in every horsetail stage shown in Fig. 4. The dotted line at 0.67 µm on the Y axis indicates the threshold of pairing. Source data are provided as a Source Data file 4.

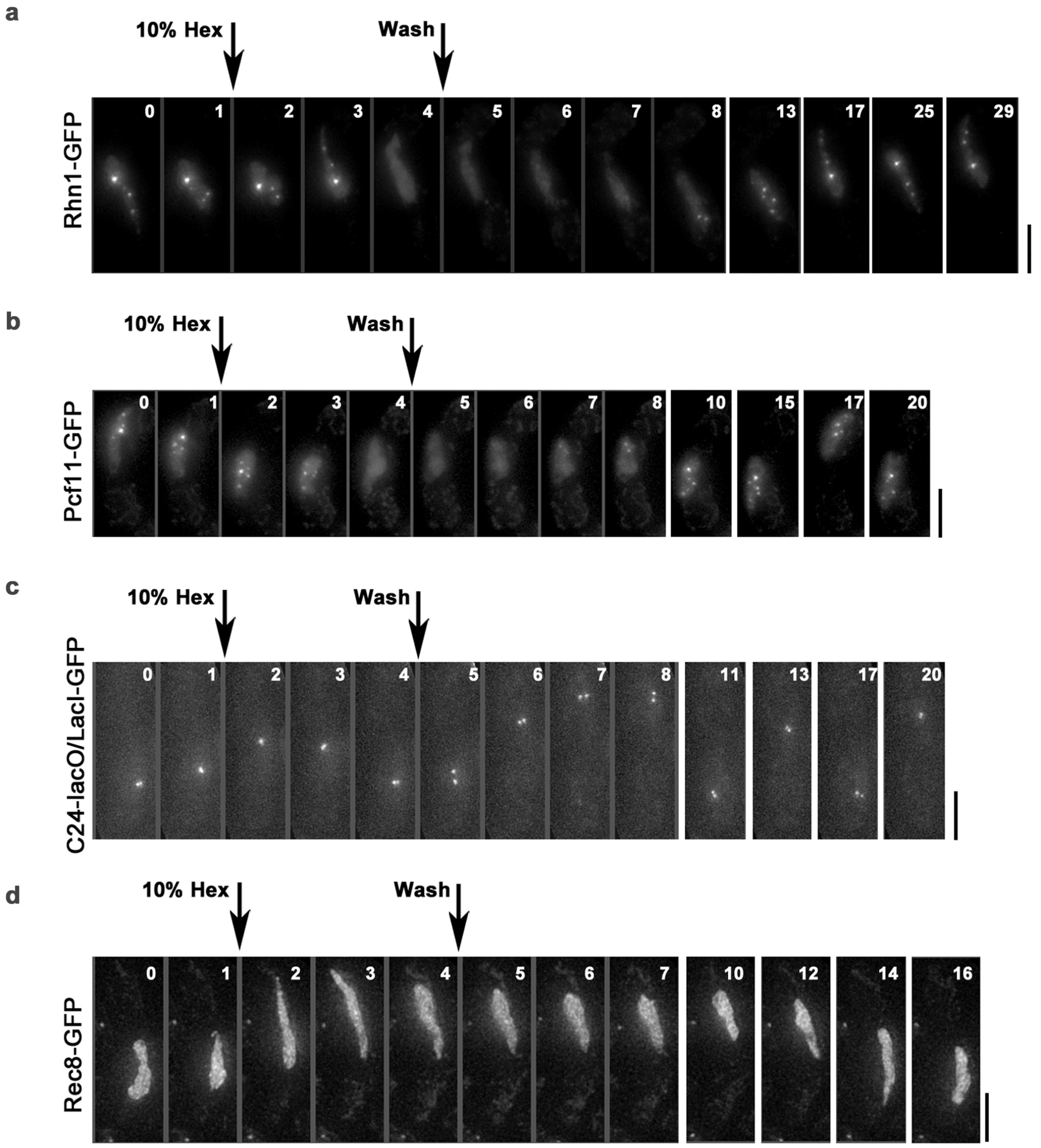

Supplementary Figure 6. Liquid-phase separation of Smp proteins and its role in pairing of homologous loci

(a) Selected projected time-lapse images of Rhn1-GFP in living cells at the horsetail stage. **(b)** Selected projected time-lapse images of Pcf11-GFP in living cells at the horsetail stage. **(c)** Selected projected time-lapse images of C24-*lacO*/LacI-GFP in living cells at the horsetail stage. (**d**) Selected projected time-lapse images of Rec8-GFP in living cells at the horsetail stage. Three-dimensional images were taken every minute. Microfluidic yeast plates (CellASIC) were used for cell culture on the microscope stage. 1,6-Hexanediol (10%) was added or removed, as indicated by the arrows. The solution in the cell culture chamber was changed in < 1 min. The scale bars represent 5 μm.

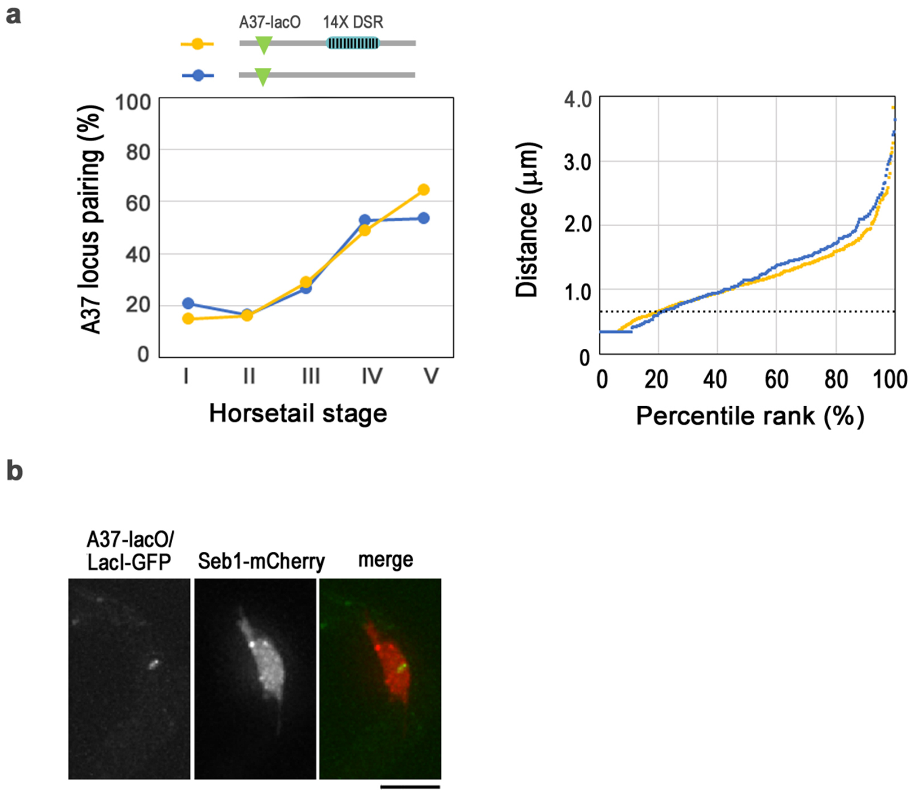

Supplementary Figure 7. Effect of DSR repeats on homologous pairing.
(a) Pairing frequency at the A37 locus in wild type (blue) and in the14×DSR motif (TTAAAC) repeats inserted strain (yellow) (left panel). A DNA fragment containing 4×U1A and 14×DSR sequences[28] was inserted in the genome at about 60 kb away from A37 *lacO* insertion (Supplemental Table 1). Right panel shows percentile rank plots of the distance between the A37 loci in the first 3 substages. The dotted line at 0.67 µm on the Y axis indicates the threshold of pairing. Source data are provided as a Source Data file S7. **(b)** Double staining of A37-*lacO*/LacI-GFP with Seb1-mCherry. No Seb1-mCherry dot was formed at the A37 locus.

Supplementary Table 1. Insertion positions of *lacO* arrays in *lacO*/LacI-GFP library

Chromosome I		Chromosome II		Chromosome III	
**Name**	**Genomic position**	**Name**	**Genomic position**	**Name**	**Genomic position**
**A01**	50946	**B01**	110029	**C01**	42728
**A02**	94498	**B02**	159887	**C02**	113974
**A03**	217912	**B03**	277440	**C03**	214549
**A04**	272653	**B04**	343050	**C04**	319994
**A05**	325930	**B05**	449000	**C05**	397988
**A06**	425597	**B06**	539260	**C06**	505789
**A07**	463596	**B07**	636874	**C07**	558957
**A08**	553980	**B08**	739653	**C08**	666416
**A09**	639405	**B09**	828428	**C09**	756290
**A10**	667382	**B10**	934372	**C10**	853882
**A11**	765526	**B11**	1006688	**C11**	971822
**A12**	842624	**B12**	1028562	**C12**	1064740
**A13**	912607	**B13**	1136375	**C13**	1140428
**A14**	981422	**B14**	1224061	**C14**	1244477
**A15**	1027014	**B15**	1277831	**C15**	1316291
**A16**	1089958	**B16**	1363490	**C16**	1448223
**A17**	1210928	**B17**	1463491	**C17**	1524066
**A18**	1276130	**B18**	1554538	**C18**	1632149
**A19**	1329091	**B19**	1648000	**C19**	1727794
**A20**	1381967	**B20**	1727771	**C20**	1822398
**A21**	1485261	**B21**	1823968	**C21**	1928730
**A22**	1572568	**B22**	1915147	**C22**	2023785
**A23**	1664658	**B23**	1962938	**C23**	2107032
**A24**	1760902	**B24**	2074884	**C24**	2209985
**A25**	1866360	**B25**	2159853	**C25**	2335879
**A26**	1921920	**B26**	2254416	**C26**	2418000
**A27**	2052316	**B27**	2366555		
**A28**	2110800	**B28**	2470824		
**A29**	2215220	**B29**	2553418		
**A30**	2304577	**B30**	2647289		
**A31**	2385377	**B31**	2750390		
**A32**	2438381	**B32**	2847916		
**A33**	2527713	**B33**	2938954		
**A34**	2600539	**B34**	3040956		
**A35**	2669250	**B35**	3140566		
**A36**	2786827	**B36**	3237304		
**A37**	2882384	**B37**	3337527		
**A38**	2937494	**B38**	3516819		
**A39**	2996013	**B39**	3530977		
**A40**	3059909	**B40**	3615774		
**A41**	3184877	**B41**	3659380		
**A42**	3281070	**B42**	3762502		
**A43**	3404852	**B43**	3862500		
**A44**	3497734	**B44**	3925880		
**A45**	3559368	**B45**	3987400		
**A46**	3675200	**B46**	4110630		
**A47**	3739076	**B47**	4172323		
**A48**	3830016	**B48**	4255523		
**A49**	3920465	**B49**	4344090		
**A50**	4004001	**B50**	4403495		
**A51**	4106221				
**A52**	4196586				
**A53**	4299146				
**A54**	4450168				
**A55**	4510983				
**A56**	4577519				
**A57**	4648678				
**A58**	4701979				
**A59**	4782589				
**A60**	4852463				
**A61**	4964028				
**A62**	5024382				
**A63**	5096313				
**A64**	5177335				
**A65**	5256188				
**A66**	5393083				
**A67**	5479451				

Supplementary Table 2. Strains used in this study

**Figure 1a**	
YY982	h90 ade6-216 leu1 mei2^{+}-mCherry::HygR leu1^{+}::nmt1p-rcd1^{+}-YFP-FLAG-His6
YY630	h90 ade6-216 leu1 ura4 lys1 mei2^{+}-mCherry::HygR pTB46(seb1^{+}-GFP)
YY660	h90 ade6-216 leu1 mei2^{+}-mCherry::HygR leu1^{+}::nmt1p-nab3^{+}-YFP-FLAG-His6
YW372-1C	h90 ade6-216 leu1 lys1 ura4-D18 mei2^{+}-mCherry::HygR rmn1^{+}-GFP::KanR
YW371-1A	h90 ade6-216 leu1 lys1 ura4-D18 mei2^{+}-mCherry::HygR pab2^{+}-GFP::KanR
YY983	h90 ade6-216 leu1 mei2^{+}-mCherry::HygR leu1^{+}::nmt1p-rna15^{+}-YFP-FLAG-His6
YY984	h90 ade6-216 leu1 mei2^{+}-mCherry::HygR leu1^{+}::nmt1p-rhn1^{+}-YFP-FLAG-His6
YY990	h90 ade6-216 leu1 mei2^{+}-mCherry::HygR leu1^{+}::nmt1p-pcf11^{+}-YFP-FLAG-His6
YW370-1A	h90 ade6-216 leu1 lys1 ura4-D18 mei2^{+}-mCherry::HygR pla1^{+}-GFP::KanR
YY991	h90 ade6-216 leu1 mei2^{+}-mCherry::HygR leu1^{+}::nmt1p-ctf1^{+}-YFP-FLAG-His6
**Figure 1b**	
YY548-13C	ade6-216 leu1 ura4-D18 sme2proxy[::ura4^{+}- KanR-lacOp] his7^{+}::LacI-GFP
YW002	h90 ade6-216 leu1 ura4-D18 sme2proxy[::ura4^{+}- KanR-lacOp] his7^{+}::LacI-GFP Δrhn1:: NatR
YW012	h90 ade6-216 leu1 ura4-D18 sme2proxy[::ura4^{+}- KanR-lacOp] his7^{+}::LacI-GFP Δpab2:: NatR
YW373-4	h90 ade6-216 leu1 ura4-D18 sme2proxy[::ura4^{+}- KanR-lacOp] his7^{+}::LacI-GFP Δctf1:: HygR
YW079-5B	h90 ade6-216 leu1 ura4-DS/E sme2proxy[::ura4^{+}- KanR-lacOp] his7^{+}::LacI-GFP seb1-5FLAG- HygR seb1-E38
YW061	h90 ade6-216 leu1 ura4-D18 sme2proxy[::ura4^{+}- KanR-lacOp] his7^{+}::LacI-GFP lys1^{+}:: Padh15-skp1-NLS-AtTIR1-NLS pcf11^{+}-IAA17-mCherry:: HygR
YW162-3	h90 ade6-216 leu1 ura4-D18 sme2proxy[::ura4+- KanR-lacOp] his7^{+}::LacI-GFP lys1^{+}:: Padh15-skp1-NLS-AtTIR1-NLS rna15^{+}-IAA17-mCherry:: HygR
YW001	h90 ade6-216 leu1 ura4-D18 sme2proxy[::ura4^{+}- KanR-lacOp] his7^{+}::LacI-GFP Δrmn1:: NatR
YW005	h90 ade6-216 leu1 ura4-D18 sme2proxy[::ura4^{+}- KanR-lacOp] his7^{+}::LacI-GFP Δnab3:: NatR
YW008	h90 ade6-216 leu1 ura4-D18 sme2proxy[::ura4^{+}- KanR-lacOp] his7^{+}::LacI-GFP Δrcd1:: NatR
**Figure 1c**	
YY906	h90 ade6-216 leu1 ura4-D18 lys1 U1Atag-sme2^{+}::HygR aur1::Pnmt1-U1Ap-mCherry
YW006	h90 ade6-216 leu1 ura4-D18 lys1 U1Atag-sme2^{+}::HygR aur1::Pnmt1-U1Ap-mCherry Δpab2:: NatR
YW004	h90 ade6-216 leu1 ura4-D18 lys1 U1Atag-sme2^{+}::HygR aur1::Pnmt1-U1Ap-mCherry Δrhn1::NatR
YW283-16B	h90 ade6-216 leu1 ura4-DS/E U1Atag-sme2^{+}::HygR aur1::Pnmt1-U1Ap-mCherry seb1-5FLAG-HygR seb1-E38
YW375-5B	h90 ade6-216 leu1 ura4-D18 lys1 U1Atag-sme2^{+}:: HygR aur1::Pnmt1-U1Ap-mCherry Δctf1::HygR
YW007	h90 ade6-2106 leu1 ura4-D18 lys1 U1Atag-sme2^{+}::HygR aur1::Pnmt1-U1Ap-mCherry Δnab3:: NatR
YW009	h90 ade6-2106 leu1 ura4-D18 lys1 U1Atag-sme2^{+}::HygR aur1::Pnmt1-U1Ap-mCherry Δrcd1:: NatR
YW010	h90 ade6-2106 leu1 ura4-D18 lys1 U1Atag-sme2^{+}::HygR aur1::Pnmt1-U1Ap-mCherry Δrmn11::NatR
**Figure 2b-d**	

YW298	h90 ade6-210 leu1 ura4-D18 A55[::ura4^{+}- KanR-lacOp] his7^{+}::LacI-GFP seb1^{+}-mCherry:: HygR
YW260	h90 ade6-216 leu1 ura4-D18 C24[::ura4^{+} KanR -lacOp] his7^{+}::LacI-GFP seb1^{+}-mCherry:: HygR
YW309-13C	h90 ade6-210 leu1 ura4-D18 seb1^{+}-mCherry::HygR pab2^{+}-GFP::KanR sme2proxy[::ura4^{+}- KanR-lacOp] A55[::ura4^{+}- KanR-lacOp] C24[::ura4^{+}-KanR-lacOp] his7^{+}::lacI-GFP
**Figure 2e**	
YW145	h-/h- ade6-216/ade6-210 lys1/lys1^{+}::mat-Pc pat1::NatR/pat1::NatR pat1-as(L95A)::HygR/pat1-as(L95A)::HygR rhn1^{+}-GFP::Kanr/rhn1^{+}-GFP::KanR
YW146	h-/h- ade6-216/ade6-210 ura4/ura4^{+} lys1/lys1^{+}::mat-Pc pat1::NatR/pat1::NatR pat1-as(L95A)::HygR/pat1-as(L95A)::HygR pab2^{+}-GFP::Kanr/pab2^{+}-GFP::KanR
YW147	h-/h- ade6-216/ade6-210 lys1/lys1^{+}::mat-Pc pat1::NatR/pat1::NatR pat1-as(L95A)::HygR/pat1-as(L95A)::HygR seb1^{+}-GFP::Kanr/seb1^{+}-GFP::KanR
**Figure 2f-g**	
YW344-14B	h90 ade6-216 leu1 ura4 Δmei2::HygR A55[::ura4^{+}-KanR-lacOp] his7^{+}::lacI-GFP aur1-r::mei2^{+} seb1^{+}-mCherry::HygR
YW346-4	h90 ade6-210 leu1 ura4 seb1^{+}-mCherry::HygR A55[::ura4^{+}- KanR -lacOp] his7^{+}::lacI-GFP Δomt3::NatR
YW345-19B	h90 ade6-210 leu1 ura4 lys1 C24[::ura4^{+}-lacOP- KanR] his7^{+}::lacI-GFP seb1^{+}-mCherry::HygR Δski3::HygR
YW349	h90 ade6-210 leu1 ura4 lys1 C24[::ura4^{+}-lacOP- KanR] his7^{+}::lacI-GFP seb1^{+}-mCherry::HygR Δski3&SPNCRNA.584::NatR
**Figure 3**	
YY548-13C	h90 ade6-216 leu1 ura4-D18 sme2proxy[::ura4^{+}- KanR-lacOp] his7^{+}::LacI-GFP
YW289-5B	h90 ade6-216 leu1 lys1 ura4-D18 A55[::ura4^{+}- KanR-lacOp] his7^{+}::lacI-GFP
YW592-2	h90 ade6-216 leu1 ura4-D18 lys1 C24[::ura4^{+}- KanR-lacOp] his7^{+}::LacI-GFP
YW002	h90 ade6-216 leu1 ura4-D18 sme2proxy[::ura4^{+}- KanR-lacOp] his7^{+}::LacI-GFP Δrhn1:: NatR
YW079-8B	h90 ade6-216 leu1 ura4-DS/E seb1-5FLAG-HygR seb1-E38 sme2proxy[::ura4^{+}-KanR-lacOp] his7^{+}::LacI-GFP
YW012	h90 ade6-216 leu1 ura4-D18 sme2proxy[::ura4^{+}- KanR-lacOp] his7^{+}::LacI-GFP Δpab2:: NatR
YW299-4C	h90 ade6-216 leu1 lys1 ura4-D18 A55[::ura4^{+}- KanR-lacOp] his7^{+}::lacI-GFP Δrhn1:: HygR
YW302-4D	h90 ade6-216 leu1 lys1 ura4-D18 A55[::ura4^{+}- KanR-lacOp] his7^{+}::lacI-GFP seb1-5FLAG-HygR seb1-E38
YW414-18	h90 ade6-216 leu1 lys1 ura4-D18 A55[::ura4^{+}- KanR-lacOp] his7^{+}::lacI-GFP Δpab2:: HygR
YW199-24	h90 ade6-216 leu1 lys1 ura4-D18 C24[::ura4^{+}- KanR-lacOp] his7^{+}::lacI-GFP Δrhn1:: HygR
YW288-10C	h90 ade6-216 leu1 lys1 ura4-D18 C24[::ura4^{+}- KanR-lacOp] his7^{+}::lacI-GFP seb1-5FLAG-HygR seb1-E38
YW417-38	h90 ade6-216 leu1 lys1 ura4-D18 C24[::ura4^{+}- KanR-lacOp] his7^{+}::lacI-GFP Δpab2:: HygR
**Figure 4**	
YY548-13C	h90 ade6-216 leu1 ura4-D18 sme2proxy[::ura4^{+}- KanR-lacOp] his7^{+}::LacI-GFP
YW417	h90 ade6-210 leu1 ura4-D18 sme2proxy[::ura4^{+}- KanR-lacOp] his7^{+}::LacI-GFP Δrec12:: KanR
YY656-7B	h90 ade6-216 leu1 ura4-D18 sme2proxy[::ura4^{+}- KanR-lacOp] his7^{+}::LacI-GFP Δsme2:: HygR

YY665-16C	h90 ade6-216 leu1 ura4-D18 sme2proxy[::ura4^{+}- KanR-lacOp] his7^{+}::LacI-GFP Δsme2:: HygR rec12-152::LEU2
YW002	h90 ade6-216 leu1 ura4-D18 sme2proxy[::ura4^{+}- KanR-lacOp] his7^{+}::LacI-GFP Δrhn1:: NatR
YW079-5B	h90 ade6-216 leu1 ura4-DS/E sme2proxy[::ura4^{+}- KanR-lacOp] his7^{+}::LacI-GFP seb1-5FLAG- HygR seb1-E38
YW287-15D	h90 ade6-216 leu1 ura4-D18 sme2proxy[::ura4^{+}- KanR-lacOp] his7^{+}::LacI-GFP Δrhn1:: NatR seb1-5FLAG- HygR seb1-E38
YW289-5B	h90 ade6-216 leu1 lys1 ura4-D18 A55[::ura4^{+}- KanR-lacOp] his7^{+}::lacI-GFP
YW286-1	h90 ade6-216 leu1 lys1 ura4-D18 A55[::ura4^{+}- KanR-lacOp] his7^{+}::lacI-GFP rec12-152::LEU2
YW346-4	h90 ade6-216 leu1 ura4-D18 A55[::ura4^{+}- KanR-lacOp] his7^{+}::lacI-GFP Δomt3:: NatR
YW429	h90 ade6-210 leu1 ura4-D18 A55[::ura4^{+}- KanR-lacOp] his7^{+}::lacI-GFP Δomt3:: HygR rec12-152::LEU2
YW299-4C	h90 ade6-216 leu1 lys1 ura4-D18 A55[::ura4^{+}- KanR-lacOp] his7^{+}::lacI-GFP Δrhn1:: HygR
YW302-4D	h90 ade6-216 leu1 lys1 ura4-D18 A55[::ura4^{+}- KanR-lacOp] his7^{+}::lacI-GFP seb1-5FLAG- HygR seb1-ts125
YW334-30B	h90 ade6-216 leu1 lys1 ura4-D18 A55[::ura4^{+}- KanR-lacOp] his7^{+}::lacI-GFP Δrhn1:: HygR seb1-5FLAG- HygR seb1-ts125
YW592-2	h90 ade6-216 leu1 ura4-D18 lys1 C24[::ura4^{+}- KanR-lacOp] his7^{+}::LacI-GFP
YY792-2	h90 ade6-210 leu1 ura4-D18 lys1 C24[::ura4^{+}- KanR-lacOp] his7^{+}::LacI-GFP rec12-152::LEU2
YW351-4	h90 ade6-210 leu1 ura4-D18 lys1 C24[::ura4^{+}- KanR-lacOp] his7^{+}::LacI-GFP ΔlncRNA584:: NatR
YW430-8	h90 ade6-210 leu1 ura4-D18 lys1 C24[::ura4^{+}- KanR-lacOp] his7^{+}::LacI-GFP rec12-152::LEU2
YW350-2A	h90 ade6-210 leu1 ura4-D18 lys1 C24[::ura4^{+}- KanR-lacOp] his7^{+}::LacI-GFP Δrhn1:: HygR
YW288-2C	h90 ade6-216 leu1 lys1 ura4-DS/E C24[::ura4^{+}- KanR-lacOp] his7^{+}::lacI-GFP seb1-5FLAG- HygR seb1-E38
YW291-4B	h90 ade6-216 leu1 lys1 ura4-D18 C24[::ura4^{+}- KanR-lacOp] his7^{+}::lacI-GFP Δrhn1:: HygR seb1-5FLAG- HygR seb1-E38
**Figure 5**	
YW096-6B	h90 ade6-216 leu1 lys1 ura4-D18 seb1^{+}-mCherry::HygR
YY906-1	h90 ade6-210 leu1 lys1 ura4-D18 U1Atag-sme2::HygR aur1::Pnmt1-U1Ap-mCherry
YW289-5B	h90 ade6-216 leu1 lys1 ura4-D18 A55[::ura4^{+}- KanR-lacOp] his7^{+}::lacI-GFP
YW286-1	h90 ade6-216 leu1 lys1 ura4-D18 A55[::ura4^{+}- KanR-lacOp] his7^{+}::lacI-GFP rec12-152::LEU2
YY548-13C	h90 ade6-216 leu1 ura4-D18 sme2proxy[::ura4^{+}- KanR-lacOp] his7^{+}::LacI-GFP
YW417	h90 ade6-210 leu1 ura4-D18 sme2proxy[::ura4^{+}- KanR-lacOp] his7^{+}::LacI-GFP ΔRec12:: KanR
YW592-2	h90 ade6-216 leu1 ura4-D18 lys1 C24[::ura4^{+}- KanR-lacOp] his7^{+}::LacI-GFP
YW430-8	h90 ade6-210 leu1 ura4-D18 lys1 C24[::ura4^{+}- KanR-lacOp] his7^{+}::LacI-GFP rec12-152::LEU2
**Figure 6**	
YW289-5B	h90 ade6-216 leu1 lys1 ura4-D18 A55[::ura4^{+}- KanR-lacOp] his7^{+}::lacI-GFP
YW346-4	h90 ade6-216 leu1 ura4-D18 A55[::ura4^{+}- KanR-lacOp] his7^{+}::lacI-GFP Δomt3:: NatR
YW445-2	h90 ade6-210 leu1 ura4-D18 lys1 A55[::ura4^{+}- KanR-lacOp] his7^{+}::lacI-GFP Δsme2:: HygR Δomt3:: sme2^{+}-NatR

YW441-1B	*hm ade6-210 leu1 ura4-D18 lys1 A55[::ura4^{+}- KanR-lacOp] his7^{+}::lacI-GFP Δsme2:: HygR*
YW446-2D	*hp ade6-210 leu1 ura4-D18 lys1 A55[::ura4^{+}- KanR-lacOp] his7^{+}::lacI-GFP Δsme2:: HygR Δomt3:: sme2^{+}-NatR*
**Supplementary Figure 1**	
YY831-7B	*h90 ade6-210 leu1 lys1 seb1^{+}-GFP::KanR*
YW055-10	*h90 ade6-216 leu1 lys1 rhn1^{+}-GFP::KanR*
YW097-4B	*h90 ade6-216 leu1 lys1 ura4-D18 seb1^{+}-mCherry::HygR pab2^{+}-GFP::KanR*
YW364-1	*h90 ade6-216 leu1 lys1 ura4-D18 seb1^{+}-mCherry::HygR rna15^{+}-GFP::KanR*
YW096-5C	*h90 ade6-216 leu1 lys1 ura4-D18 seb1^{+}-mCherry::HygR rhn1^{+}-GFP::KanR*
YW365-2	*h90 ade6-216 leu1 lys1 ura4-D18 seb1^{+}-mCherry::HygR pcf11^{+}-GFP::KanR*
YW369-1	*h90 ade6-216 leu1 lys1 ura4-D18 seb1^{+}-mCherry::HygR ctf1^{+}-GFP::KanR*
**Supplementary Figure 2**	
YW061	*h90 ade6-216 leu1 ura4-D18 sme2proxy[::ura4^{+}- KanR-lacOp] his7^{+}::LacI-GFP lys1^{+}:: Padh15-skp1-NLS-AtTIR1-NLS pcf11^{+}-IAA17-mCherry:: HygR*
YW162-3	*h90 ade6-216 leu1 ura4-D18 sme2proxy[::ura4+- KanR-lacOp] his7^{+}::LacI-GFP lys1^{+}:: Padh15-skp1-NLS-AtTIR1-NLS rna15^{+}-IAA17-mCherry:: HygR*
EON125	*hp ade6-210 otrR(SphI)::ura4+ leu1-32 ura4-DS/E seb1-5Flag-hphMX6 seb1-E38*
L968	*h90*
**Supplementary Figure 4**	
YW448-9A	*h90 ade6-210 leu1 lys1 seb1^{+}-GFP::KanR Δsme2::ura4+ Δomt3:: KanR ΔlncRNA584:: HygR*
**Supplementary Figure 6**	
PY183	*h90 ade6-216 leu1-32 rec8^{+}::GFP-KanR*
**Supplementary Figure 7**	
YY699-C6	*h90 ade6-216 leu1 lys1 ura4-D18 A37[::ura4^{+}- KanR-lacOp] his7^{+}::lacI-GFP*
YW388-2	*h90 ade6-216 leu1 lys1 ura4-D18 A37[::ura4^{+}- KanR-lacOp] his7^{+}::lacI-GFP C02::4XU1A-14DSR-NatR*
YW404-22	*h90 ade6-216 leu1 lys1 ura4-D18 A37[::ura4^{+}- KanR-lacOp] his7^{+}::lacI-GFP C02::4XU1A-14DSR-NatR seb1^{+}-mCherry::HygR*
**Video 1**	
YW096-6B	*h90 ade6-216 leu1 lys1 ura4-D18 seb1^{+}-mCherry::HygR*
**Video 2**	
YW055-10	*h90 ade6-216 leu1 lys1 ura4-D18 rhn1^{+}-GFP::KanR*
**Video 3**	
YW052-1	*h90 ade6-216 leu1 lys1 ura4-D18 rna15^{+}-GFP::KanR*
**Video 4**	
YY906-1	*h90 ade6-210 leu1 lys1 ura4-D18 U1Atag-sme2::HygR aur1::Pnmt1-U1Ap-mCherry*
**Video 5**	
YW289-5B	*h90 ade6-216 leu1 lys1 ura4-D18 A55[::ura4^{+}- KanR-lacOp] his7^{+}::lacI-GFP*
**Video 6**	
YW592-2	*h90 ade6-216 leu1 ura4-D18 lys1 C24[::ura4^{+}- KanR-lacOp] his7^{+}::LacI-GFP*

All strains are constructed in this study, except for PY183 (Watanabe & Nurse, 1999).

Supplementary Table 3. List of primers for gene disruption

mei2 deletion
Forward1: GCATTGAGCCGTCATTGATC
Reverse1: cggcggggacgaggcaagctTTACGAAAGAGATCCTGTATTG
Forward2: gatactaacgccgccatccagtTTATGTGTTAAAACGGCAACAA
Reverse2: CTAACAACCATCTAACCCCTTCC

omt3 deletion
Forward: CGCCGCATTCGATTTTAATGTATCACGTATAAATCGGTAAATATGTGTGTACCACGGT TGTAGAAGTGCTCGAAGACAAagcttgcctcgtccccgccg
Reverse: CCTAATTTTTAAAAGTTTATCGACTTGGCTTCAACACATAGTGCTTCTATAAGTAACA AACATGTTTTAAAACAAGATAactggatggcggcgttagt

ski3 deletion
Forward1: CACTCCTTGGGTTCTCTATC
Reverse1: cggcggggacgaggcaagctGACAATGGCAGTTTATTATG
Forward2: gatactaacgccgccatccaGAACTTGTATTTATTATCAA
Reverse2: GAATCTGACAGTTCATTAGC

ski3-lncRNA584 deletion
Forward1: CACTCCTTGGGTTCTCTATC
Reverse1: cggcggggacgaggcaagctGACAATGGCAGTTTATTATG
Forward2: gatactaacgccgccatccaGAACTTGTATTTATTATCAA
Reverse2: AACCTTTTACTTGATTATAAGCGCCAATAATTGGCTGTTTGAATTTCAACCCACTGC TAAATCGATGACGAATTACGCTGactggatggcggcgttagt

rhn1 deletion
Forward: TTTATTACTTATGCTTATGATTTCGTTTGCGATGCTAACAGCAGTGTGTCGTAAGAAA CTCACCATCAGCATAAAACAAGagcttgcctcgtccccgccg
Reverse: TGACTAAGACTGCCACATTGATTGACAGTTTAAAAAAAAAAAGAAAAATAATGAAAAC TAAGCATTACATTTCATGGATTactggatggcggcgttagta

pab2 deletion
Forward1: TATAAGCAGGCAGTCCAAATCCT
Reverse1: ttaattaacccggggatccgTTTTTCAAATTTTCTGCTAC
Forward2: gatactaacgccgccatccaGTAATCGCTTTGATGACTT
Reverse2: GAATTACATTATGTCAGCATAT

ctf1 deletion
Forward1: GTACTTACCCATGAATTGTCCC
Reverse1: cggcggggacgaggcaagctGCTGGTAGTCGCGTCTTTCG
Forward2: gatactaacgccgccatccaGTCTAATTTAGTAATATCA
Reverse2: GTTGATGTTACCACCTAC

rcd1 deletion
Forward1: ACGCATAGCACGAGGACTGAAATT
Reverse1: ttaattaacccggggatccgTTTTTCTAGAATGCGGGAAA
Forward2: gatactaacgccgccatccaCTGCCCTTCGTTTCCTATCTG
Reverse2: ATCAGAGTAAGTTCTGTCCT

nab3 deletion
Forward1: CTTTACACATTTGGTTGAGTGT
Reverse1: ttaattaacccggggatccgTTATTGATTGACTTGAAAAG
Forward2: gatactaacgccgccatccaCAAGTGTTTTAGGTAGCAC
Reverse2: CAGTAAAAATCAAGTATCGAAAC

rmn1 deletion
Forward1: TCTCATAATCGTCAGAGTTTGA
Reverse1: ttaattaacccggggatccgTGTGATGGAGCAAAGTGCT
Forward2: gatactaacgccgccatccaCTGCATTGTATGCGGCTGTC
Reverse2: CATGAGTTTTCTCTCCTAGCCG

Supplementary Table 4. List of smFISH probes

sme2_1	atacccactaagtctgttta	ncRNA130_1	gttattaatgcttccttctt	ncRNA584_1	cgttctatcctaagagtgga
sme2_2	cggcagaagattgaccaaca	ncRNA130_2	gaattcccaacctcttttta	ncRNA584_2	tgctgacactctacgatcat
sme2_3	gcatattccgtcttacaata	ncRNA130_3	cgcttttactctttttttgt	ncRNA584_3	tatagtgccacgaatcgttc
sme2_4	accaactaaagcgatcttgc	ncRNA130_4	atctcttatacatgcataca	ncRNA584_4	tctgcactttttgagctttt
sme2_5	gaccatttcaaaatgttgca	ncRNA130_5	tcaaaaaccatcatcagcct	ncRNA584_5	gcggcataaatattgtccac
sme2_6	taccgaatccagctttttga	ncRNA130_6	agcaacatcgctaatgatca	ncRNA584_6	cagattggcaatatccggat
sme2_7	cagagcttagaagacaaggt	ncRNA130_7	ttcactgactctcgctttac	ncRNA584_7	caatccaaccactcactcaa
sme2_8	taactggaccccatcaagaa	ncRNA130_8	actcagcaaagctttaatct	ncRNA584_8	cttacctagagccatttttt
sme2_9	taaaccaacttgggggttgg	ncRNA130_9	tacgaaacgcattgtcccaa	ncRNA584_9	tgctttaccgtgtacgaatc
sme2_10	tctaagctactattcatcca	ncRNA130_10	acaattcgtcacgtacatca	ncRNA584_10	gatgcttcgtaggaacttgt
sme2_11	agtagattccatcagtcata	ncRNA130_11	tcaggccttcatttttttta	ncRNA584_11	actcatgaggcactagtttc
sme2_12	tgcagccaaaaagtgtacca	ncRNA130_12	accatagagatagaagcggt	ncRNA584_12	tcagtcctatcgtttgcaaa
sme2_13	cattgtaagtgctttcaagg	ncRNA130_13	gttctttcctcaaattctca	ncRNA584_13	cttcacgaccagacgatttg
sme2_14	ttcagtcattcgcaaagttt	ncRNA130_14	acaagattcctcttaggttt	ncRNA584_14	gcatttgctactacgttgta
sme2_15	agtcgttttatttcttttct	ncRNA130_15	gtcatgttgatttttcatcc	ncRNA584_15	accgttatgcttcaaagtct
sme2_16	gtttcaacaatagttcaggt	ncRNA130_16	gtttcgcttccaagatgtat	ncRNA584_16	gcttctagttggactttact
sme2_17	tctgtttcaggaatacgttt	ncRNA130_17	aagctcttcaacttcctttc	ncRNA584_17	ctgtatgctctgcgattgaa
sme2_18	tgtttcgcatcaaactttca	ncRNA130_18	aattcctttgctgactgaca	ncRNA584_18	gtcttggtgcaatagctatt
sme2_19	gcgtttaaacaaactgcggg	ncRNA130_19	aaggtttctttcacgtcact	ncRNA584_19	caagccgcaagggaacaatt
sme2_20	tggtttcagcacgttttcaa	ncRNA130_20	ttctcgagaagcatgaagca	ncRNA584_20	agtatccaaggtgttatggg
sme2_21	ttggtttgcagggtttaacg	ncRNA130_21	ataaaaacccaagaccgcct	ncRNA584_21	tgagcgattcaaatgccgac
sme2_22	tgaattagcttgctgtggtt	ncRNA130_22	ccaaatatcaaatgggacct	ncRNA584_22	ttaaacggactacccttgga
sme2_23	ccaatgcaacataccattgt	ncRNA130_23	ttaggtttcactgcagcaac	ncRNA584_23	gatgcaaaagccttactcgg
sme2_24	aagagggagcacagcacaac	ncRNA130_24	ggtgagacttagcttacgag	ncRNA584_24	cttccttactagaacacctt
sme2_25	atgtgttgctattctcttct	ncRNA130_25	aacctcctgtttgcataaga	ncRNA584_25	tcttggggaaaatccaaccg
sme2_26	gaaggcatgttgaatttgga	ncRNA130_26	acatggatcaatcatccatt	ncRNA584_26	catgcagtaaacaccgagga
sme2_27	attaatagctgttccttgcg			ncRNA584_27	ttggatctattcggaaggca
sme2_28	ccttaaagtgtgttttaagc			ncRNA584_28	ttctatctctattgacccag
