# Supplementary material for: Chromosome-associated RNA–protein complexes promote pairing of homologous chromosomes during meiosis in Schizosaccharomyces pombe
Source: Nat Commun. 2019 Dec 6;10:5598. doi: 10.1038/s41467-019-13609-0 (PMC6898681; doi:10.1038/s41467-019-13609-0)
Supplement: Supplementary file 3 — Description of Additional Supplementary Files [file 41467_2019_13609_MOESM3_ESM.pdf]

Description of Additional Supplementary Files

File Name: Supplementary Data 1
Description: ChIP-seq data of three Smp proteins in meiotic and vegetative growth.

File Name: Supplementary Movie 1
Description: Disappearance of Seb1-mCherry dots in the meiotic prophase nucleus upon 1,6-hexanediol treatment.

File Name: Supplementary Movie 2
Description: Disappearance of Rhn1-GFP dots in the meiotic prophase nucleus upon 1,6-hexanediol treatment.

File Name: Supplementary Movie 3
Description: Disappearance of Rna15-GFP dots in the meiotic prophase nucleus upon 1,6-hexanediol treatment.

File Name: Supplementary Movie 4
Description: Scattering of sme2-RNA focus upon 1,6-hexanediol treatment.

File Name: Supplementary Movie 5
Description: Separation of the paired A55 locus upon 1,6-hexanediol treatment.

File Name: Supplementary Movie 6
Description: Separation of the paired C24 locus upon 1,6-hexanediol treatment.
