# Supplementary material for: Chromosome-associated RNA–protein complexes promote pairing of homologous chromosomes during meiosis in Schizosaccharomyces pombe
Source: Nat Commun. 2019 Dec 6;10:5598. doi: 10.1038/s41467-019-13609-0 (PMC6898681; doi:10.1038/s41467-019-13609-0)
Supplement: Supplementary file 4 — Supplementary Data 1 [file 41467_2019_13609_MOESM4_ESM.pdf]

I_1_1

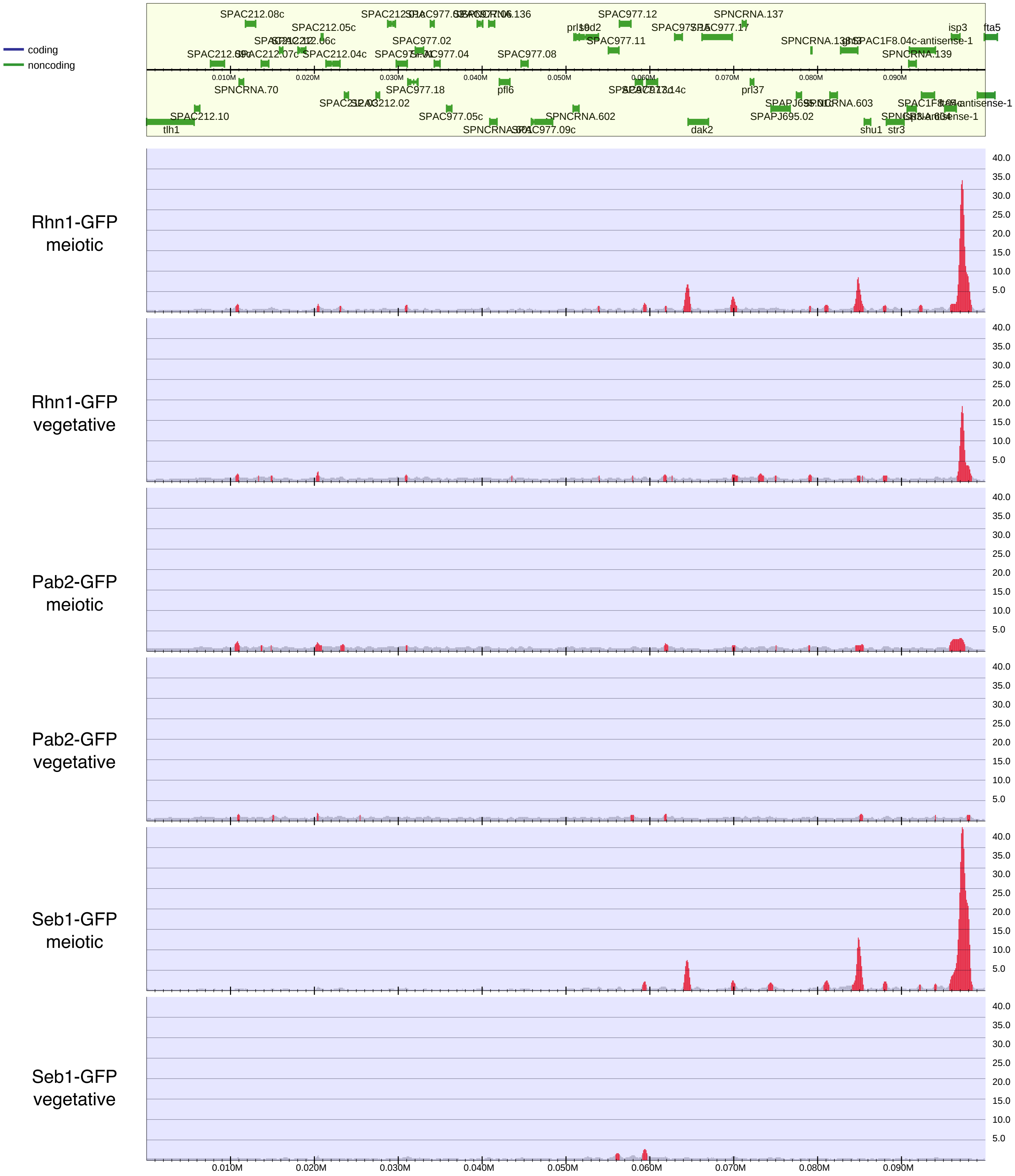
coding
noncoding
SPAC212.08c
SPAC212.05c
SPAC977.02
SPAC977.08
SPAC977.12
SPAC977.11
SPNCRNA.137
SPNCRNA.139
isp3
fta5
SPNCRNA.70
SPAC977.18
pfl6
prl37
SPAC212.10
tlh1
SPAC977.05c
SPNCRNA.602
dak2
SPAPJ695.02
shu1
str3
0.010M
0.020M
0.030M
0.040M
0.050M
0.060M
0.070M
0.080M
0.090M
40.0
35.0
30.0
25.0
20.0
15.0
10.0
5.0
Rhn1-GFP meiotic
Rhn1-GFP vegetative
Pab2-GFP meiotic
Pab2-GFP vegetative
Seb1-GFP meiotic
Seb1-GFP vegetative

I_1_2

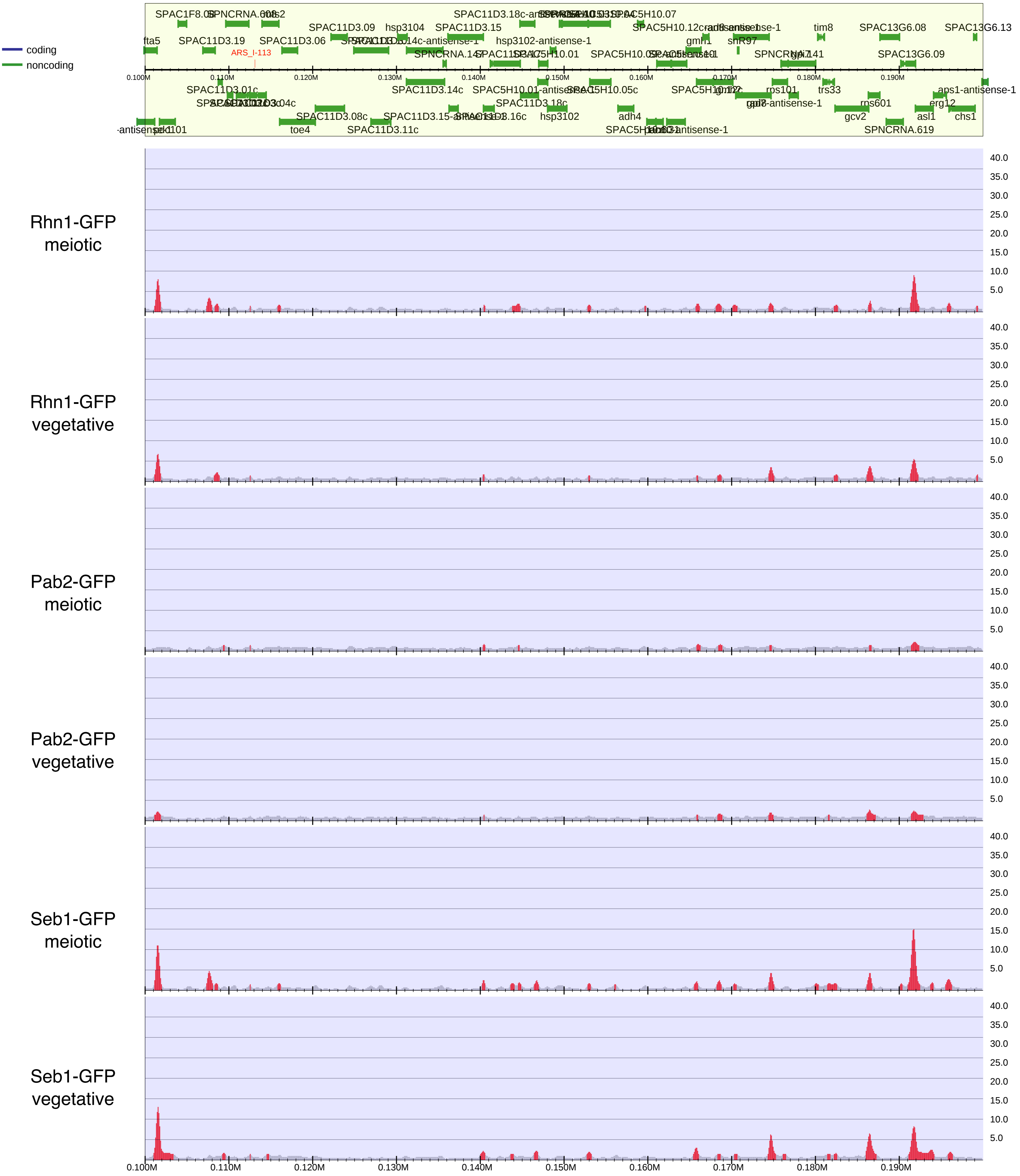
coding
noncoding
Rhn1-GFP meiotic
Rhn1-GFP vegetative
Pab2-GFP meiotic
Pab2-GFP vegetative
Seb1-GFP meiotic
Seb1-GFP vegetative
0.100M
0.110M
0.120M
0.130M
0.140M
0.150M
0.160M
0.170M
0.180M
0.190M
40.0
35.0
30.0
25.0
20.0
15.0
10.0
5.0
fta5
SPAC11D3.19
SPAC11D3.06
SPAC11D3.09
hsp3104
SPAC11D3.15
ARS_I-113
SPAC11D3.14c
SPAC11D3.18c
SPAC5H10.01
SPAC5H10.05c
SPAC5H10.07
hsp3102
hsp3102-antisense-1
adh4
gmh1
snR97
tim8
trs33
rps101
rps601
gcv2
erg12
asl1
chs1
SPAC13G6.08
SPAC13G6.09
SPAC13G6.13
aps1-antisense-1
SPNCRNA.619
SPAC11D3.08c
SPAC11D3.11c
toe4
SPAC11D3.01c

I_1_3

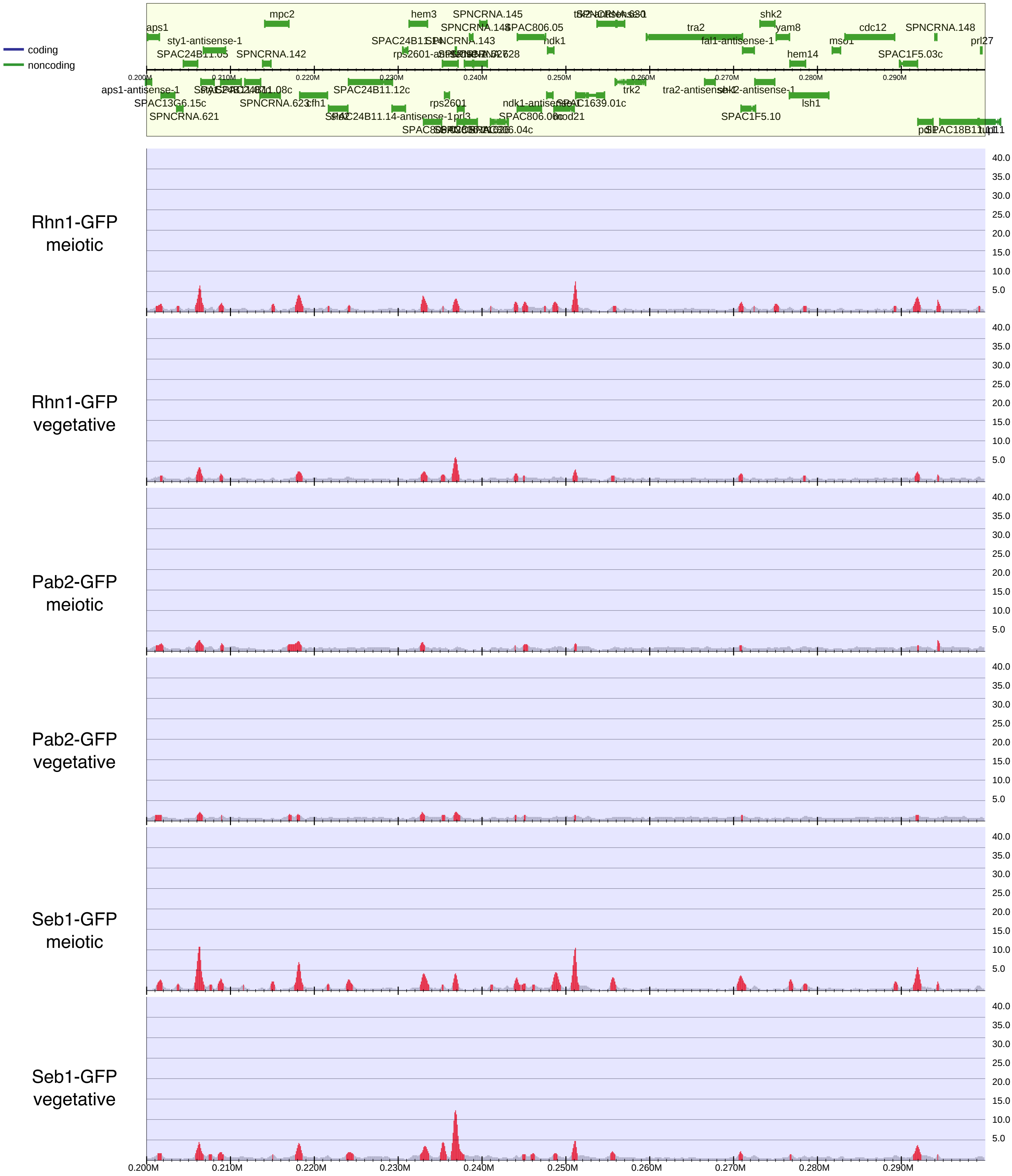
coding
noncoding
mpc2
hem3
SPNCRNA.145
shk2
aps1
tra2
yam8
cdc12
SPNCRNA.148
sty1-antisense-1
ndk1
fal1-antisense-1
prl27
SPNCRNA.142
hem14
SPAC1F5.03c
SPAC24B11.12c
trk2
lsh1
SPAC13G6.15c
SPNCRNA.621
rps2601
SPAC1F5.10
Rhn1-GFP
meiotic
Rhn1-GFP
vegetative
Pab2-GFP
meiotic
Pab2-GFP
vegetative
Seb1-GFP
meiotic
Seb1-GFP
vegetative
40.0
35.0
30.0
25.0
20.0
15.0
10.0
5.0
0.200M
0.210M
0.220M
0.230M
0.240M
0.250M
0.260M
0.270M
0.280M
0.290M

I_1_4

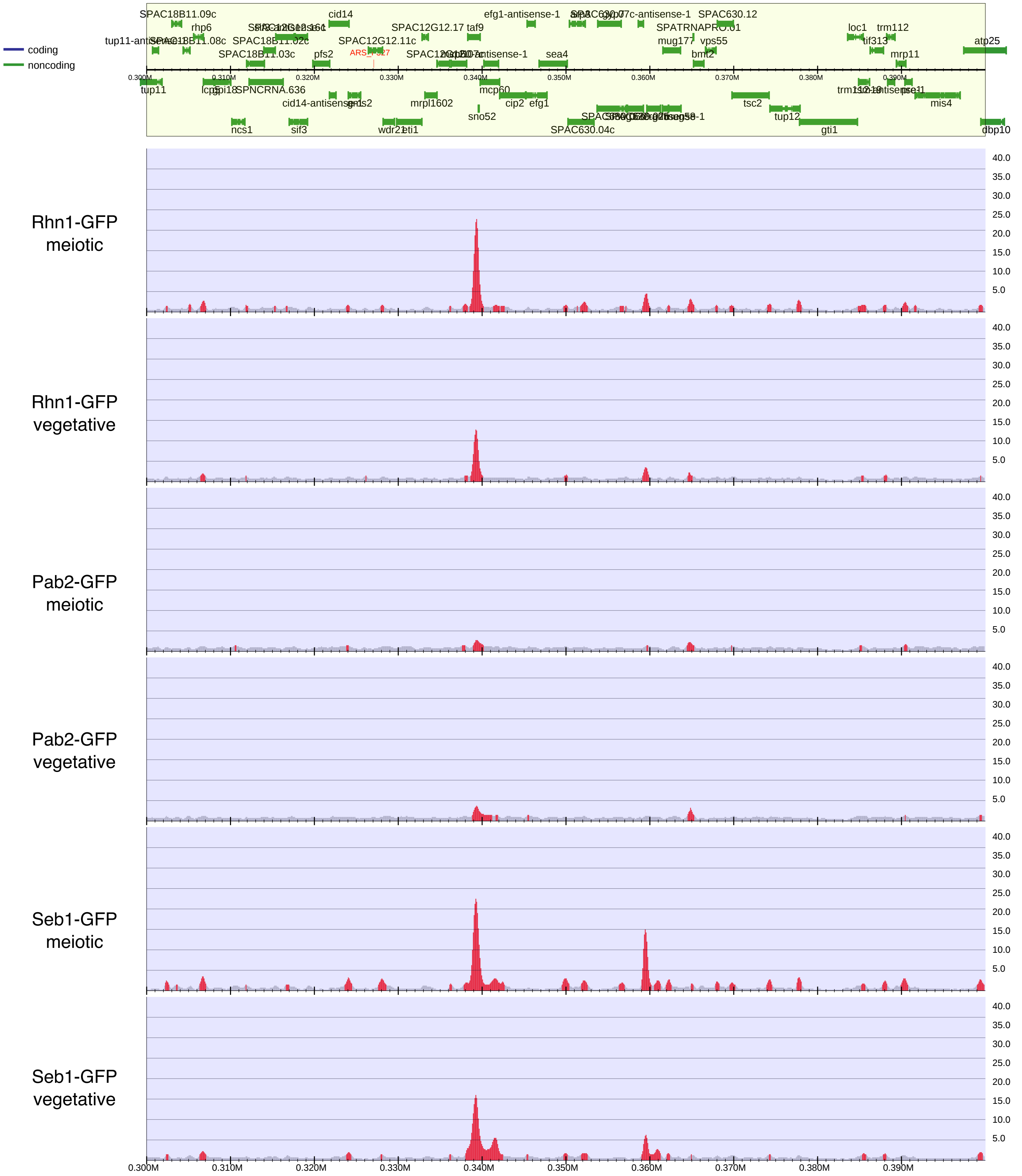
coding
noncoding
SPAC18B11.09c
rhp6
cid14
efg1-antisense-1
SPAC630.12
SPAC12G12.17
taf9
SPATRNAPRO.01
loc1
trm112
SPAC18B11.08c
SPAC18B11.02c
SPAC12G12.11c
mug177
vps55
tif313
atp25
SPAC18B11.03c
pfs2
ARS_I_327
sea4
bmt2
mrp11
tup11
SPNCRNA.636
mcp60
tsc2
mis4
cip2
efg1
mrpl1602
sno52
tup12
ncs1
sif3
wdr21
eti1
SPAC630.04c
gti1
dbp10
0.300M
0.310M
0.320M
0.330M
0.340M
0.350M
0.360M
0.370M
0.380M
0.390M
Rhn1-GFP meiotic
Rhn1-GFP vegetative
Pab2-GFP meiotic
Pab2-GFP vegetative
Seb1-GFP meiotic
Seb1-GFP vegetative
40.0
35.0
30.0
25.0
20.0
15.0
10.0
5.0

I_1_5

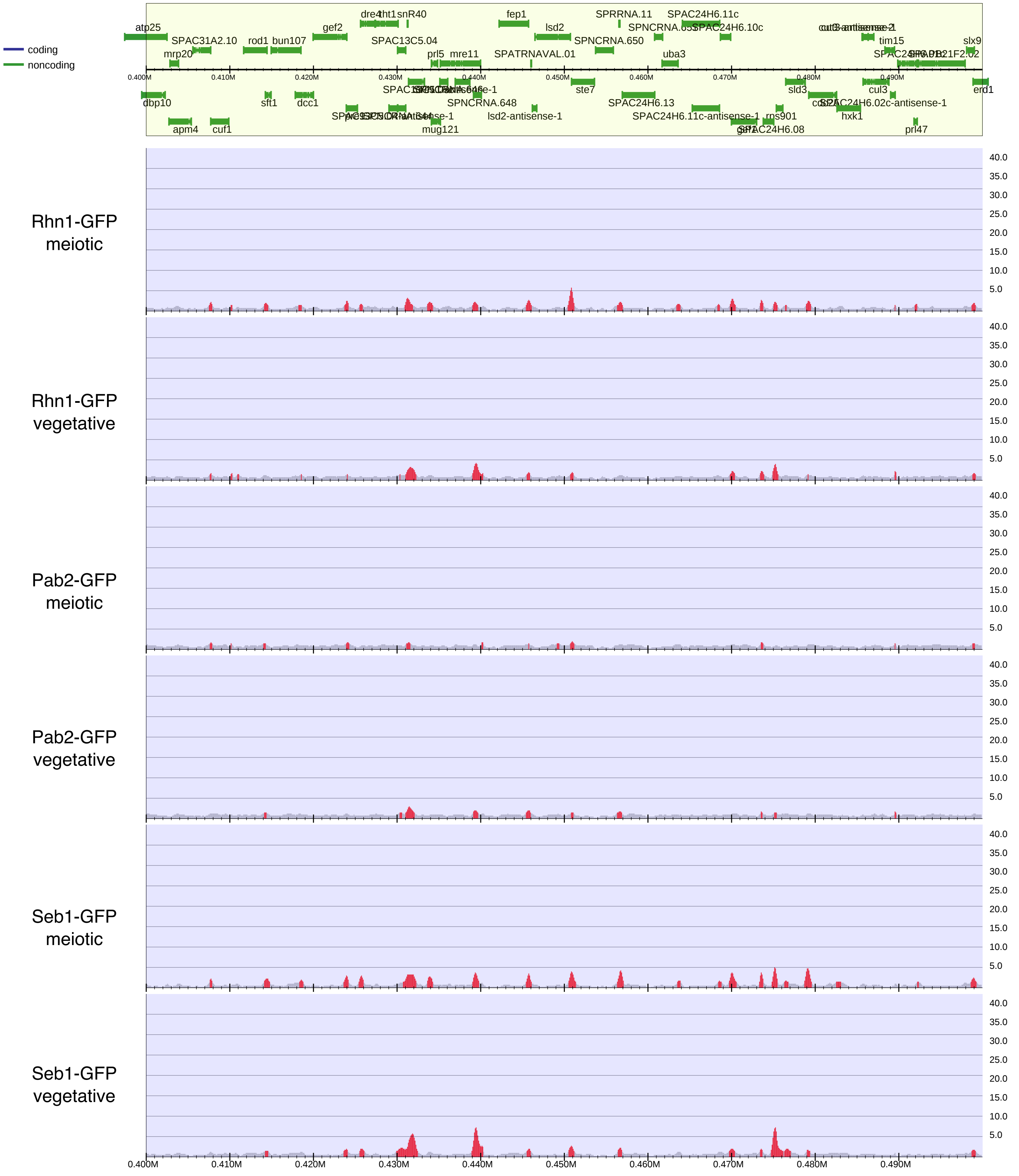
coding
noncoding
Rhn1-GFP meiotic
Rhn1-GFP vegetative
Pab2-GFP meiotic
Pab2-GFP vegetative
Seb1-GFP meiotic
Seb1-GFP vegetative
0.400M
0.410M
0.420M
0.430M
0.440M
0.450M
0.460M
0.470M
0.480M
0.490M

I_1_6

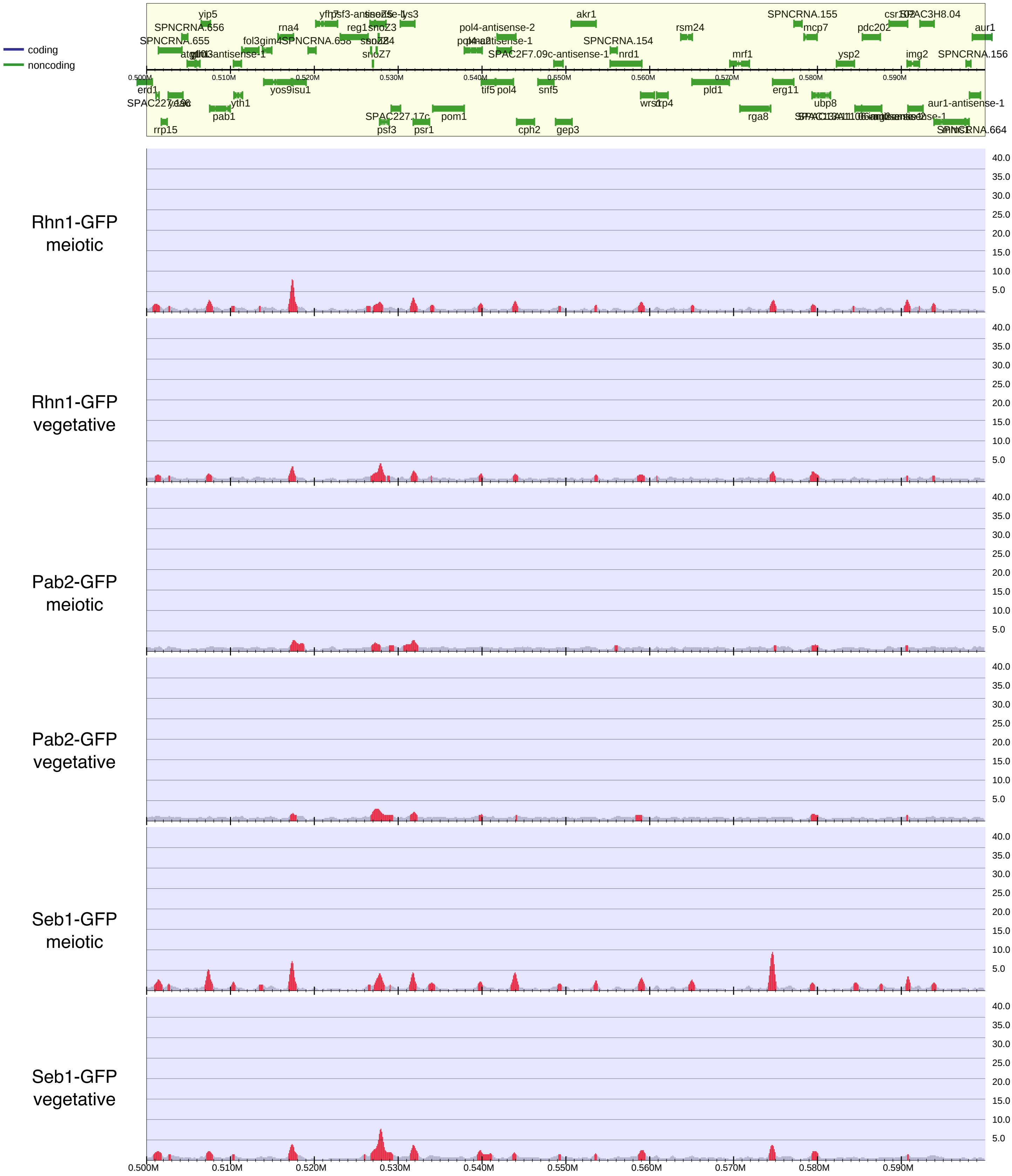
coding
noncoding
Rhn1-GFP meiotic
Rhn1-GFP vegetative
Pab2-GFP meiotic
Pab2-GFP vegetative
Seb1-GFP meiotic
Seb1-GFP vegetative
0.500M
0.510M
0.520M
0.530M
0.540M
0.550M
0.560M
0.570M
0.580M
0.590M
40.0
35.0
30.0
25.0
20.0
15.0
10.0
5.0
yip5
SPNCRNA.656
SPNCRNA.655
rna4
akr1
rsm24
SPNCRNA.155
mcp7
pdc202
aur1
SPNCRNA.154
nrd1
mrf1
ysp2
img2
SPNCRNA.156
erd1
yos9
isu1
tif5
pol4
snf5
pld1
erg11
ubp8
aur1-antisense-1
pab1
yth1
pom1
rga8
rrp15
psf3
psr1
cph2
gep3
pol4-antisense-2
reg1
snoZ3
snoZ7
lys3
fol3
gim4

I_1_7

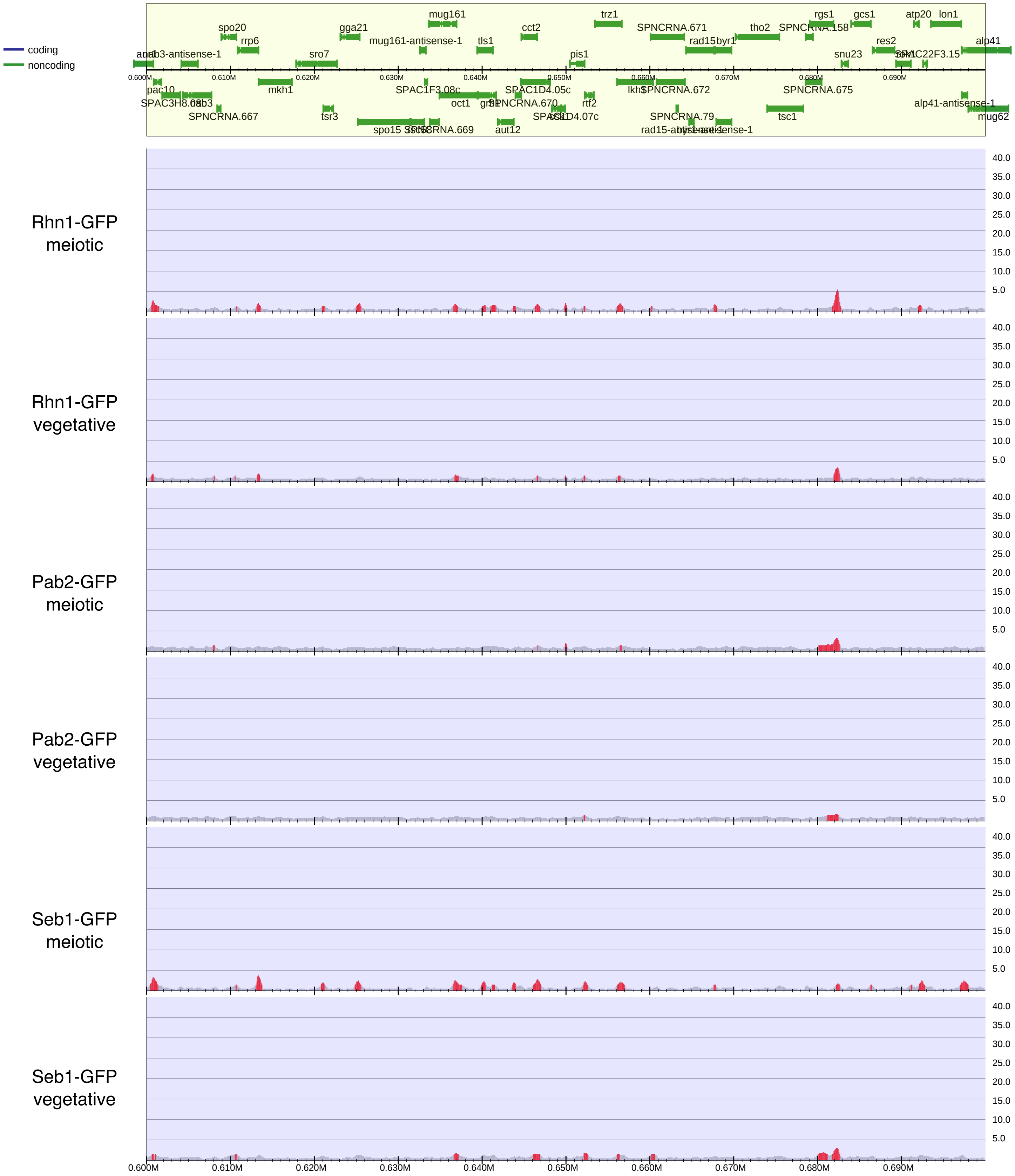

coding
noncoding
Rhn1-GFP meiotic
Rhn1-GFP vegetative
Pab2-GFP meiotic
Pab2-GFP vegetative
Seb1-GFP meiotic
Seb1-GFP vegetative
40.0
35.0
30.0
25.0
20.0
15.0
10.0
5.0
0.600M
0.610M
0.620M
0.630M
0.640M
0.650M
0.660M
0.670M
0.680M
0.690M

I_1_8

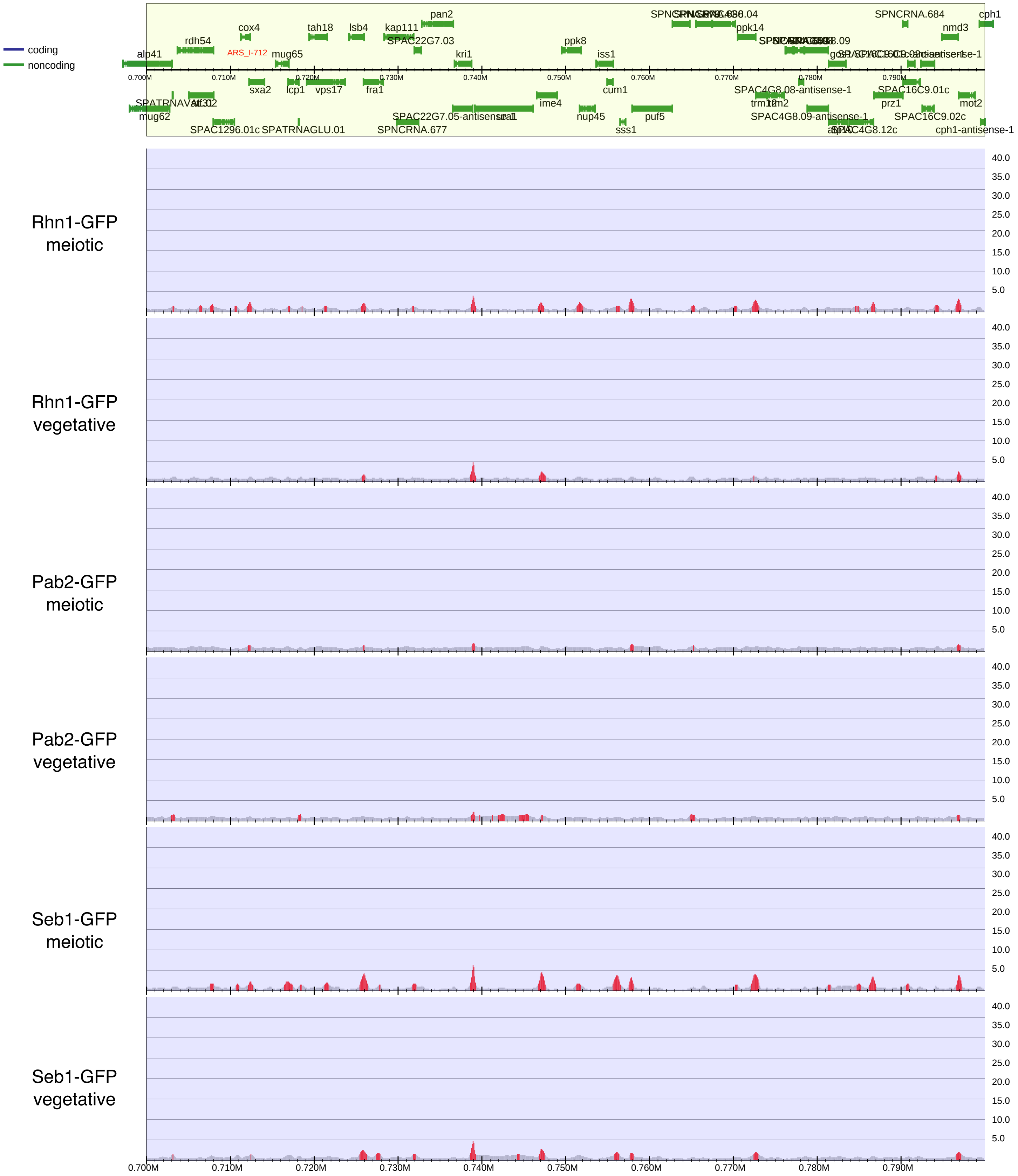
coding
noncoding
alp41
rdh54
cox4
ARS_I-712
mug65
tah18
lsb4
kap111
SPAC22G7.03
pan2
kri1
ppk8
iss1
ppk14
SPNCRNA.684
nmd3
cph1
0.700M
0.710M
0.720M
0.730M
0.740M
0.750M
0.760M
0.770M
0.780M
0.790M
sxa2
lcp1
vps17
fra1
cum1
SPAC4G8.08-antisense-1
SPAC16C9.01c
ime4
prz1
mot2
mug62
SPAC22G7.05-antisense-1
nup45
puf5
SPAC4G8.09-antisense-1
SPAC16C9.02c
SPAC1296.01c
SPATRNAGLU.01
SPNCRNA.677
sss1
SPAC4G8.12c
cph1-antisense-1
Rhn1-GFP meiotic
Rhn1-GFP vegetative
Pab2-GFP meiotic
Pab2-GFP vegetative
Seb1-GFP meiotic
Seb1-GFP vegetative
40.0
35.0
30.0
25.0
20.0
15.0
10.0
5.0

I_1_9

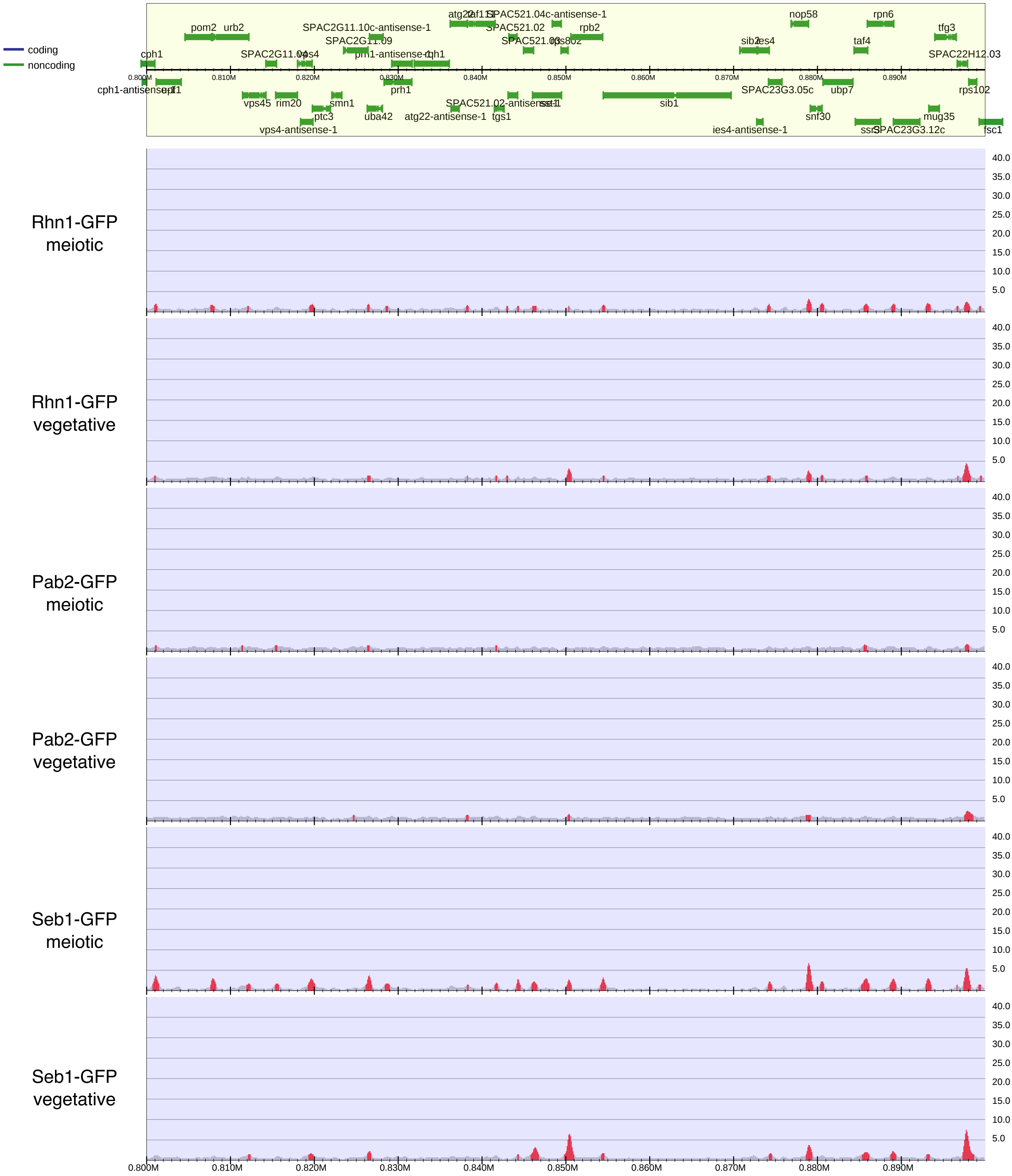
coding
noncoding
pom2
urb2
cph1
SPAC2G11.10c-antisense-1
SPAC2G11.09
SPAC521.04c-antisense-1
SPAC521.02
rpb2
nop58
rpn6
tfg3
taf4
SPAC22H12.03
prh1
vps45
rim20
smn1
ptc3
uba42
vps4-antisense-1
atg22-antisense-1
tgs1
sib1
SPAC23G3.05c
ubp7
rps102
snf30
mug35
ies4-antisense-1
SPAC23G3.12c
fsc1
Rhn1-GFP
meiotic
Rhn1-GFP
vegetative
Pab2-GFP
meiotic
Pab2-GFP
vegetative
Seb1-GFP
meiotic
Seb1-GFP
vegetative
40.0
35.0
30.0
25.0
20.0
15.0
10.0
5.0
0.800M
0.810M
0.820M
0.830M
0.840M
0.850M
0.860M
0.870M
0.880M
0.890M

I_1_10

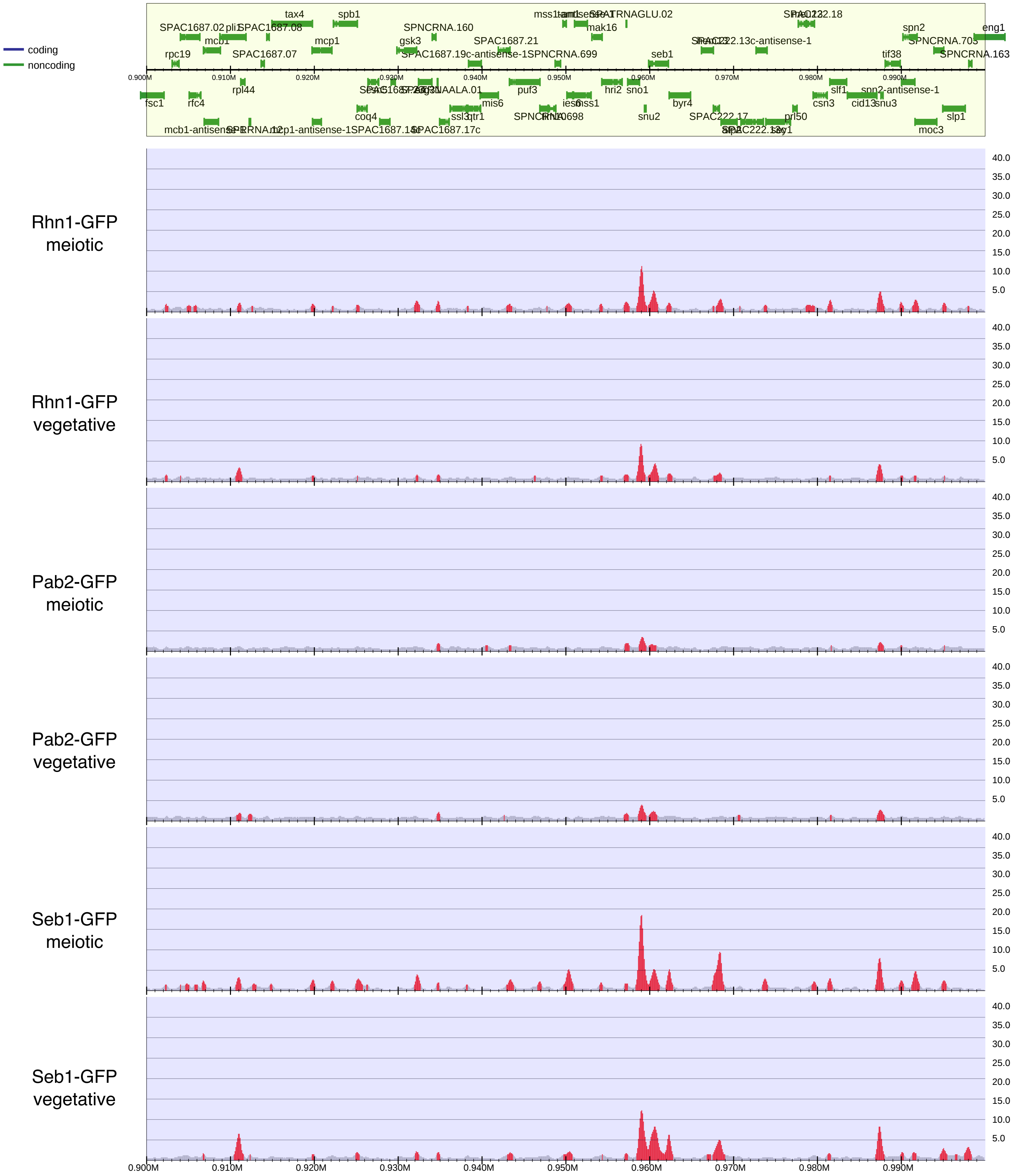
coding
noncoding
Rhn1-GFP meiotic
Rhn1-GFP vegetative
Pab2-GFP meiotic
Pab2-GFP vegetative
Seb1-GFP meiotic
Seb1-GFP vegetative
0.900M
0.910M
0.920M
0.930M
0.940M
0.950M
0.960M
0.970M
0.980M
0.990M
40.0
35.0
30.0
25.0
20.0
15.0
10.0
5.0

I_1_11

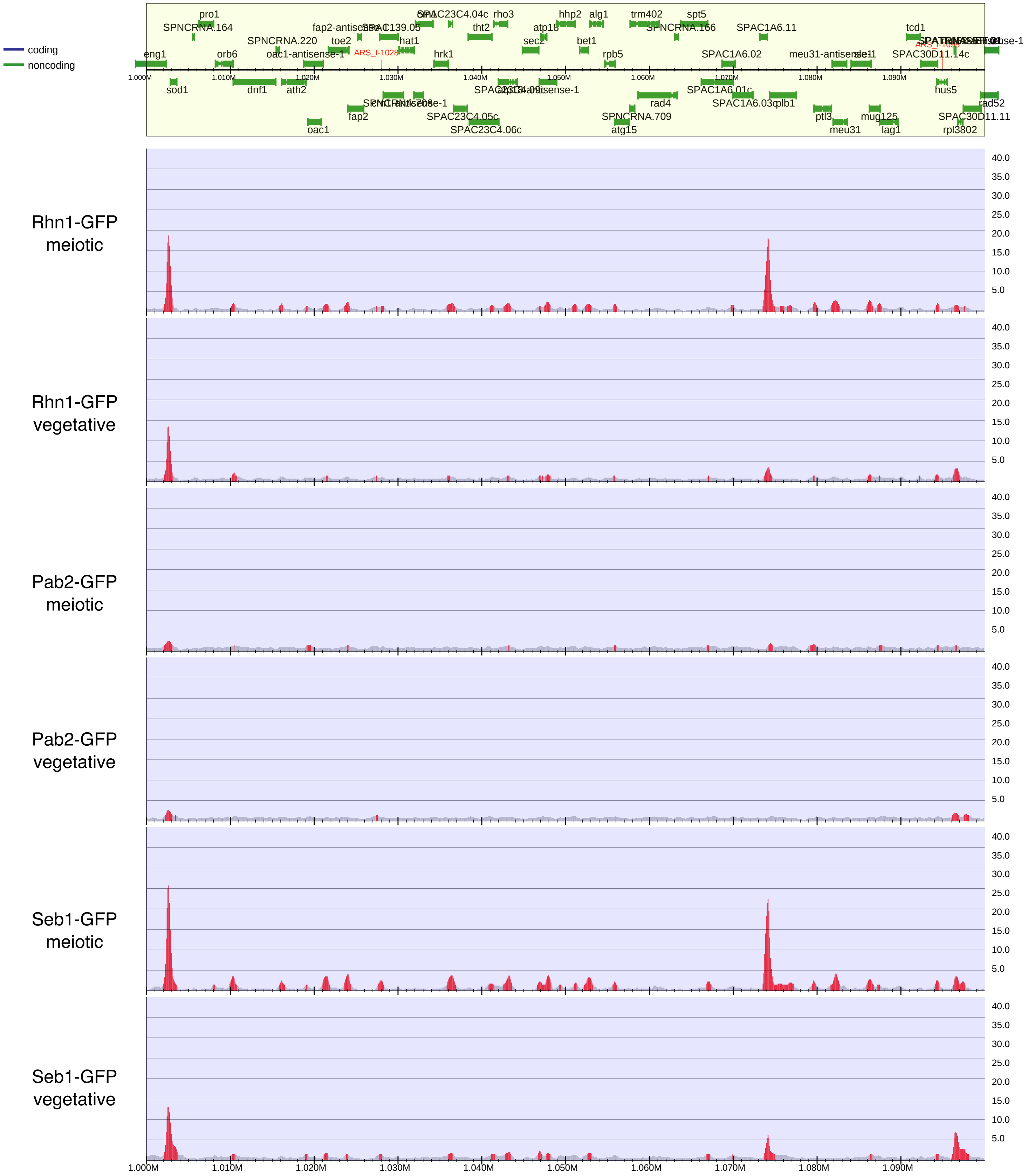
coding
noncoding
Rhn1-GFP meiotic
Rhn1-GFP vegetative
Pab2-GFP meiotic
Pab2-GFP vegetative
Seb1-GFP meiotic
Seb1-GFP vegetative
1.000M
1.010M
1.020M
1.030M
1.040M
1.050M
1.060M
1.070M
1.080M
1.090M
40.0
35.0
30.0
25.0
20.0
15.0
10.0
5.0

I_1_12

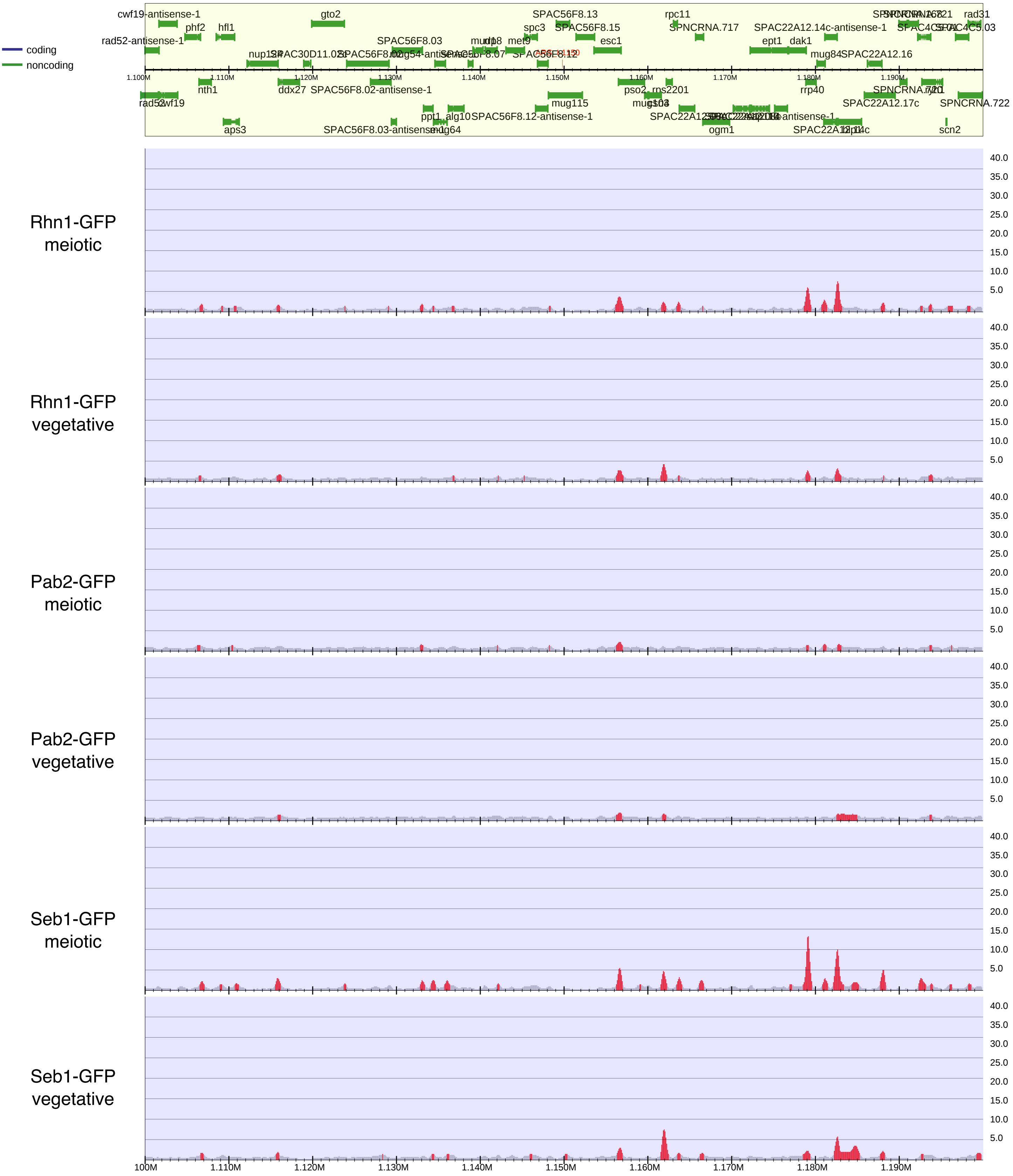
coding
noncoding
cwf19-antisense-1
gto2
SPAC56F8.13
rpc11
rad31
phf2
hfl1
spc3
SPAC56F8.15
SPNCRNA.717
SPAC22A12.14c-antisense-1
rad52-antisense-1
SPAC56F8.03
esc1
ept1
dak1
mug84
SPAC22A12.16
nth1
ddx27
SPAC56F8.02-antisense-1
mug115
pso2
rps2201
rrp40
SPAC22A12.17c
SPNCRNA.722
ppt1
alg10
SPAC56F8.12-antisense-1
aps3
ogm1
scn2
Rhn1-GFP
meiotic
Rhn1-GFP
vegetative
Pab2-GFP
meiotic
Pab2-GFP
vegetative
Seb1-GFP
meiotic
Seb1-GFP
vegetative
40.0
35.0
30.0
25.0
20.0
15.0
10.0
5.0
100M
1.110M
1.120M
1.130M
1.140M
1.150M
1.160M
1.170M
1.180M
1.190M

I_1_13

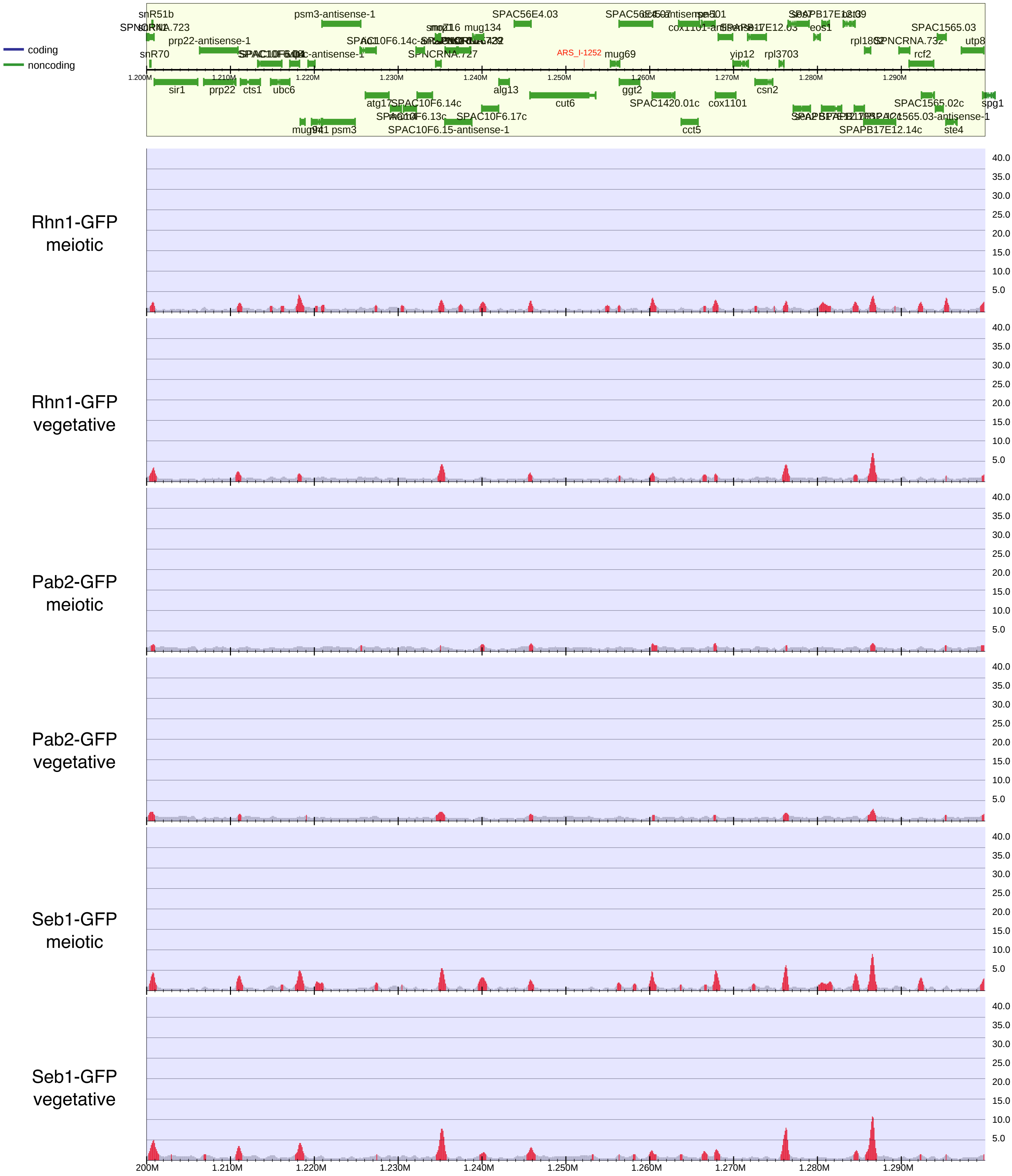

coding
noncoding
snR51b
psm3-antisense-1
SPAC56E4.03
prp22-antisense-1
SPAC1565.03
snR70
SPNCRNA.727
ARS_I-1252
mug69
yip12
rpl3703
rcf2
utp8
sir1
prp22
cts1
ubc6
alg13
cut6
ggt2
csn2
atg17
SPAC10F6.14c
SPAC1420.01c
cox1101
SPAC1565.02c
spg1
SPAC10F6.13c
SPAC10F6.17c
SPAC10F6.15-antisense-1
cct5
SPAPB17E12.14c
ste4
mug134
eos1
1.200M
1.210M
1.220M
1.230M
1.240M
1.250M
1.260M
1.270M
1.280M
1.290M
Rhn1-GFP meiotic
Rhn1-GFP vegetative
Pab2-GFP meiotic
Pab2-GFP vegetative
Seb1-GFP meiotic
Seb1-GFP vegetative
40.0
35.0
30.0
25.0
20.0
15.0
10.0
5.0
200M
1.210M
1.220M
1.230M
1.240M
1.250M
1.260M
1.270M
1.280M
1.290M

I_1_14

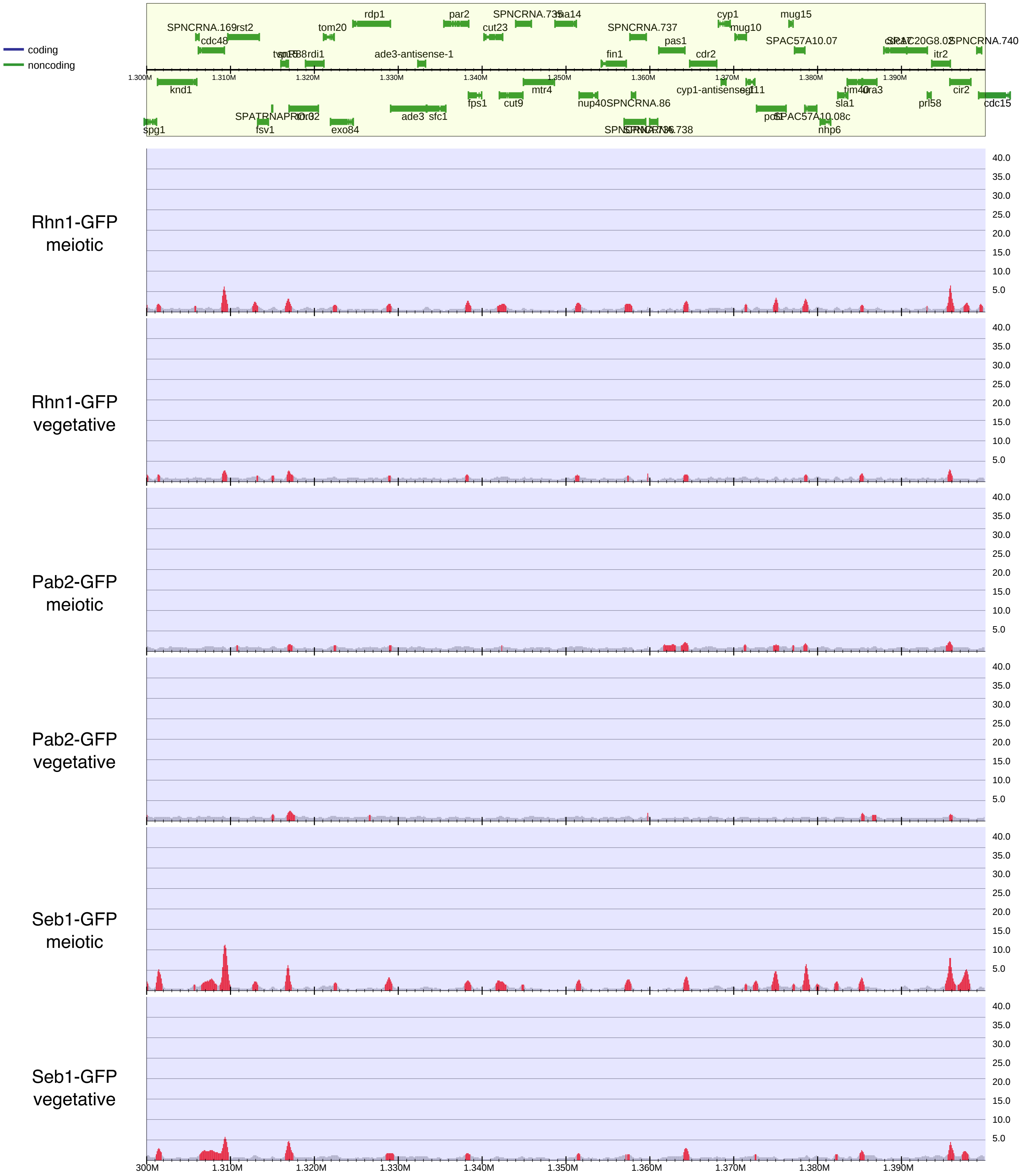
coding
noncoding
rdp1
par2
SPNCRNA.735
sla14
cyp1
mug15
SPNCRNA.169
rst2
tom20
cut23
SPNCRNA.737
mug10
cdc48
pas1
SPAC57A10.07
SPAC20G8.02
SPNCRNA.740
ade3-antisense-1
fin1
cdr2
itr2
1.300M
1.310M
1.320M
1.330M
1.340M
1.350M
1.360M
1.370M
1.380M
1.390M
knd1
mtr4
cyp1-antisense-1
tim40
cir2
fps1
cut9
nup40
SPNCRNA.86
sla1
prl58
cdc15
ade3
sfc1
SPAC57A10.08c
spg1
fsv1
exo84
nhp6
Rhn1-GFP meiotic
Rhn1-GFP vegetative
Pab2-GFP meiotic
Pab2-GFP vegetative
Seb1-GFP meiotic
Seb1-GFP vegetative
40.0
35.0
30.0
25.0
20.0
15.0
10.0
5.0
300M
1.310M
1.320M
1.330M
1.340M
1.350M
1.360M
1.370M
1.380M
1.390M

I_1_15

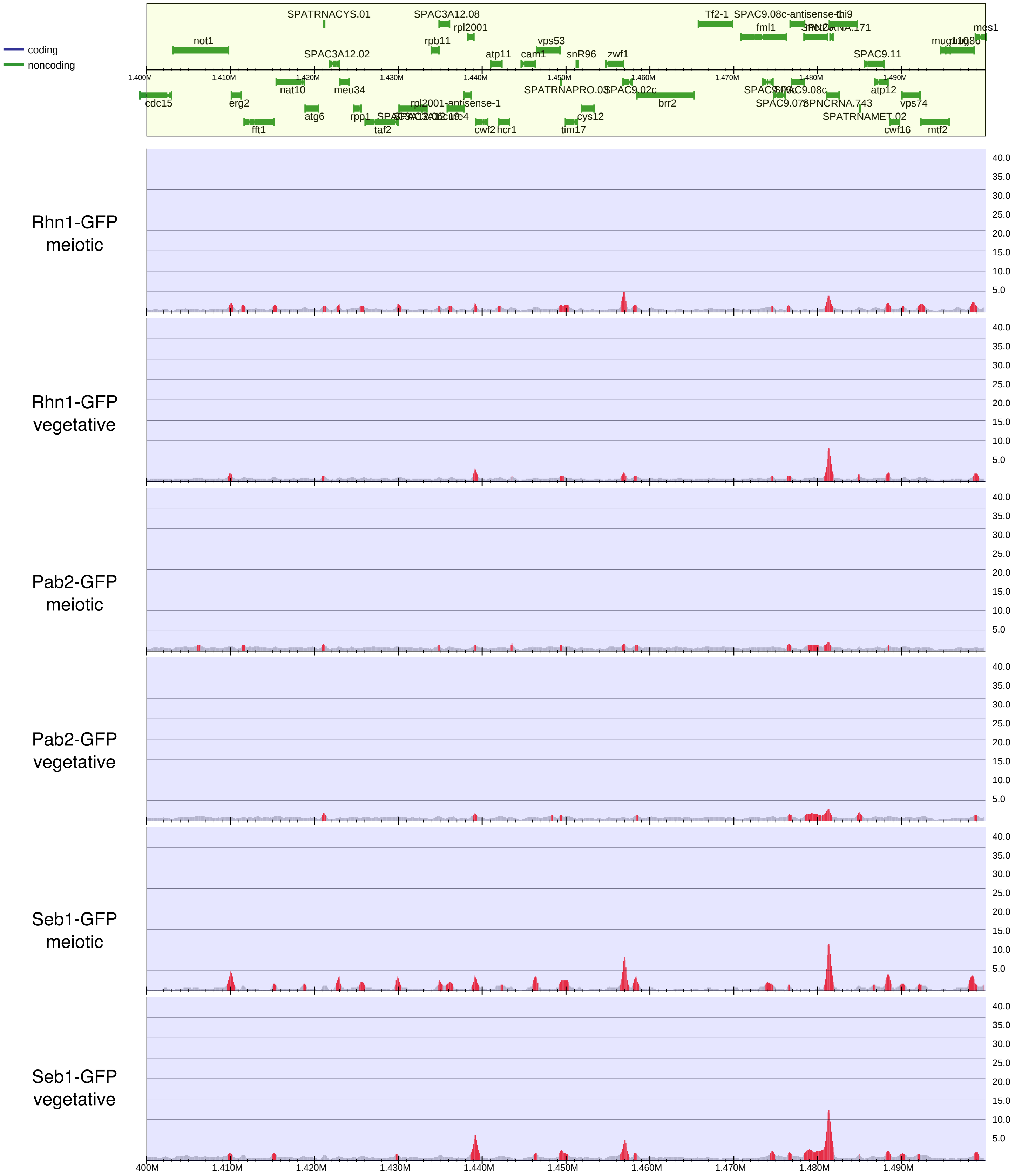

coding
noncoding
SPATRNACYS.01
SPAC3A12.08
rpl2001
Tf2-1
SPAC9.08c-antisense-1
thi9
fml1
mes1
not1
rpb11
vps53
SPAC3A12.02
atp11
cam1
snR96
zwf1
SPAC9.11
cdc15
erg2
nat10
meu34
atg6
rpp1
rpl2001-antisense-1
SPATRNAPRO.03
SPAC9.02c
brr2
SPAC9.08c
atp12
SPAC9.07c
SPNCRNA.743
vps74
SPATRNAMET.02
cys12
fft1
taf2
cwf2
hcr1
tim17
cwf16
mtf2
Rhn1-GFP meiotic
Rhn1-GFP vegetative
Pab2-GFP meiotic
Pab2-GFP vegetative
Seb1-GFP meiotic
Seb1-GFP vegetative
40.0
35.0
30.0
25.0
20.0
15.0
10.0
5.0
400M
1.410M
1.420M
1.430M
1.440M
1.450M
1.460M
1.470M
1.480M
1.490M

I_1_16

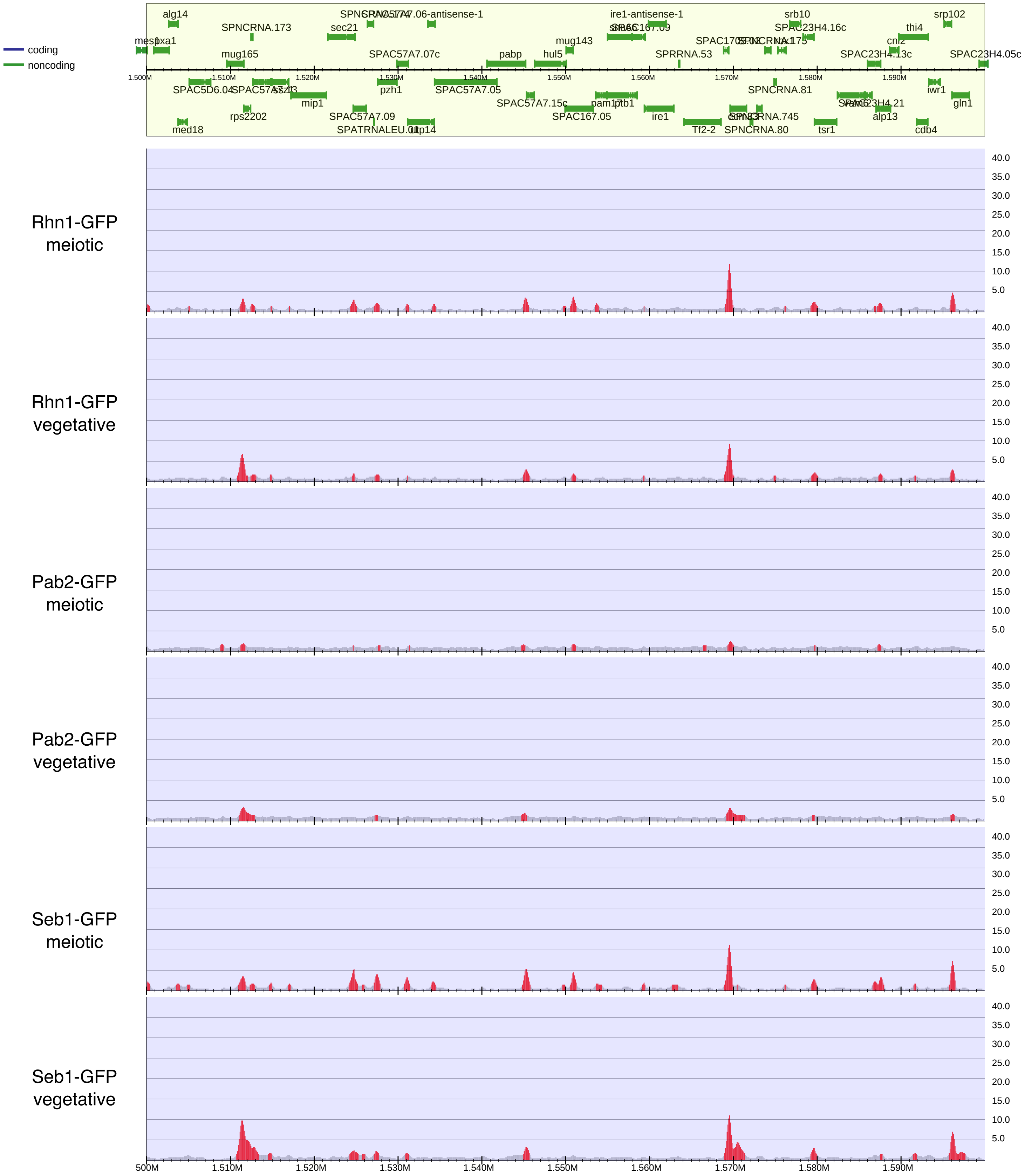

coding
noncoding
Rhn1-GFP meiotic
Rhn1-GFP vegetative
Pab2-GFP meiotic
Pab2-GFP vegetative
Seb1-GFP meiotic
Seb1-GFP vegetative
40.0
35.0
30.0
25.0
20.0
15.0
10.0
5.0
500M
1.510M
1.520M
1.530M
1.540M
1.550M
1.560M
1.570M
1.580M
1.590M

I_1_17

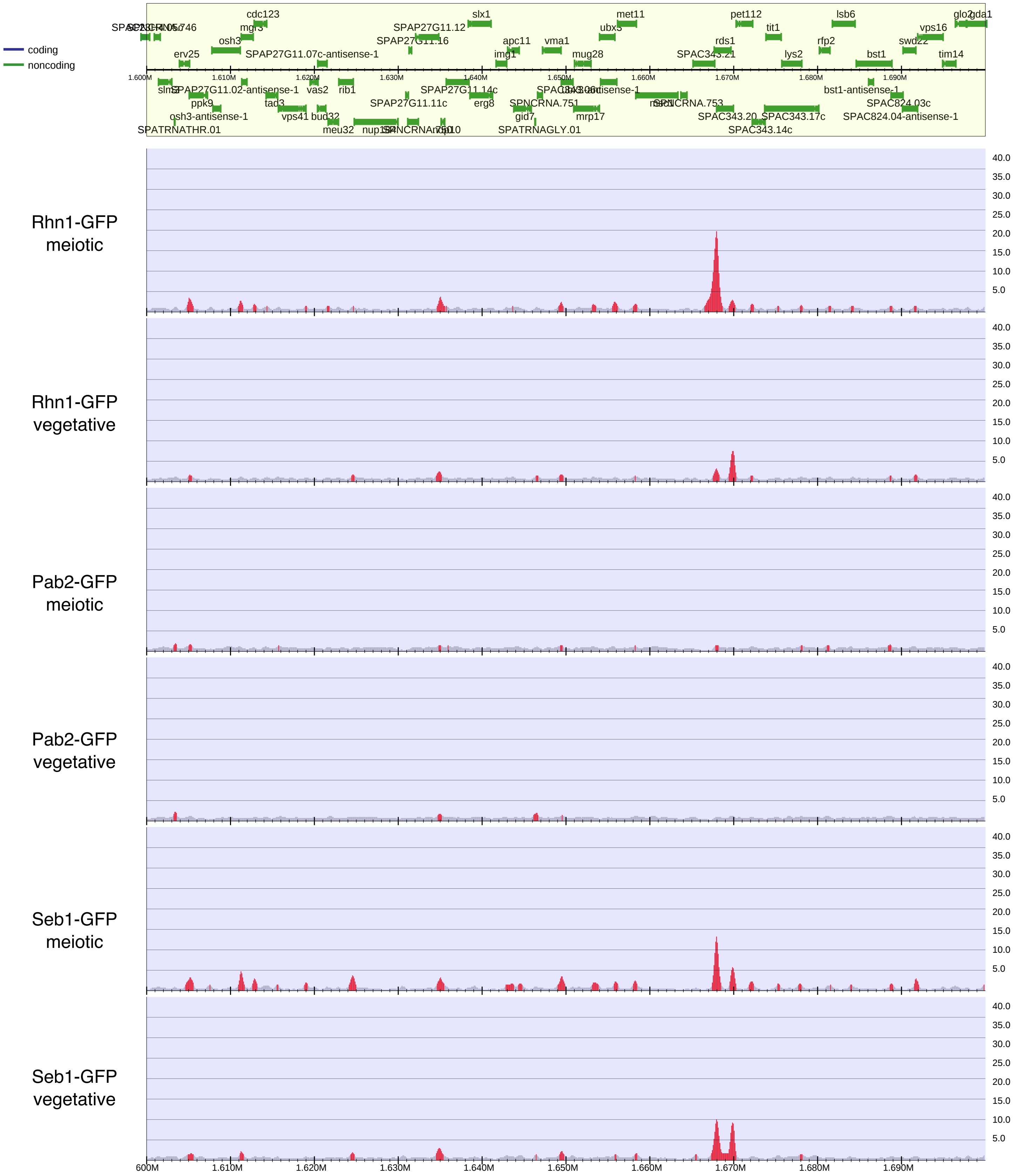
coding
noncoding
cdc123
slx1
met11
pet112
lsb6
glo2
gda1
mfr3
SPAP27G11.12
ubx3
tit1
vps16
osh3
SPAP27G11.16
apc11
vma1
rds1
rfp2
swd22
erv25
SPAP27G11.07c-antisense-1
img1
mug28
SPAC343.21
lys2
bst1
tim14
1.600M
1.610M
1.620M
1.630M
1.640M
1.650M
1.660M
1.670M
1.680M
1.690M
vas2
rib1
SPAP27G11.14c
bst1-antisense-1
ppk9
tad3
SPAP27G11.11c
erg8
SPNCRNA.751
SPNCRNA.753
SPAC824.03c
osh3-antisense-1
vps41
bud32
gid7
mrp17
SPAC343.20
SPAC343.17c
SPAC824.04-antisense-1
SPATRNATHR.01
meu32
SPATRNAGLY.01
SPAC343.14c
Rhn1-GFP meiotic
Rhn1-GFP vegetative
Pab2-GFP meiotic
Pab2-GFP vegetative
Seb1-GFP meiotic
Seb1-GFP vegetative
40.0
35.0
30.0
25.0
20.0
15.0
10.0
5.0
600M
1.610M
1.620M
1.630M
1.640M
1.650M
1.660M
1.670M
1.680M
1.690M

I_1_18

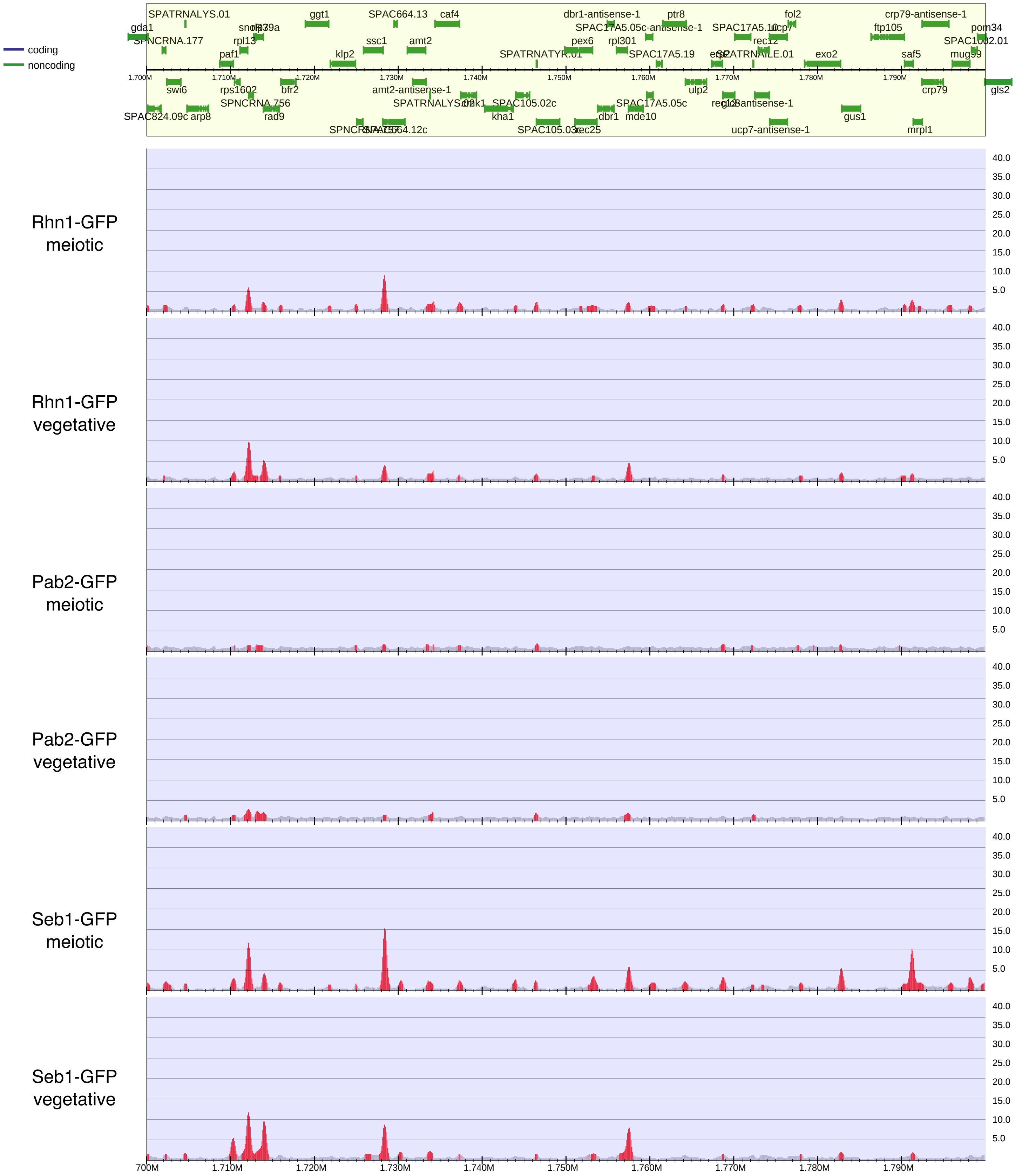
coding
noncoding
Rhn1-GFP meiotic
Rhn1-GFP vegetative
Pab2-GFP meiotic
Pab2-GFP vegetative
Seb1-GFP meiotic
Seb1-GFP vegetative
1.710M
1.720M
1.730M
1.740M
1.750M
1.760M
1.770M
1.780M
1.790M
40.0
35.0
30.0
25.0
20.0
15.0
10.0
5.0

I_1_19

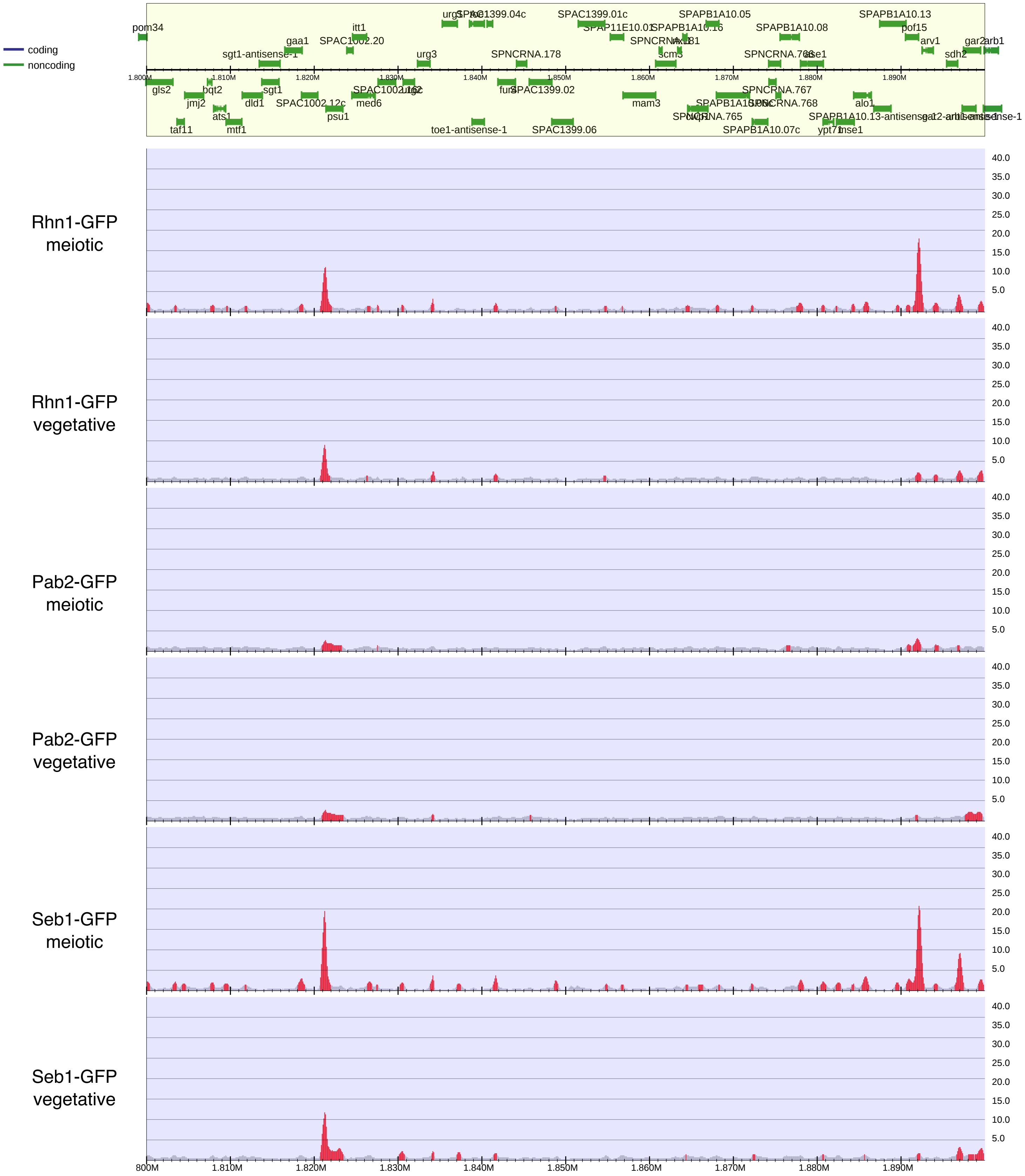
coding
noncoding
Rhn1-GFP meiotic
Rhn1-GFP vegetative
Pab2-GFP meiotic
Pab2-GFP vegetative
Seb1-GFP meiotic
Seb1-GFP vegetative
40.0
35.0
30.0
25.0
20.0
15.0
10.0
5.0
800M
1.810M
1.820M
1.830M
1.840M
1.850M
1.860M
1.870M
1.880M
1.890M

I_1_20

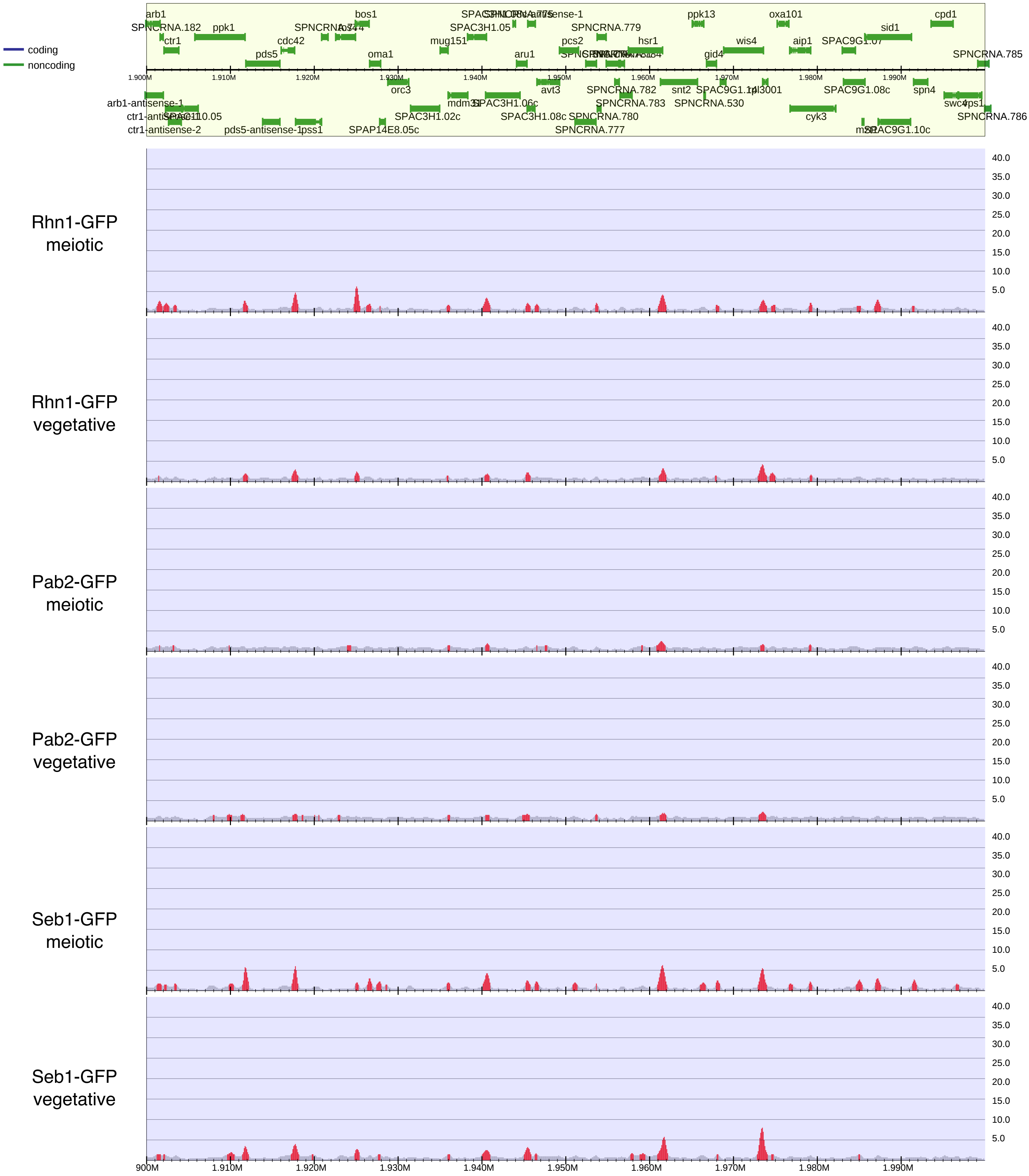

coding
noncoding
Rhn1-GFP meiotic
Rhn1-GFP vegetative
Pab2-GFP meiotic
Pab2-GFP vegetative
Seb1-GFP meiotic
Seb1-GFP vegetative
40.0
35.0
30.0
25.0
20.0
15.0
10.0
5.0
900M
1.910M
1.920M
1.930M
1.940M
1.950M
1.960M
1.970M
1.980M
1.990M

I_1_21

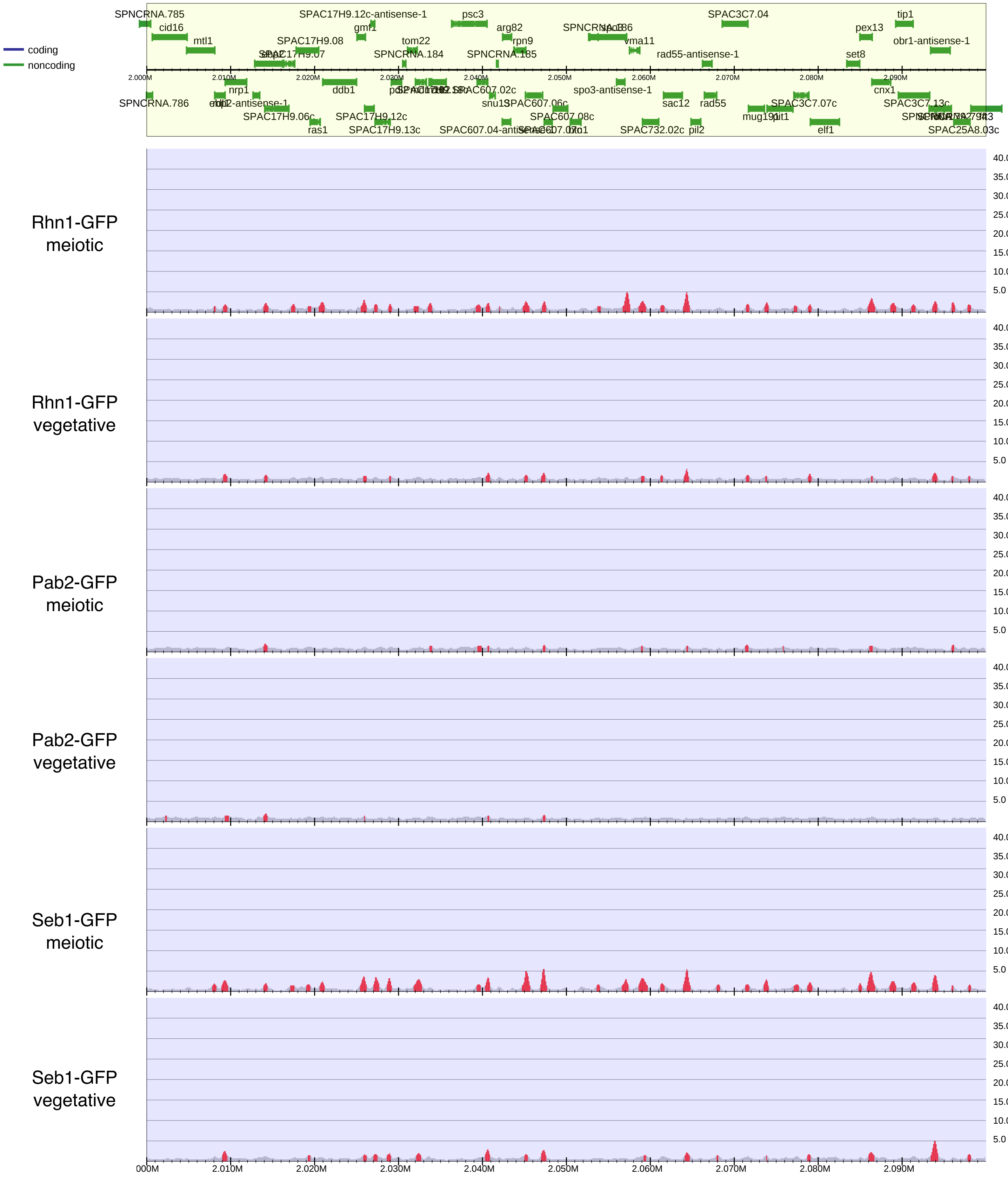
coding
noncoding
Rhn1-GFP meiotic
Rhn1-GFP vegetative
Pab2-GFP meiotic
Pab2-GFP vegetative
Seb1-GFP meiotic
Seb1-GFP vegetative
40.0
35.0
30.0
25.0
20.0
15.0
10.0
5.0
000M
2.010M
2.020M
2.030M
2.040M
2.050M
2.060M
2.070M
2.080M
2.090M

I_1_22

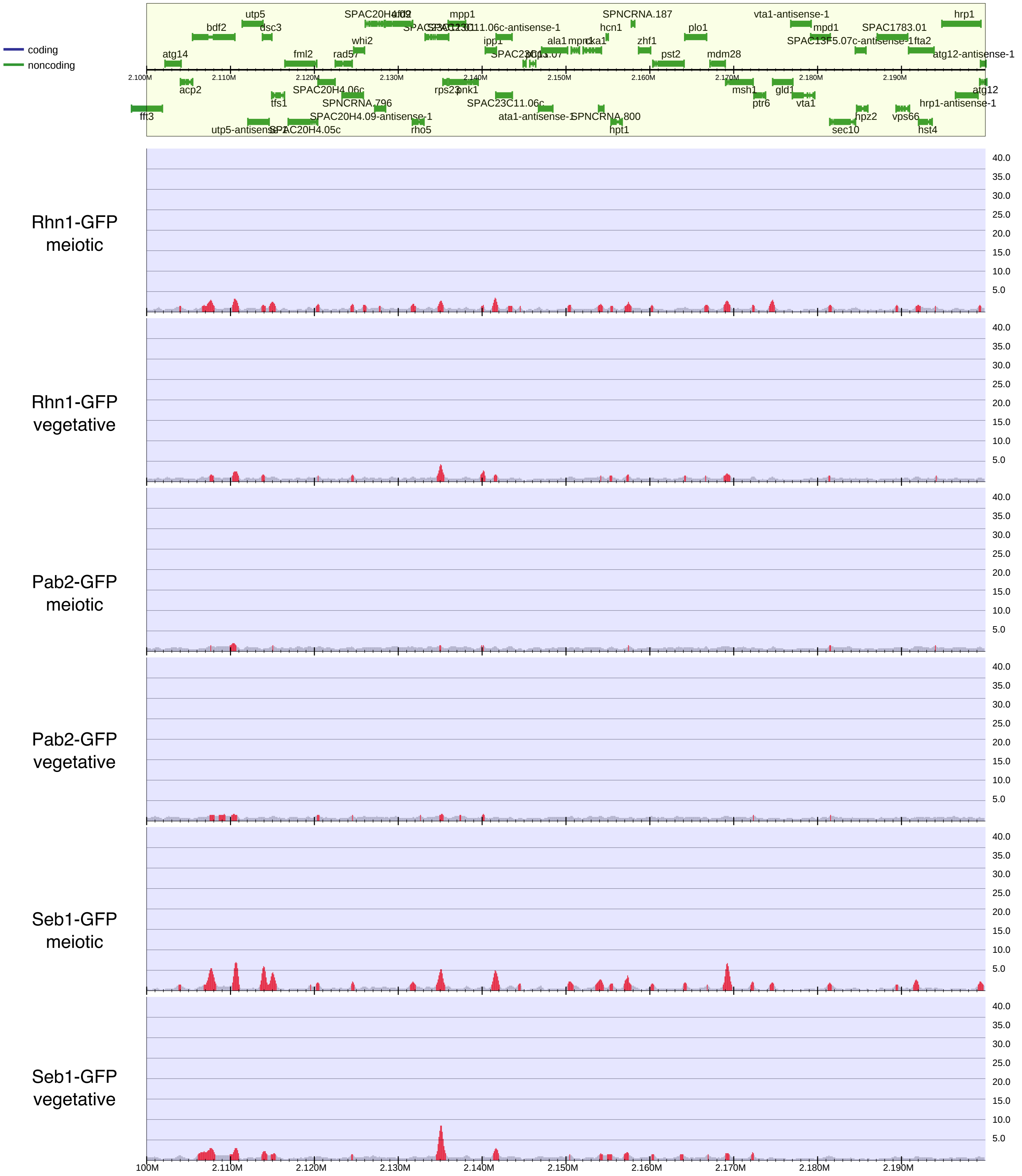

coding
noncoding
utp5
bdf2
dsc3
atg14
acp2
fft3
utp5-antisense-1
tfs1
fml2
rad57
whi2
SPAC20H4.06c
SPNCRNA.796
SPAC20H4.09-antisense-1
SPAC20H4.05c
mpp1
ipp1
rps23
pnk1
rho5
SPAC23C11.06c-antisense-1
SPAC23C11.06c
SPAC23C11.07
ala1
ata1-antisense-1
SPNCRNA.800
SPNCRNA.187
hcn1
zhf1
hpt1
plo1
pst2
mdm28
vta1-antisense-1
mpd1
SPAC1783.01
msh1
ptr6
gld1
vta1
hpz2
sec10
vps66
hst4
hrp1
fta2
atg12-antisense-1
atg12
hrp1-antisense-1
Rhn1-GFP meiotic
Rhn1-GFP vegetative
Pab2-GFP meiotic
Pab2-GFP vegetative
Seb1-GFP meiotic
Seb1-GFP vegetative
40.0
35.0
30.0
25.0
20.0
15.0
10.0
5.0
100M
2.110M
2.120M
2.130M
2.140M
2.150M
2.160M
2.170M
2.180M
2.190M

I_1_23

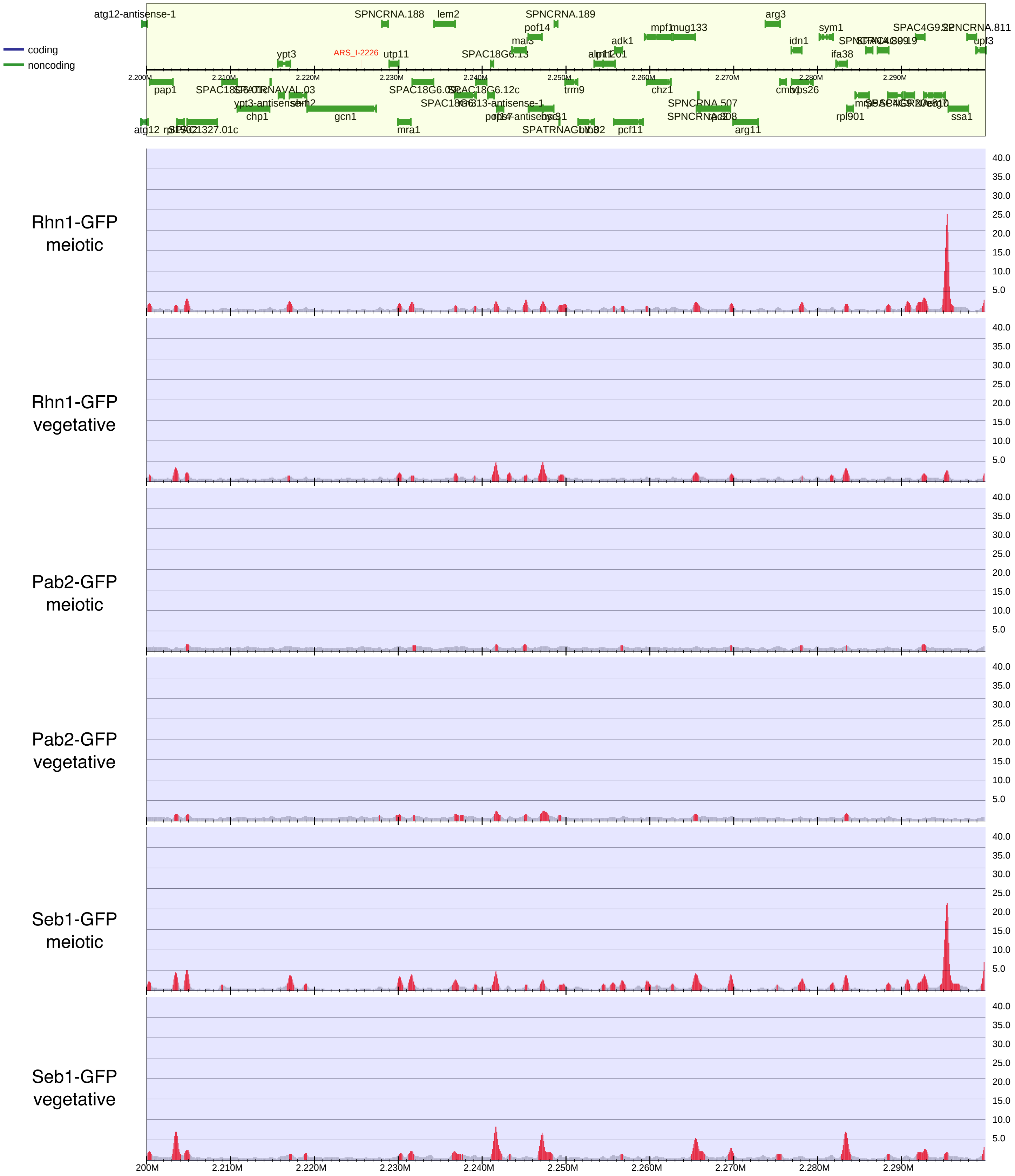
coding
noncoding
atg12-antisense-1
SPNCRNA.188
lem2
SPNCRNA.189
arg3
pof14
mpf1
mug133
sym1
upf3
mal3
adk1
idn1
ypt3
ARS_I-2226
utp11
SPAC18G6.13
ifa38
pap1
trm9
chz1
chp1
gcn1
SPAC18G6.12c
SPNCRNA.507
ssa1
atg12
mra1
pcf11
arg11
rpl901
Rhn1-GFP meiotic
Rhn1-GFP vegetative
Pab2-GFP meiotic
Pab2-GFP vegetative
Seb1-GFP meiotic
Seb1-GFP vegetative
40.0
35.0
30.0
25.0
20.0
15.0
10.0
5.0
200M
2.210M
2.220M
2.230M
2.240M
2.250M
2.260M
2.270M
2.280M
2.290M

I_1_24

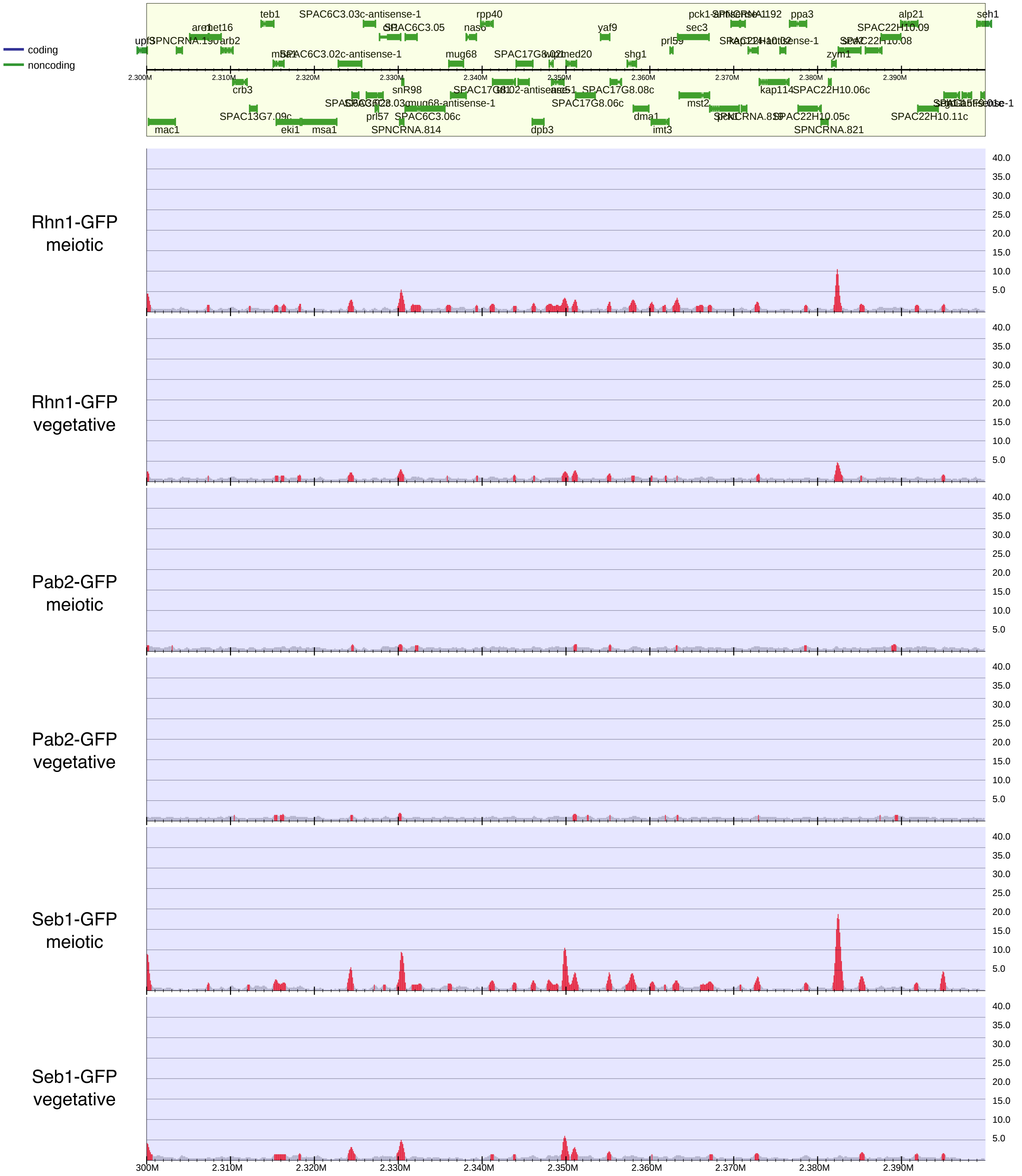
coding
noncoding
Rhn1-GFP meiotic
Rhn1-GFP vegetative
Pab2-GFP meiotic
Pab2-GFP vegetative
Seb1-GFP meiotic
Seb1-GFP vegetative
300M
2.310M
2.320M
2.330M
2.340M
2.350M
2.360M
2.370M
2.380M
2.390M

I_1_25

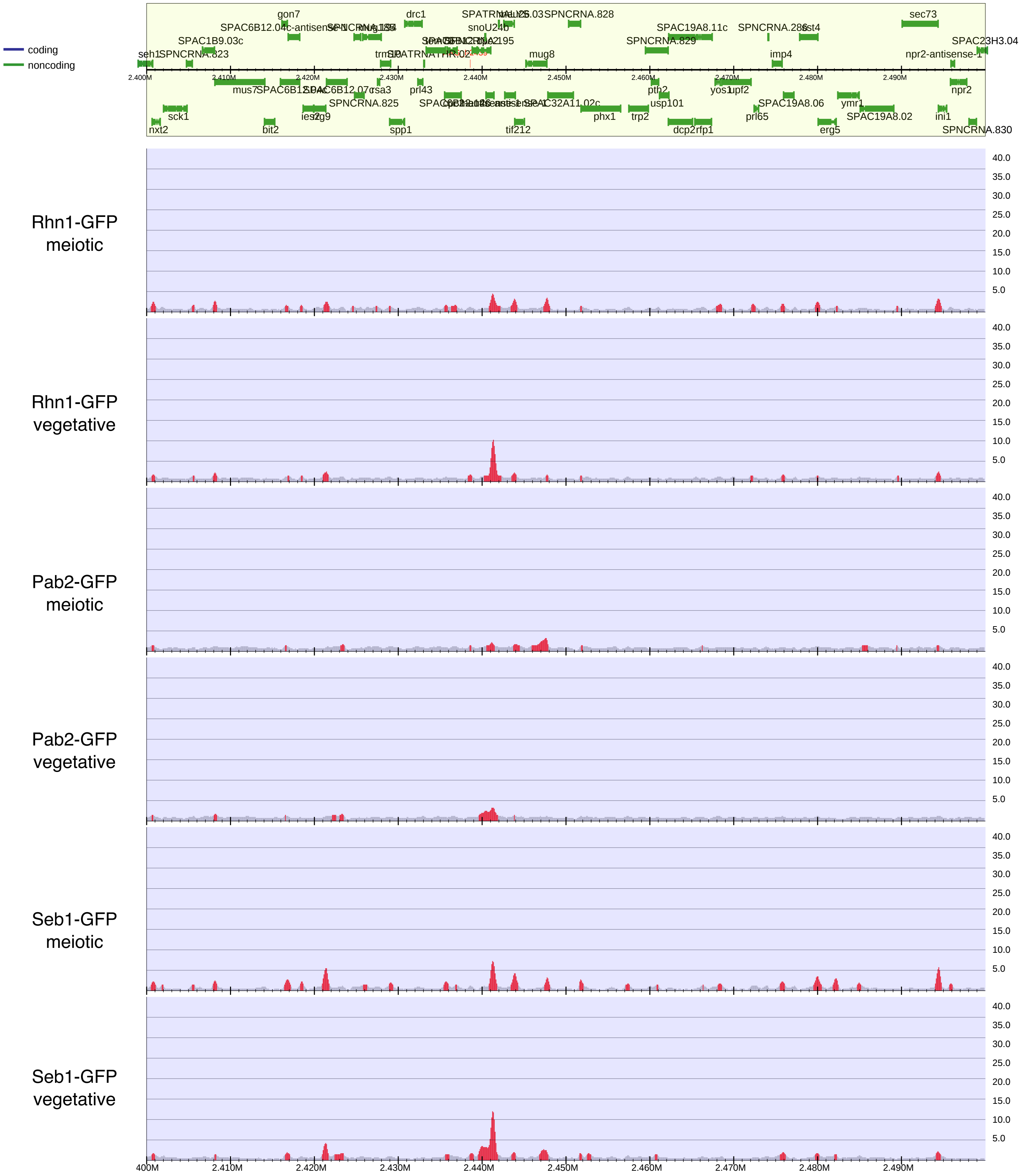

coding
noncoding
gon7
drc1
sec73
SPAC6B12.04c-antisense-1
SPNCRNA.828
SPAC19A8.11c
SPAC1B9.03c
SPNCRNA.829
SPAC23H3.04
seh1
SPNCRNA.823
mug8
imp4
npr2-antisense-1
mus7
prl43
pth2
npr2
SPNCRNA.825
usp101
SPAC19A8.06
ymr1
sck1
phx1
trp2
prl65
SPAC19A8.02
ini1
nxt2
bit2
spp1
tif212
erg5
SPNCRNA.830
Rhn1-GFP meiotic
Rhn1-GFP vegetative
Pab2-GFP meiotic
Pab2-GFP vegetative
Seb1-GFP meiotic
Seb1-GFP vegetative
40.0
35.0
30.0
25.0
20.0
15.0
10.0
5.0
400M
2.410M
2.420M
2.430M
2.440M
2.450M
2.460M
2.470M
2.480M
2.490M

I_1_26

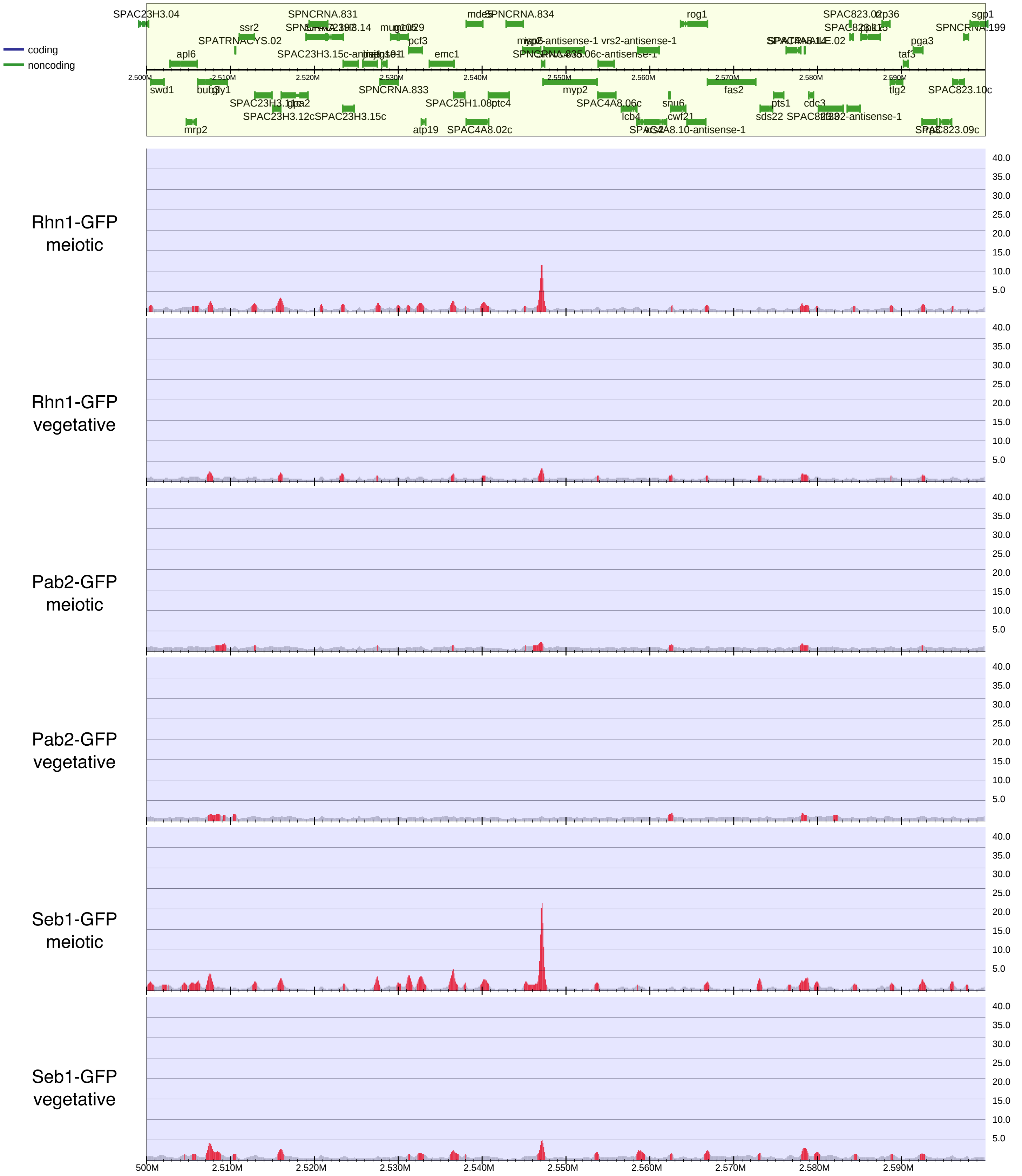

coding
noncoding
Rhn1-GFP meiotic
Rhn1-GFP vegetative
Pab2-GFP meiotic
Pab2-GFP vegetative
Seb1-GFP meiotic
Seb1-GFP vegetative
500M
2.510M
2.520M
2.530M
2.540M
2.550M
2.560M
2.570M
2.580M
2.590M

I_1_27

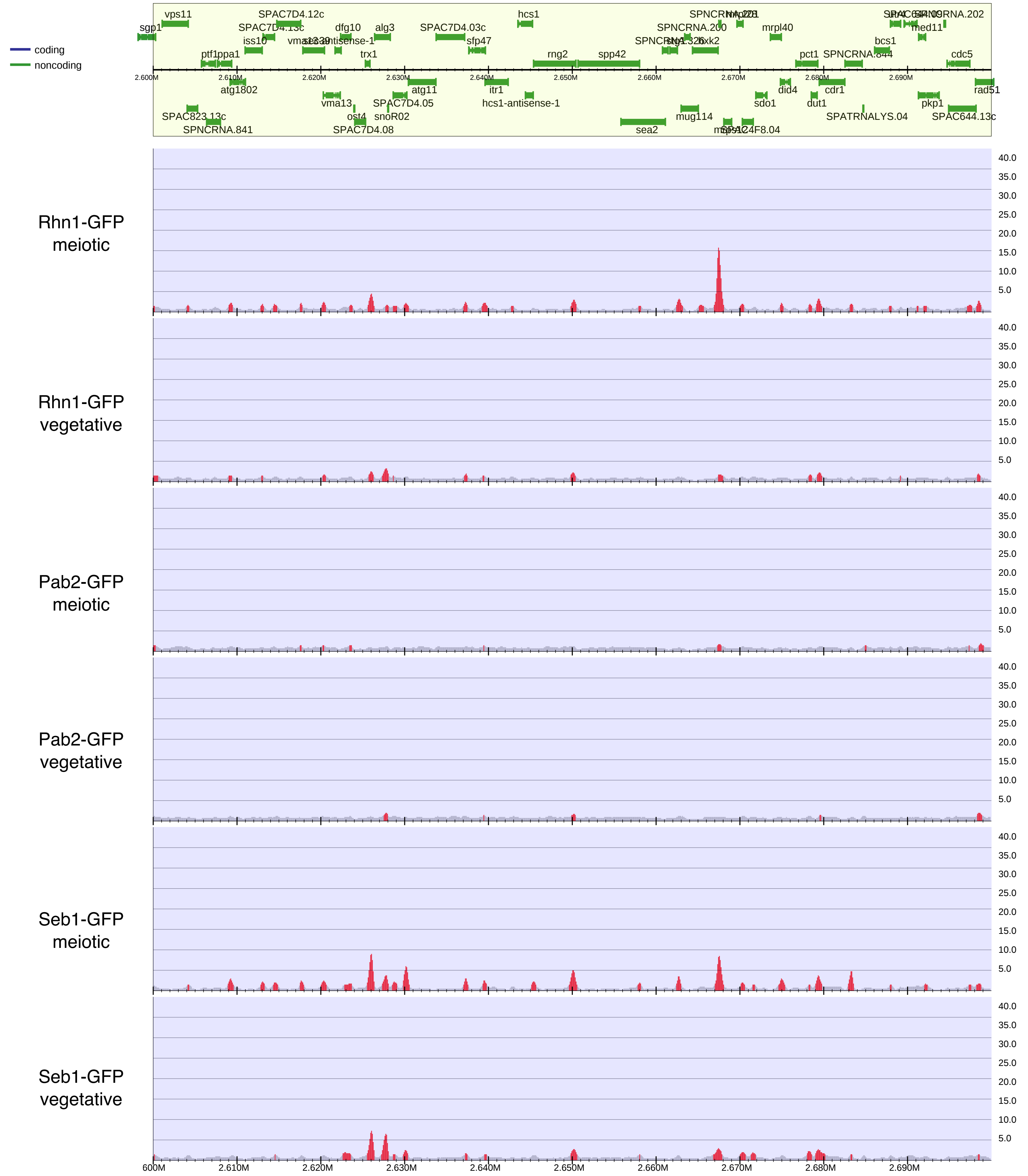
coding
noncoding
vps11
SPAC7D4.12c
hcs1
sgp1
dfg10
alg3
SPAC7D4.03c
SPNCRNA.200
mrpl40
iss10
sfp47
ptf1ppa1
trx1
rng2
spp42
pct1
bcs1
med11
cdc5
atg1802
atg11
itr1
did4
cdr1
rad51
vma13
SPAC7D4.05
hcs1-antisense-1
sdo1
dut1
pkp1
SPAC823.13c
ost4
snoR02
mug114
SPATRNALYS.04
SPAC644.13c
SPNCRNA.841
SPAC7D4.08
sea2
2.600M
2.610M
2.620M
2.630M
2.640M
2.650M
2.660M
2.670M
2.680M
2.690M
Rhn1-GFP meiotic
Rhn1-GFP vegetative
Pab2-GFP meiotic
Pab2-GFP vegetative
Seb1-GFP meiotic
Seb1-GFP vegetative
40.0
35.0
30.0
25.0
20.0
15.0
10.0
5.0
600M
2.610M
2.620M
2.630M
2.640M
2.650M
2.660M
2.670M
2.680M
2.690M

I_1_28

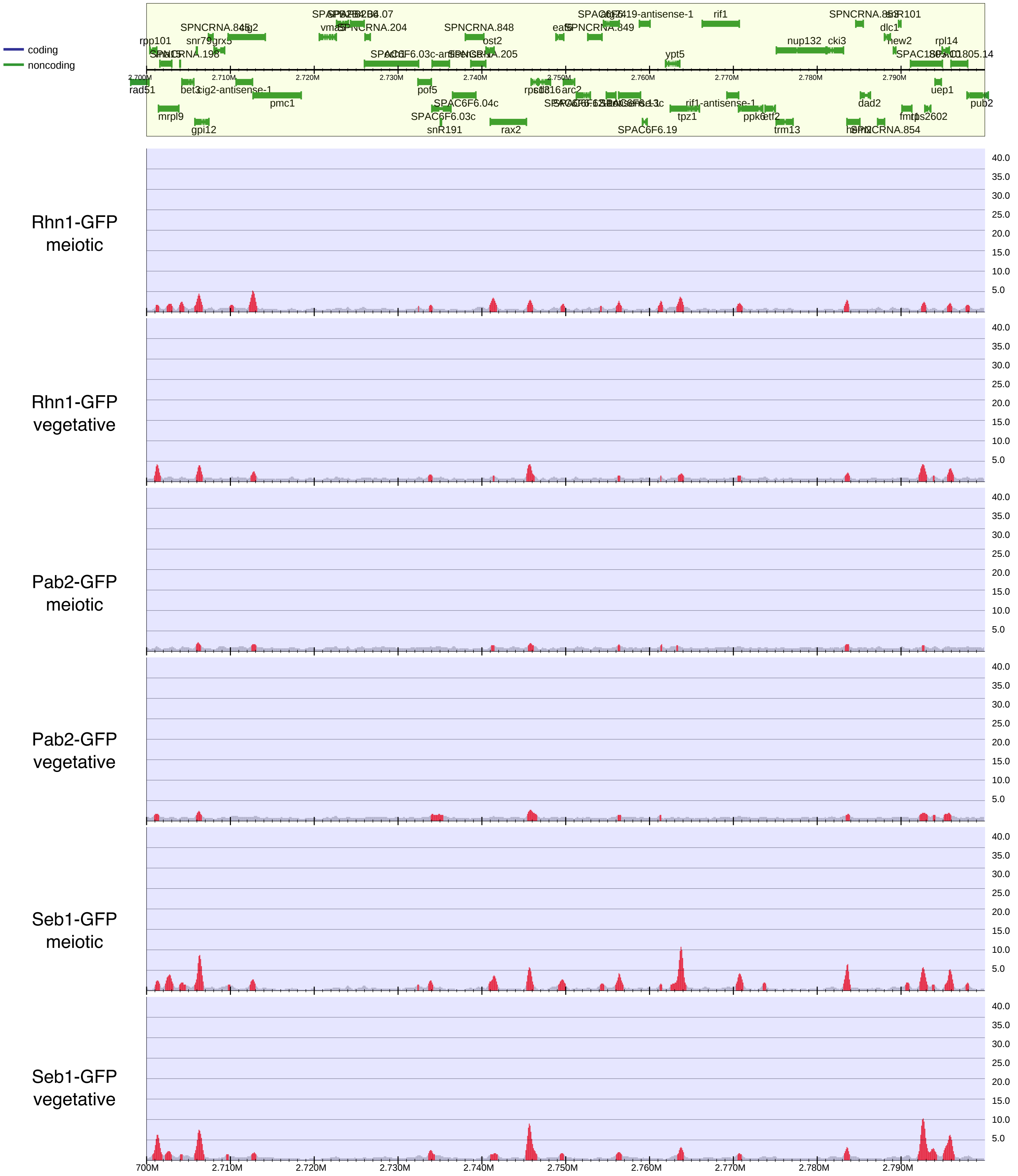
coding
noncoding
rad51
rpp101
mrpl9
gpi12
bet3
cig2-antisense-1
pmc1
pof5
SPAC6F6.04c
SPAC6F6.03c
snR191
rax2
ost2
SPNCRNA.848
SPNCRNA.204
rif1
rif1-antisense-1
tpz1
ypt5
SPAC6F6.19
nup132
cki3
dad2
dlc1
uep1
rpl14
pub2
trm13
2.700M
2.710M
2.720M
2.730M
2.740M
2.750M
2.760M
2.770M
2.780M
2.790M
Rhn1-GFP meiotic
Rhn1-GFP vegetative
Pab2-GFP meiotic
Pab2-GFP vegetative
Seb1-GFP meiotic
Seb1-GFP vegetative
40.0
35.0
30.0
25.0
20.0
15.0
10.0
5.0
700M

I_1_29

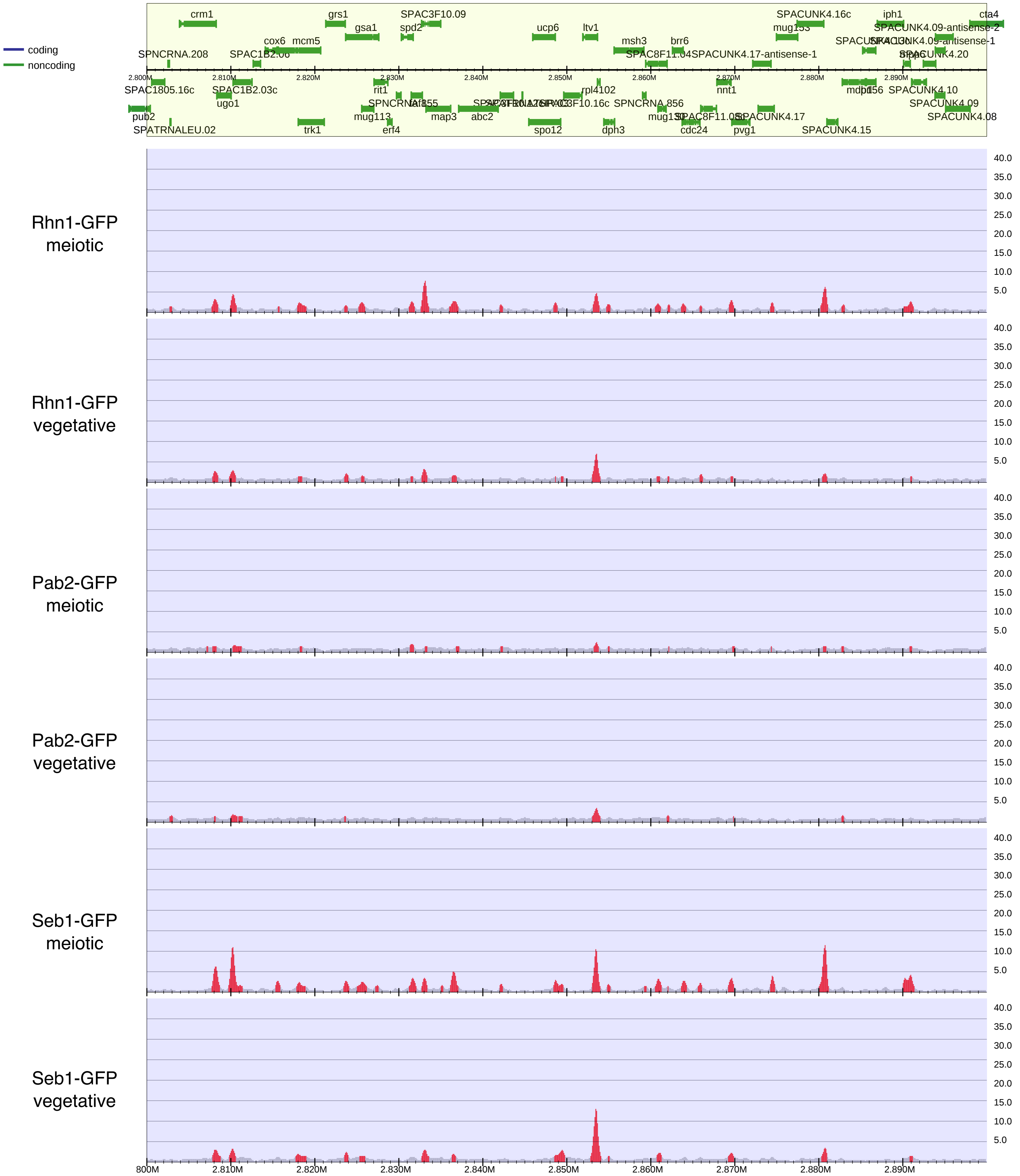
coding
noncoding
crm1
grs1
SPAC3F10.09
SPACUNK4.16c
iph1
cta4
gsa1
spd2
ucp6
ltv1
mug153
cox6
mcm5
msh3
brr6
SPNCRNA.208
SPAC8F11.04
SPACUNK4.17-antisense-1
SPACUNK4.20
SPAC1805.16c
SPAC1B2.03c
rit1
rpl4102
nnt1
SPACUNK4.10
ugo1
SPNCRNA.856
SPACUNK4.09
pub2
mug113
map3
abc2
SPACUNK4.17
SPACUNK4.08
SPATRNALEU.02
trk1
erf4
spo12
dph3
cdc24
pvg1
SPACUNK4.15
Rhn1-GFP meiotic
Rhn1-GFP vegetative
Pab2-GFP meiotic
Pab2-GFP vegetative
Seb1-GFP meiotic
Seb1-GFP vegetative
40.0
35.0
30.0
25.0
20.0
15.0
10.0
5.0
800M
2.810M
2.820M
2.830M
2.840M
2.850M
2.860M
2.870M
2.880M
2.890M

I_1_30

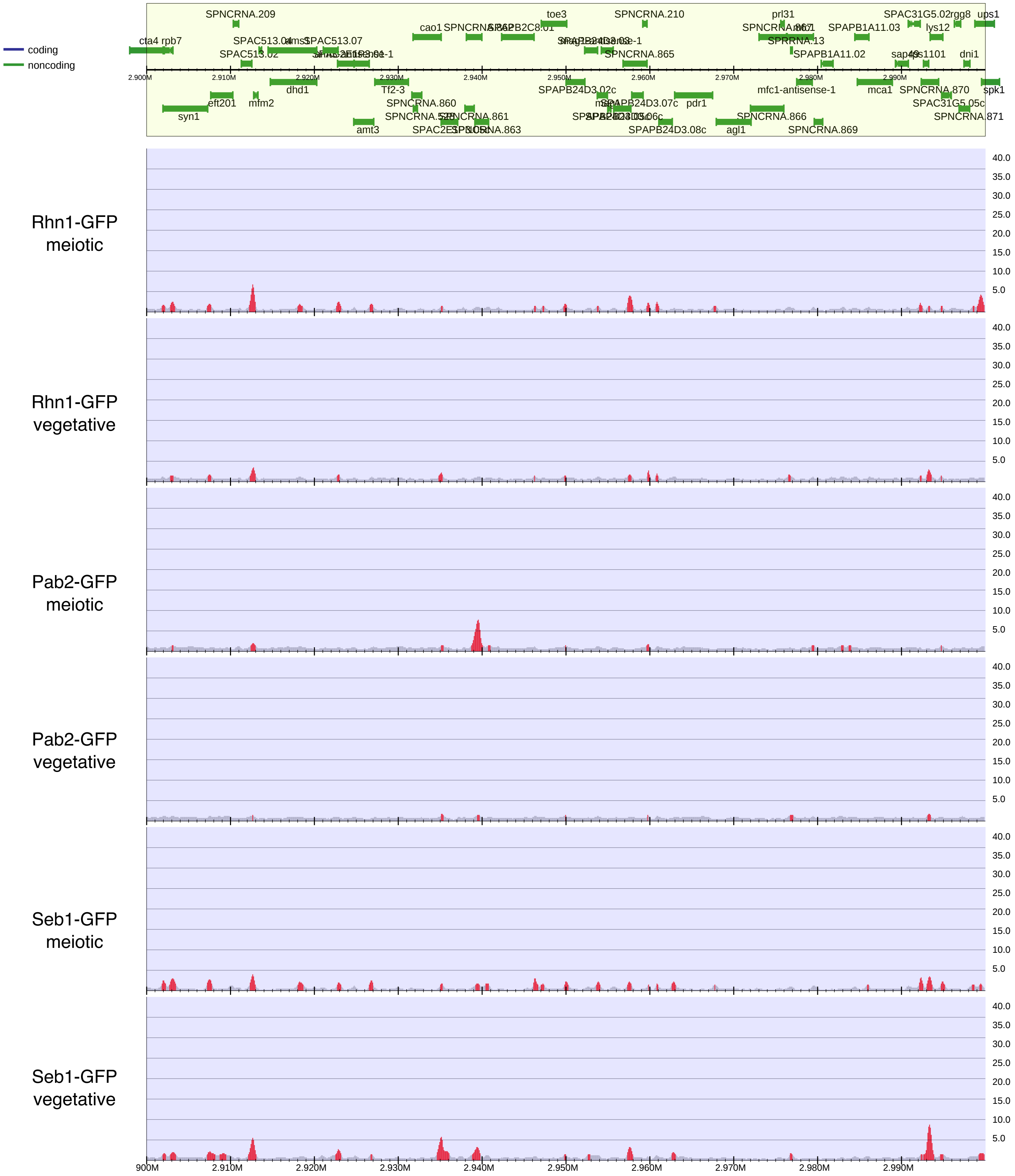
coding
noncoding
SPNCRNA.209
cta4 rpb7
SPAC513.02
toe3
SPNCRNA.210
prl31
ups1
cao1
SPNCRNA.865
SPRRNA.13
SPAPB1A11.03
SPAPB1A11.02
lys12
dni1
dhd1
Tf2-3
SPAPB24D3.02c
mfc1-antisense-1
mca1
SPNCRNA.870
spk1
eft201
mfm2
SPNCRNA.860
pdr1
SPAC31G5.05c
syn1
SPNCRNA.861
SPNCRNA.866
SPNCRNA.871
amt3
SPNCRNA.863
SPAPB24D3.08c
agl1
SPNCRNA.869
Rhn1-GFP
meiotic
Rhn1-GFP
vegetative
Pab2-GFP
meiotic
Pab2-GFP
vegetative
Seb1-GFP
meiotic
Seb1-GFP
vegetative
40.0
35.0
30.0
25.0
20.0
15.0
10.0
5.0
900M
2.910M
2.920M
2.930M
2.940M
2.950M
2.960M
2.970M
2.980M
2.990M

I_1_31

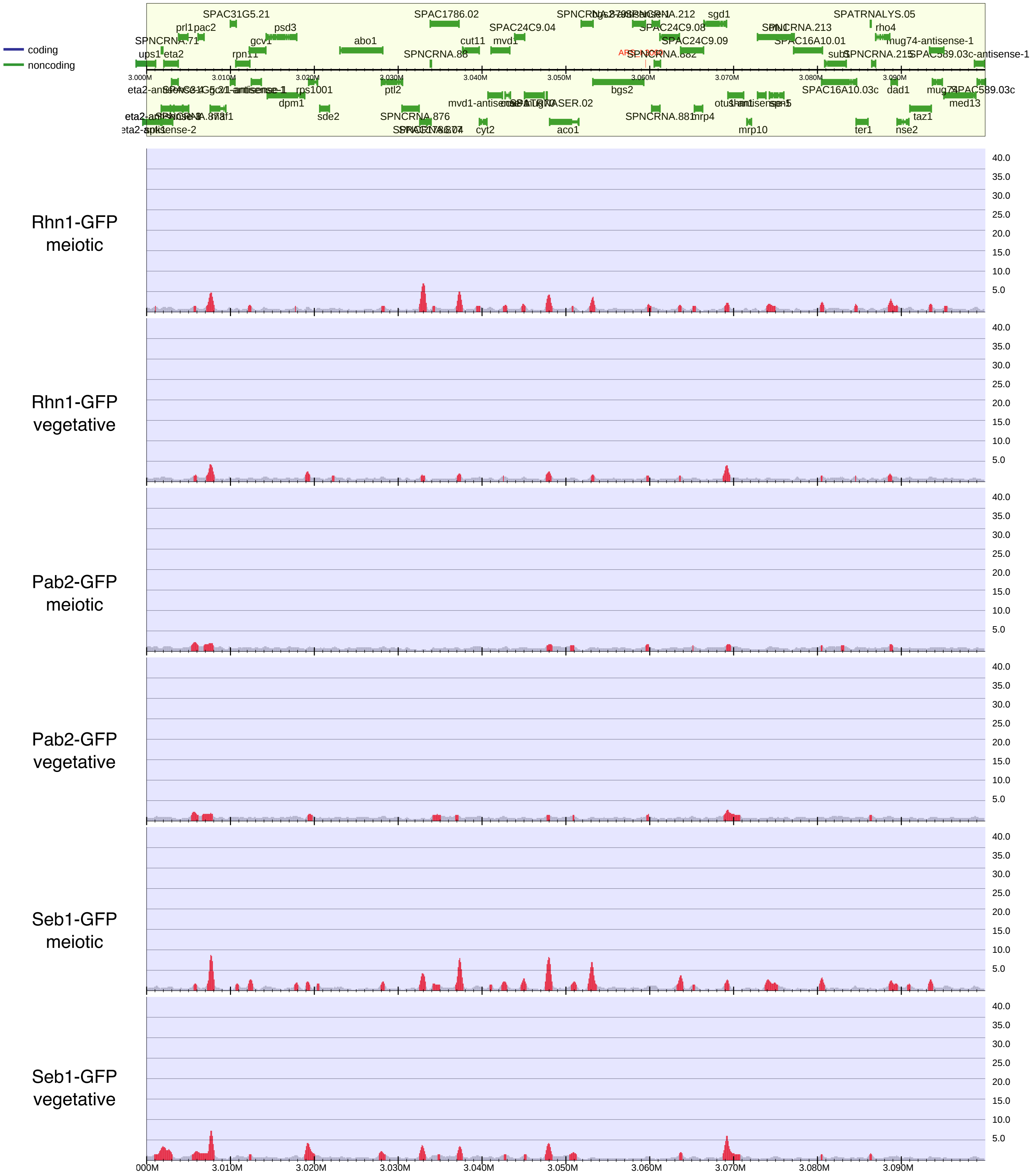
coding
noncoding
Rhn1-GFP meiotic
Rhn1-GFP vegetative
Pab2-GFP meiotic
Pab2-GFP vegetative
Seb1-GFP meiotic
Seb1-GFP vegetative
40.0
35.0
30.0
25.0
20.0
15.0
10.0
5.0
000M
3.010M
3.020M
3.030M
3.040M
3.050M
3.060M
3.070M
3.080M
3.090M

I_1_32

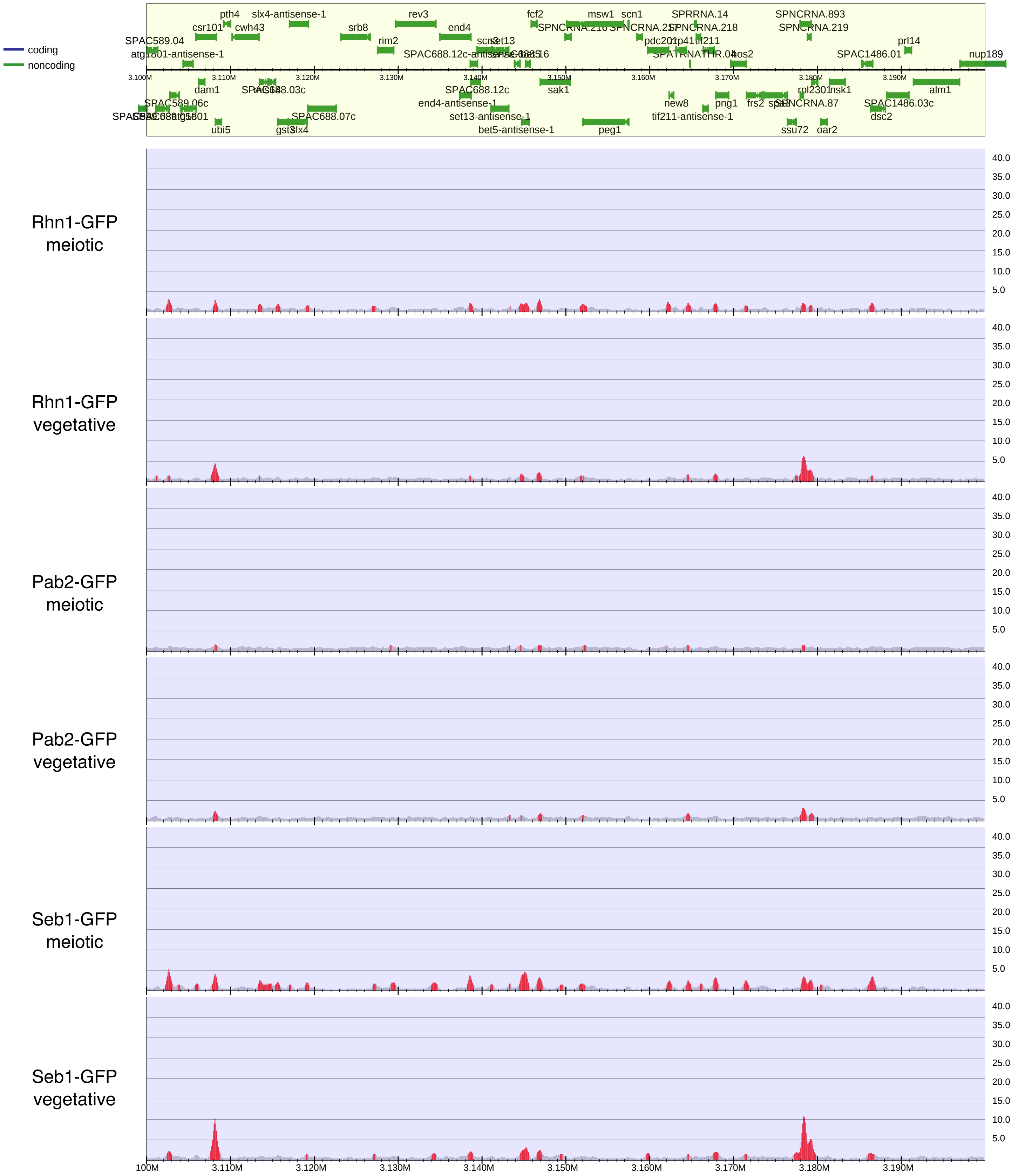
coding
noncoding
Rhn1-GFP meiotic
Rhn1-GFP vegetative
Pab2-GFP meiotic
Pab2-GFP vegetative
Seb1-GFP meiotic
Seb1-GFP vegetative
100M
3.110M
3.120M
3.130M
3.140M
3.150M
3.160M
3.170M
3.180M
3.190M

I_1_33

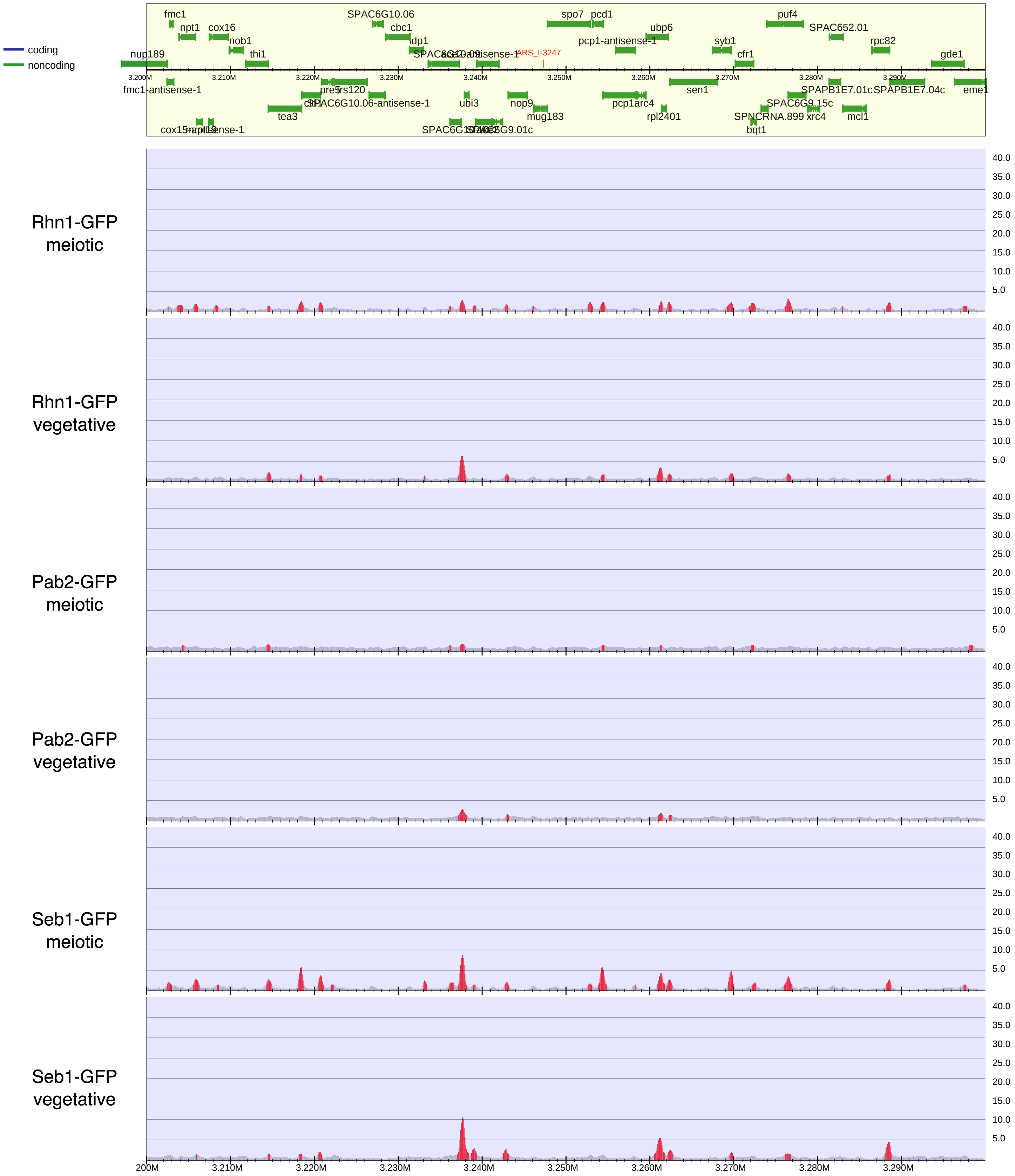
coding
noncoding
fmc1
npt1
cox16
nob1
nup189
thi1
SPAC6G10.06
cbc1
idp1
ARS_I-3247
spo7
pcd1
ubp6
pcp1-antisense-1
syb1
cfr1
puf4
SPAC652.01
rpc82
gde1
fmc1-antisense-1
tea3
ubi3
nop9
mug183
pcp1
arc4
rpl2401
sen1
SPAPB1E7.01c
SPAPB1E7.04c
eme1
SPAC6G9.15c
xrc4
mcl1
bqt1
3.200M
3.210M
3.220M
3.230M
3.240M
3.250M
3.260M
3.270M
3.280M
3.290M
Rhn1-GFP meiotic
Rhn1-GFP vegetative
Pab2-GFP meiotic
Pab2-GFP vegetative
Seb1-GFP meiotic
Seb1-GFP vegetative
40.0
35.0
30.0
25.0
20.0
15.0
10.0
5.0

I_1_34

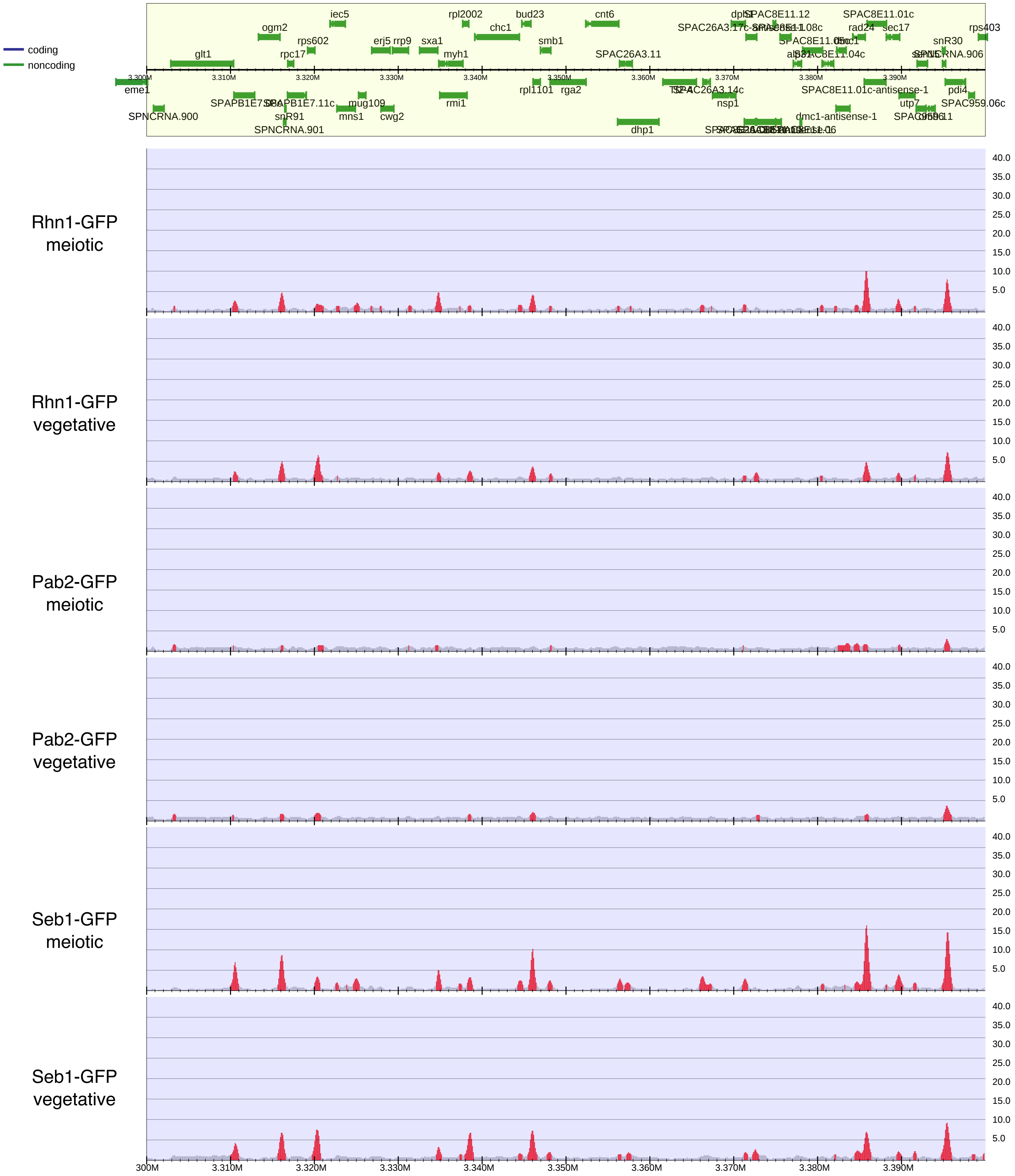

coding
noncoding
Rhn1-GFP meiotic
Rhn1-GFP vegetative
Pab2-GFP meiotic
Pab2-GFP vegetative
Seb1-GFP meiotic
Seb1-GFP vegetative
glt1
ogm2
iec5
rps602
rpc17
erj5 rrp9
sxa1
rpl2002
myh1
chc1
bud23
smb1
cnt6
SPAC26A3.11
eme1
SPAPB1E7.11c
mug109
snR91
SPNCRNA.900
SPNCRNA.901
mns1
cwg2
rmi1
rpl1101
rga2
dhp1
nsp1
SPAC26A3.14c
rad24
sec17
SPAC8E11.01c
SPAC8E11.04c
snR30
rps403
SPAC8E11.01c-antisense-1
utp7
pdi4
SPAC959.06c
dmc1-antisense-1
3.300M 3.310M 3.320M 3.330M 3.340M 3.350M 3.360M 3.370M 3.380M 3.390M
40.0 35.0 30.0 25.0 20.0 15.0 10.0 5.0
300M 3.310M 3.320M 3.330M 3.340M 3.350M 3.360M 3.370M 3.380M 3.390M

I_1_35

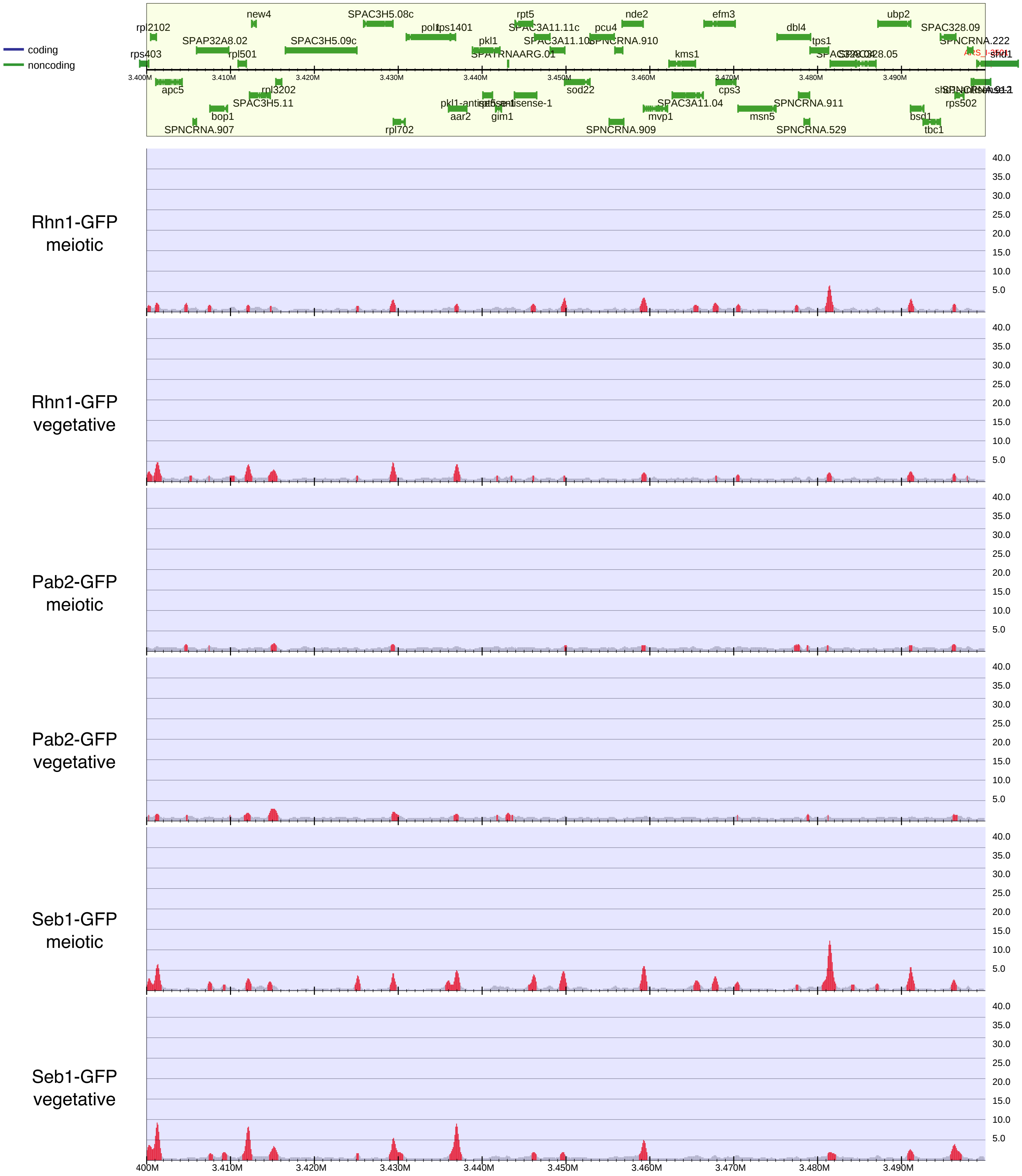
coding
noncoding
rpl2102
rps403
SPAP32A8.02
new4
rpl501
SPAC3H5.09c
SPAC3H5.08c
rpt5
nde2
efm3
ubp2
dbl4
SPAC328.09
pcu4
tps1
kms1
apc5
rpl3202
SPAC3H5.11
bop1
SPNCRNA.907
rpl702
aar2
gim1
sod22
cps3
SPAC3A11.04
mvp1
msn5
SPNCRNA.911
SPNCRNA.909
SPNCRNA.529
rps502
bsd1
tbc1
3.400M
3.410M
3.420M
3.430M
3.440M
3.450M
3.460M
3.470M
3.480M
3.490M
Rhn1-GFP meiotic
Rhn1-GFP vegetative
Pab2-GFP meiotic
Pab2-GFP vegetative
Seb1-GFP meiotic
Seb1-GFP vegetative
40.0
35.0
30.0
25.0
20.0
15.0
10.0
5.0
400M
3.410M
3.420M
3.430M
3.440M
3.450M
3.460M
3.470M
3.480M
3.490M

I_1_36

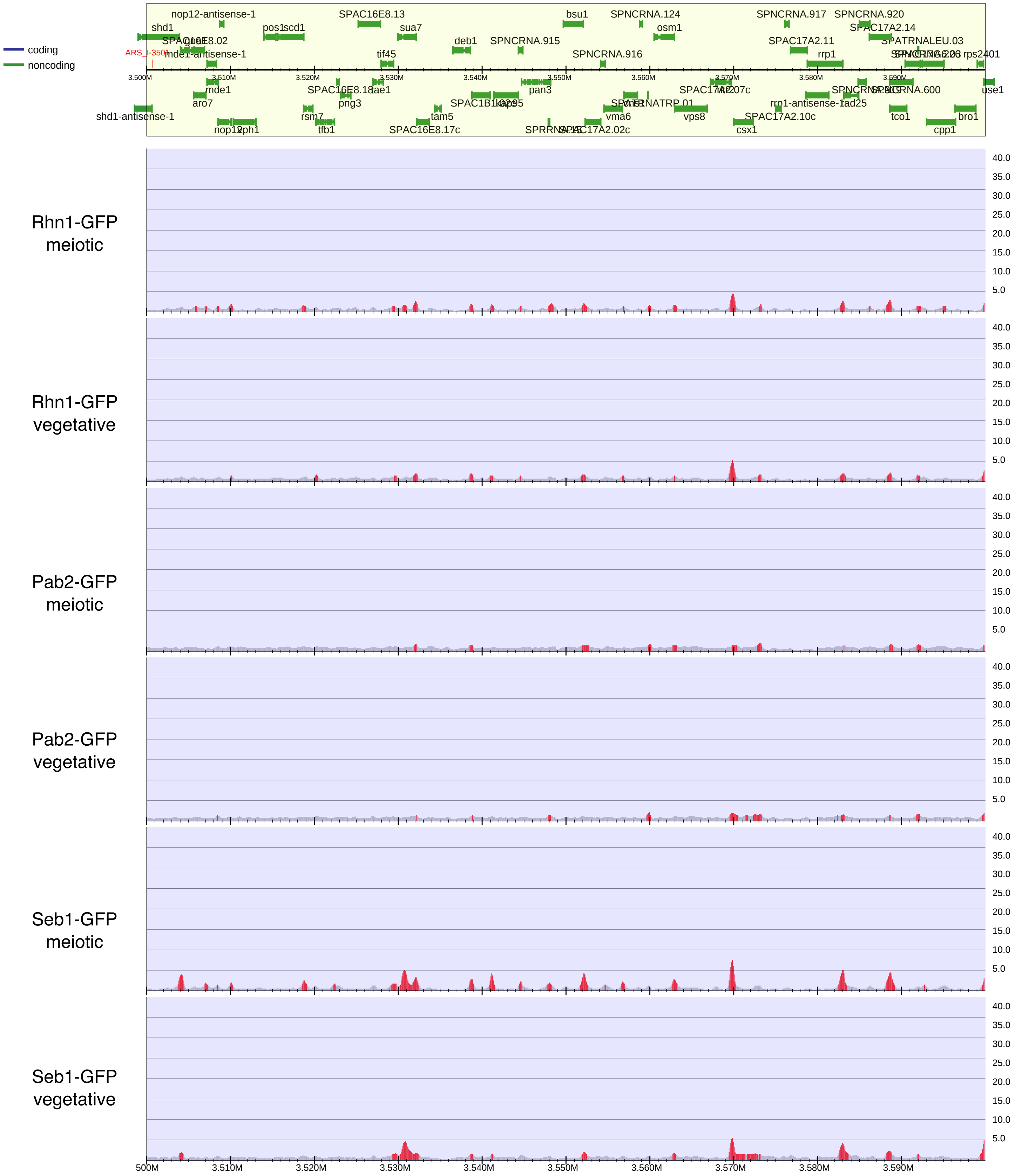
coding
noncoding
ARS_I-3501
Rhn1-GFP meiotic
Rhn1-GFP vegetative
Pab2-GFP meiotic
Pab2-GFP vegetative
Seb1-GFP meiotic
Seb1-GFP vegetative
40.0
35.0
30.0
25.0
20.0
15.0
10.0
5.0
500M
3.510M
3.520M
3.530M
3.540M
3.550M
3.560M
3.570M
3.580M
3.590M

I_1_37

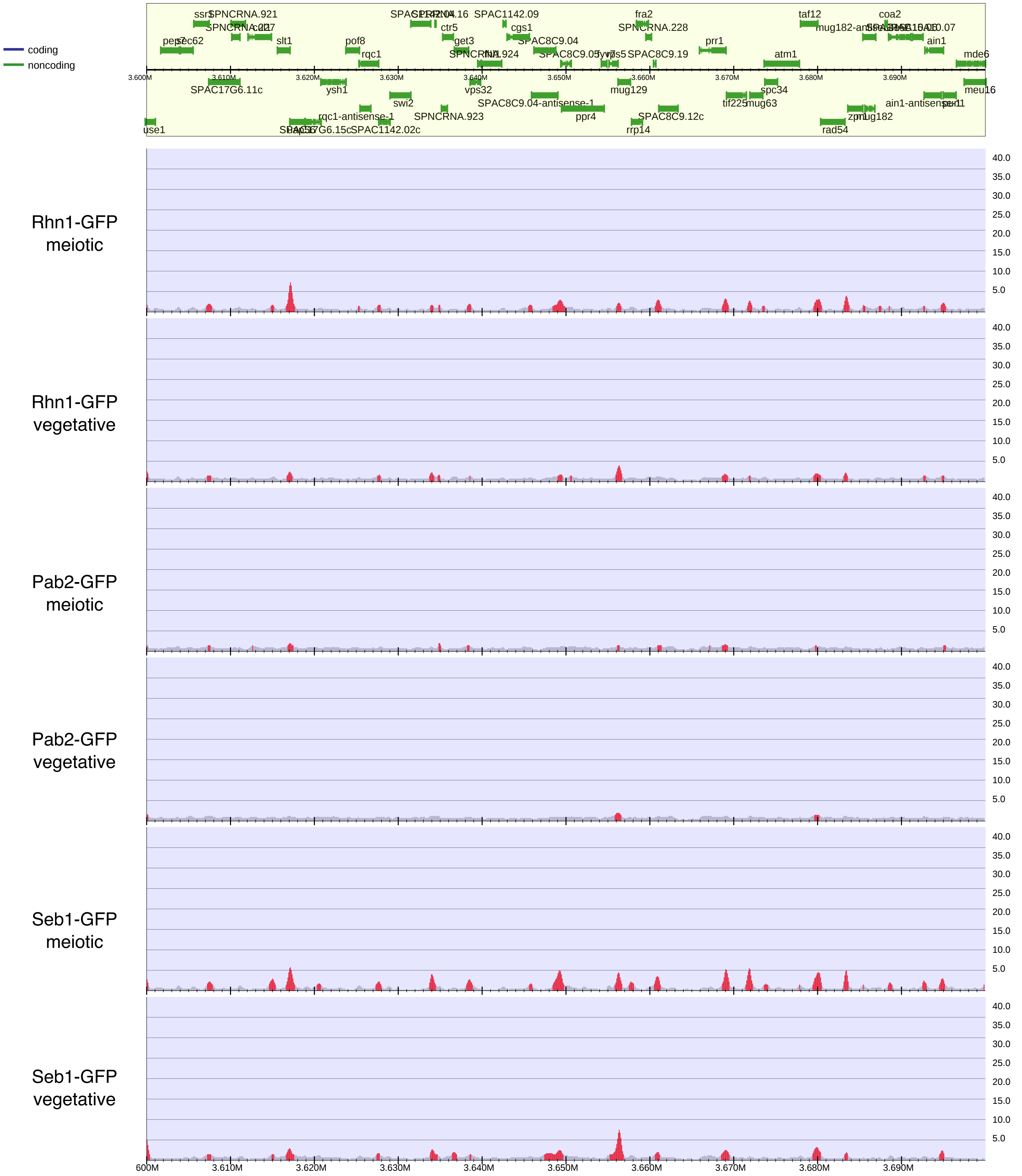

coding
noncoding
SPAC17G6.11c
ysh1
swi2
vps32
mug129
spc34
tif225
mug63
meu16
ppr4
rrp14
rad54
use1
pof8
rqc1
slt1
get3
ctr5
cgs1
fra2
prr1
atm1
taf12
coa2
ain1
mde6
SPAC8C9.04
SPAC8C9.12c
SPAC8C9.19
SPNCRNA.228
SPNCRNA.923
SPNCRNA.921
rqc1-antisense-1
ain1-antisense-1
SPAC8C9.04-antisense-1
Rhn1-GFP meiotic
Rhn1-GFP vegetative
Pab2-GFP meiotic
Pab2-GFP vegetative
Seb1-GFP meiotic
Seb1-GFP vegetative
40.0
35.0
30.0
25.0
20.0
15.0
10.0
5.0
600M
3.610M
3.620M
3.630M
3.640M
3.650M
3.660M
3.670M
3.680M
3.690M

I_1_38

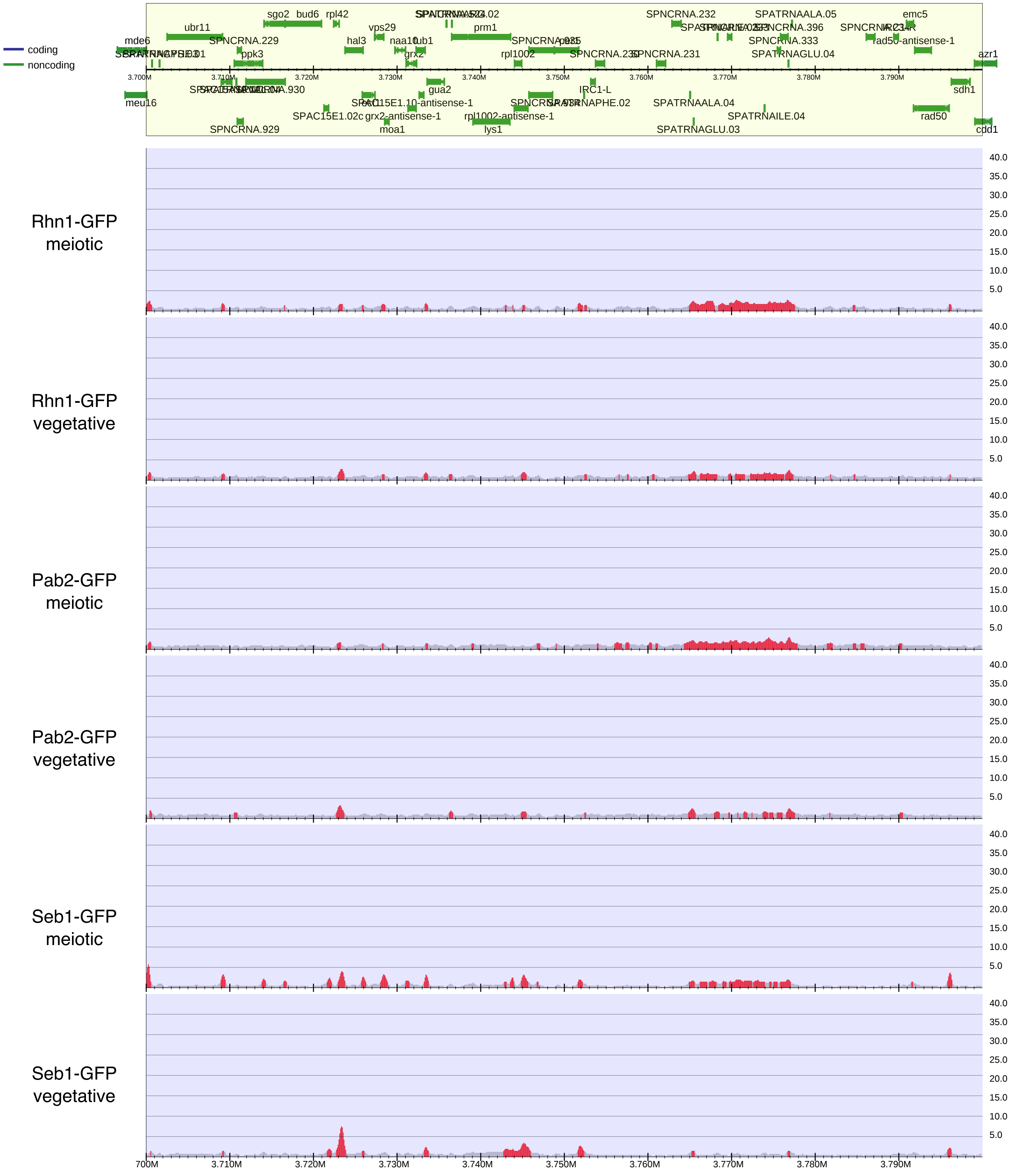
coding
noncoding
Rhn1-GFP meiotic
Rhn1-GFP vegetative
Pab2-GFP meiotic
Pab2-GFP vegetative
Seb1-GFP meiotic
Seb1-GFP vegetative
sgo2
bud6
rpl42
ubr11
mde6
SPNCRNA.229
hal3
vps29
naa10
ub1
prm1
ppk3
grx2
rpl1002
SPNCRNA.232
SPATRNAALA.05
emc5
SPNCRNA.396
SPNCRNA.333
SPATRNAGLU.04
SPNCRNA.231
rad50-antisense-1
azr1
meu16
gua2
IRC1-L
sdh1
SPNCRNA.930
SPAC15E1.10-antisense-1
SPATRNAPHE.02
SPATRNAALA.04
SPAC15E1.02c
grx2-antisense-1
rpl1002-antisense-1
SPATRNAILE.04
rad50
SPNCRNA.929
moa1
lys1
SPATRNAGLU.03
cdd1
40.0
35.0
30.0
25.0
20.0
15.0
10.0
5.0
3.700M
3.710M
3.720M
3.730M
3.740M
3.750M
3.760M
3.770M
3.780M
3.790M

I_1_39

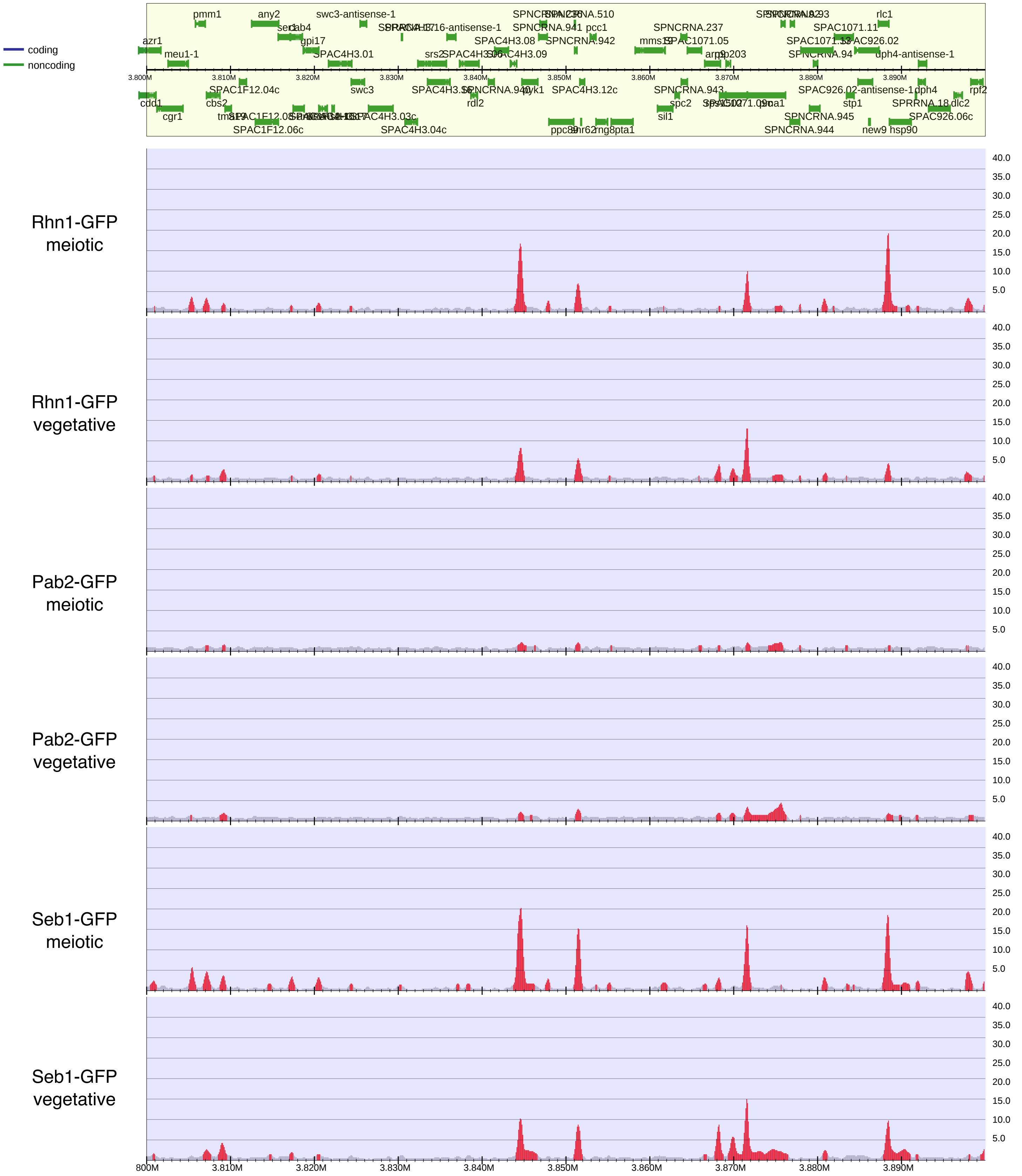
coding
noncoding
Rhn1-GFP meiotic
Rhn1-GFP vegetative
Pab2-GFP meiotic
Pab2-GFP vegetative
Seb1-GFP meiotic
Seb1-GFP vegetative
3.800M
3.810M
3.820M
3.830M
3.840M
3.850M
3.860M
3.870M
3.880M
3.890M

I_1_40

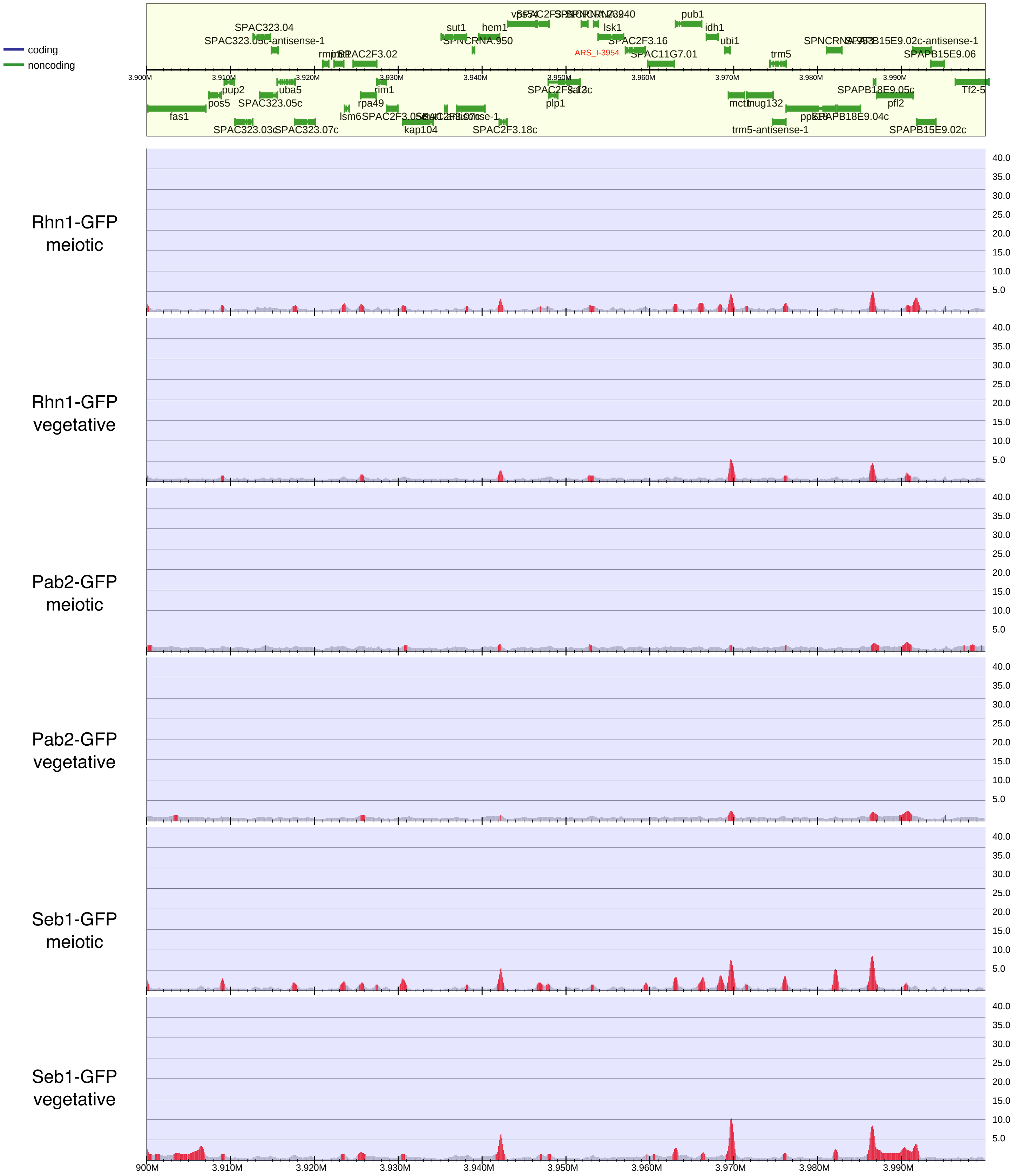

coding
noncoding
Rhn1-GFP meiotic
Rhn1-GFP vegetative
Pab2-GFP meiotic
Pab2-GFP vegetative
Seb1-GFP meiotic
Seb1-GFP vegetative
40.0
35.0
30.0
25.0
20.0
15.0
10.0
5.0
900M
3.910M
3.920M
3.930M
3.940M
3.950M
3.960M
3.970M
3.980M
3.990M
ARS_I-3954
fas1
pos5
pup2
uba5
rim1
rpa49
kap104
plp1
pub1
idh1
ubi1
trm5
trm5-antisense-1
mct1
pfl2
Tf2-5
sut1
hem1
lsk1
SPAC323.04
SPAC323.03c
SPAC323.05c
SPAC323.07c
SPAC2F3.02
SPAC2F3.16
SPAC2F3.18c
SPAC11G7.01
SPNCRNA.950
SPAPB18E9.05c
SPAPB18E9.04c
SPAPB15E9.06
SPAPB15E9.02c

I_1_41

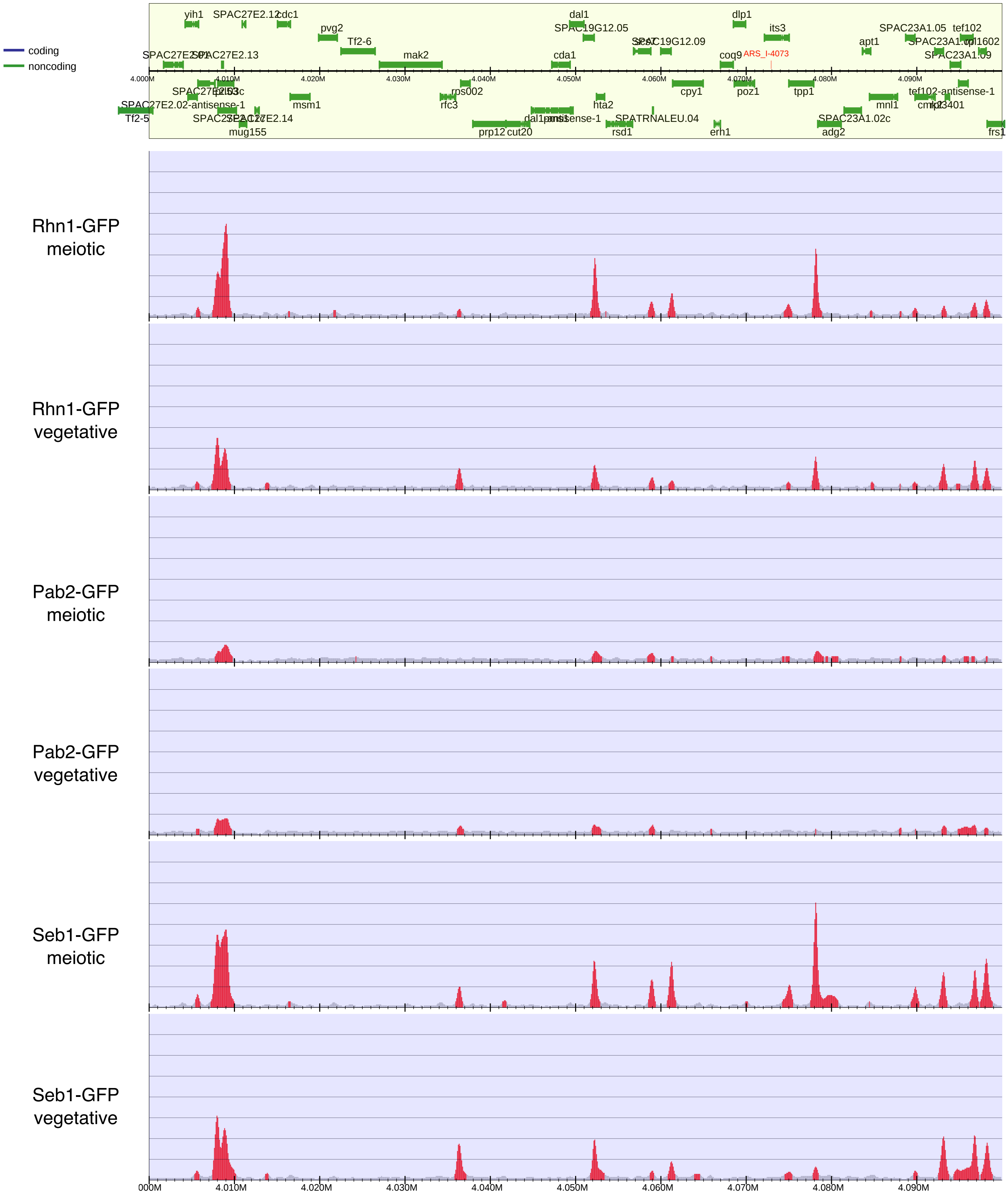
coding
noncoding
Rhn1-GFP meiotic
Rhn1-GFP vegetative
Pab2-GFP meiotic
Pab2-GFP vegetative
Seb1-GFP meiotic
Seb1-GFP vegetative
ARS_I-4073
4.000M
4.010M
4.020M
4.030M
4.040M
4.050M
4.060M
4.070M
4.080M
4.090M
40.0
35.0
30.0
25.0
20.0
15.0
10.0
5.0

I_1_42

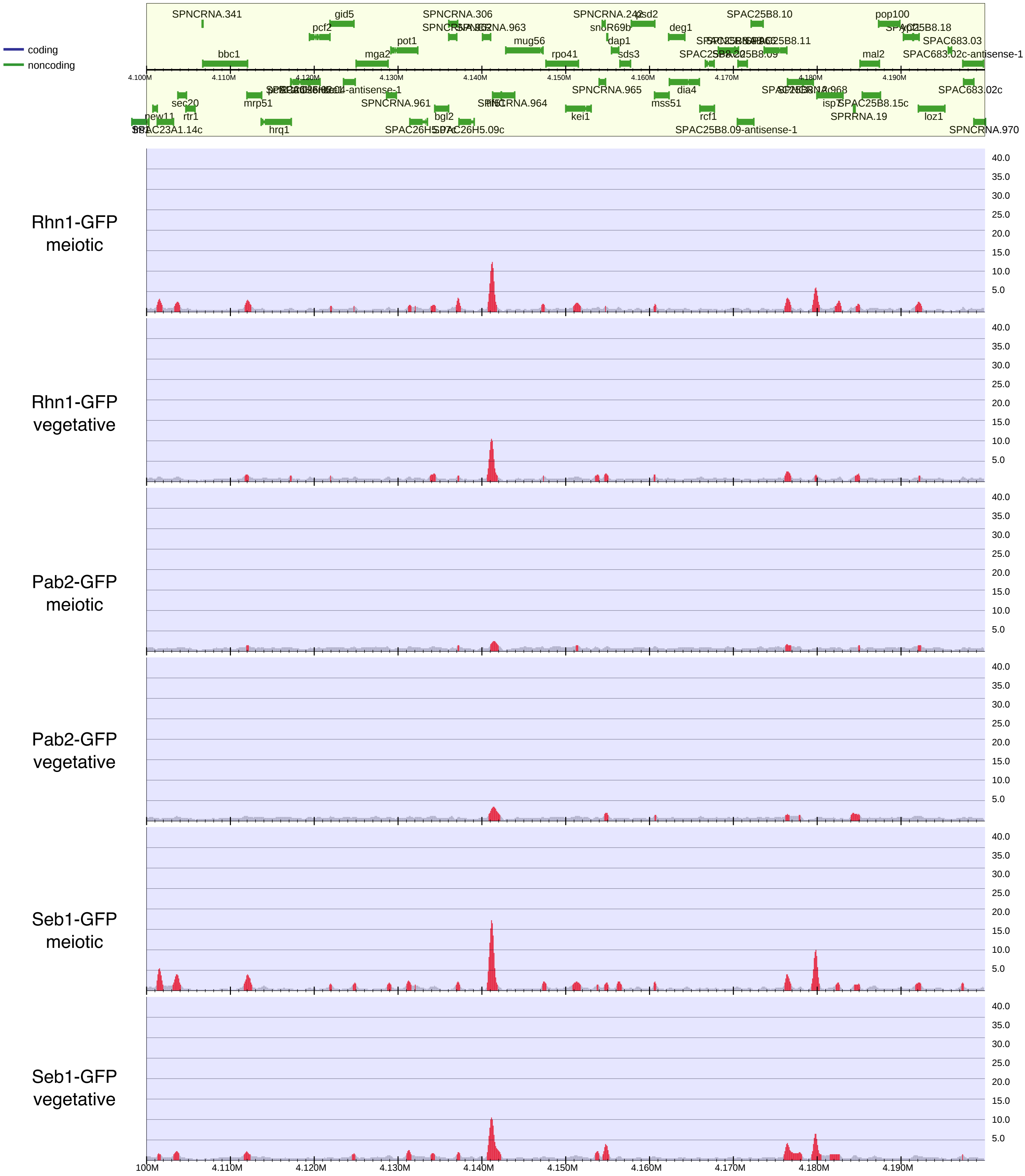
coding
noncoding
SPNCRNA.341
gid5
pcf2
SPNCRNA.306
pot1
mug56
SPNCRNA.242
psd2
snoR69b
dap1
sds3
deg1
SPAC25B8.10
pop100
SPAC25B8.18
SPAC683.03
SPAC683.02c-antisense-1
mal2
bbc1
mga2
rpo41
4.100M
4.110M
4.120M
4.130M
4.140M
4.150M
4.160M
4.170M
4.180M
4.190M
SPNCRNA.961
SPNCRNA.964
SPNCRNA.965
dia4
mss51
SPAC683.02c
sec20
mrp51
new11
rtr1
hrq1
bgl2
kei1
rcf1
SPAC25B8.09-antisense-1
isp7
SPAC25B8.15c
SPRRNA.19
loz1
SPNCRNA.970
SPAC23A1.14c
Rhn1-GFP meiotic
Rhn1-GFP vegetative
Pab2-GFP meiotic
Pab2-GFP vegetative
Seb1-GFP meiotic
Seb1-GFP vegetative
40.0
35.0
30.0
25.0
20.0
15.0
10.0
5.0
100M
4.110M
4.120M
4.130M
4.140M
4.150M
4.160M
4.170M
4.180M
4.190M

I_1_43

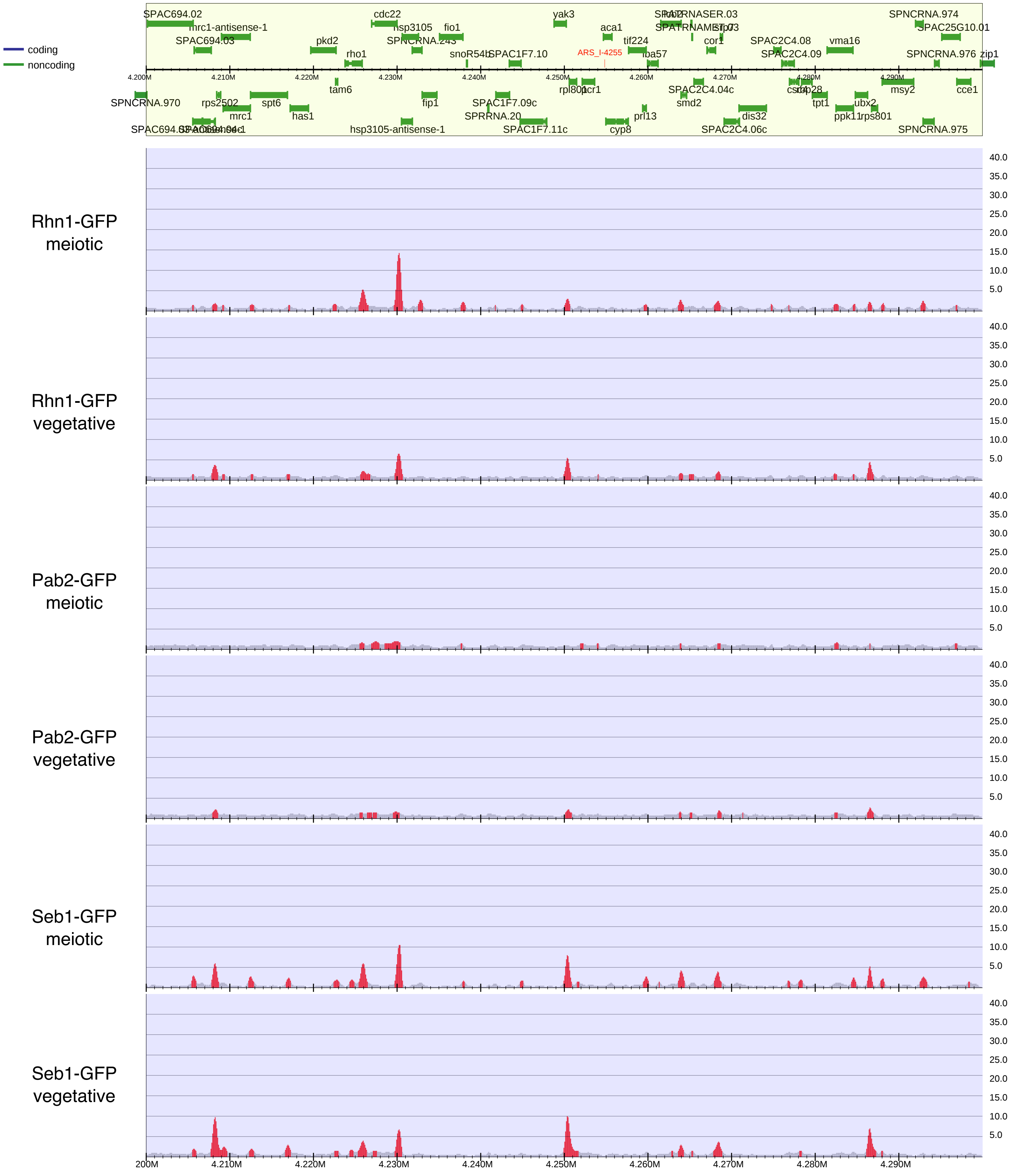
coding
noncoding
Rhn1-GFP meiotic
Rhn1-GFP vegetative
Pab2-GFP meiotic
Pab2-GFP vegetative
Seb1-GFP meiotic
Seb1-GFP vegetative
4.200M
4.210M
4.220M
4.230M
4.240M
4.250M
4.260M
4.270M
4.280M
4.290M
40.0
35.0
30.0
25.0
20.0
15.0
10.0
5.0
ARS_I-4255

I_1_44

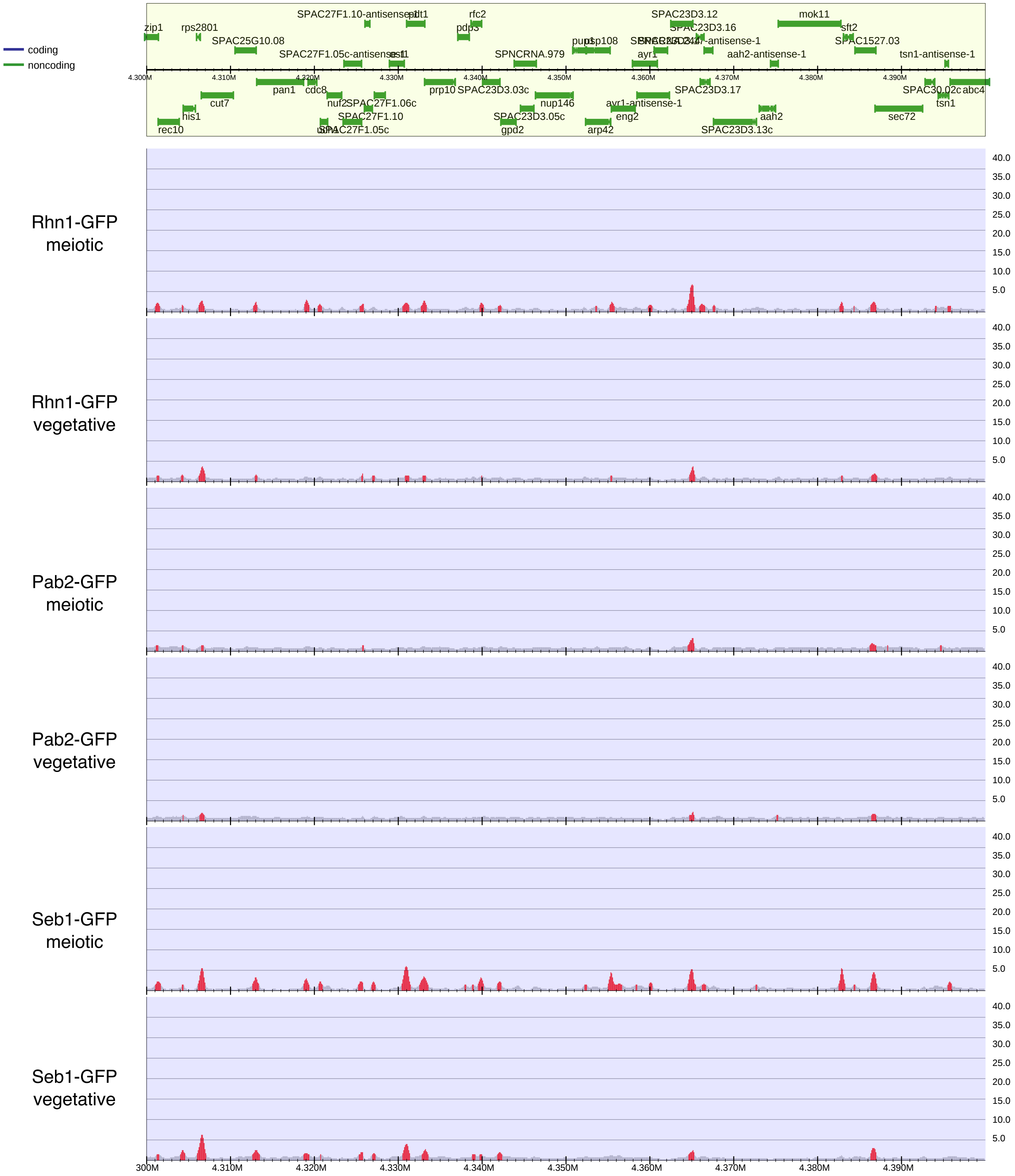

coding
noncoding
zip1
rps2801
SPAC25G10.08
SPAC27F1.10-antisense-1
pdt1
rfc2
pdp3
SPAC27F1.05c-antisense-1
SPNCRNA.979
pup1
psp108
SPAC23D3.12
SPAC23D3.16
ayr1
aah2-antisense-1
mok11
sft2
SPAC1527.03
tsn1-antisense-1
rec10
his1
cut7
pan1
cdc8
nuf2
SPAC27F1.06c
SPAC27F1.10
SPAC27F1.05c
prp10
SPAC23D3.03c
SPAC23D3.05c
gpd2
nup146
ayr1-antisense-1
eng2
arp42
SPAC23D3.17
SPAC23D3.13c
aah2
sec72
SPAC30.02c
abc4
tsn1
Rhn1-GFP meiotic
Rhn1-GFP vegetative
Pab2-GFP meiotic
Pab2-GFP vegetative
Seb1-GFP meiotic
Seb1-GFP vegetative
40.0
35.0
30.0
25.0
20.0
15.0
10.0
5.0
300M
4.310M
4.320M
4.330M
4.340M
4.350M
4.360M
4.370M
4.380M
4.390M

I_1_45

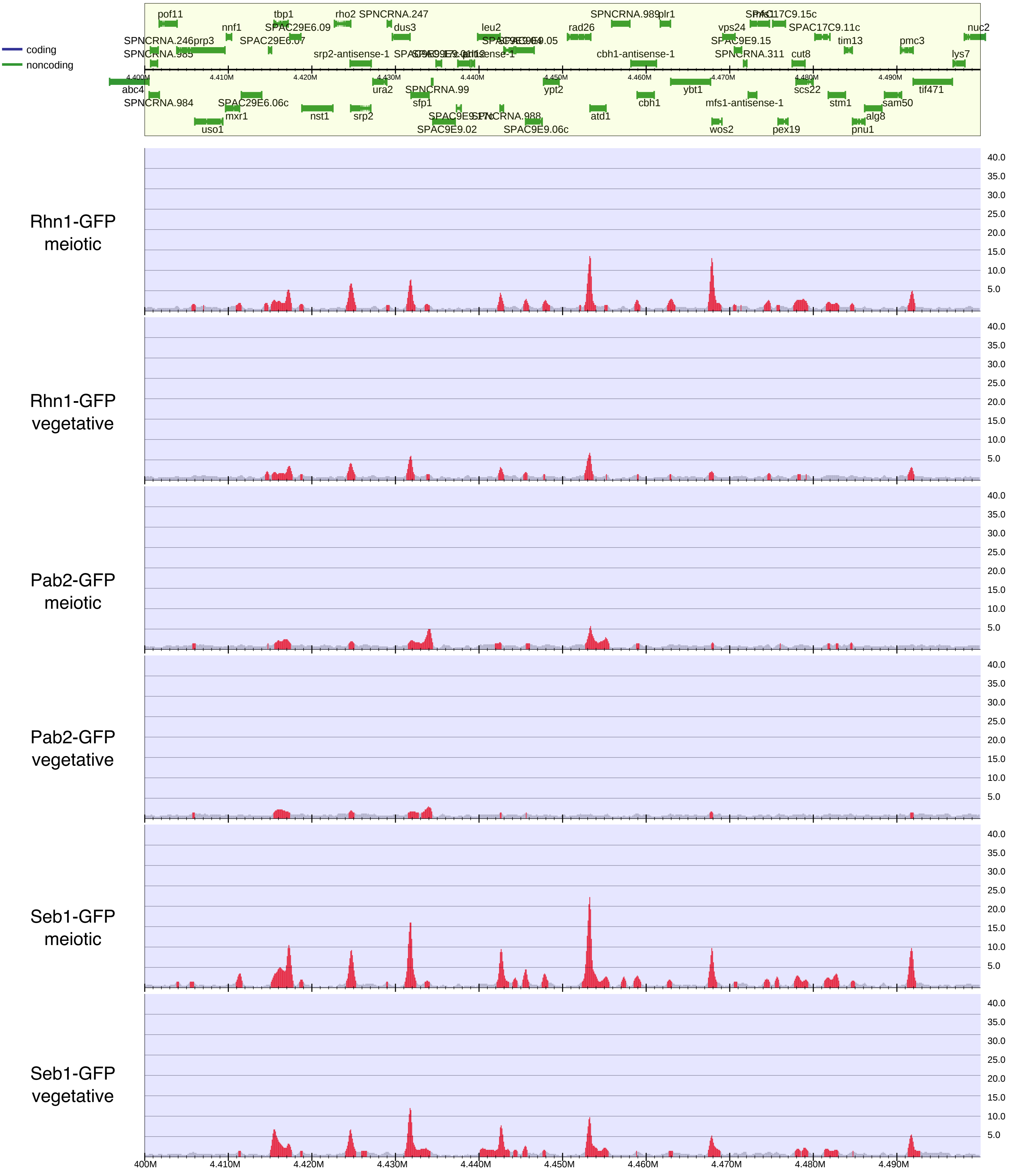

coding
noncoding
Rhn1-GFP meiotic
Rhn1-GFP vegetative
Pab2-GFP meiotic
Pab2-GFP vegetative
Seb1-GFP meiotic
Seb1-GFP vegetative
400M
4.410M
4.420M
4.430M
4.440M
4.450M
4.460M
4.470M
4.480M
4.490M

I_1_46

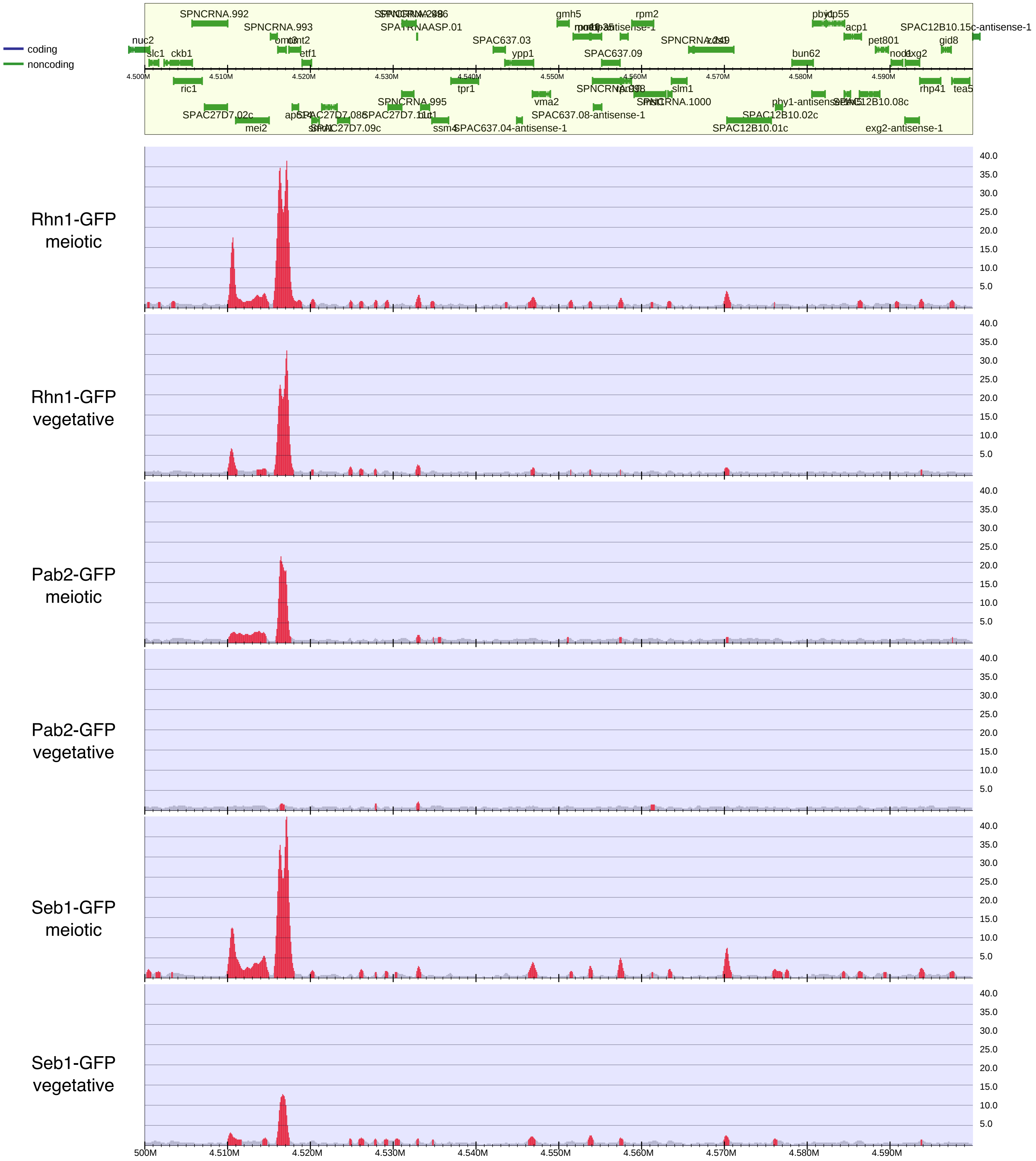
coding
noncoding
Rhn1-GFP meiotic
Rhn1-GFP vegetative
Pab2-GFP meiotic
Pab2-GFP vegetative
Seb1-GFP meiotic
Seb1-GFP vegetative
500M
4.510M
4.520M
4.530M
4.540M
4.550M
4.560M
4.570M
4.580M
4.590M

I_1_47

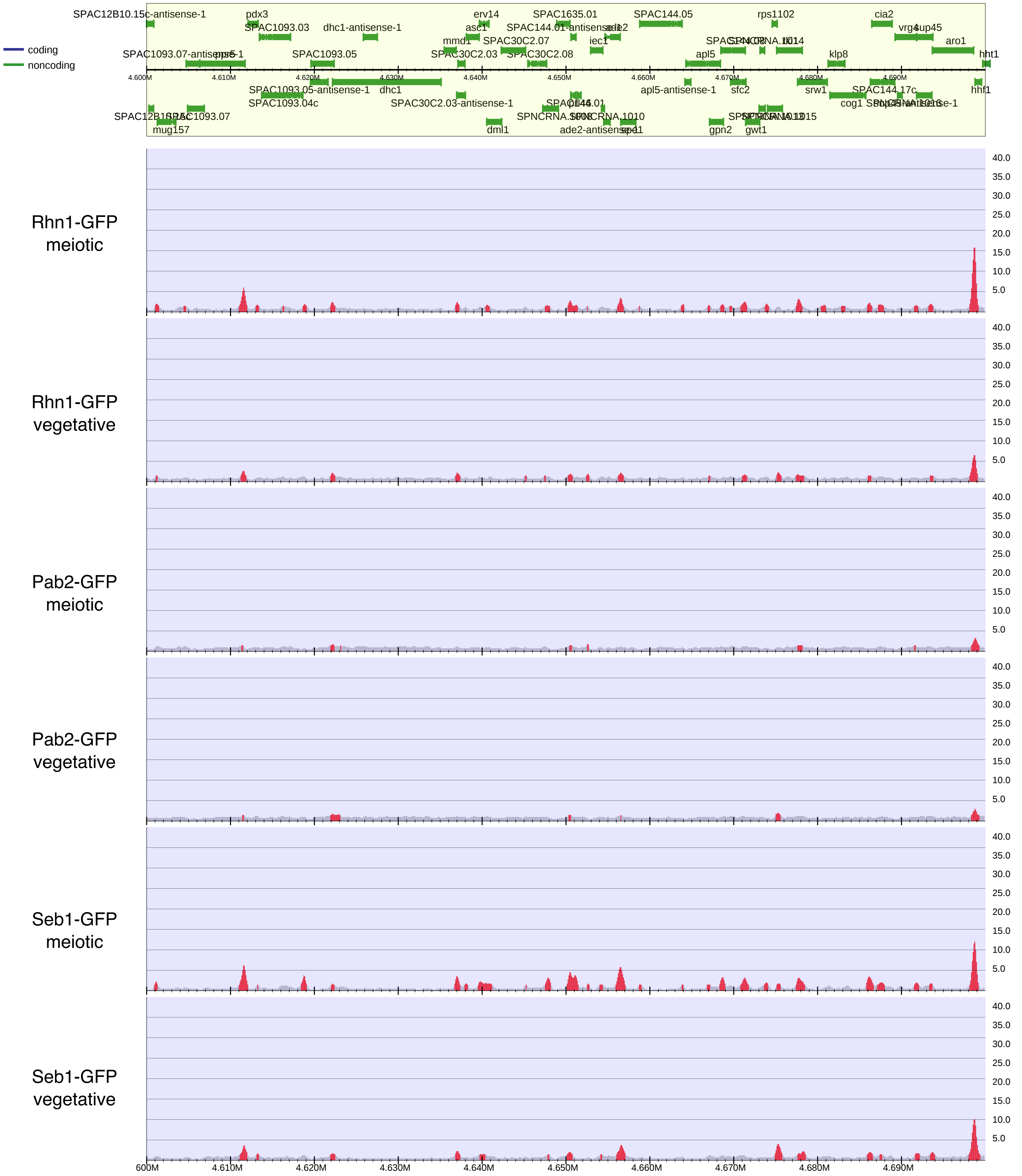

coding
noncoding
Rhn1-GFP meiotic
Rhn1-GFP vegetative
Pab2-GFP meiotic
Pab2-GFP vegetative
Seb1-GFP meiotic
Seb1-GFP vegetative
600M
4.610M
4.620M
4.630M
4.640M
4.650M
4.660M
4.670M
4.680M
4.690M

I_1_48

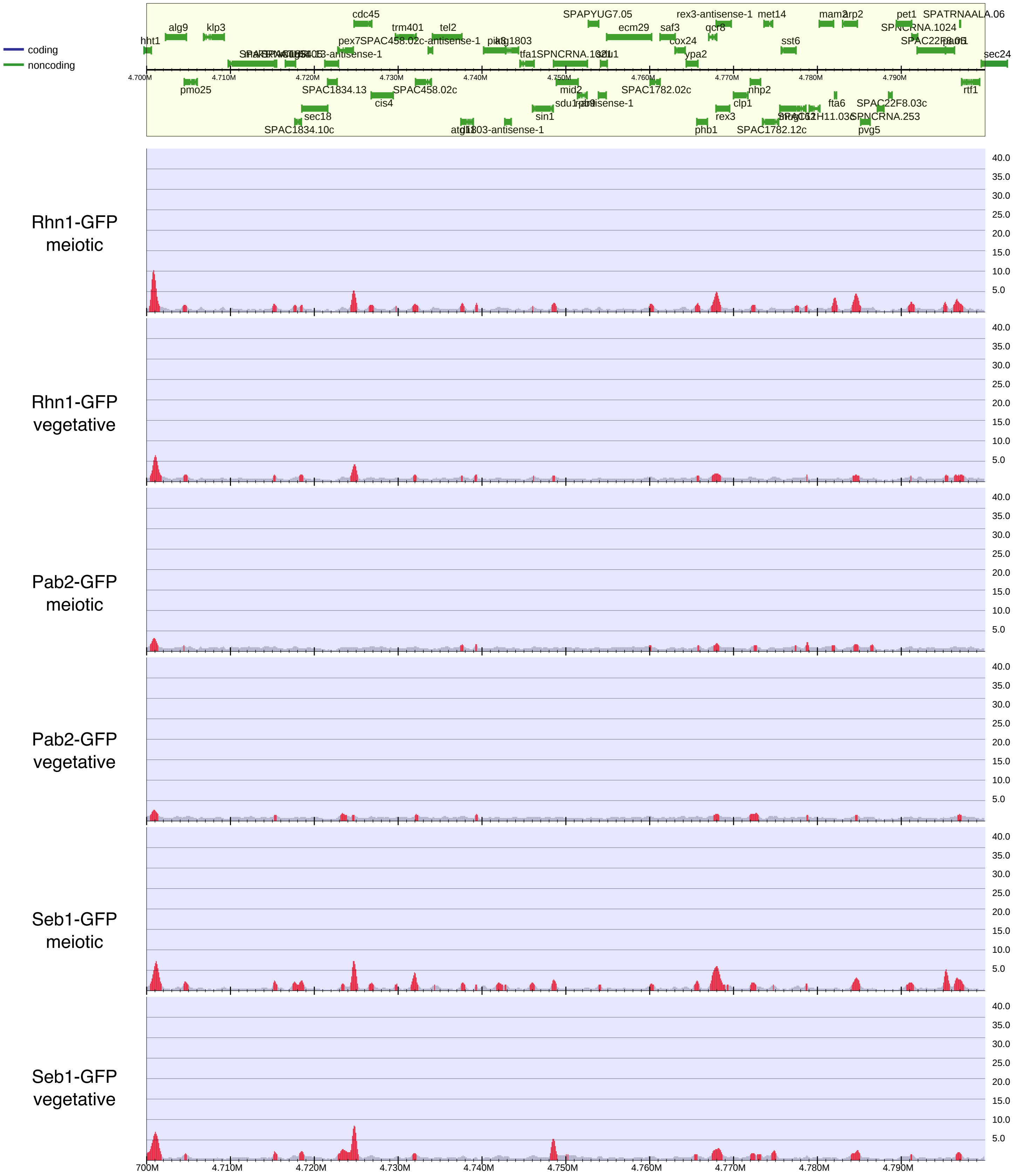
coding
noncoding
hht1
alg9
klp3
pmo25
cdc45
trm401
tel2
SPAC1834.13
SPAC458.02c
cis4
sec18
SPAC1834.10c
SPAPYUG7.05
ecm29
saf3
cox24
ypa2
mid2
sin1
rex3-antisense-1
met14
qcr8
sst6
clp1
nhp2
rex3
phb1
SPAC1782.02c
SPAC1782.12c
mam2
arp2
fta6
SPAC22F8.03c
SPNCRNA.253
pvg5
pet1
SPATRNAALA.06
SPNCRNA.1024
sec24
rtf1
4.700M
4.710M
4.720M
4.730M
4.740M
4.750M
4.760M
4.770M
4.780M
4.790M
Rhn1-GFP meiotic
Rhn1-GFP vegetative
Pab2-GFP meiotic
Pab2-GFP vegetative
Seb1-GFP meiotic
Seb1-GFP vegetative
40.0
35.0
30.0
25.0
20.0
15.0
10.0
5.0
700M
4.710M
4.720M
4.730M
4.740M
4.750M
4.760M
4.770M
4.780M
4.790M

I_1_49

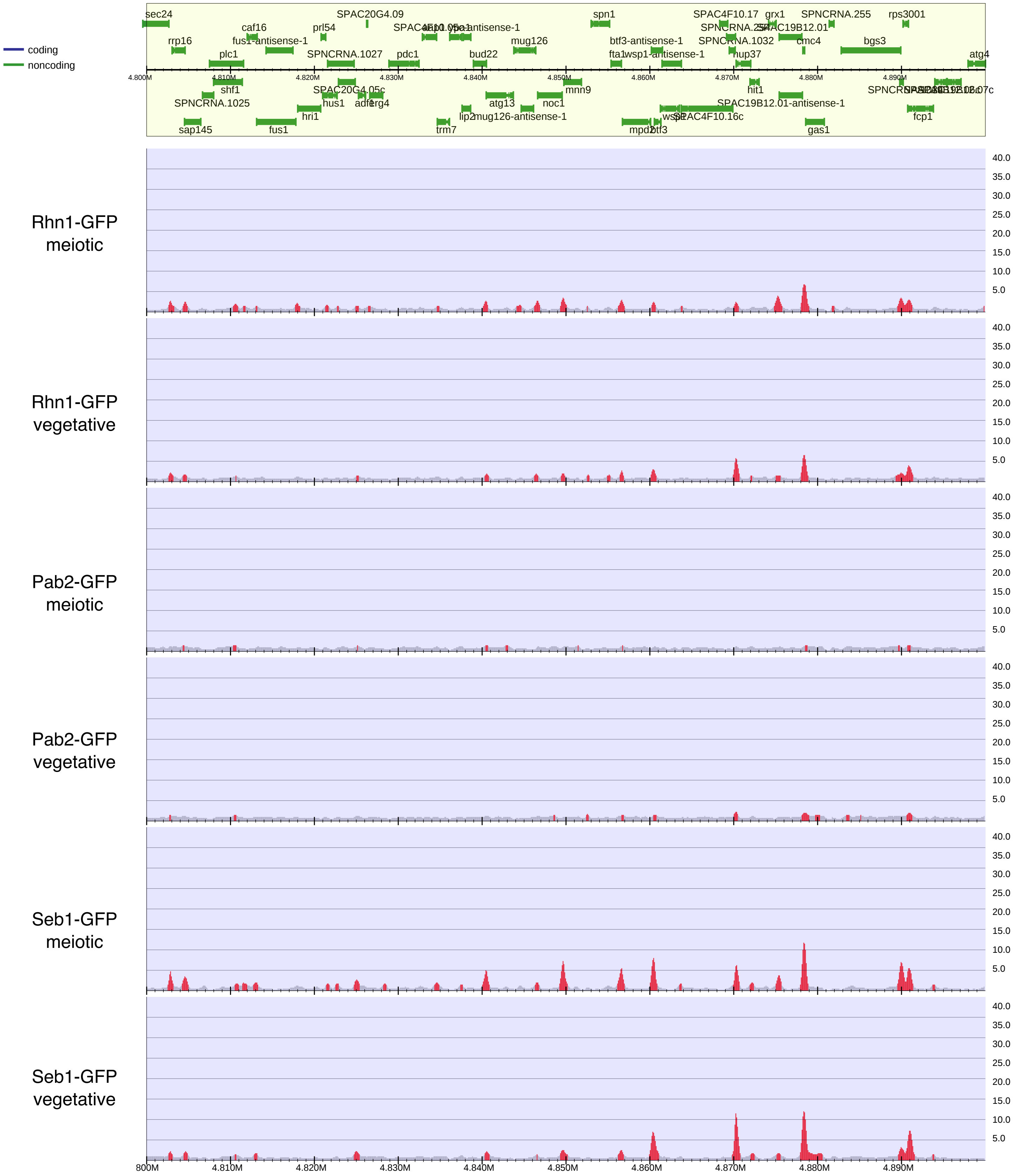
coding
noncoding
Rhn1-GFP meiotic
Rhn1-GFP vegetative
Pab2-GFP meiotic
Pab2-GFP vegetative
Seb1-GFP meiotic
Seb1-GFP vegetative
800M
4.810M
4.820M
4.830M
4.840M
4.850M
4.860M
4.870M
4.880M
4.890M

I_1_50

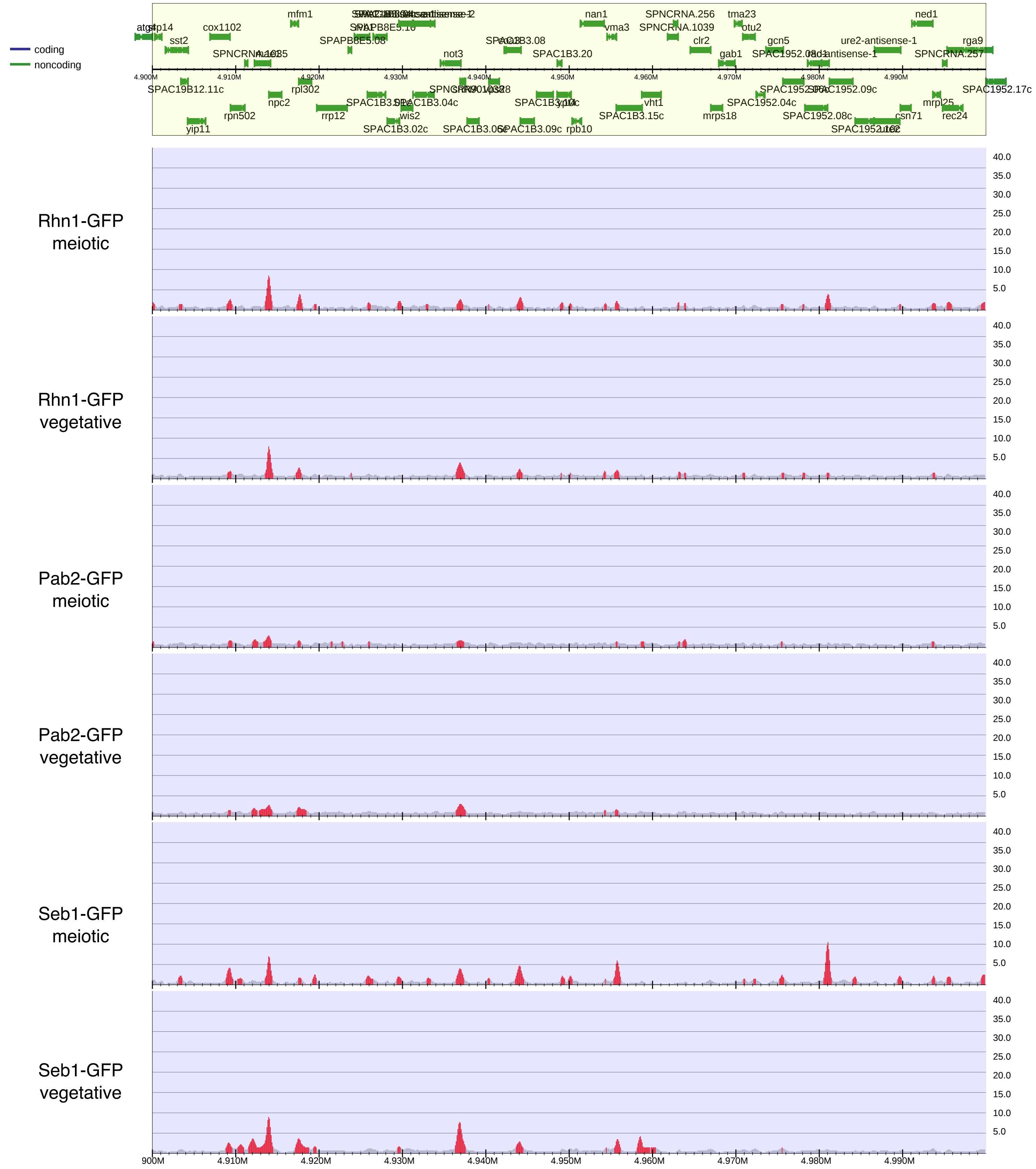
coding
noncoding
Rhn1-GFP meiotic
Rhn1-GFP vegetative
Pab2-GFP meiotic
Pab2-GFP vegetative
Seb1-GFP meiotic
Seb1-GFP vegetative
40.0
35.0
30.0
25.0
20.0
15.0
10.0
5.0
900M
4.910M
4.920M
4.930M
4.940M
4.950M
4.960M
4.970M
4.980M
4.990M

I_1_51

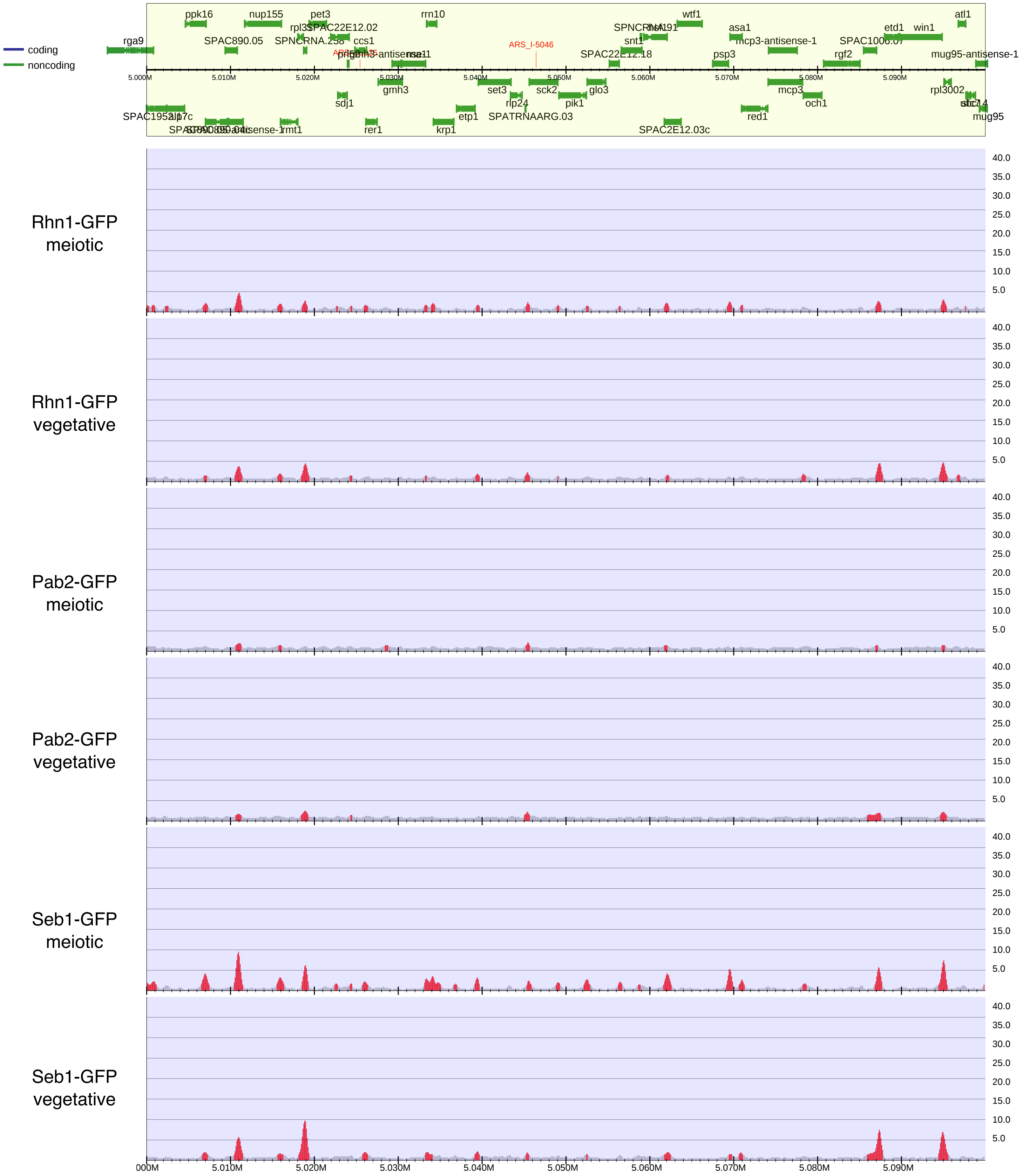

coding
noncoding
ppk16
nup155
pet3
rrn10
wtf1
atl1
rga9
SPAC890.05
SPAC22E12.02
ccs1
ARS_I-5046
asa1
etd1
win1
mcp3-antisense-1
SPAC22E12.18
psp3
rgf2
mug95-antisense-1
gmh3
set3
sck2
glo3
mcp3
rpl3002
sdj1
rlp24
pik1
och1
etp1
SPATRNAARG.03
red1
rer1
krp1
rmt1
SPAC2E12.03c
mug95
5.000M
5.010M
5.020M
5.030M
5.040M
5.050M
5.060M
5.070M
5.080M
5.090M
Rhn1-GFP meiotic
Rhn1-GFP vegetative
Pab2-GFP meiotic
Pab2-GFP vegetative
Seb1-GFP meiotic
Seb1-GFP vegetative
40.0
35.0
30.0
25.0
20.0
15.0
10.0
5.0

I_1_52

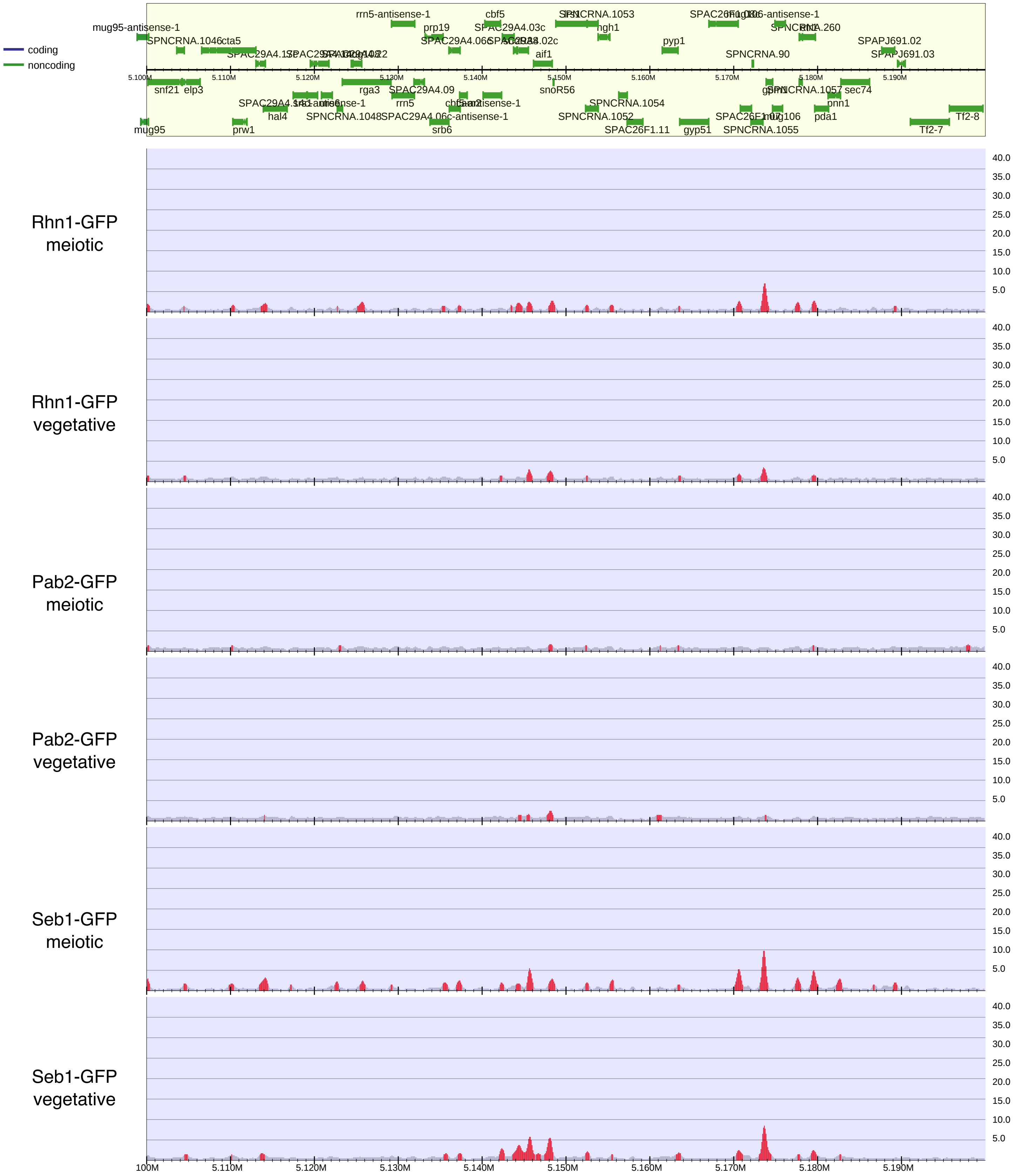
coding
noncoding
mug95-antisense-1
rrn5-antisense-1
cbf5
prp19
SPAC29A4.03c
ngh1
pyp1
aif1
SPNCRNA.90
SPAPJ691.02
SPAPJ691.03
snf21
elp3
rga3
SPAC29A4.09
snoR56
sec74
hal4
rrn5
SPNCRNA.1054
pnn1
SPNCRNA.1048
SPNCRNA.1052
pda1
Tf2-8
mug95
prw1
srb6
SPAC26F1.11
gyp51
SPNCRNA.1055
Tf2-7
5.100M
5.110M
5.120M
5.130M
5.140M
5.150M
5.160M
5.170M
5.180M
5.190M
Rhn1-GFP meiotic
Rhn1-GFP vegetative
Pab2-GFP meiotic
Pab2-GFP vegetative
Seb1-GFP meiotic
Seb1-GFP vegetative
40.0
35.0
30.0
25.0
20.0
15.0
10.0
5.0
100M
5.110M
5.120M
5.130M
5.140M
5.150M
5.160M
5.170M
5.180M
5.190M

I_1_53

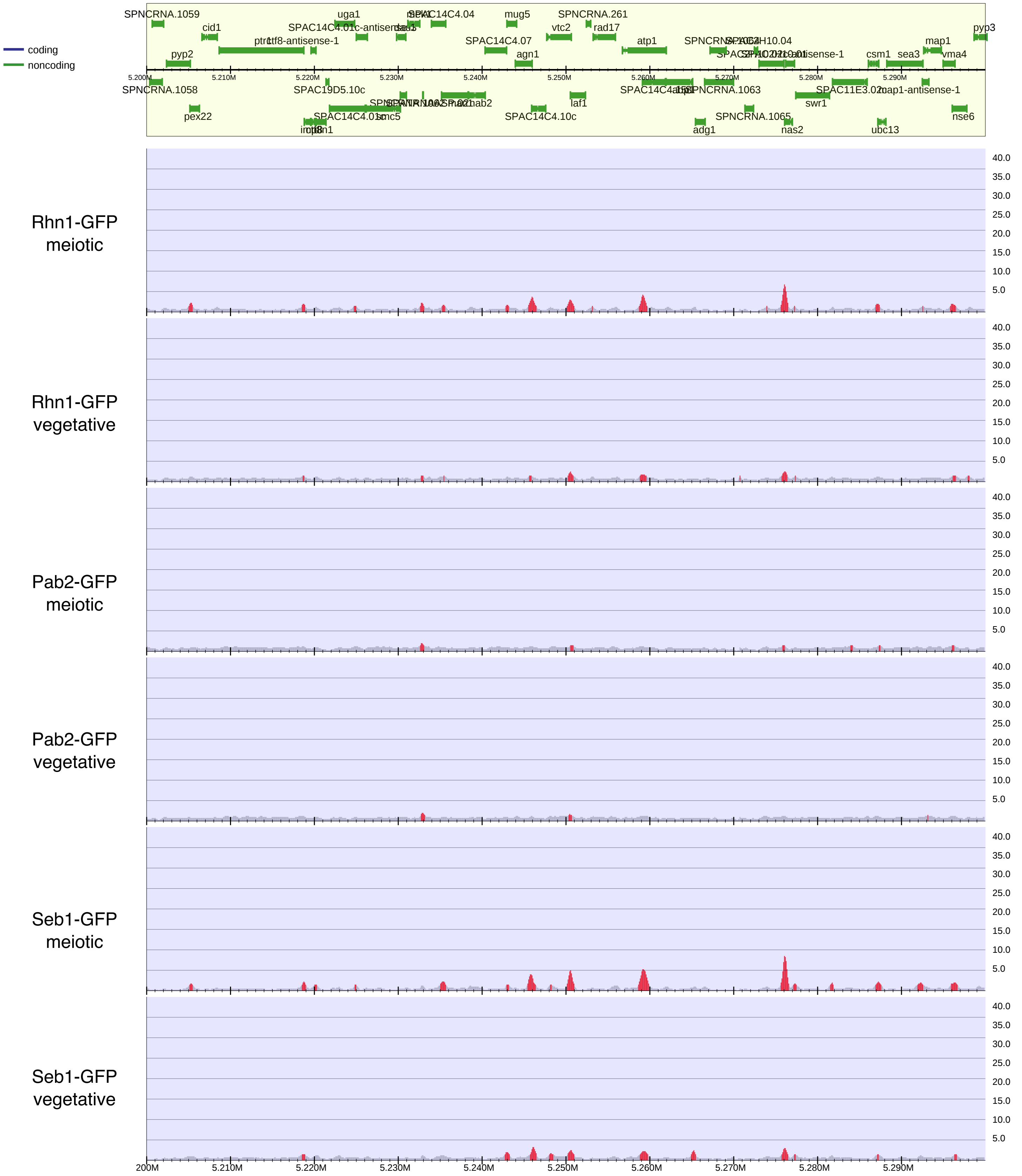
coding
noncoding
Rhn1-GFP meiotic
Rhn1-GFP vegetative
Pab2-GFP meiotic
Pab2-GFP vegetative
Seb1-GFP meiotic
Seb1-GFP vegetative
40.0
35.0
30.0
25.0
20.0
15.0
10.0
5.0
200M
5.210M
5.220M
5.230M
5.240M
5.250M
5.260M
5.270M
5.280M
5.290M
SPNCRNA.1059
uga1
SPAC14C4.04
mug5
SPNCRNA.261
cid1
vtc2
rad17
pyp3
agn1
atp1
map1
pyp2
SPAC14C4.07
csm1
sea3
vma4
SPNCRNA.1058
SPAC19D5.10c
SPNCRNA.1063
swr1
laf1
pex22
SPAC14C4.10c
nse6
SPNCRNA.1065
adg1
nas2
ubc13

I_1_54

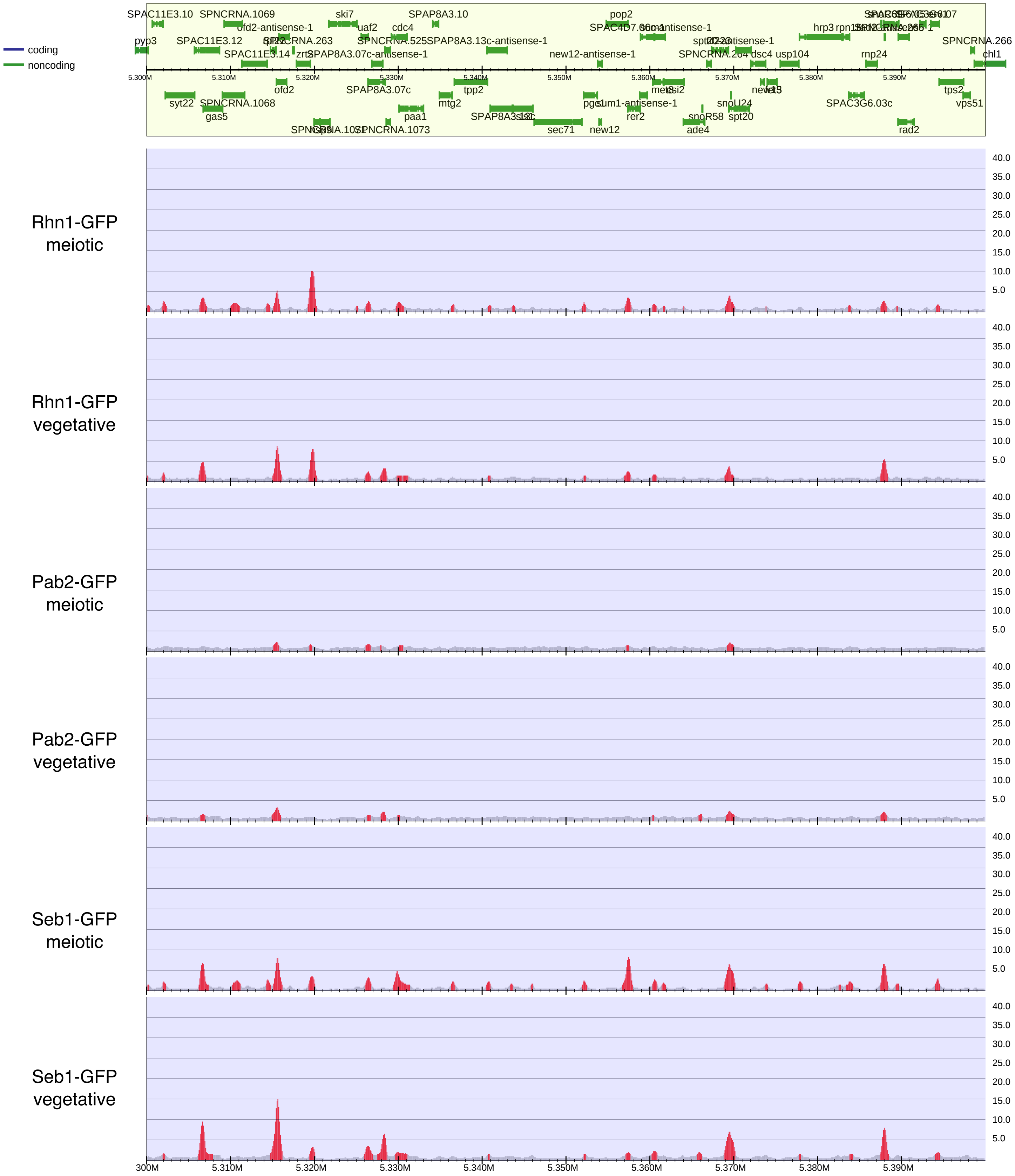

coding
noncoding
Rhn1-GFP meiotic
Rhn1-GFP vegetative
Pab2-GFP meiotic
Pab2-GFP vegetative
Seb1-GFP meiotic
Seb1-GFP vegetative

I_1_55

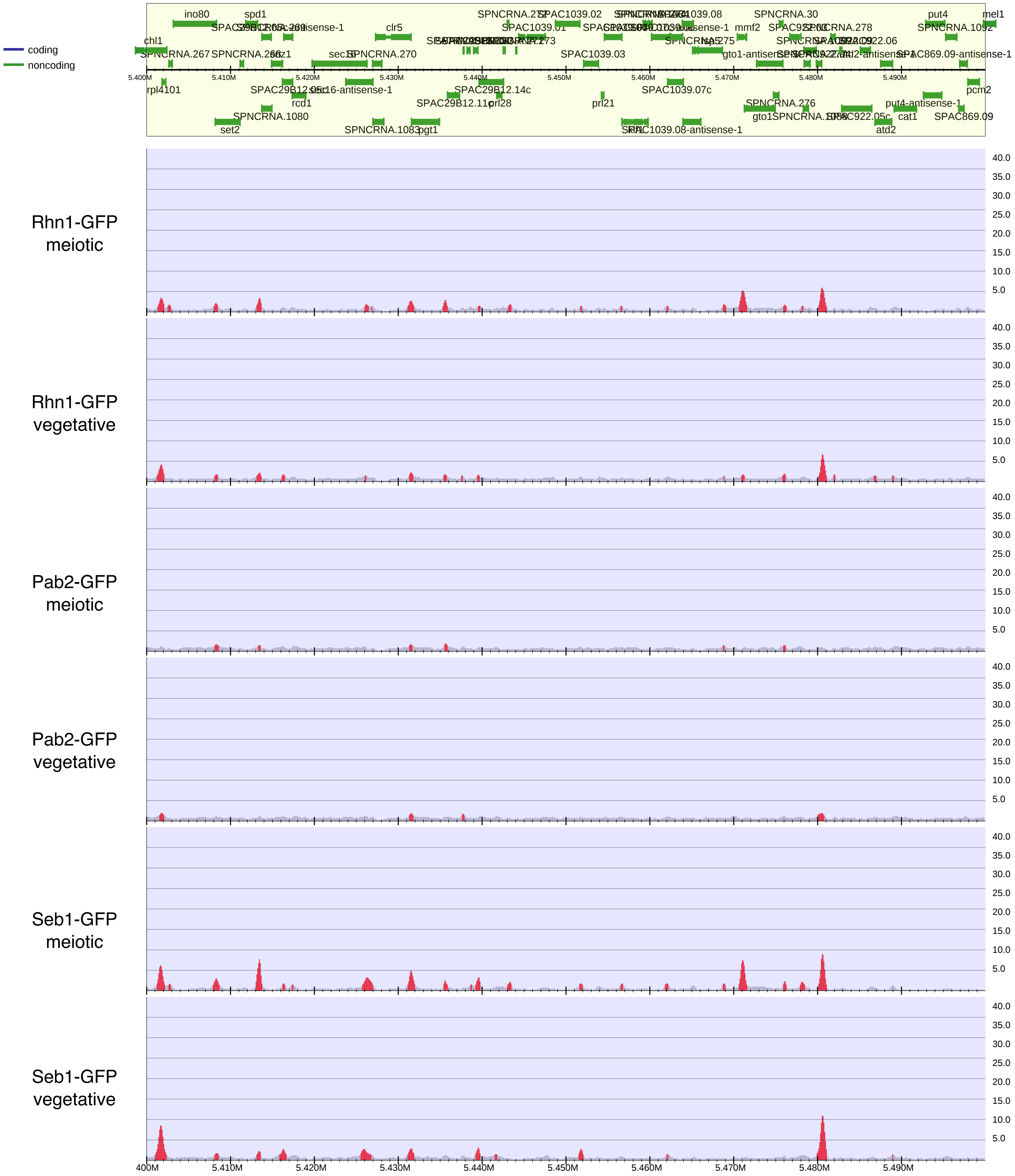

coding
noncoding
Rhn1-GFP meiotic
Rhn1-GFP vegetative
Pab2-GFP meiotic
Pab2-GFP vegetative
Seb1-GFP meiotic
Seb1-GFP vegetative
40.0
35.0
30.0
25.0
20.0
15.0
10.0
5.0
400M
5.410M
5.420M
5.430M
5.440M
5.450M
5.460M
5.470M
5.480M
5.490M

I_1_56

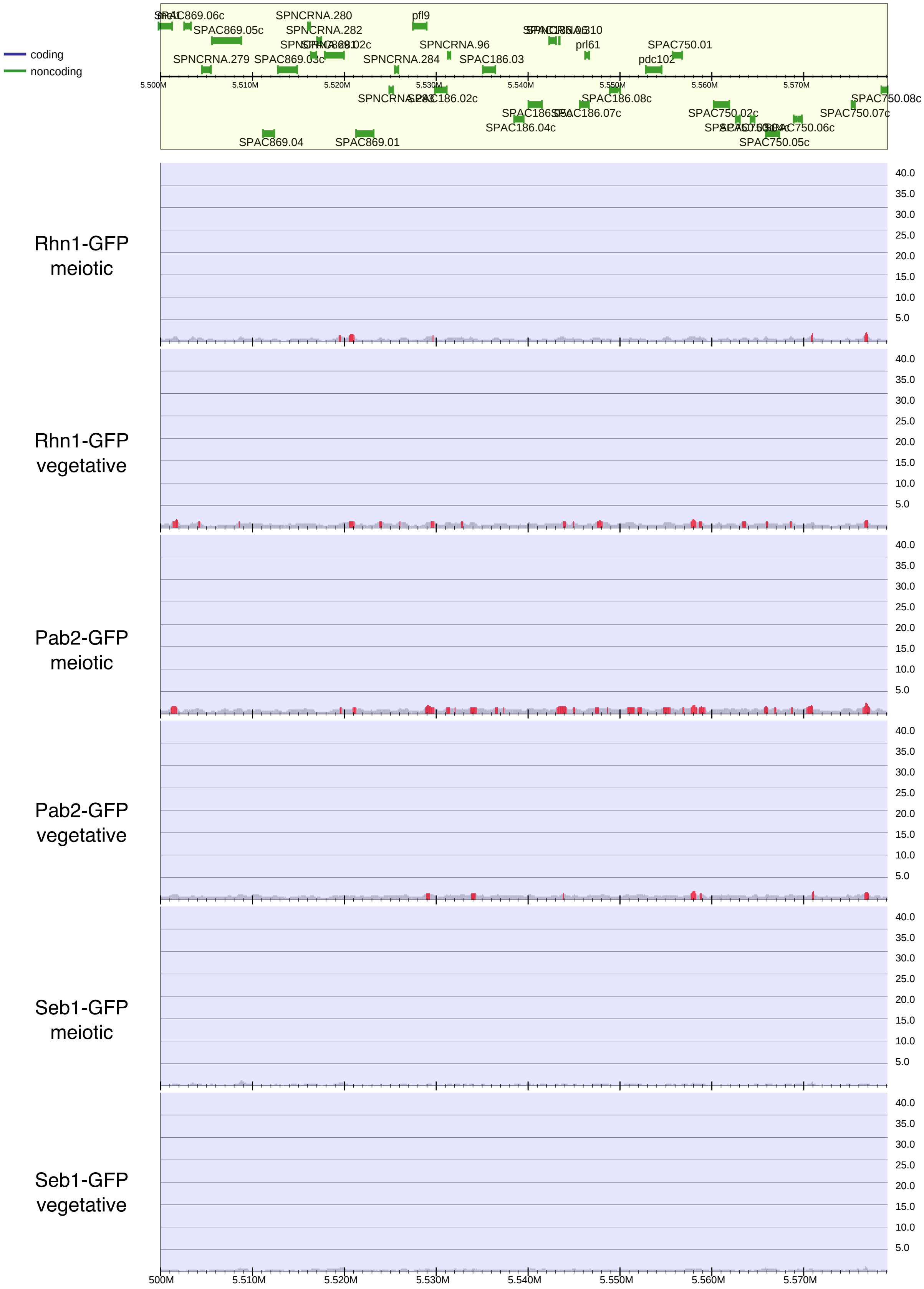

coding
noncoding
SPAC869.06c
SPNCRNA.280
pfl9
SPAC869.05c
SPNCRNA.282
prl61
SPAC750.01
SPNCRNA.96
SPNCRNA.279
SPAC869.03c
SPNCRNA.284
SPAC186.03
pdc102
5.500M
5.510M
5.520M
5.530M
5.540M
5.550M
5.560M
5.570M
SPAC186.02c
SPAC186.08c
SPAC750.08c
SPAC186.07c
SPAC750.02c
SPAC750.07c
SPAC186.04c
SPAC750.06c
SPAC869.04
SPAC869.01
SPAC750.05c
Rhn1-GFP
meiotic
Rhn1-GFP
vegetative
Pab2-GFP
meiotic
Pab2-GFP
vegetative
Seb1-GFP
meiotic
Seb1-GFP
vegetative
40.0
35.0
30.0
25.0
20.0
15.0
10.0
5.0
500M
5.510M
5.520M
5.530M
5.540M
5.550M
5.560M
5.570M

II_1_1

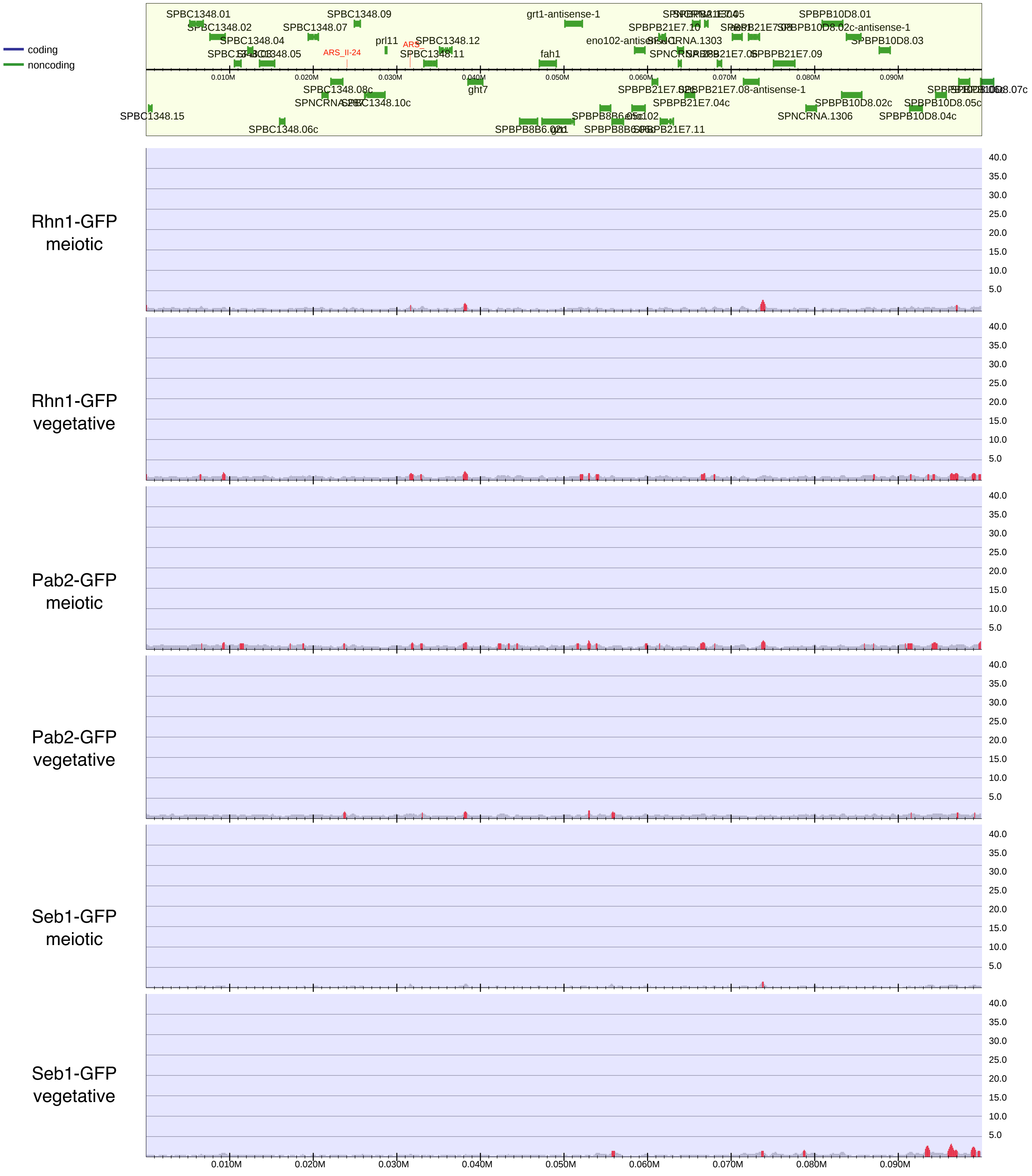

coding
noncoding
Rhn1-GFP meiotic
Rhn1-GFP vegetative
Pab2-GFP meiotic
Pab2-GFP vegetative
Seb1-GFP meiotic
Seb1-GFP vegetative
0.010M
0.020M
0.030M
0.040M
0.050M
0.060M
0.070M
0.080M
0.090M

II_1_2

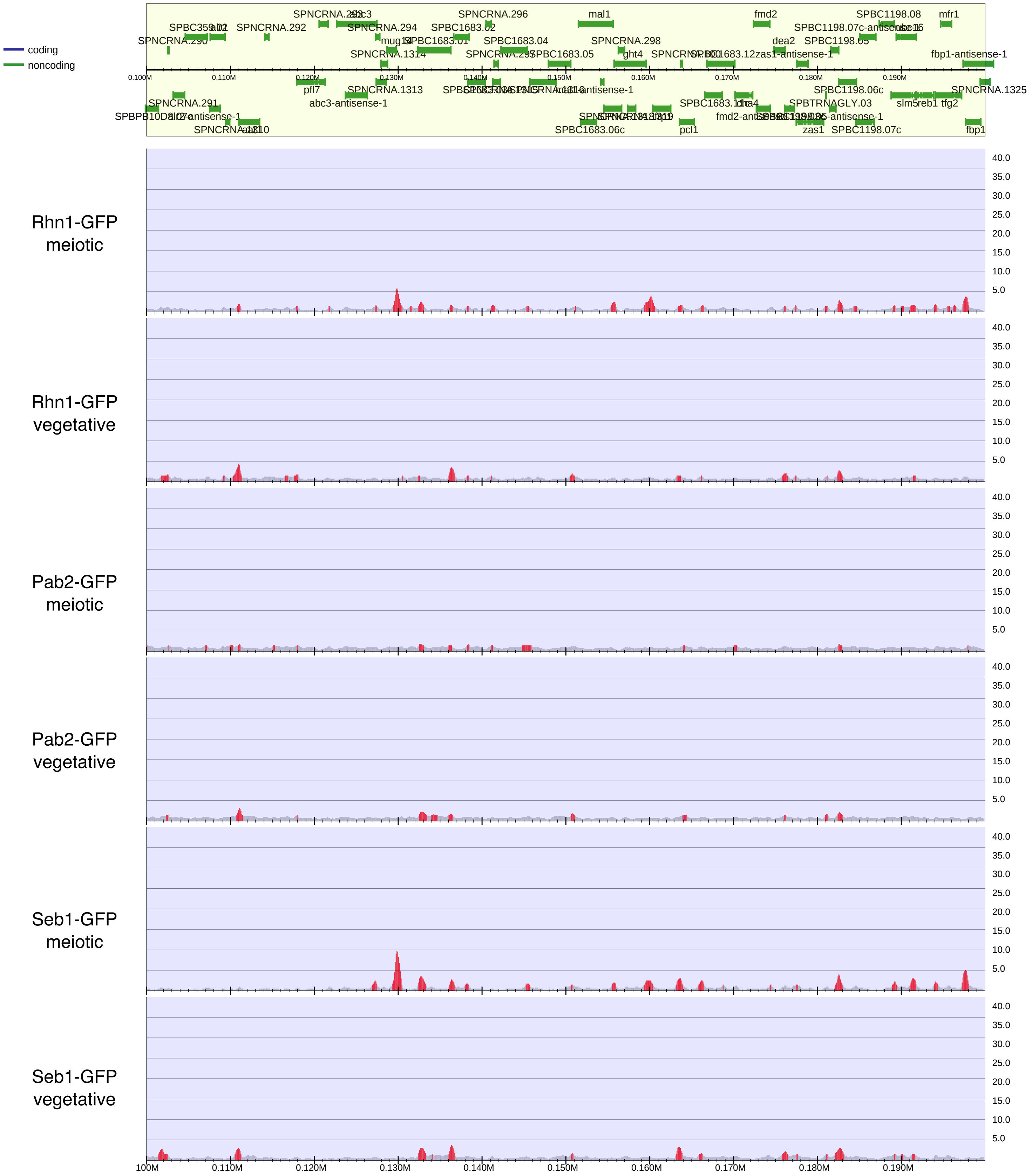

coding
noncoding
Rhn1-GFP meiotic
Rhn1-GFP vegetative
Pab2-GFP meiotic
Pab2-GFP vegetative
Seb1-GFP meiotic
Seb1-GFP vegetative
40.0
35.0
30.0
25.0
20.0
15.0
10.0
5.0
100M
0.110M
0.120M
0.130M
0.140M
0.150M
0.160M
0.170M
0.180M
0.190M

II_1_3

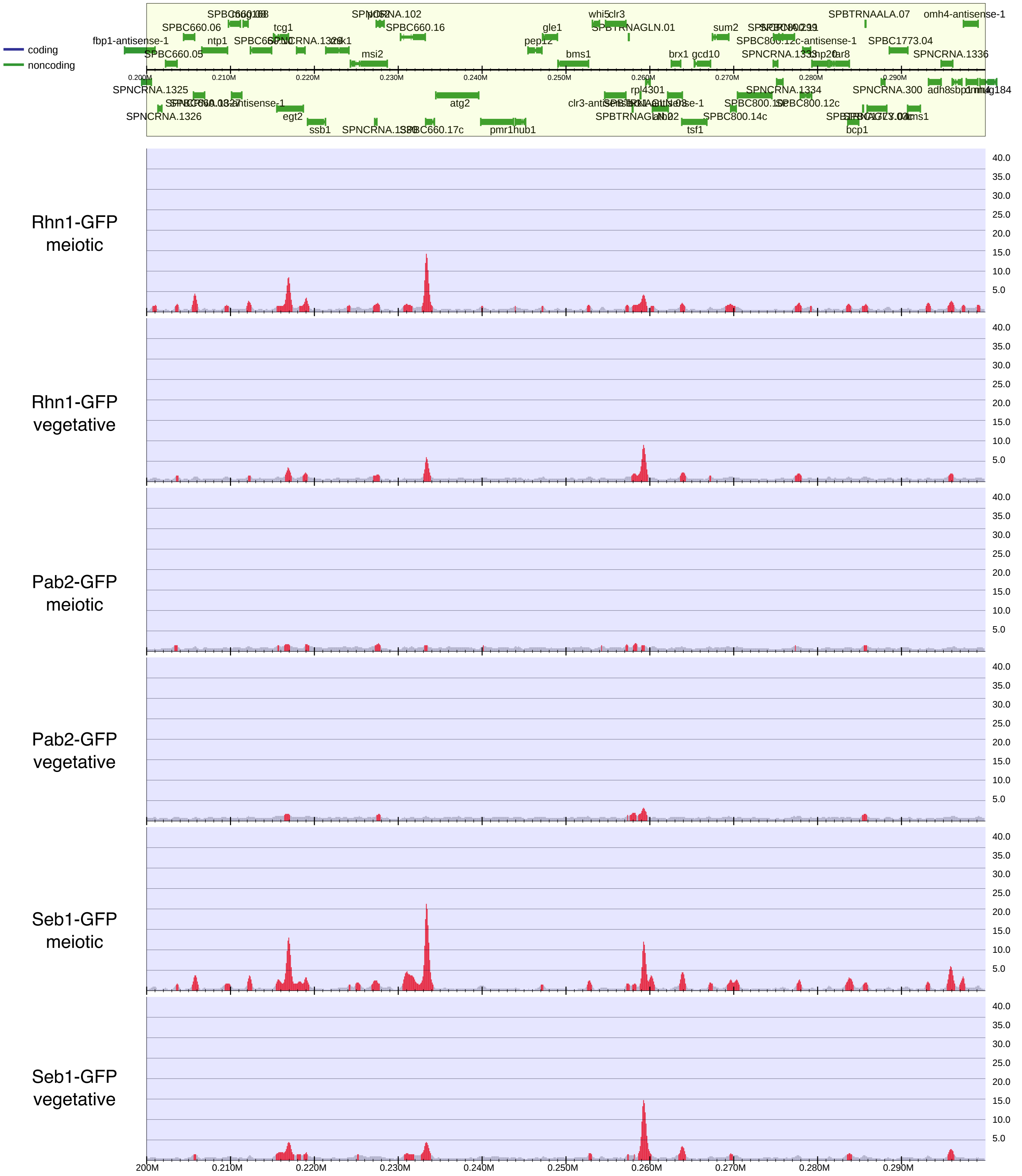
coding
noncoding
Rhn1-GFP meiotic
Rhn1-GFP vegetative
Pab2-GFP meiotic
Pab2-GFP vegetative
Seb1-GFP meiotic
Seb1-GFP vegetative
40.0
35.0
30.0
25.0
20.0
15.0
10.0
5.0
200M
0.210M
0.220M
0.230M
0.240M
0.250M
0.260M
0.270M
0.280M
0.290M

II_1_4

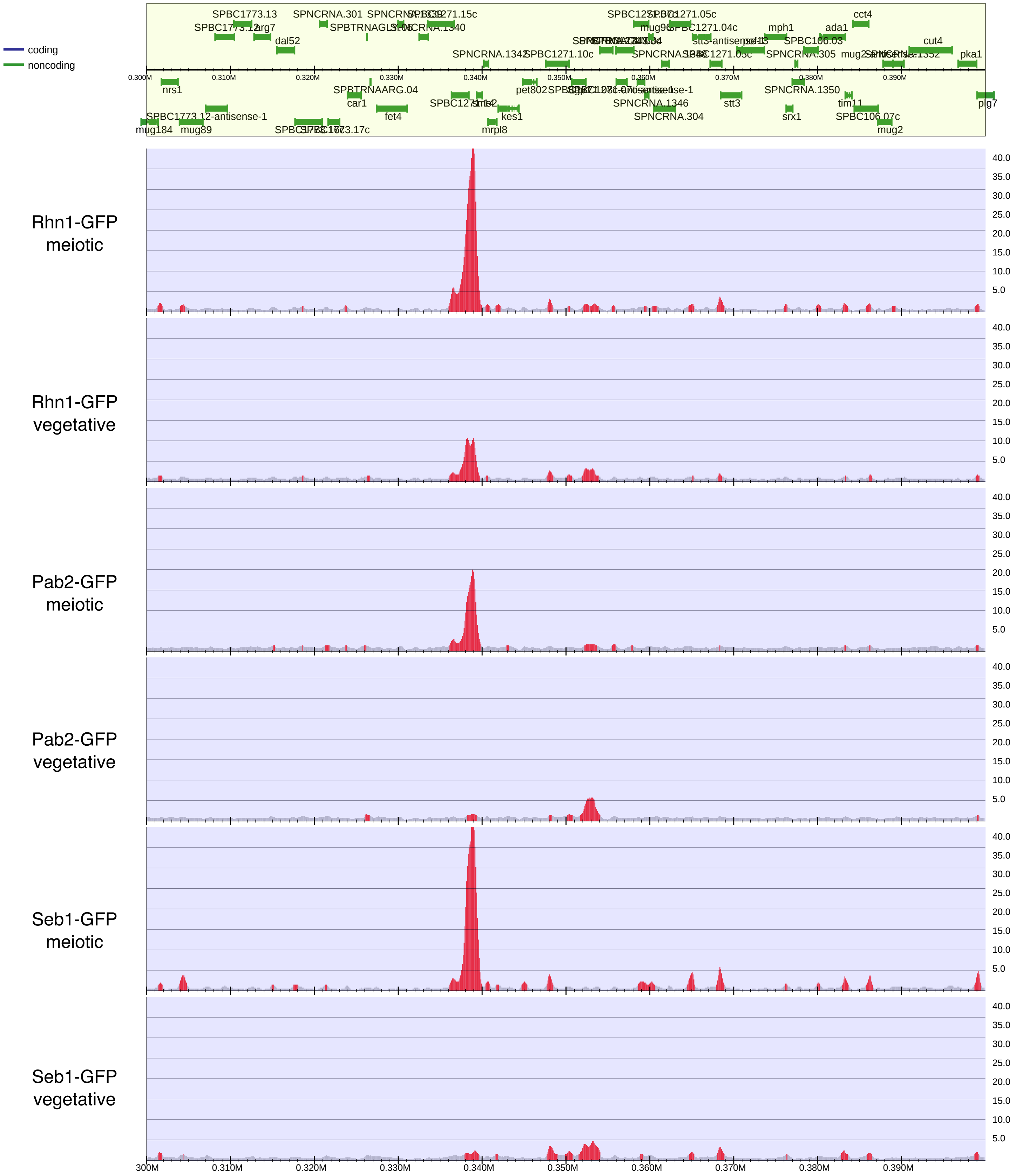
coding
noncoding
Rhn1-GFP meiotic
Rhn1-GFP vegetative
Pab2-GFP meiotic
Pab2-GFP vegetative
Seb1-GFP meiotic
Seb1-GFP vegetative
300M
0.310M
0.320M
0.330M
0.340M
0.350M
0.360M
0.370M
0.380M
0.390M
40.0
35.0
30.0
25.0
20.0
15.0
10.0
5.0

II_1_5

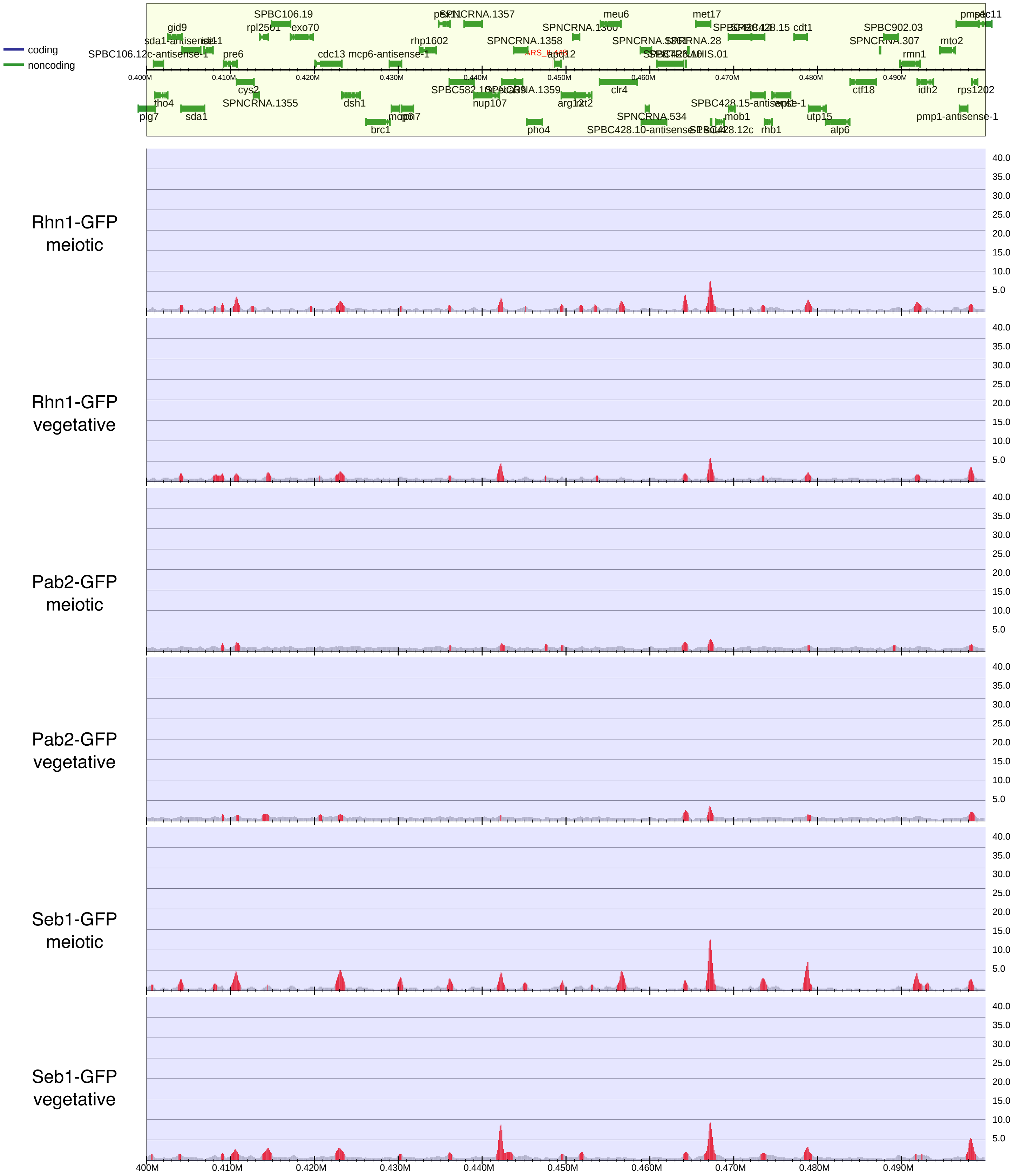
coding
noncoding
gid9
rpl2501
SPBC106.19
exo70
sda1-antisense-1
pre6
SPBC106.12c-antisense-1
cdc13
mcp6-antisense-1
rhp1602
SPNCRNA.1357
SPNCRNA.1358
meu6
SPNCRNA.1360
met17
cdt1
SPBC902.03
SPNCRNA.307
mto2
rmn1
0.400M
0.410M
0.420M
0.430M
0.440M
0.450M
0.460M
0.470M
0.480M
0.490M
cys2
tho4
plg7
sda1
SPNCRNA.1355
dsh1
brc1
nup107
pho4
clr4
SPNCRNA.534
mob1
rhb1
utp15
alp6
ctf18
idh2
rps1202
pmp1-antisense-1
Rhn1-GFP
meiotic
Rhn1-GFP
vegetative
Pab2-GFP
meiotic
Pab2-GFP
vegetative
Seb1-GFP
meiotic
Seb1-GFP
vegetative
40.0
35.0
30.0
25.0
20.0
15.0
10.0
5.0
400M
410M
420M
430M
440M
450M
460M
470M
480M
490M

II_1_6

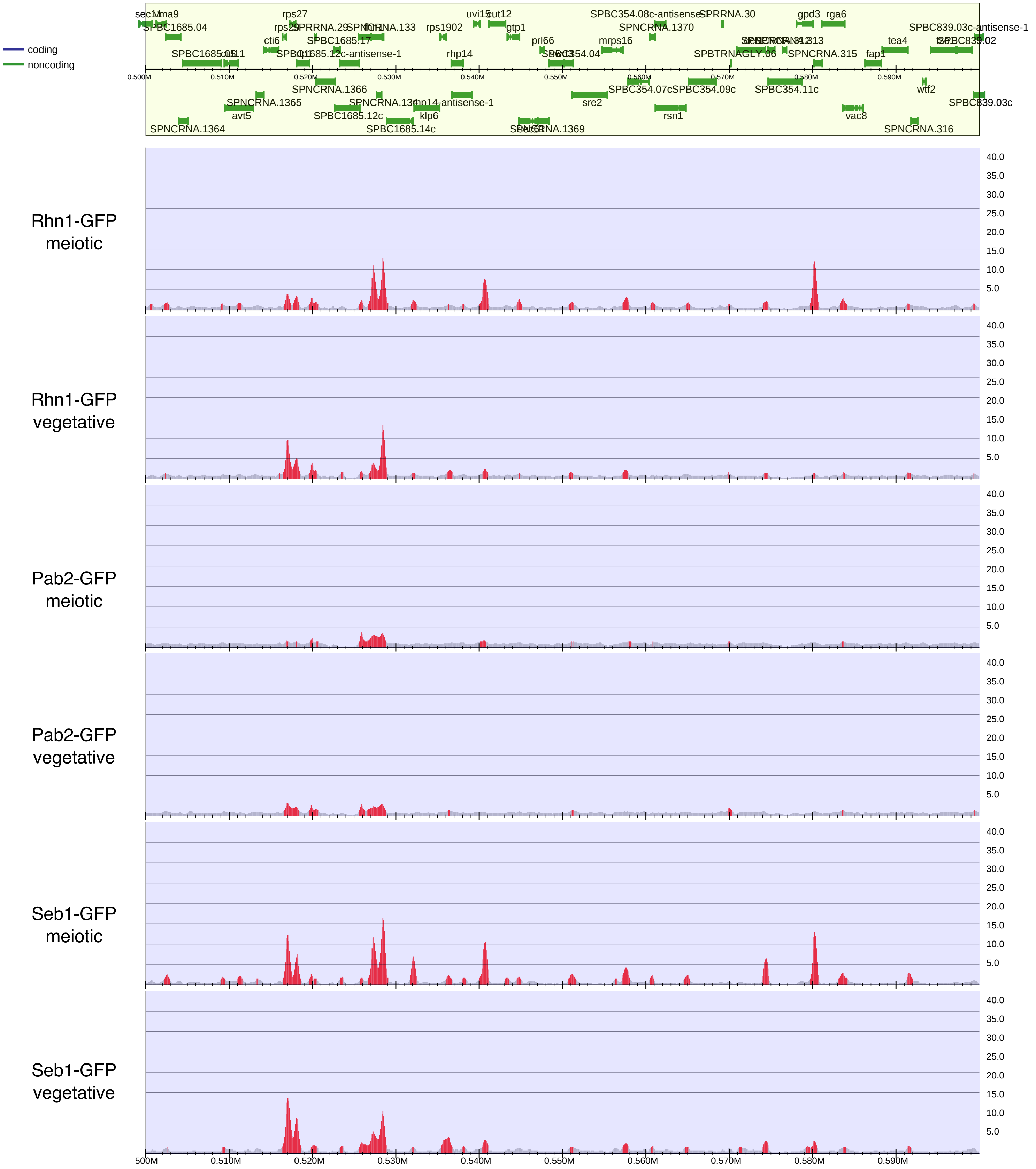
coding
noncoding
rps27
SPBC1685.04
SPBC1685.17
SPBC1685.12c-antisense-1
rps1902
rhp14
uvi15
cut12
gtp1
prl66
SPBC354.08c-antisense-1
SPNCRNA.1370
mrps16
SPBC354.04
gpd3
rga6
SPBC839.03c-antisense-1
SPBC839.02
tea4
fap1
SPNCRNA.315
SPBTRNAGLY.06
SPNCRNA.1366
SPNCRNA.1365
avt5
SPBC1685.12c
klp6
SPBC1685.14c
rhp14-antisense-1
SPNCRNA.1364
sre2
SPBC354.07c
SPBC354.09c
SPBC354.11c
rsn1
vac8
wtf2
SPBC839.03c
SPNCRNA.316
Rhn1-GFP meiotic
Rhn1-GFP vegetative
Pab2-GFP meiotic
Pab2-GFP vegetative
Seb1-GFP meiotic
Seb1-GFP vegetative
40.0
35.0
30.0
25.0
20.0
15.0
10.0
5.0
500M
0.510M
0.520M
0.530M
0.540M
0.550M
0.560M
0.570M
0.580M
0.590M

II_1_7

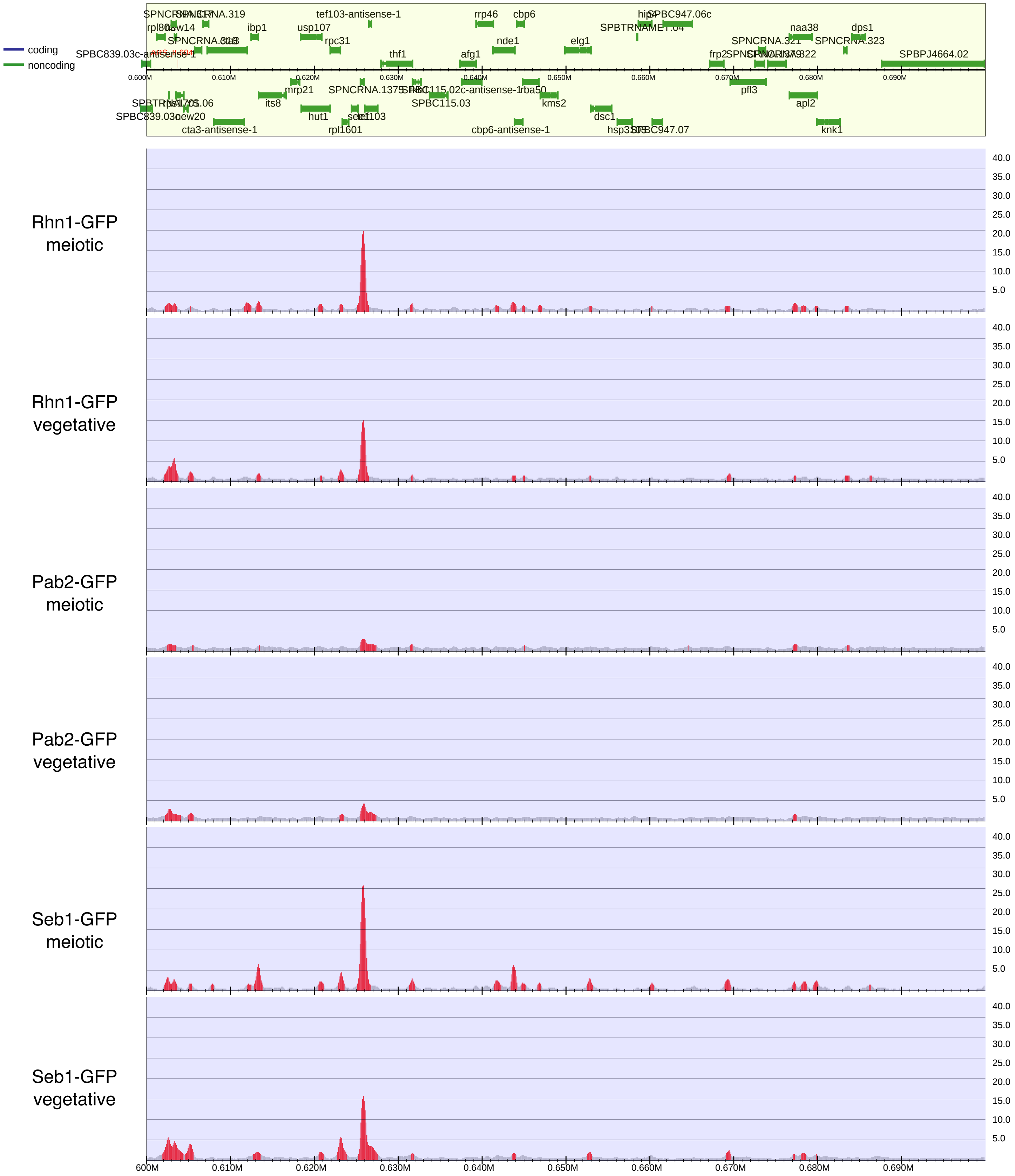

coding
noncoding
tef103-antisense-1
rrp46
cbp6
ibp1
usp107
naa38
dps1
rpc31
nde1
elg1
thf1
afg1
SPBPJ4664.02
mrp21
its8
hut1
rpl1601
cta3-antisense-1
SPBC115.03
kms2
dsc1
cbp6-antisense-1
pfl3
apl2
knk1
Rhn1-GFP meiotic
Rhn1-GFP vegetative
Pab2-GFP meiotic
Pab2-GFP vegetative
Seb1-GFP meiotic
Seb1-GFP vegetative
40.0
35.0
30.0
25.0
20.0
15.0
10.0
5.0
600M
0.610M
0.620M
0.630M
0.640M
0.650M
0.660M
0.670M
0.680M
0.690M

II_1_8

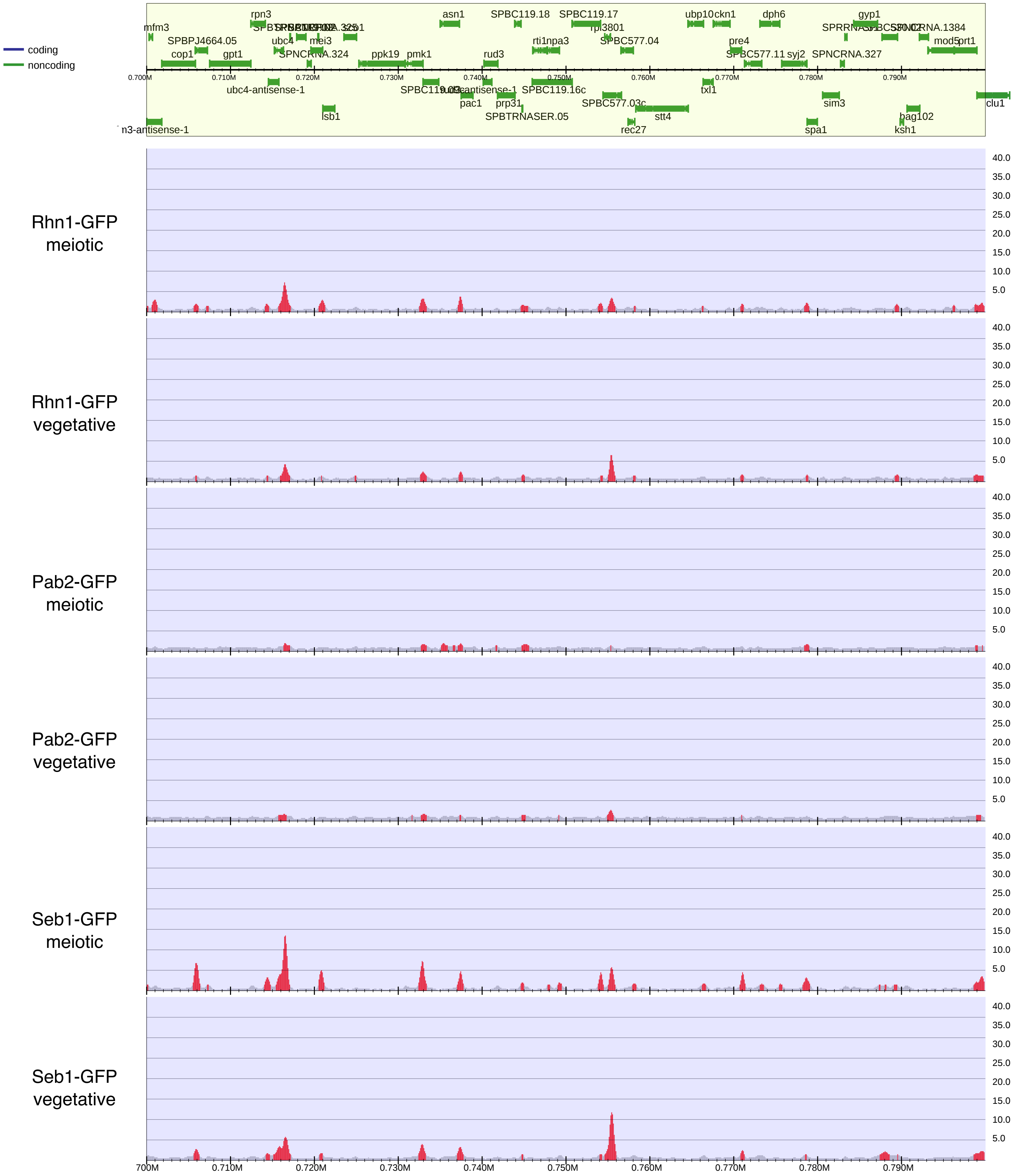
coding
noncoding
Rhn1-GFP meiotic
Rhn1-GFP vegetative
Pab2-GFP meiotic
Pab2-GFP vegetative
Seb1-GFP meiotic
Seb1-GFP vegetative
700M
0.710M
0.720M
0.730M
0.740M
0.750M
0.760M
0.770M
0.780M
0.790M
40.0
35.0
30.0
25.0
20.0
15.0
10.0
5.0

II_1_9

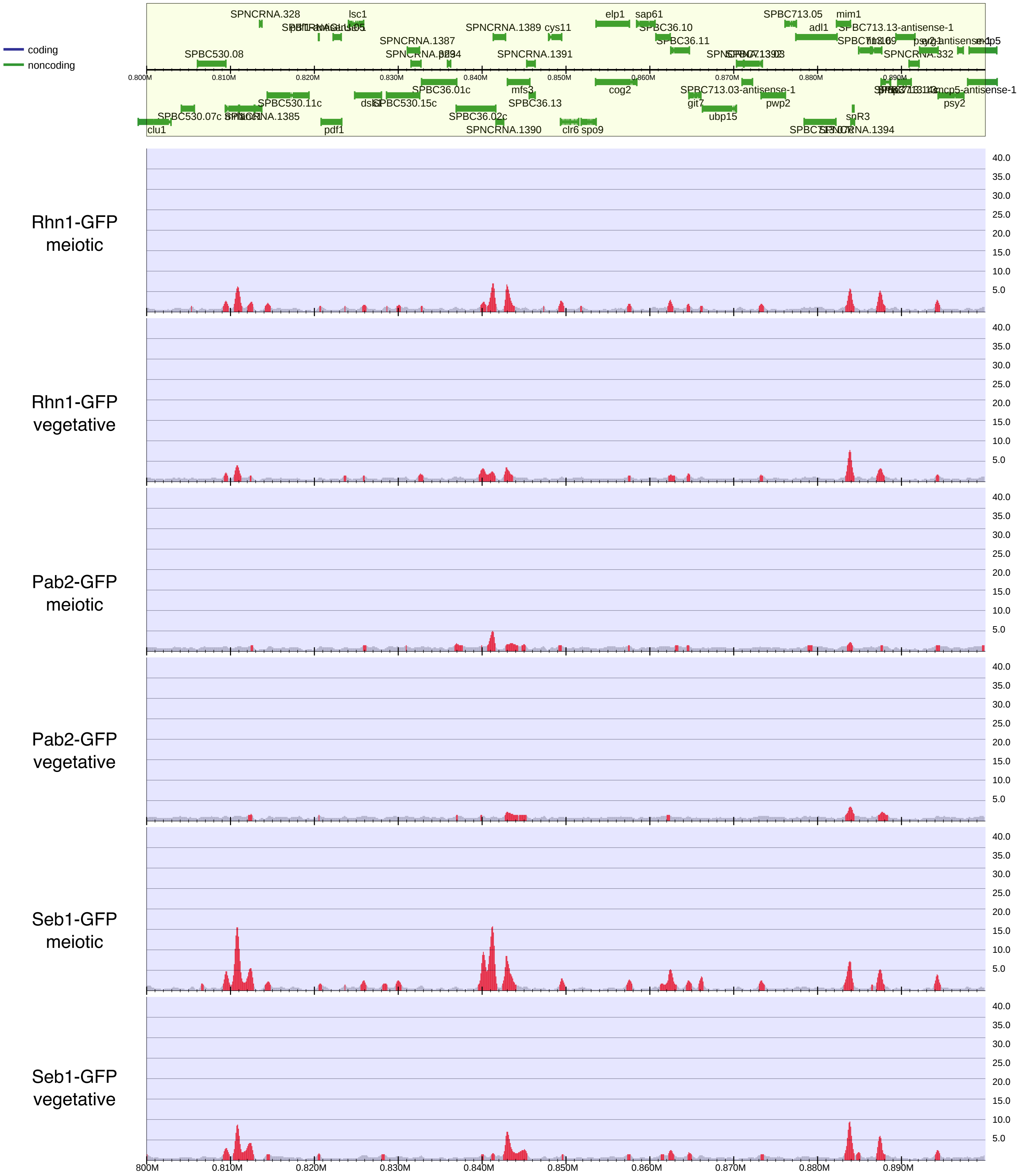
coding
noncoding
SPNCRNA.328
lsc1
SPNCRNA.1387
SPNCRNA.1389
cys11
elp1
sap61
SPBC36.10
SPBC36.11
SPBC713.05
mim1
adl1
SPBC713.13-antisense-1
SPNCRNA.332
SPBC530.08
SPNCRNA.1391
SPBC36.01c
mfs3
SPBC36.13
cog2
SPBC713.03-antisense-1
git7
pwp2
psy2
SPBC530.11c
SPBC530.15c
SPBC530.07c
SPBC36.02c
ubp15
snR3
clu1
pdf1
SPNCRNA.1390
clr6
spo9
SPNCRNA.1394
Rhn1-GFP meiotic
Rhn1-GFP vegetative
Pab2-GFP meiotic
Pab2-GFP vegetative
Seb1-GFP meiotic
Seb1-GFP vegetative
40.0
35.0
30.0
25.0
20.0
15.0
10.0
5.0
800M
0.810M
0.820M
0.830M
0.840M
0.850M
0.860M
0.870M
0.880M
0.890M

II_1_10

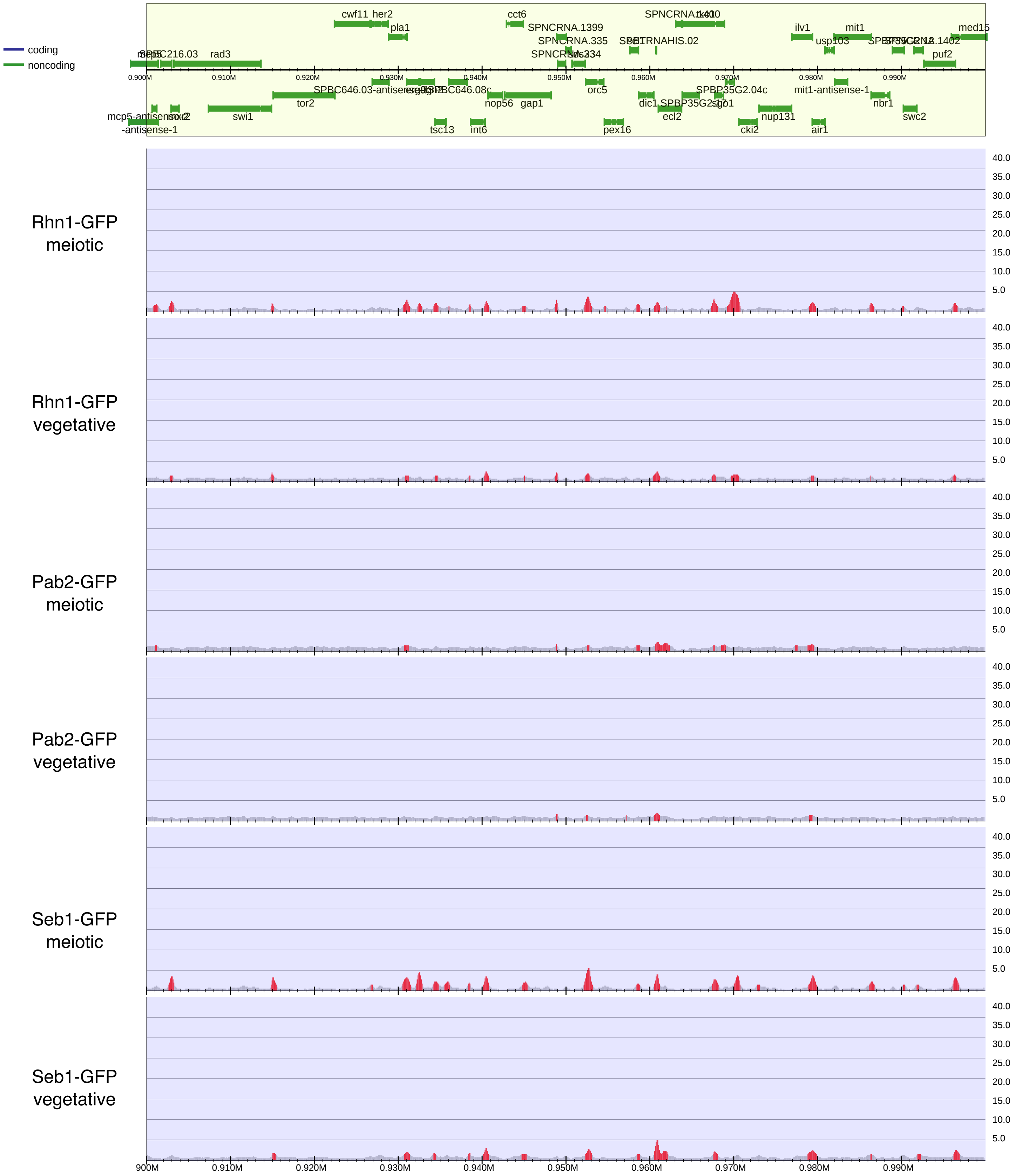
coding
noncoding
Rhn1-GFP meiotic
Rhn1-GFP vegetative
Pab2-GFP meiotic
Pab2-GFP vegetative
Seb1-GFP meiotic
Seb1-GFP vegetative
900M
0.910M
0.920M
0.930M
0.940M
0.950M
0.960M
0.970M
0.980M
0.990M

II_1_11

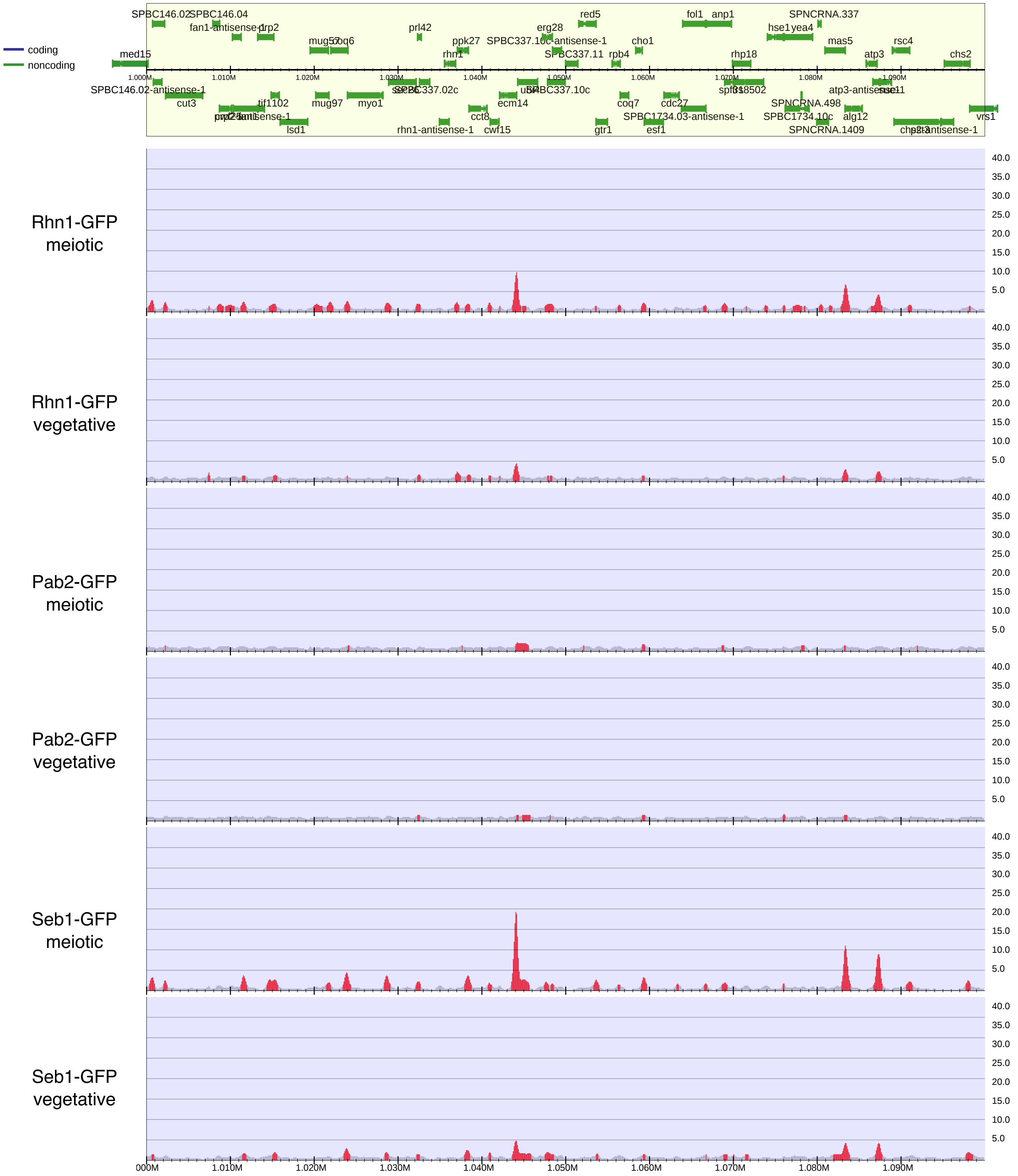

coding
noncoding
SPBC146.02
SPBC146.04
fan1-antisense-1
trp2
prl42
red5
fol1
anp1
SPNCRNA.337
hse1
yea4
mug57
coq6
ppk27
SPBC337.10c-antisense-1
erg28
cho1
mas5
rsc4
med15
rhn1
SPBC337.11
rpb4
rhp18
atp3
chs2
SPBC146.02-antisense-1
cut3
tif1102
mug97
myo1
ecm14
SPBC337.10c
coq7
cdc27
SPNCRNA.498
atp3-antisense-1
lsd1
cct8
cwf15
rhn1-antisense-1
gtr1
esf1
SPBC1734.03-antisense-1
SPBC1734.10c
alg12
SPNCRNA.1409
vrs1
Rhn1-GFP meiotic
Rhn1-GFP vegetative
Pab2-GFP meiotic
Pab2-GFP vegetative
Seb1-GFP meiotic
Seb1-GFP vegetative
40.0
35.0
30.0
25.0
20.0
15.0
10.0
5.0
000M
1.010M
1.020M
1.030M
1.040M
1.050M
1.060M
1.070M
1.080M
1.090M

II_1_12

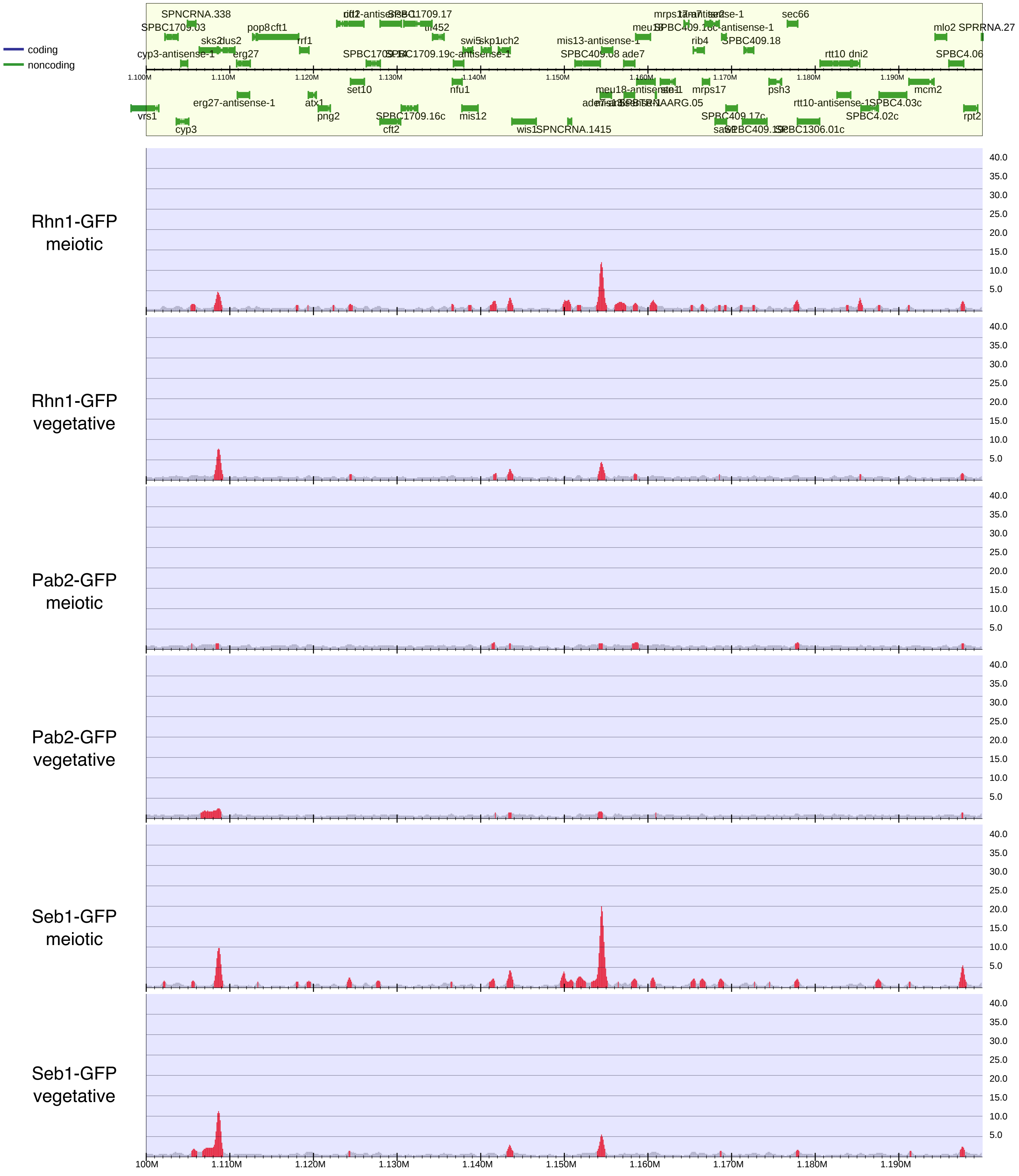
coding
noncoding
Rhn1-GFP meiotic
Rhn1-GFP vegetative
Pab2-GFP meiotic
Pab2-GFP vegetative
Seb1-GFP meiotic
Seb1-GFP vegetative
100M
1.110M
1.120M
1.130M
1.140M
1.150M
1.160M
1.170M
1.180M
1.190M

II_1_13

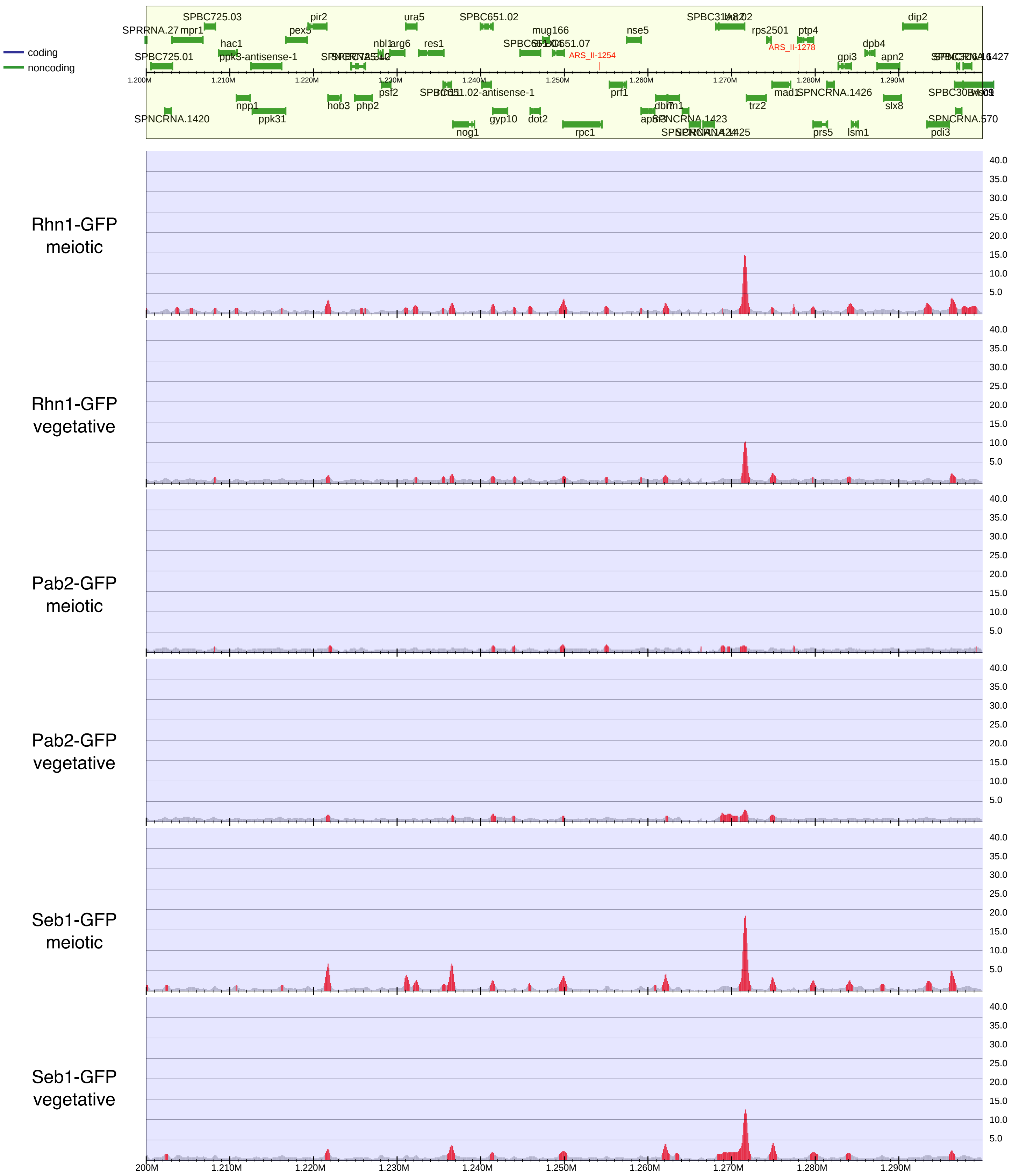
coding
noncoding
SPBC725.03
pir2
ura5
SPBC651.02
dip2
SPRRNA.27
mpr1
pex5
mug166
nse5
rps2501
ptp4
hac1
nbl1
arg6
res1
dpb4
SPBC725.01
ppk3-antisense-1
ARS_II-1254
ARS_II-1278
gpi3
apn2
psf2
prf1
mad1
SPNCRNA.1426
npp1
hob3
php2
trz2
slx8
SPNCRNA.1420
ppk31
gyp10
dot2
SPNCRNA.1423
SPNCRNA.570
nog1
rpc1
prs5
lsm1
pdi3
Rhn1-GFP
meiotic
Rhn1-GFP
vegetative
Pab2-GFP
meiotic
Pab2-GFP
vegetative
Seb1-GFP
meiotic
Seb1-GFP
vegetative
40.0
35.0
30.0
25.0
20.0
15.0
10.0
5.0
200M
1.210M
1.220M
1.230M
1.240M
1.250M
1.260M
1.270M
1.280M
1.290M

II_1_14

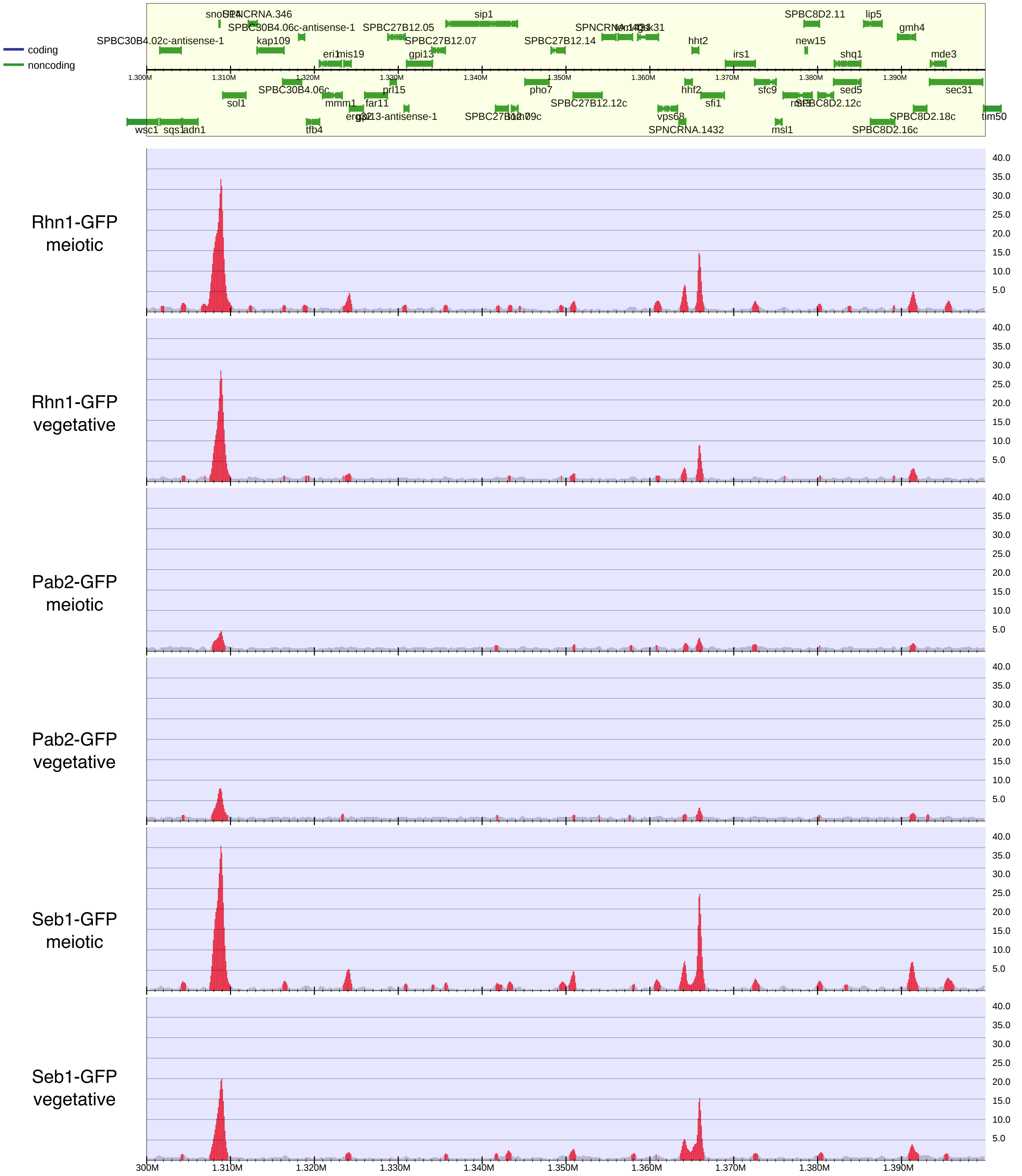

coding
noncoding
Rhn1-GFP meiotic
Rhn1-GFP vegetative
Pab2-GFP meiotic
Pab2-GFP vegetative
Seb1-GFP meiotic
Seb1-GFP vegetative
40.0
35.0
30.0
25.0
20.0
15.0
10.0
5.0
300M
1.310M
1.320M
1.330M
1.340M
1.350M
1.360M
1.370M
1.380M
1.390M
sip1
SPBC8D2.11
lip5
gmh4
kap109
hht2
new15
irs1
shq1
mde3
sol1
pho7
hhf2
sfc9
sed5
sec31
SPBC27B12.12c
sfi1
SPBC8D2.18c
tim50
wsc1
tfb4
SPNCRNA.1432
msl1
SPBC8D2.16c

II_1_15

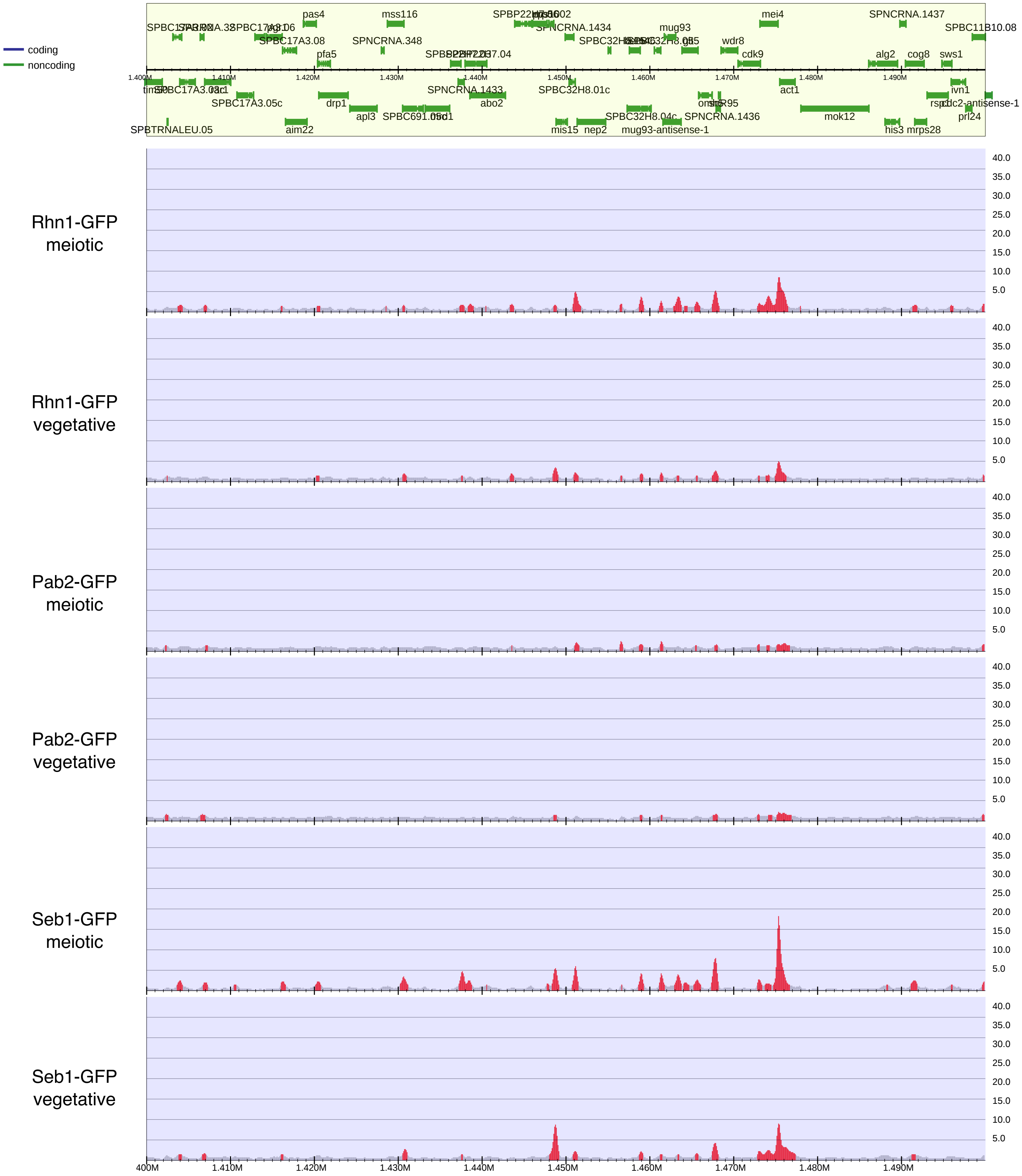
coding
noncoding
pas4
mss116
mei4
SPNCRNA.1437
SPNCRNA.1434
mug93
SPBC11B10.08
SPBC17A3.08
SPNCRNA.348
wdr8
pfa5
cdk9
alg2
cog8
sws1
1.400M
1.410M
1.420M
1.430M
1.440M
1.450M
1.460M
1.470M
1.480M
1.490M
SPNCRNA.1433
SPBC32H8.01c
act1
ivn1
SPBC17A3.05c
drp1
abo2
apl3
mok12
prl24
SPBTRNALEU.05
aim22
mis15
nep2
mug93-antisense-1
SPNCRNA.1436
his3
mrps28
Rhn1-GFP meiotic
Rhn1-GFP vegetative
Pab2-GFP meiotic
Pab2-GFP vegetative
Seb1-GFP meiotic
Seb1-GFP vegetative
40.0
35.0
30.0
25.0
20.0
15.0
10.0
5.0
400M
1.410M
1.420M
1.430M
1.440M
1.450M
1.460M
1.470M
1.480M
1.490M

II_1_16

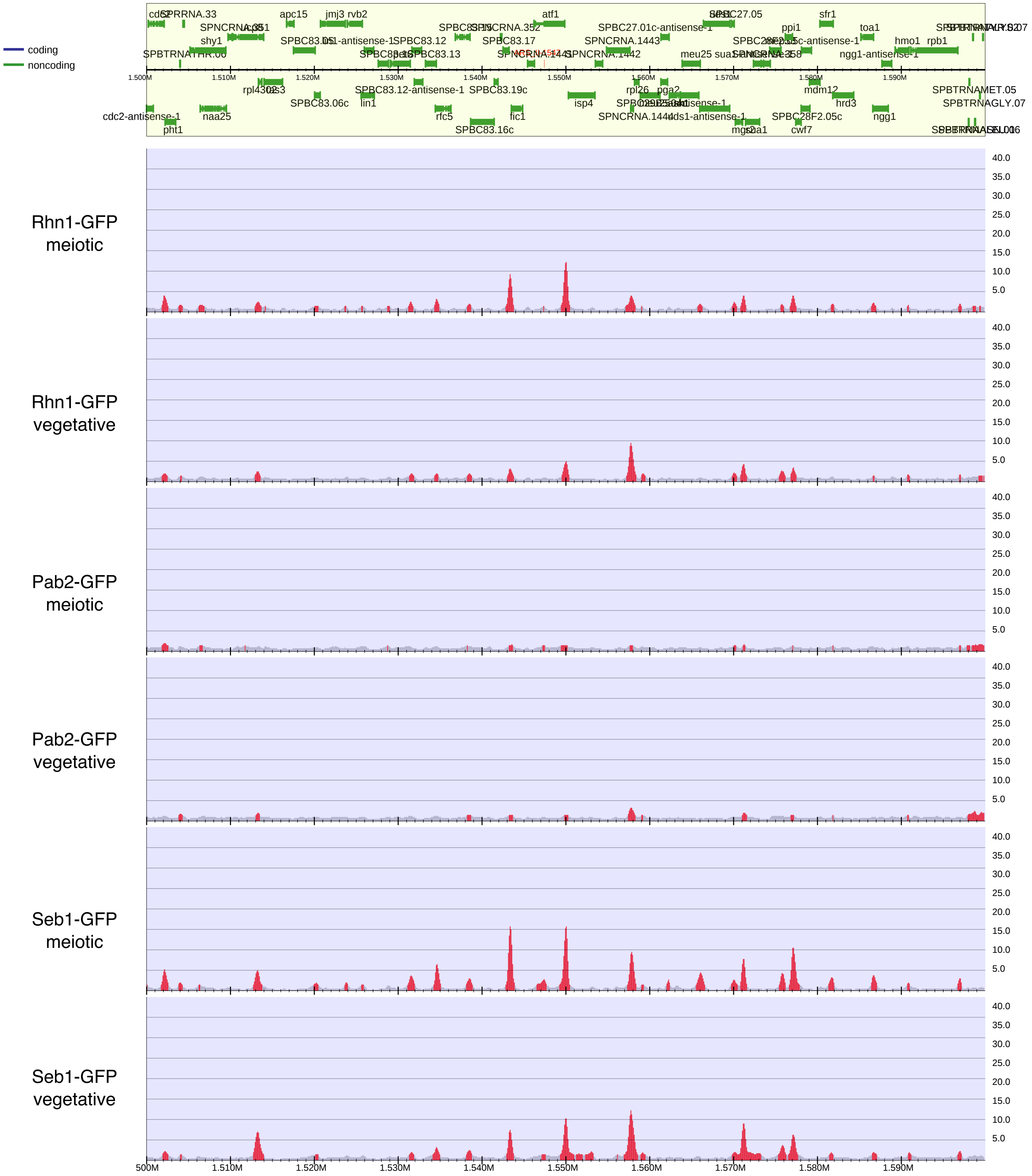
coding
noncoding
Rhn1-GFP meiotic
Rhn1-GFP vegetative
Pab2-GFP meiotic
Pab2-GFP vegetative
Seb1-GFP meiotic
Seb1-GFP vegetative
500M
1.510M
1.520M
1.530M
1.540M
1.550M
1.560M
1.570M
1.580M
1.590M

II_1_17

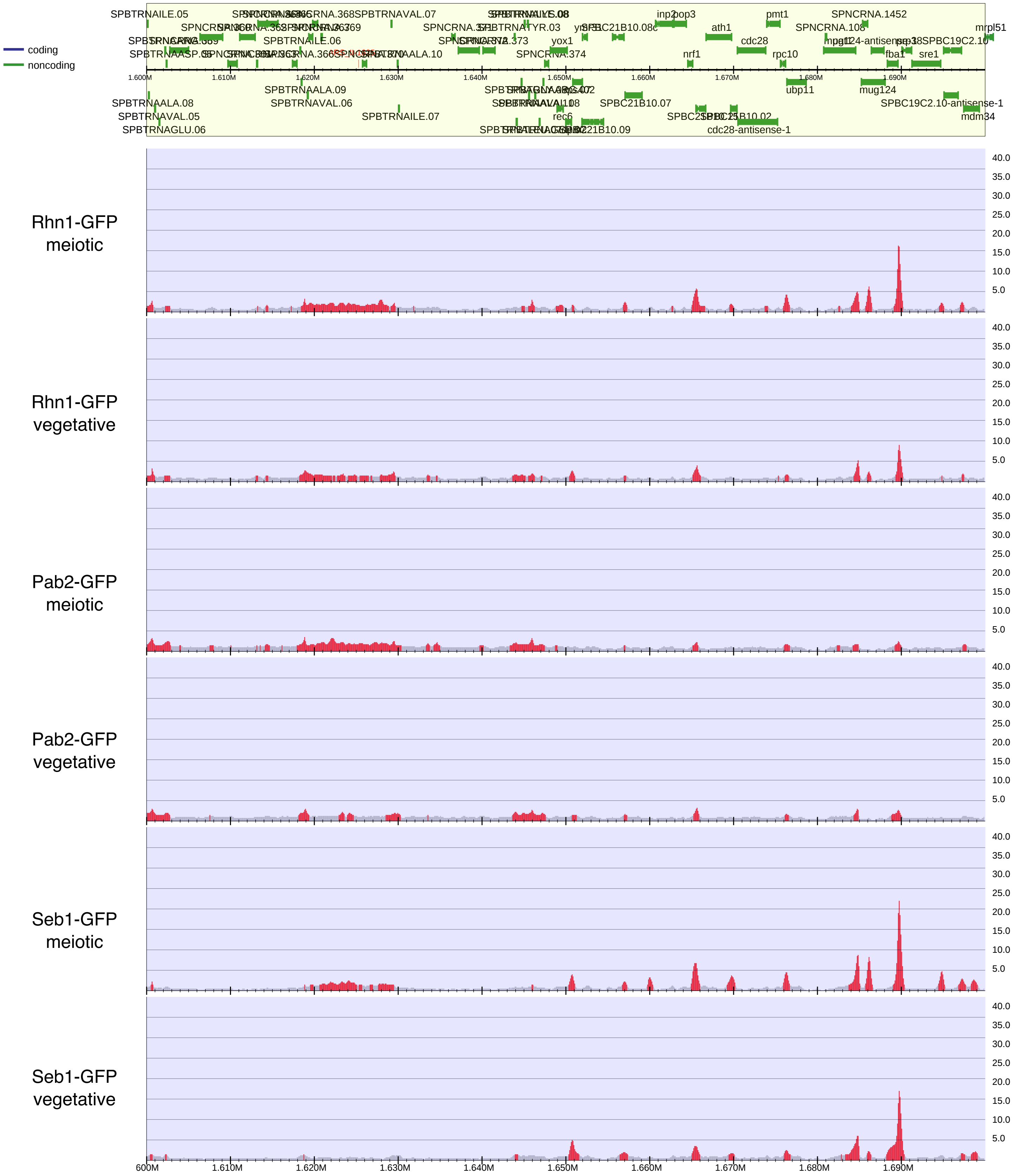

coding
noncoding
SPBTRNAILE.05
SPBTRNAVAL.07
inp2
pop3
pmt1
SPNCRNA.1452
ath1
SPNCRNA.108
mrpl51
SPBTRNAILE.06
yox1
cdc28
SPNCRNA.374
nrf1
rpc10
fba1
sre1
SPBTRNAALA.09
ubp11
mug124
SPBTRNAALA.08
SPBTRNAVAL.06
SPBC21B10.07
SPBC19C2.10-antisense-1
SPBTRNAVAL.05
SPBTRNAILE.07
rec6
mdm34
SPBTRNAGLU.06
cdc28-antisense-1
Rhn1-GFP
meiotic
Rhn1-GFP
vegetative
Pab2-GFP
meiotic
Pab2-GFP
vegetative
Seb1-GFP
meiotic
Seb1-GFP
vegetative
40.0
35.0
30.0
25.0
20.0
15.0
10.0
5.0
600M
1.610M
1.620M
1.630M
1.640M
1.650M
1.660M
1.670M
1.680M
1.690M

II_1_18

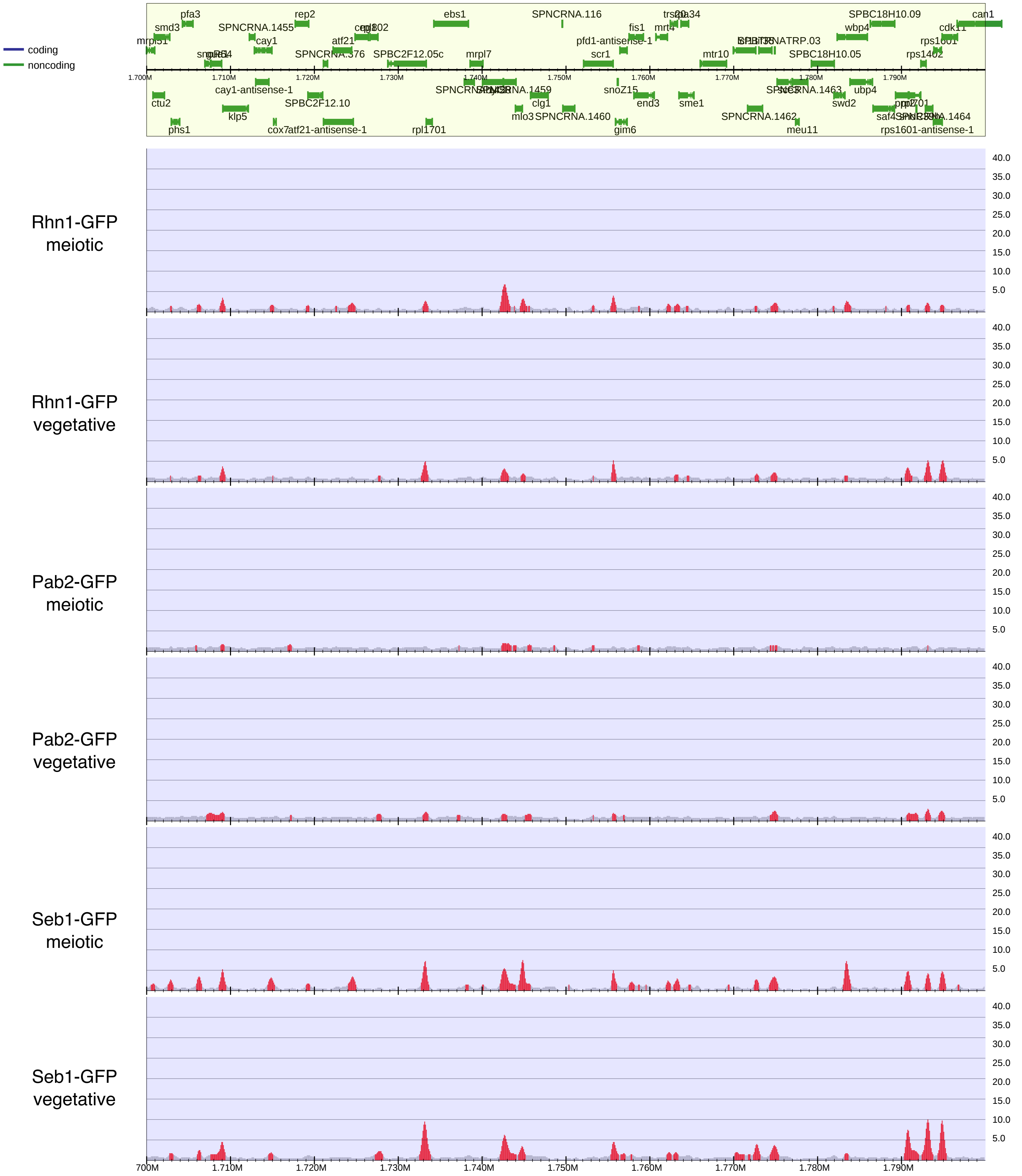
coding
noncoding
pfa3
smd3
mrpl51
SPNCRNA.1455
cay1
rep2
atf21
SPNCRNA.376
SPBC2F12.05c
ebs1
mrpl7
SPNCRNA.116
pfd1-antisense-1
scr1
fis1
mrt4
mtr10
SPBC18H10.09
wbp4
SPBC18H10.05
can1
cdk11
rps1601
rps1402
cay1-antisense-1
ctu2
klp5
phs1
SPBC2F12.10
cox7
atf21-antisense-1
rpl1701
SPNCRNA.1459
clg1
mlo3
SPNCRNA.1460
snoZ15
end3
gim6
sme1
SPNCRNA.1462
SPNCRNA.1463
ubp4
swd2
meu11
SPNCRNA.1464
rps1601-antisense-1
1.700M
1.710M
1.720M
1.730M
1.740M
1.750M
1.760M
1.770M
1.780M
1.790M
Rhn1-GFP meiotic
Rhn1-GFP vegetative
Pab2-GFP meiotic
Pab2-GFP vegetative
Seb1-GFP meiotic
Seb1-GFP vegetative
40.0
35.0
30.0
25.0
20.0
15.0
10.0
5.0
700M
1.710M
1.720M
1.730M
1.740M
1.750M
1.760M
1.770M
1.780M
1.790M

II_1_19

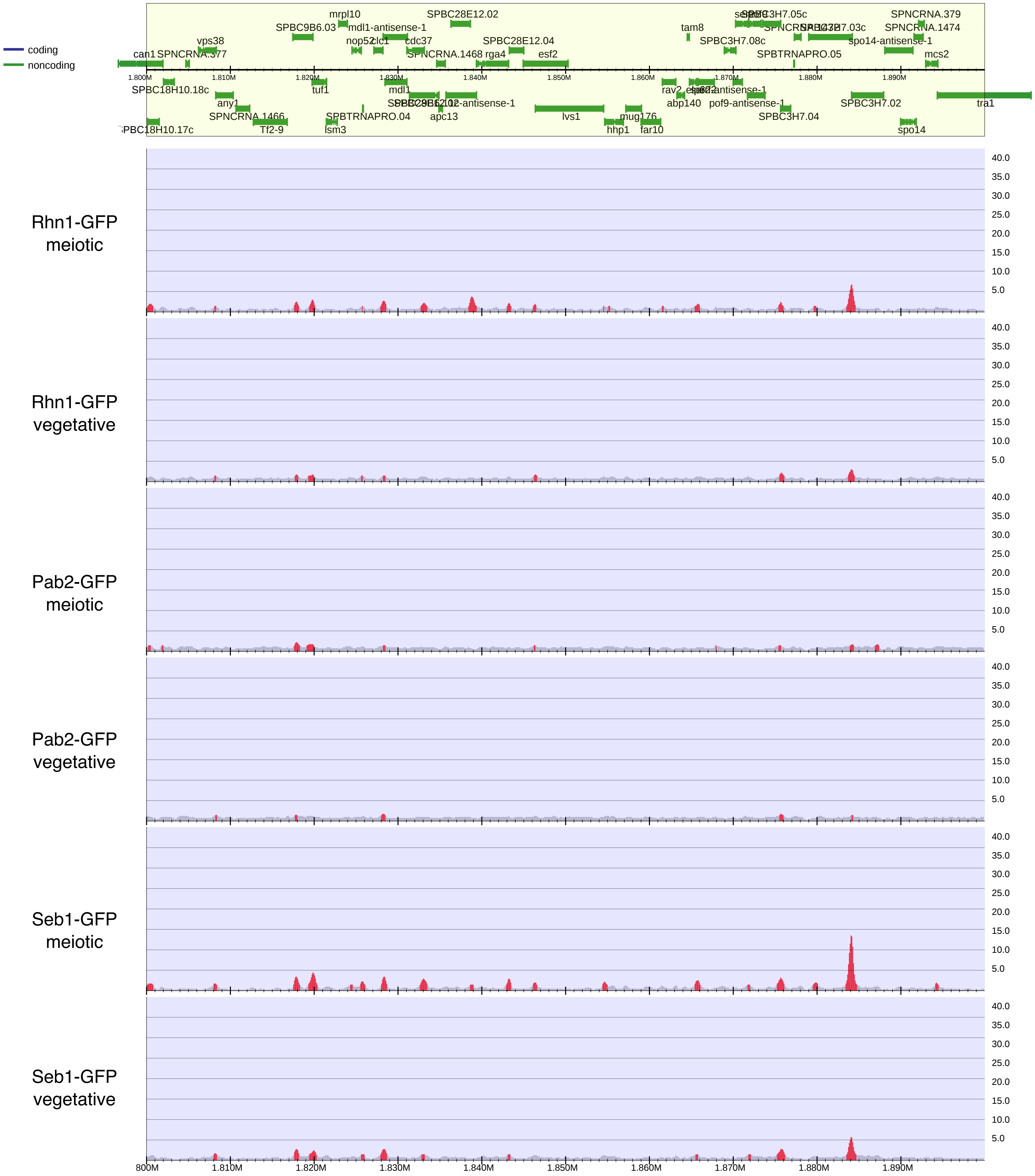
coding
noncoding
mrpl10
SPBC28E12.02
SPBC3H7.05c
SPNCRNA.379
SPBC9B6.03
mdl1-antisense-1
tam8
SPNCRNA.1474
vps38
nop52
clc1
cdc37
SPBC28E12.04
SPBC3H7.08c
spo14-antisense-1
can1
SPNCRNA.377
SPNCRNA.1468
rga4
esf2
SPBTRNAPRO.05
mcs2
SPBC18H10.18c
tuf1
mdl1
rav2
abp140
pof9-antisense-1
SPBC3H7.02
tra1
any1
apc13
lvs1
SPNCRNA.1466
SPBTRNAPRO.04
mug176
SPBC3H7.04
Tf2-9
lsm3
hhp1
far10
spo14
Rhn1-GFP meiotic
Rhn1-GFP vegetative
Pab2-GFP meiotic
Pab2-GFP vegetative
Seb1-GFP meiotic
Seb1-GFP vegetative
40.0
35.0
30.0
25.0
20.0
15.0
10.0
5.0
800M
1.810M
1.820M
1.830M
1.840M
1.850M
1.860M
1.870M
1.880M
1.890M

II_1_20

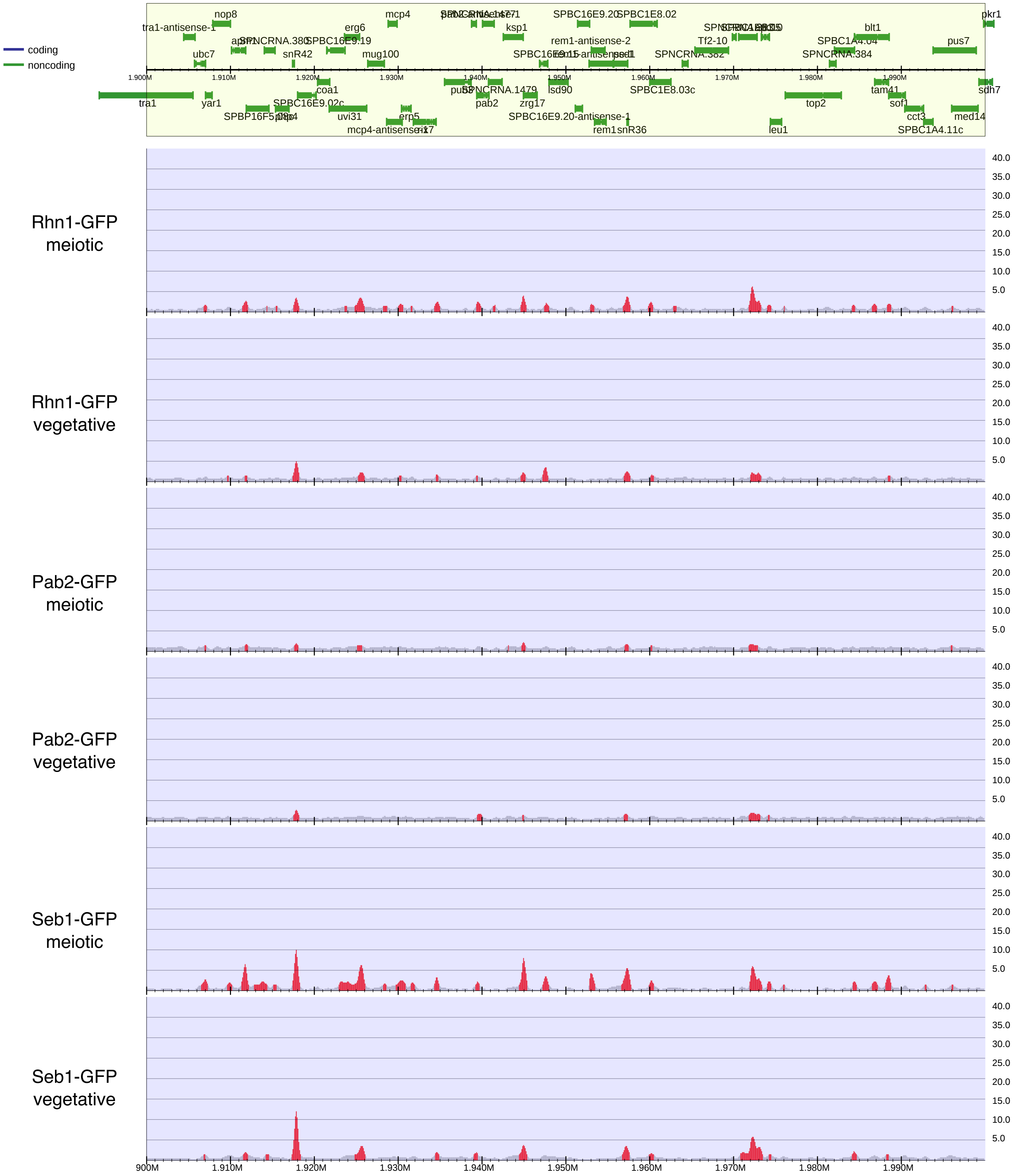

coding
noncoding
Rhn1-GFP meiotic
Rhn1-GFP vegetative
Pab2-GFP meiotic
Pab2-GFP vegetative
Seb1-GFP meiotic
Seb1-GFP vegetative
40.0
35.0
30.0
25.0
20.0
15.0
10.0
5.0
900M
1.910M
1.920M
1.930M
1.940M
1.950M
1.960M
1.970M
1.980M
1.990M

II_1_21

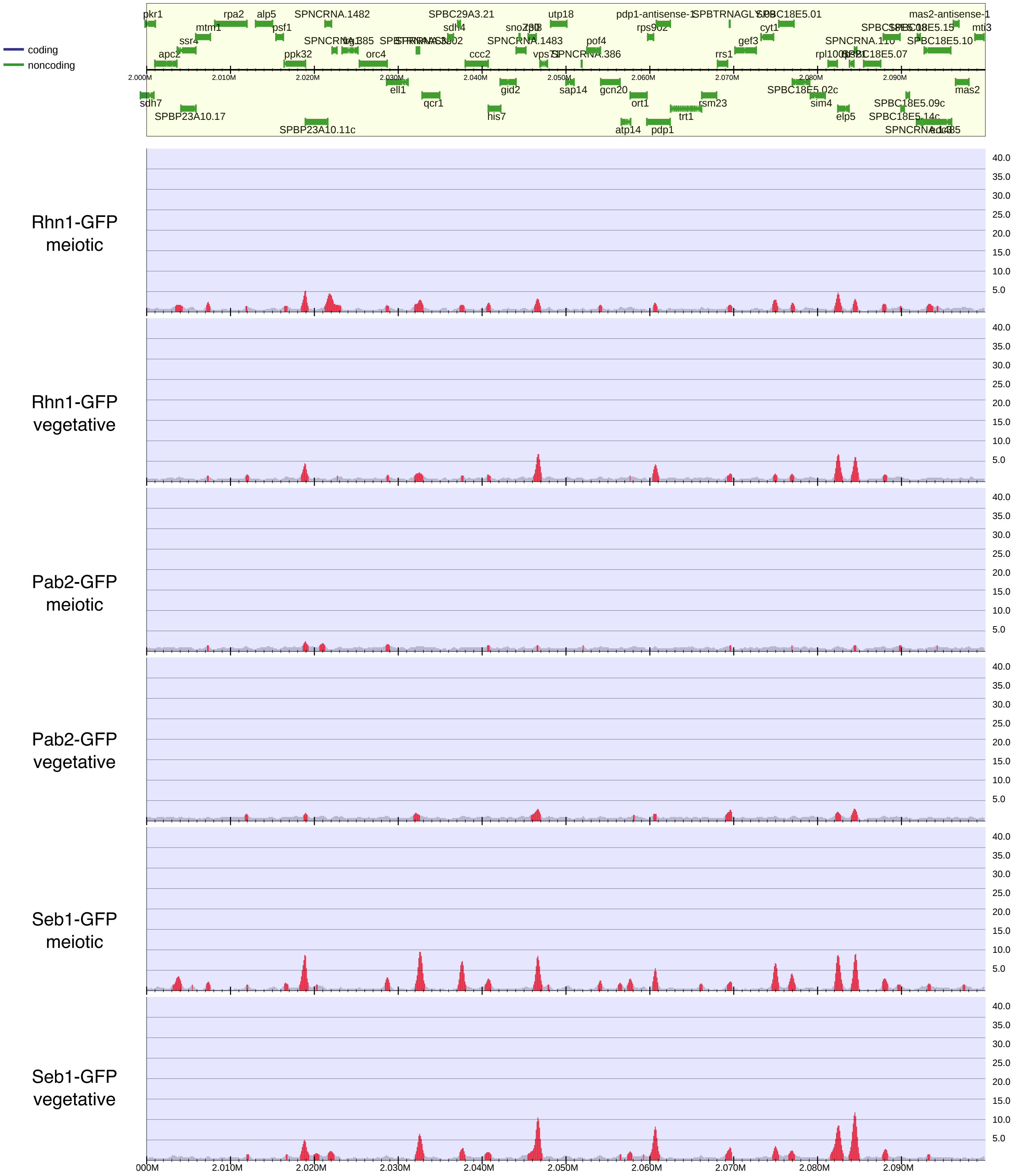
coding
noncoding
Rhn1-GFP meiotic
Rhn1-GFP vegetative
Pab2-GFP meiotic
Pab2-GFP vegetative
Seb1-GFP meiotic
Seb1-GFP vegetative
2.010M
2.020M
2.030M
2.040M
2.050M
2.060M
2.070M
2.080M
2.090M

II_1_22

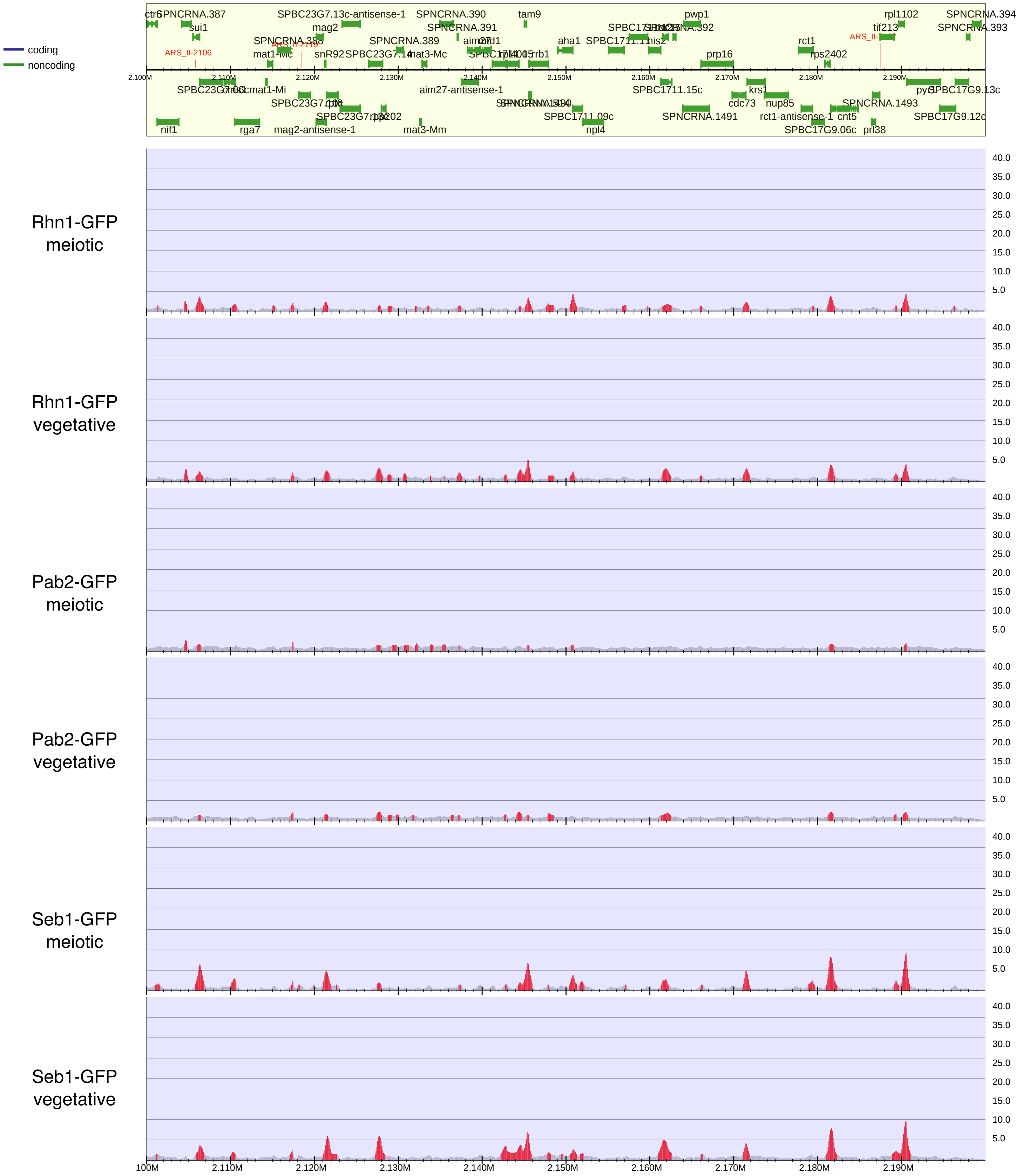
coding
noncoding
Rhn1-GFP meiotic
Rhn1-GFP vegetative
Pab2-GFP meiotic
Pab2-GFP vegetative
Seb1-GFP meiotic
Seb1-GFP vegetative
40.0
35.0
30.0
25.0
20.0
15.0
10.0
5.0
100M
2.110M
2.120M
2.130M
2.140M
2.150M
2.160M
2.170M
2.180M
2.190M

II_1_23

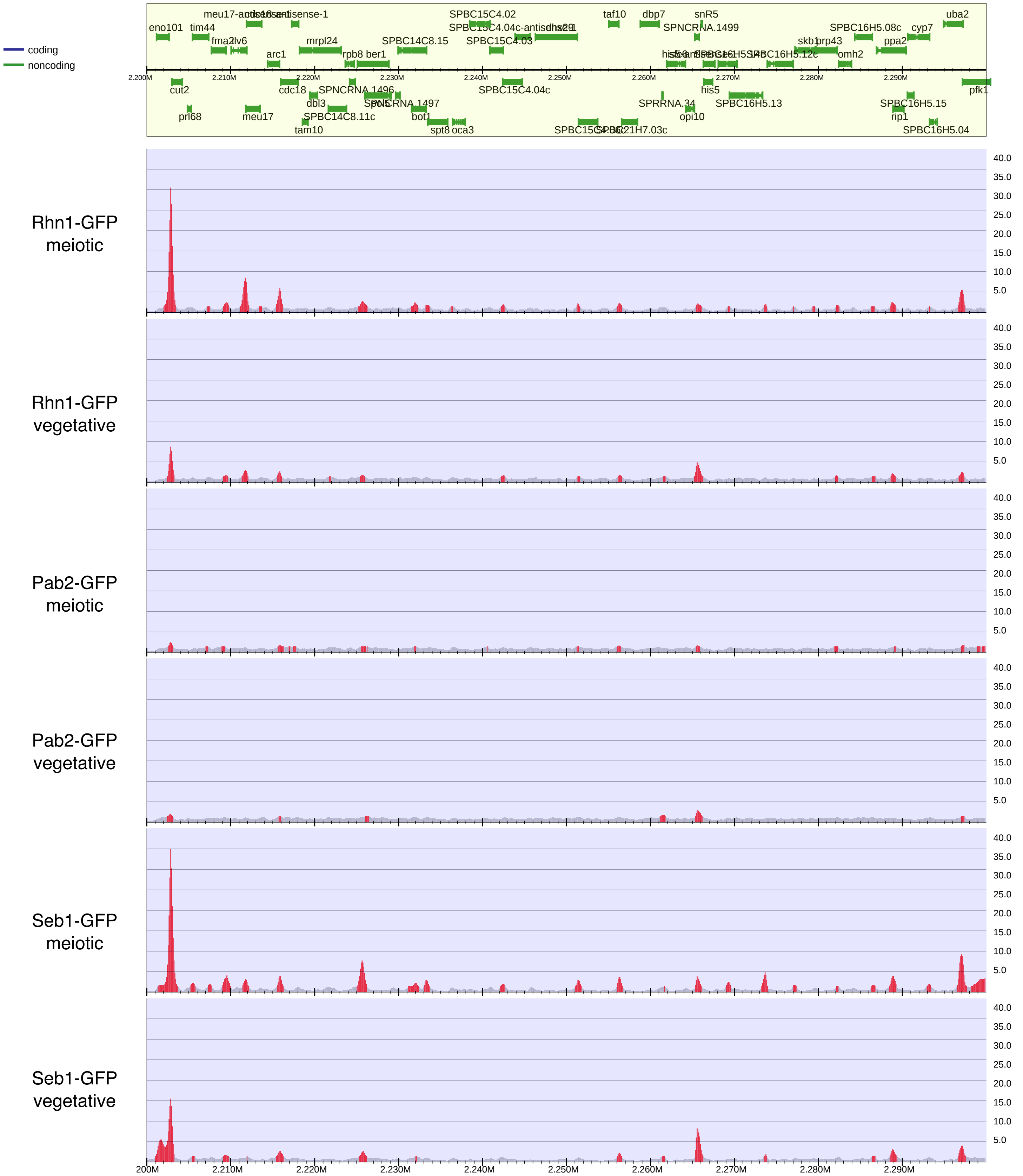
coding
noncoding
eno101
tim44
fma2
lv6
arc1
mrpl24
rpb8 ber1
SPBC14C8.15
SPBC15C4.02
SPBC15C4.03
taf10
dbp7
snR5
SPNCRNA.1499
skb1
prp43
SPBC16H5.08c
cyp7
ppa2
omh2
uba2
cut2
prl68
meu17
cdc18
dbl3
SPNCRNA.1496
SPBC14C8.11c
tam10
bot1
spt8
oca3
SPBC15C4.04c
SPRRNA.34
opi10
his5
SPBC16H5.13
SPBC16H5.15
rip1
SPBC16H5.04
pfk1
2.200M
2.210M
2.220M
2.230M
2.240M
2.250M
2.260M
2.270M
2.280M
2.290M
Rhn1-GFP meiotic
Rhn1-GFP vegetative
Pab2-GFP meiotic
Pab2-GFP vegetative
Seb1-GFP meiotic
Seb1-GFP vegetative
40.0
35.0
30.0
25.0
20.0
15.0
10.0
5.0
200M
2.210M
2.220M
2.230M
2.240M
2.250M
2.260M
2.270M
2.280M
2.290M

II_1_24

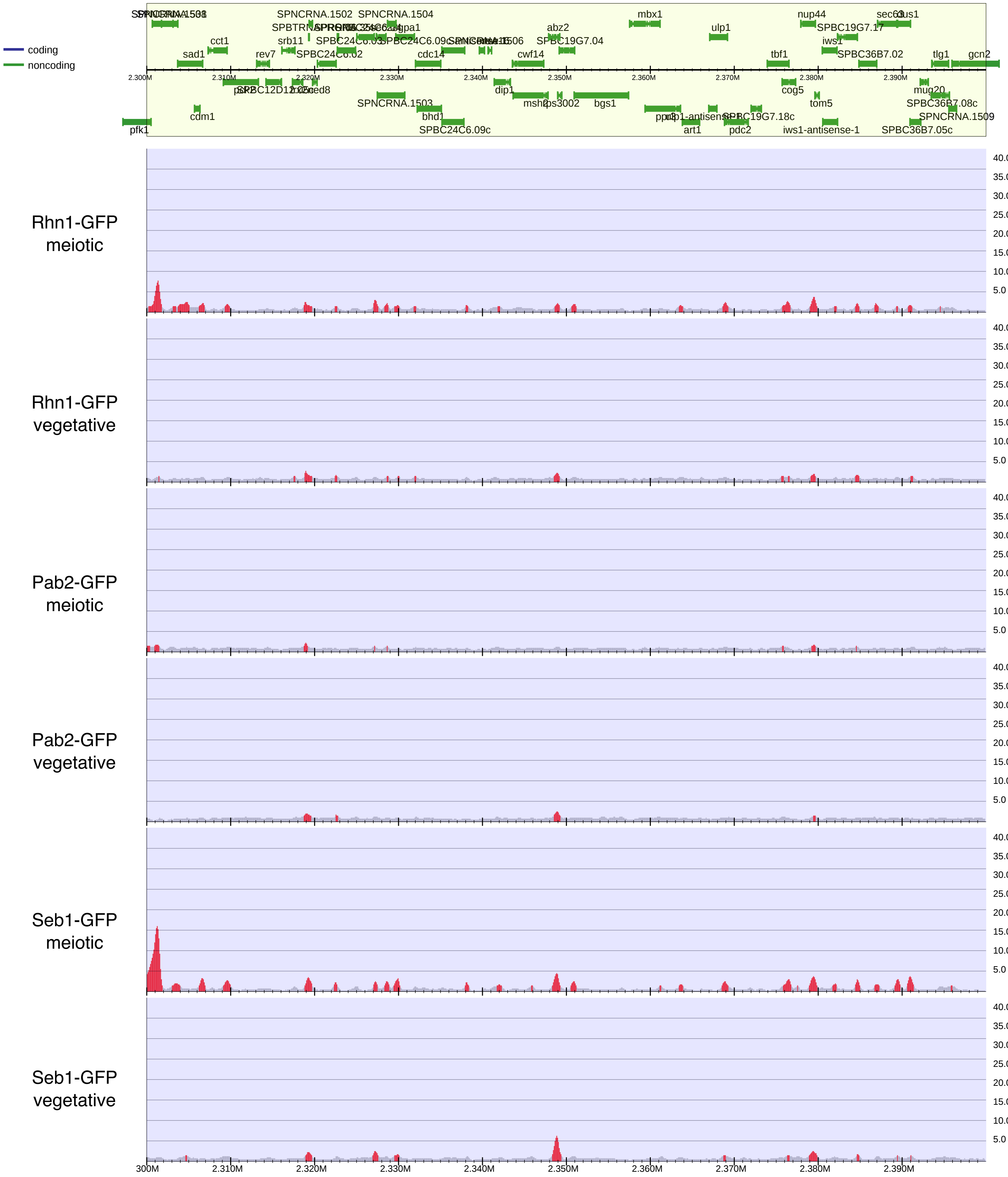
coding
noncoding
Rhn1-GFP meiotic
Rhn1-GFP vegetative
Pab2-GFP meiotic
Pab2-GFP vegetative
Seb1-GFP meiotic
Seb1-GFP vegetative
pfk1
sad1
cct1
cdm1
rev7
srb11
SPNCRNA.1502
SPNCRNA.1504
SPBC24C6.02
SPBC24C6.03
gpa1
cdc14
SPNCRNA.1503
bhd1
SPBC24C6.09c
dip1
cwf14
abz2
SPBC19G7.04
bgs1
mbx1
ulp1
art1
pdc2
tbf1
cog5
tom5
nup44
iws1
iws1-antisense-1
SPBC19G7.17
SPBC36B7.02
SPBC36B7.08c
SPBC36B7.05c
tlg1
gcn2
mug20
SPNCRNA.1509
300M
2.310M
2.320M
2.330M
2.340M
2.350M
2.360M
2.370M
2.380M
2.390M
40.0
35.0
30.0
25.0
20.0
15.0
10.0
5.0

II_1_25

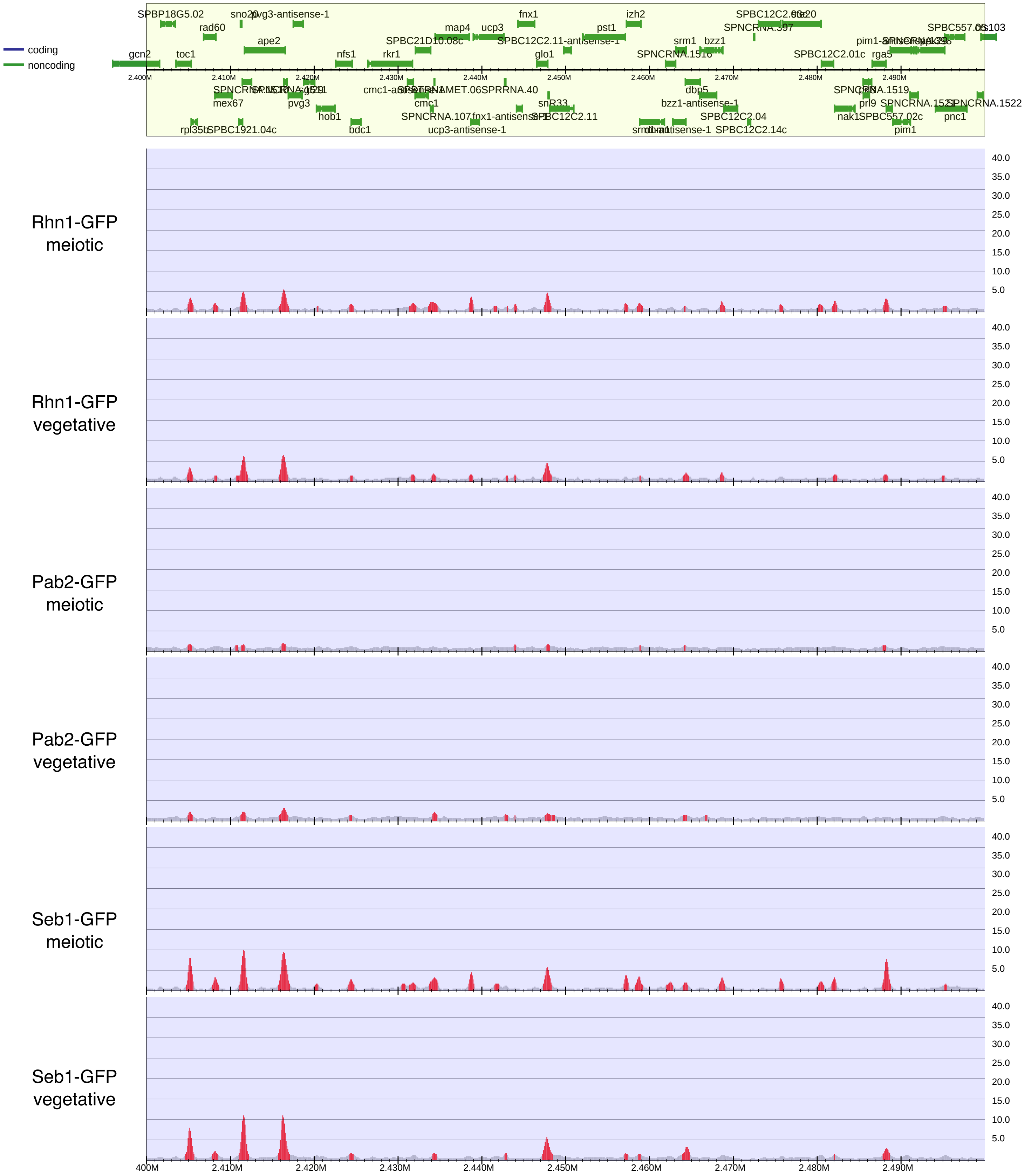
coding
noncoding
Rhn1-GFP meiotic
Rhn1-GFP vegetative
Pab2-GFP meiotic
Pab2-GFP vegetative
Seb1-GFP meiotic
Seb1-GFP vegetative
40.0
35.0
30.0
25.0
20.0
15.0
10.0
5.0
400M
2.410M
2.420M
2.430M
2.440M
2.450M
2.460M
2.470M
2.480M
2.490M

II_1_26

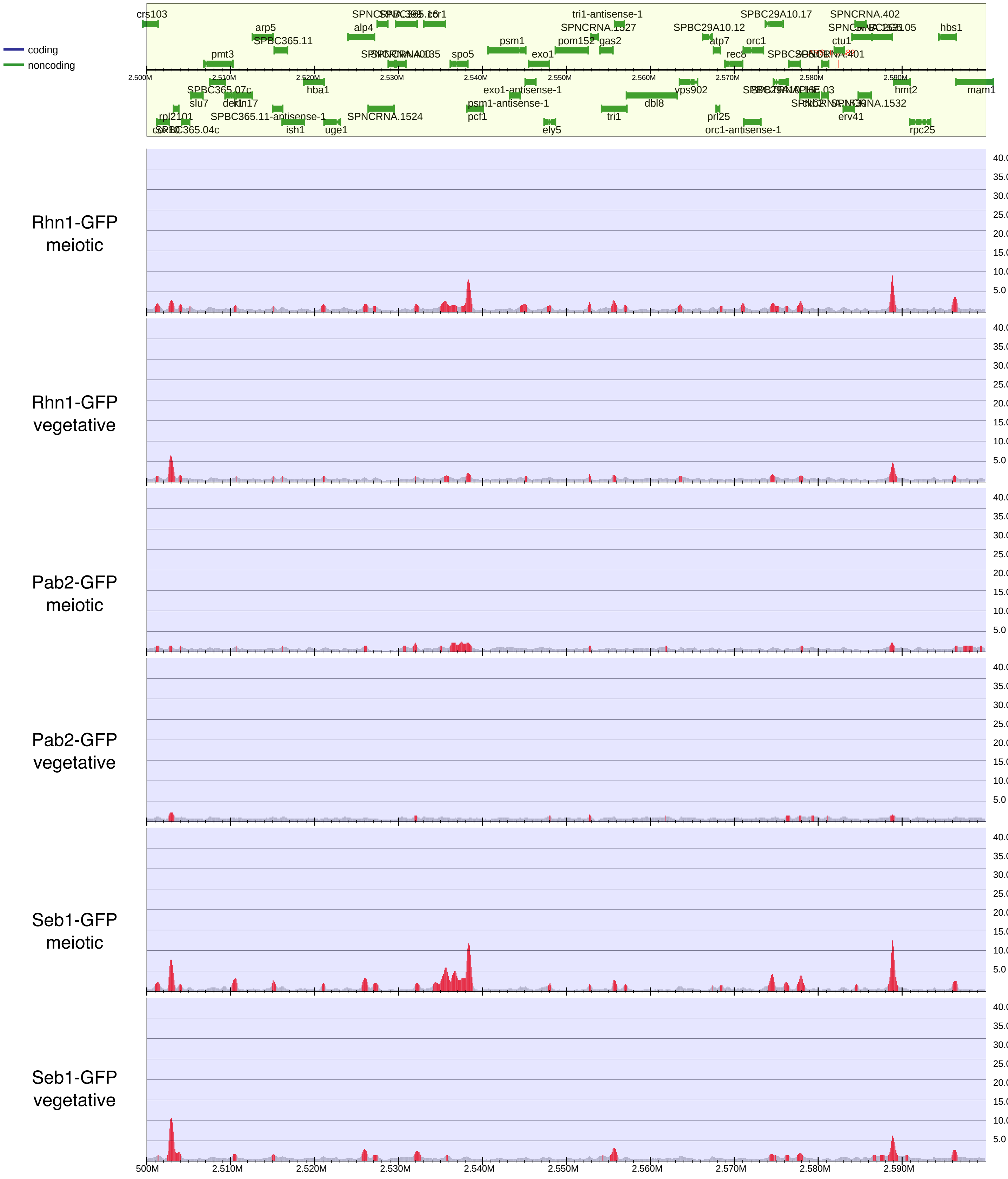
coding
noncoding
Rhn1-GFP meiotic
Rhn1-GFP vegetative
Pab2-GFP meiotic
Pab2-GFP vegetative
Seb1-GFP meiotic
Seb1-GFP vegetative
40.0
35.0
30.0
25.0
20.0
15.0
10.0
5.0
500M
2.510M
2.520M
2.530M
2.540M
2.550M
2.560M
2.570M
2.580M
2.590M

II_1_27

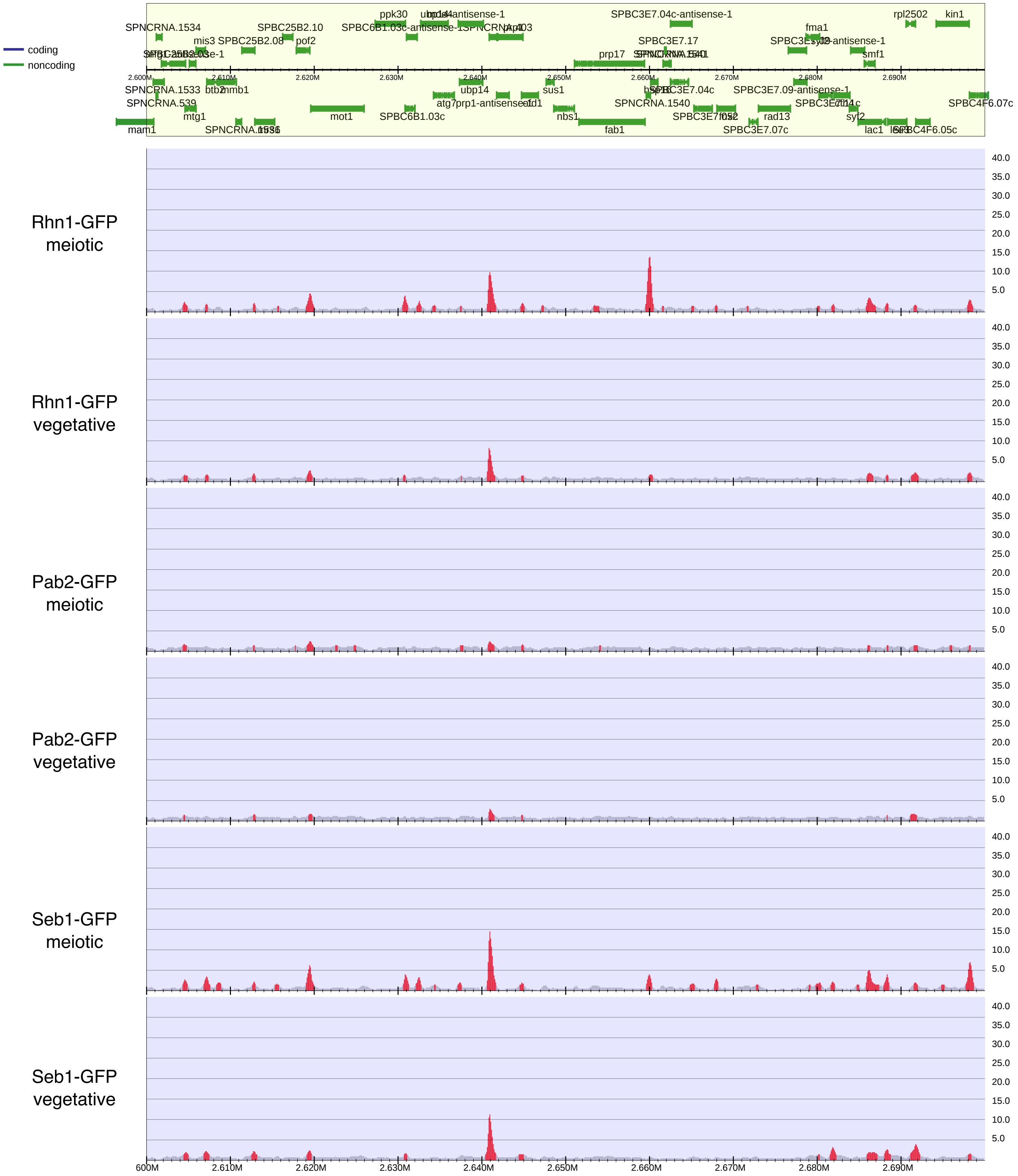

coding
noncoding
SPNCRNA.1534
SPBC25B2.10
ppk30
SPBC3E7.04c-antisense-1
rpl2502
kin1
mis3
pof2
fma1
SPBC3E7.17
prp17
smf1
SPNCRNA.1533
SPNCRNA.539
mtg1
mam1
mot1
SPBC6B1.03c
ubp14
sus1
nbs1
fab1
SPNCRNA.1540
rad13
SPBC3E7.07c
SPBC3E7.09-antisense-1
syt2
lac1
SPBC4F6.07c
SPBC4F6.05c
Rhn1-GFP meiotic
Rhn1-GFP vegetative
Pab2-GFP meiotic
Pab2-GFP vegetative
Seb1-GFP meiotic
Seb1-GFP vegetative
40.0
35.0
30.0
25.0
20.0
15.0
10.0
5.0
600M
2.610M
2.620M
2.630M
2.640M
2.650M
2.660M
2.670M
2.680M
2.690M

II_1_28

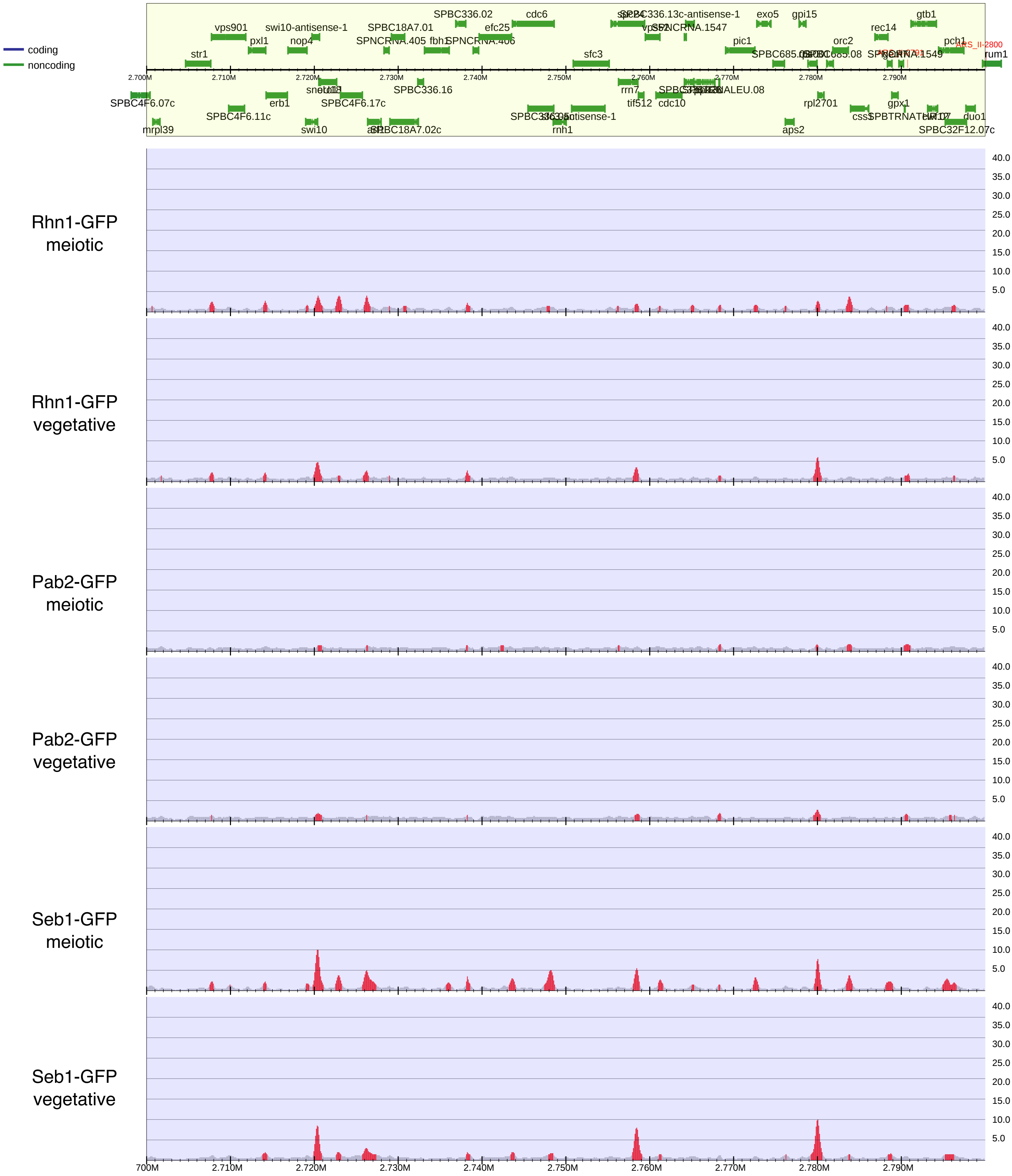

coding
noncoding
Rhn1-GFP meiotic
Rhn1-GFP vegetative
Pab2-GFP meiotic
Pab2-GFP vegetative
Seb1-GFP meiotic
Seb1-GFP vegetative
40.0
35.0
30.0
25.0
20.0
15.0
10.0
5.0
700M
2.710M
2.720M
2.730M
2.740M
2.750M
2.760M
2.770M
2.780M
2.790M
vps901
pxl1
str1
swi10-antisense-1
nop4
SPBC18A7.01
SPNCRNA.405
fbh1
SPBC336.02
efc25
cdc6
sfc3
SPBC336.13c-antisense-1
SPNCRNA.1547
exo5
gpi15
pic1
orc2
rec14
gtb1
pch1
rum1
ARS_II-2800
SPBC4F6.07c
SPBC4F6.11c
erb1
mrpl39
swi10
SPBC4F6.17c
SPBC336.16
SPBC18A7.02c
rnh1
rrn7
tif512
cdc10
rpl2701
aps2
gpx1
duo1
SPBC32F12.07c

II_1_29

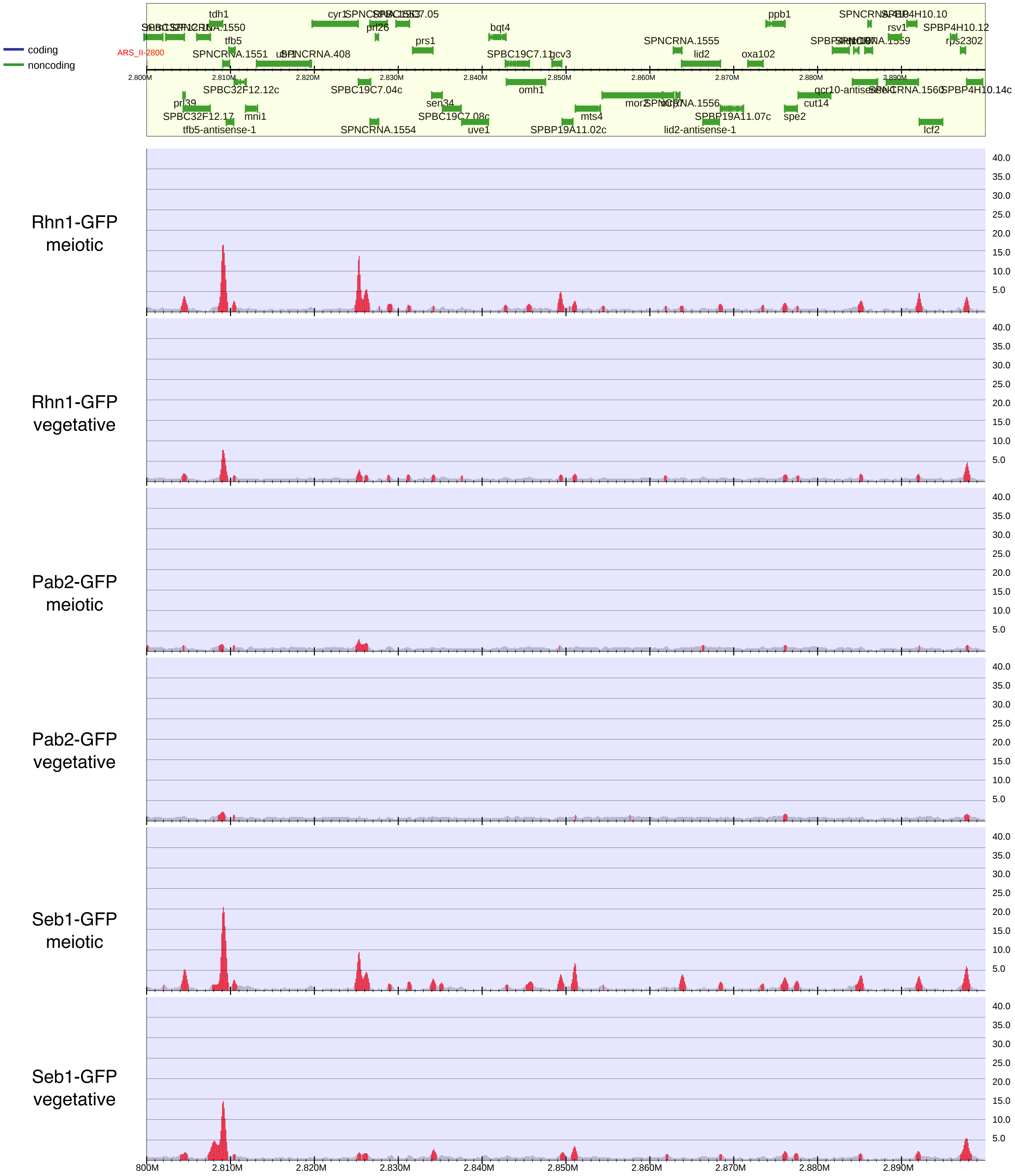

coding
noncoding
ARS_II-2800
tdh1
tfb5
cyr1
prl26
prs1
bqt4
SPBC19C7.04c
omh1
sen34
mts4
uve1
SPBP19A11.02c
lid2
oxa102
ppb1
rsv1
SPBP4H10.12
rps2302
cut14
spe2
lcf2
mni1
prl39
SPBC32F12.12c
SPBC32F12.17
tfb5-antisense-1
SPNCRNA.1554
SPNCRNA.1555
SPNCRNA.1556
SPBP19A11.07c
lid2-antisense-1
Rhn1-GFP meiotic
Rhn1-GFP vegetative
Pab2-GFP meiotic
Pab2-GFP vegetative
Seb1-GFP meiotic
Seb1-GFP vegetative
800M
2.810M
2.820M
2.830M
2.840M
2.850M
2.860M
2.870M
2.880M
2.890M
40.0
35.0
30.0
25.0
20.0
15.0
10.0
5.0

II_1_30

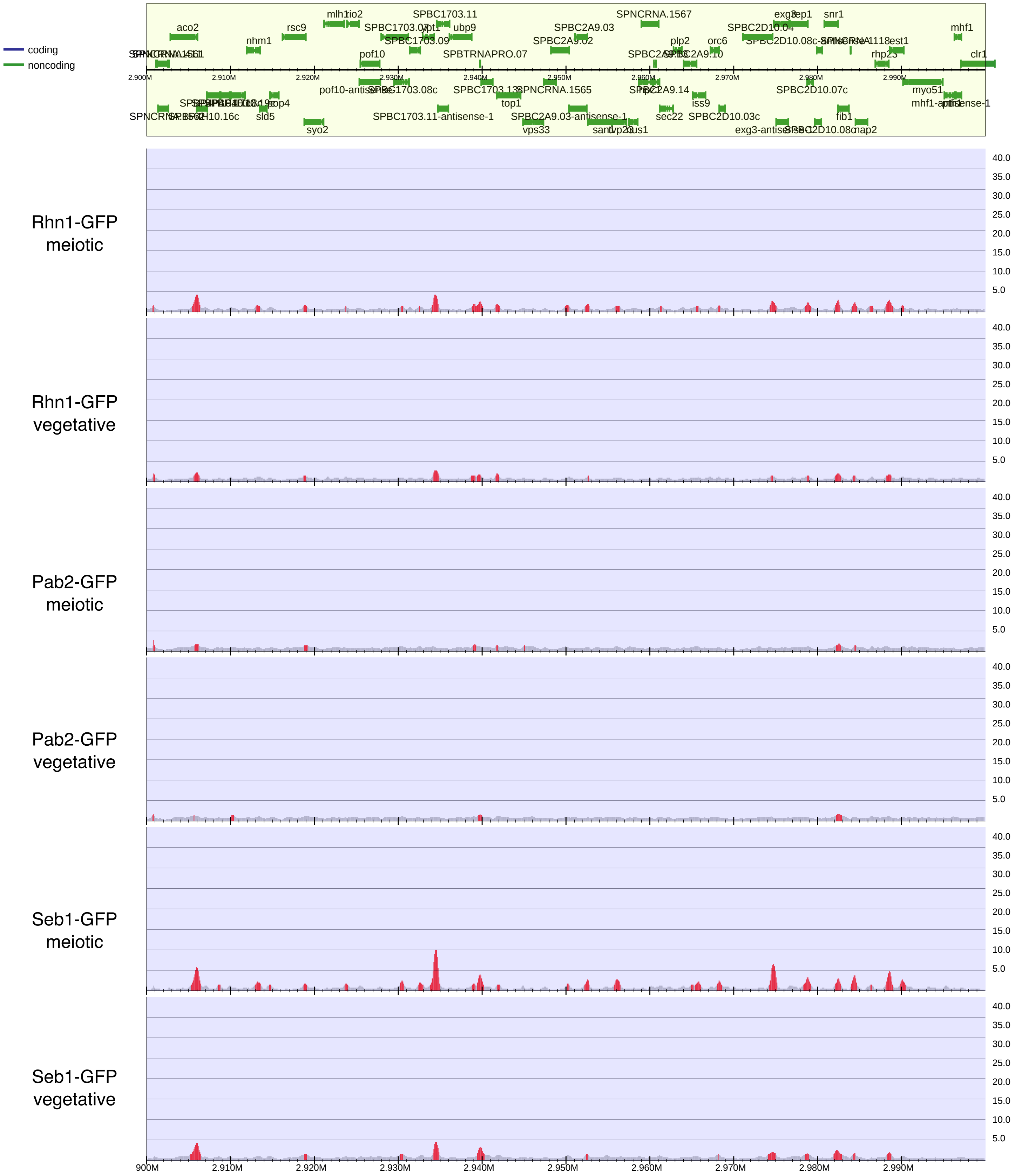
coding
noncoding
aco2
rsc9
nhm1
mlh1
rio2
SPBC1703.11
ubp9
SPBC1703.09
pof10
SPBTRNAPRO.07
SPBC2A9.03
SPBC2A9.02
SPNCRNA.1567
plp2
orc6
SPBC2D10.04
snr1
rhp23
mhf1
clr1
SPBC1703.08c
top1
SPNCRNA.1565
SPBC1703.11-antisense-1
SPBC2A9.03-antisense-1
SPBC2A9.14
iss9
sec22
SPBC2D10.03c
SPBC2D10.07c
fib1
myo51
sld5
syo2
vps33
nap2
2.900M
2.910M
2.920M
2.930M
2.940M
2.950M
2.960M
2.970M
2.980M
2.990M
Rhn1-GFP meiotic
Rhn1-GFP vegetative
Pab2-GFP meiotic
Pab2-GFP vegetative
Seb1-GFP meiotic
Seb1-GFP vegetative
40.0
35.0
30.0
25.0
20.0
15.0
10.0
5.0
900M
2.910M
2.920M
2.930M
2.940M
2.950M
2.960M
2.970M
2.980M
2.990M

II_1_31

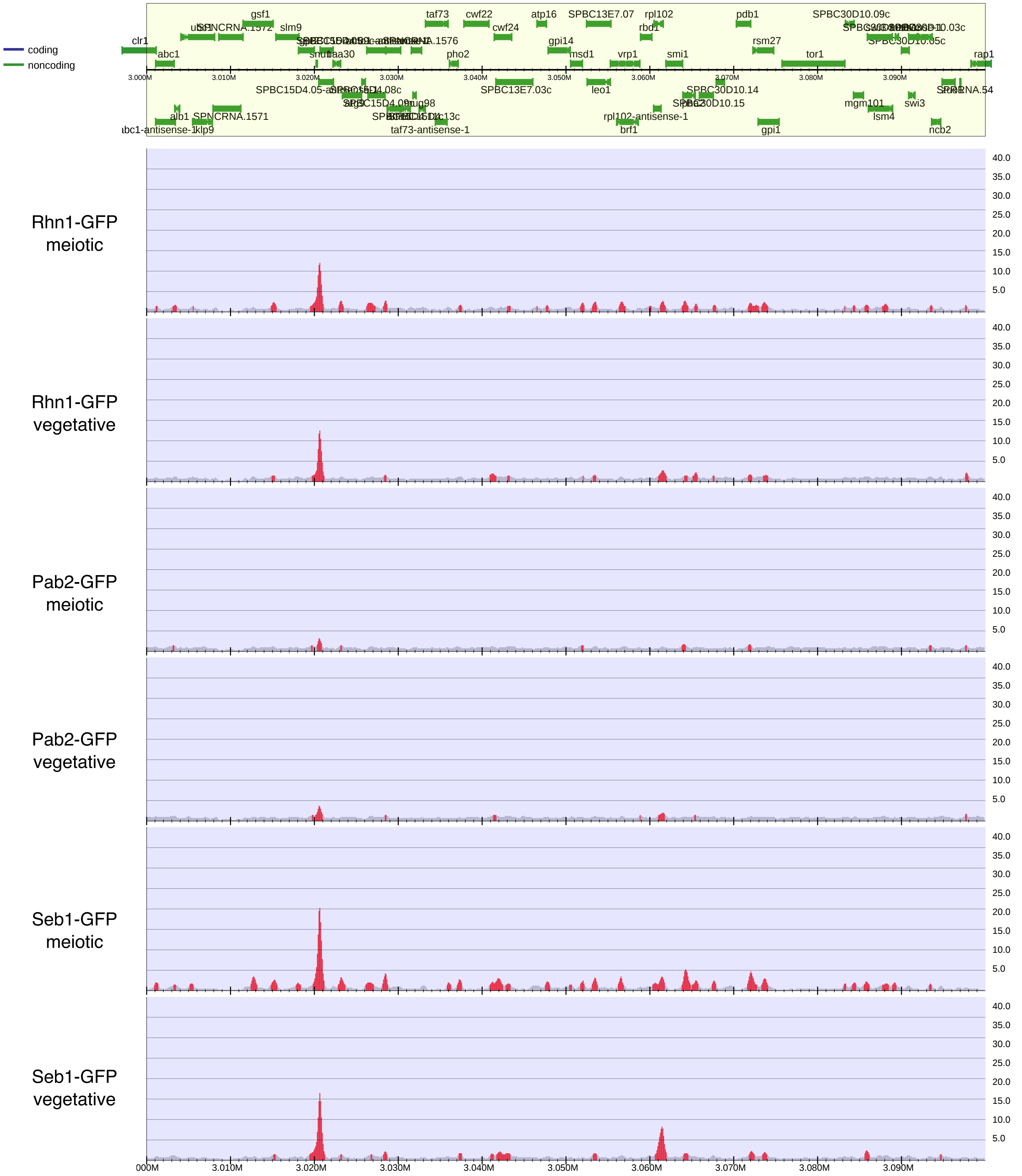

coding
noncoding
Rhn1-GFP meiotic
Rhn1-GFP vegetative
Pab2-GFP meiotic
Pab2-GFP vegetative
Seb1-GFP meiotic
Seb1-GFP vegetative
3.010M
3.020M
3.030M
3.040M
3.050M
3.060M
3.070M
3.080M
3.090M
40.0
35.0
30.0
25.0
20.0
15.0
10.0
5.0

II_1_32

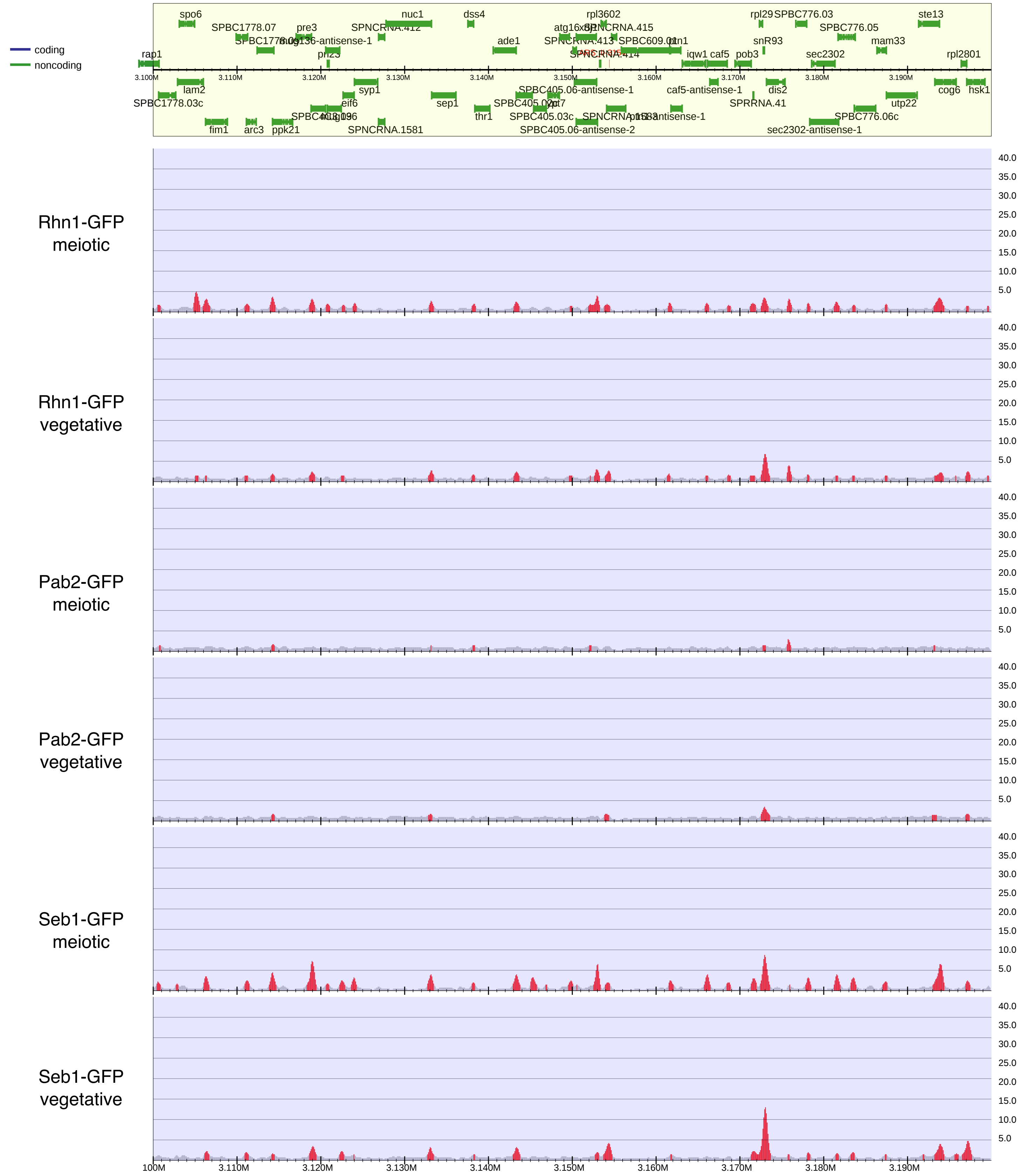
coding
noncoding
spo6
SPBC1778.07
pre3
nuc1
dss4
rpl3602
ade1
rpl29
SPBC776.03
SPBC776.05
ste13
snR93
mam33
rap1
prz3
iqw1
caf5
pob3
sec2302
rpl2801
lam2
syp1
SPBC405.06-antisense-1
caf5-antisense-1
dis2
cog6
hsk1
SPBC1778.03c
eif6
sep1
SPRRNA.41
utp22
thr1
SPBC405.03c
SPBC776.06c
fim1
arc3
ppk21
SPNCRNA.1581
SPBC405.06-antisense-2
sec2302-antisense-1
Rhn1-GFP meiotic
Rhn1-GFP vegetative
Pab2-GFP meiotic
Pab2-GFP vegetative
Seb1-GFP meiotic
Seb1-GFP vegetative
40.0
35.0
30.0
25.0
20.0
15.0
10.0
5.0
100M
3.110M
3.120M
3.130M
3.140M
3.150M
3.160M
3.170M
3.180M
3.190M

II_1_33

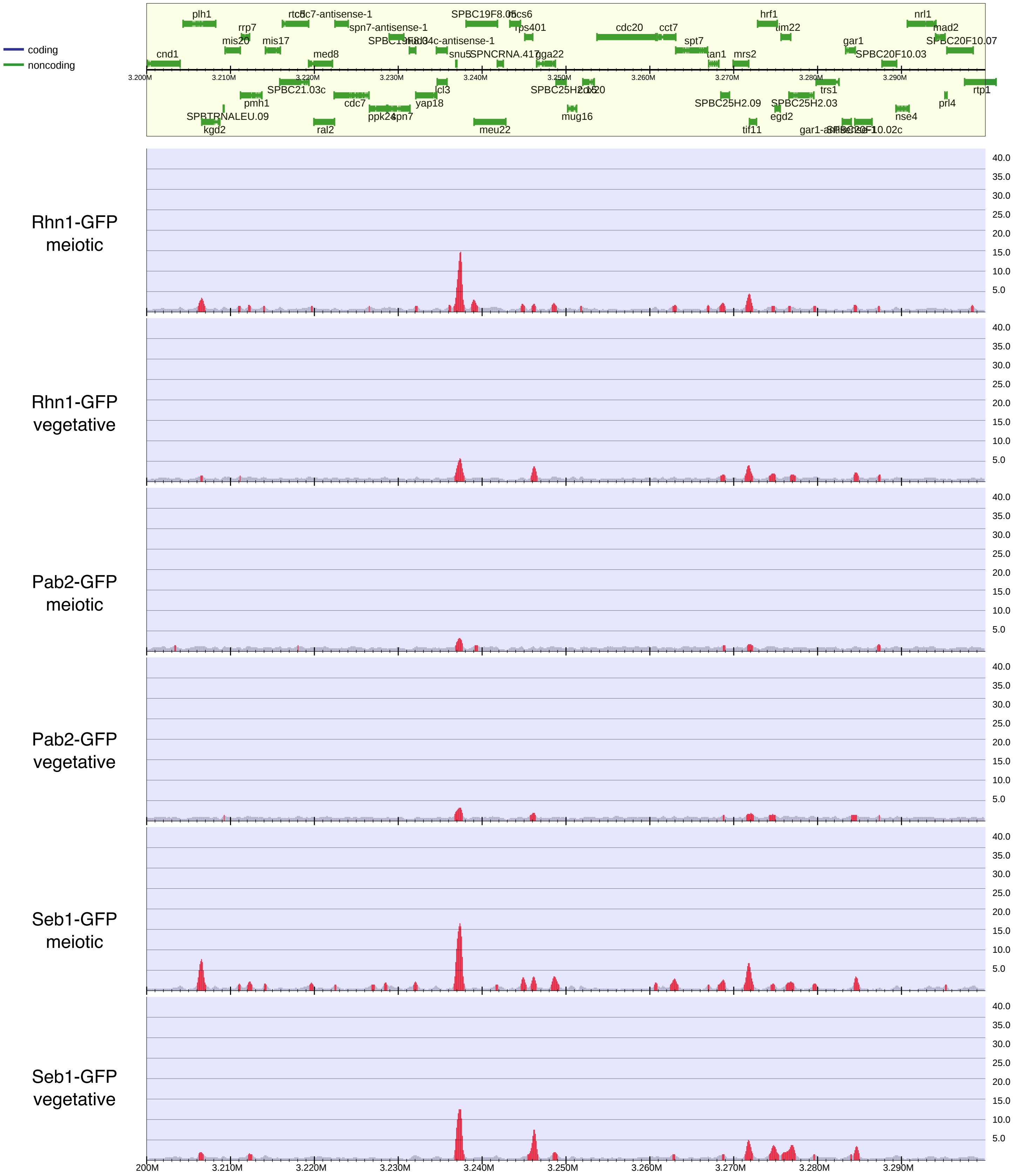

coding
noncoding
Rhn1-GFP meiotic
Rhn1-GFP vegetative
Pab2-GFP meiotic
Pab2-GFP vegetative
Seb1-GFP meiotic
Seb1-GFP vegetative
200M
3.210M
3.220M
3.230M
3.240M
3.250M
3.260M
3.270M
3.280M
3.290M

II_1_34

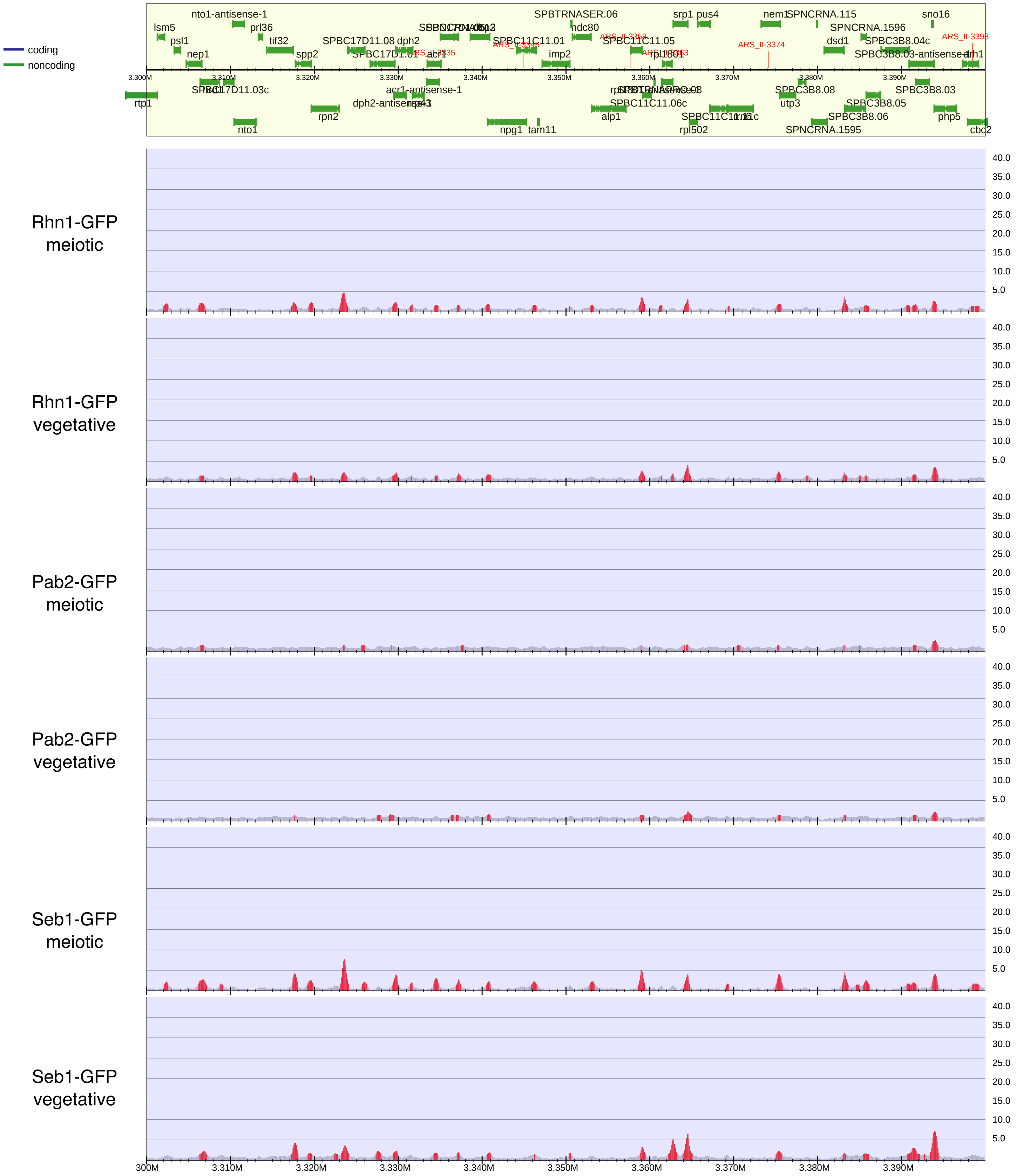

coding
noncoding
Rhn1-GFP meiotic
Rhn1-GFP vegetative
Pab2-GFP meiotic
Pab2-GFP vegetative
Seb1-GFP meiotic
Seb1-GFP vegetative
40.0
35.0
30.0
25.0
20.0
15.0
10.0
5.0
300M
3.310M
3.320M
3.330M
3.340M
3.350M
3.360M
3.370M
3.380M
3.390M

II_1_35

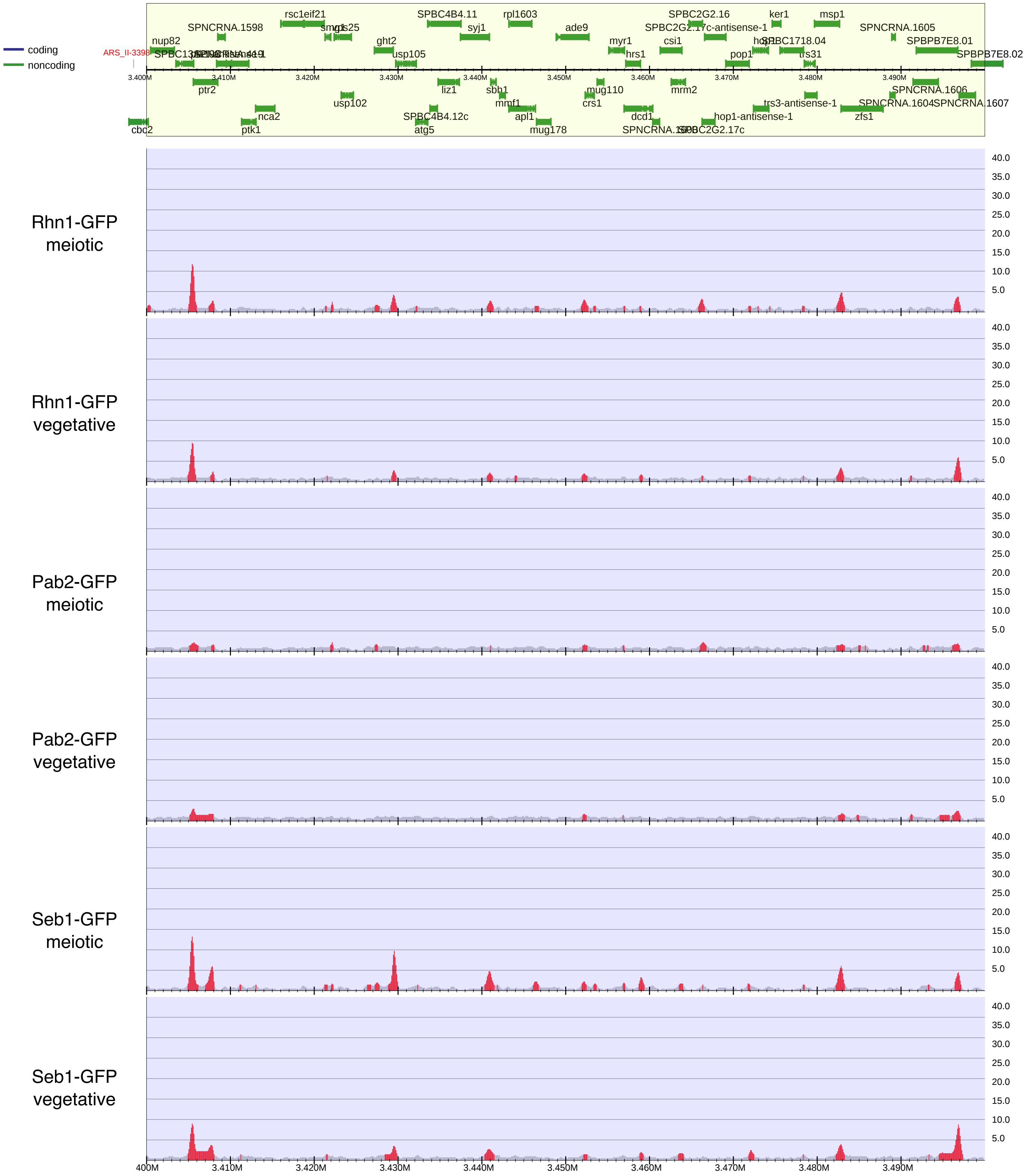

coding
noncoding
ARS_II-3398
rsc1eif21
SPBC4B4.11
rpl1603
SPBC2G2.16
ker1
msp1
SPNCRNA.1598
smg1
gis25
syj1
ade9
SPBC2G2.17c-antisense-1
SPNCRNA.1605
nup82
ght2
myr1
csi1
SPBC1718.04
SPBPB7E8.01
usp105
hrs1
pop1
trs31
SPBPB7E8.02
ptr2
liz1
sbh1
mug110
mrm2
SPNCRNA.1606
usp102
mmf1
crs1
trs3-antisense-1
SPNCRNA.1604
SPNCRNA.1607
nca2
SPBC4B4.12c
apl1
dcd1
hop1-antisense-1
zfs1
cbc2
ptk1
atg5
mug178
SPBC2G2.17c
Rhn1-GFP meiotic
Rhn1-GFP vegetative
Pab2-GFP meiotic
Pab2-GFP vegetative
Seb1-GFP meiotic
Seb1-GFP vegetative
40.0
35.0
30.0
25.0
20.0
15.0
10.0
5.0
3.400M
3.410M
3.420M
3.430M
3.440M
3.450M
3.460M
3.470M
3.480M
3.490M
400M

II_1_36

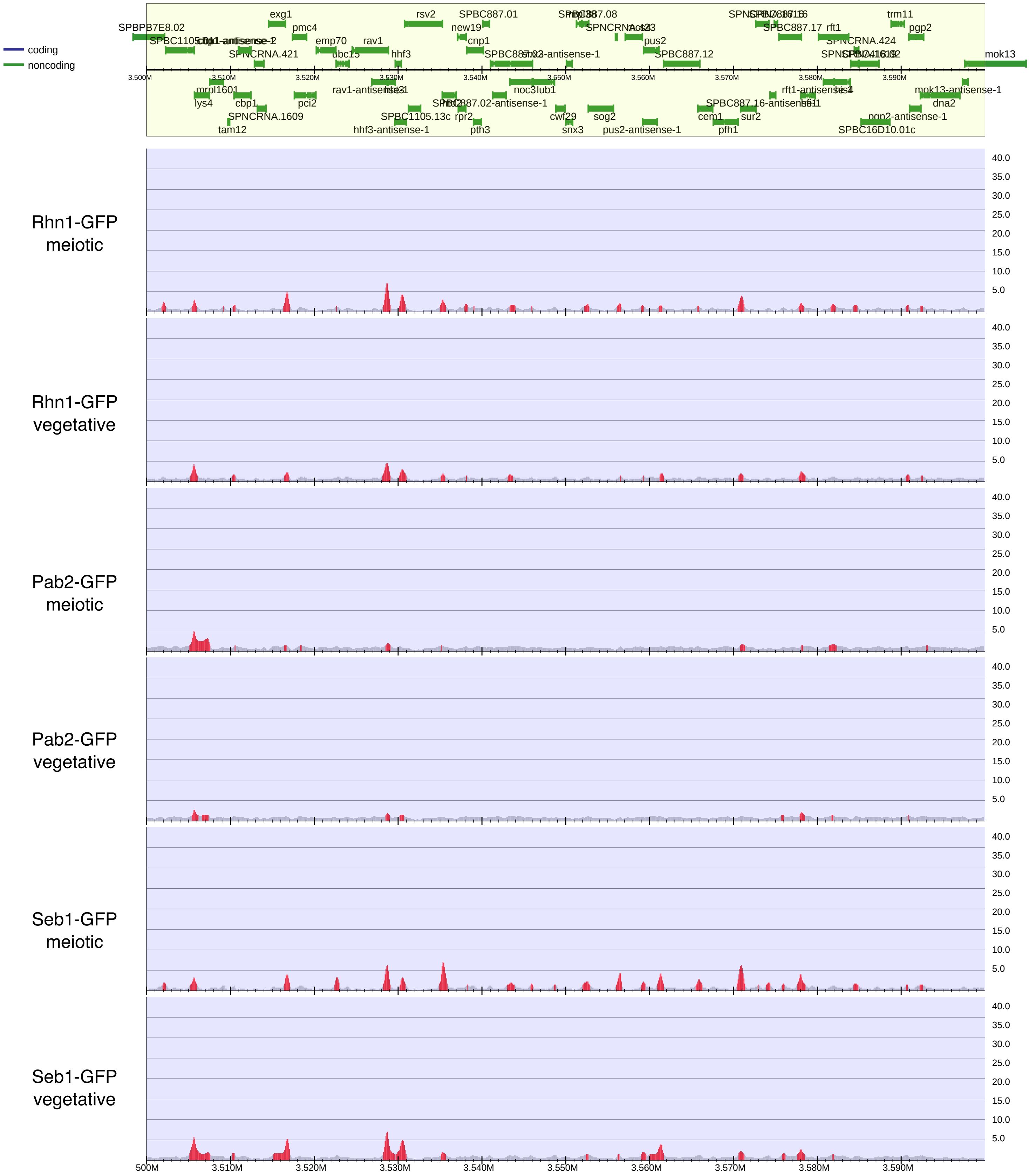
coding
noncoding
Rhn1-GFP meiotic
Rhn1-GFP vegetative
Pab2-GFP meiotic
Pab2-GFP vegetative
Seb1-GFP meiotic
Seb1-GFP vegetative
40.0
35.0
30.0
25.0
20.0
15.0
10.0
5.0
500M
3.510M
3.520M
3.530M
3.540M
3.550M
3.560M
3.570M
3.580M
3.590M

II_1_37

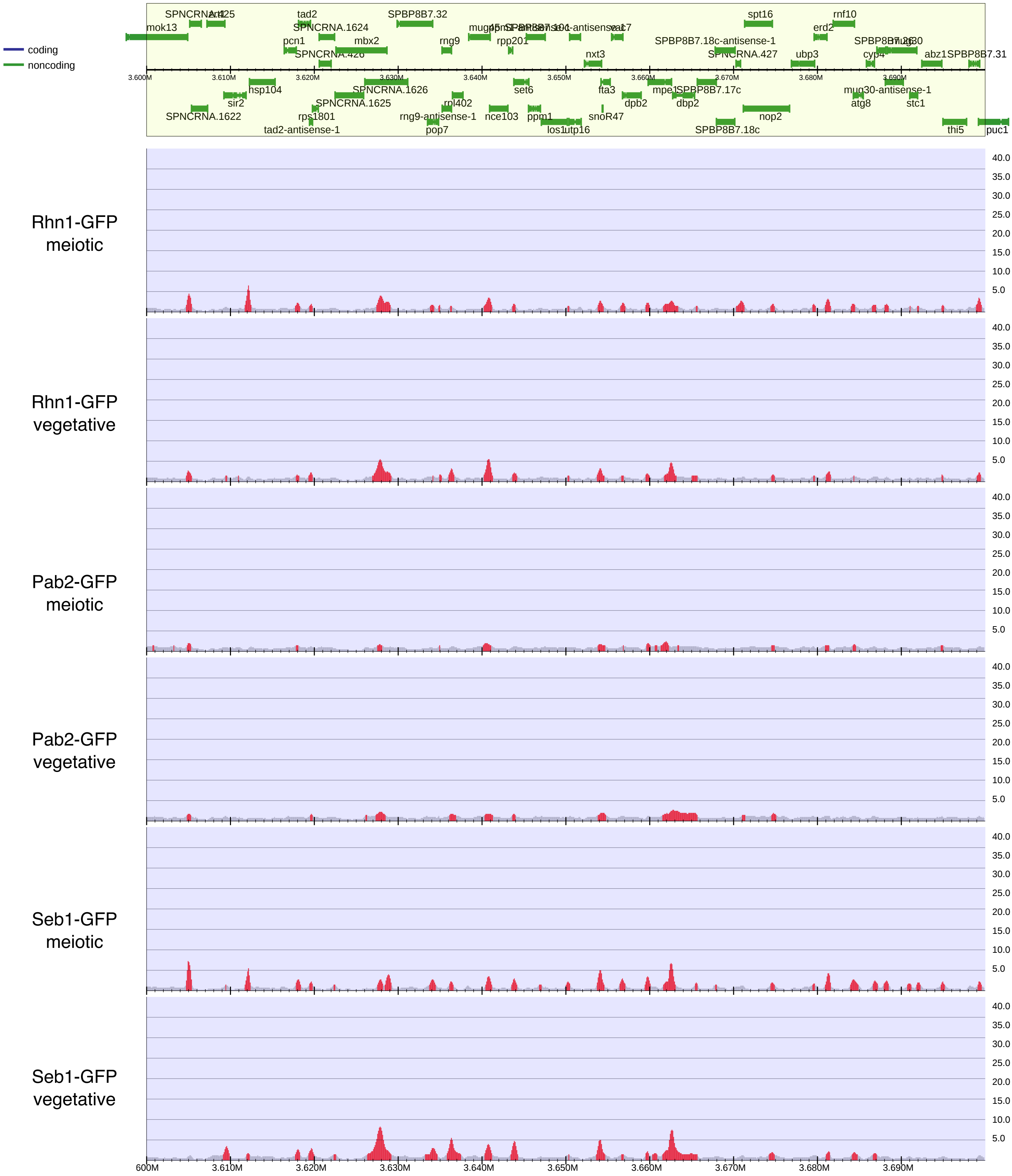
coding
noncoding
Rhn1-GFP meiotic
Rhn1-GFP vegetative
Pab2-GFP meiotic
Pab2-GFP vegetative
Seb1-GFP meiotic
Seb1-GFP vegetative
mok13
SPNCRNA.1625
tad2
SPBP8B7.32
spt16
rnf10
erd2
pcn1
mbx2
rng9
rpp201
nxt3
ubp3
cyp4
abz1
hsp104
SPNCRNA.1626
set6
fta3
sir2
rpl402
dpb2
dbp2
atg8
stc1
SPNCRNA.1622
rps1801
rng9-antisense-1
nce103
ppm1
snoR47
nop2
tad2-antisense-1
pop7
los1
utp16
SPBP8B7.18c
thi5
puc1
40.0
35.0
30.0
25.0
20.0
15.0
10.0
5.0
600M
3.610M
3.620M
3.630M
3.640M
3.650M
3.660M
3.670M
3.680M
3.690M

II_1_38

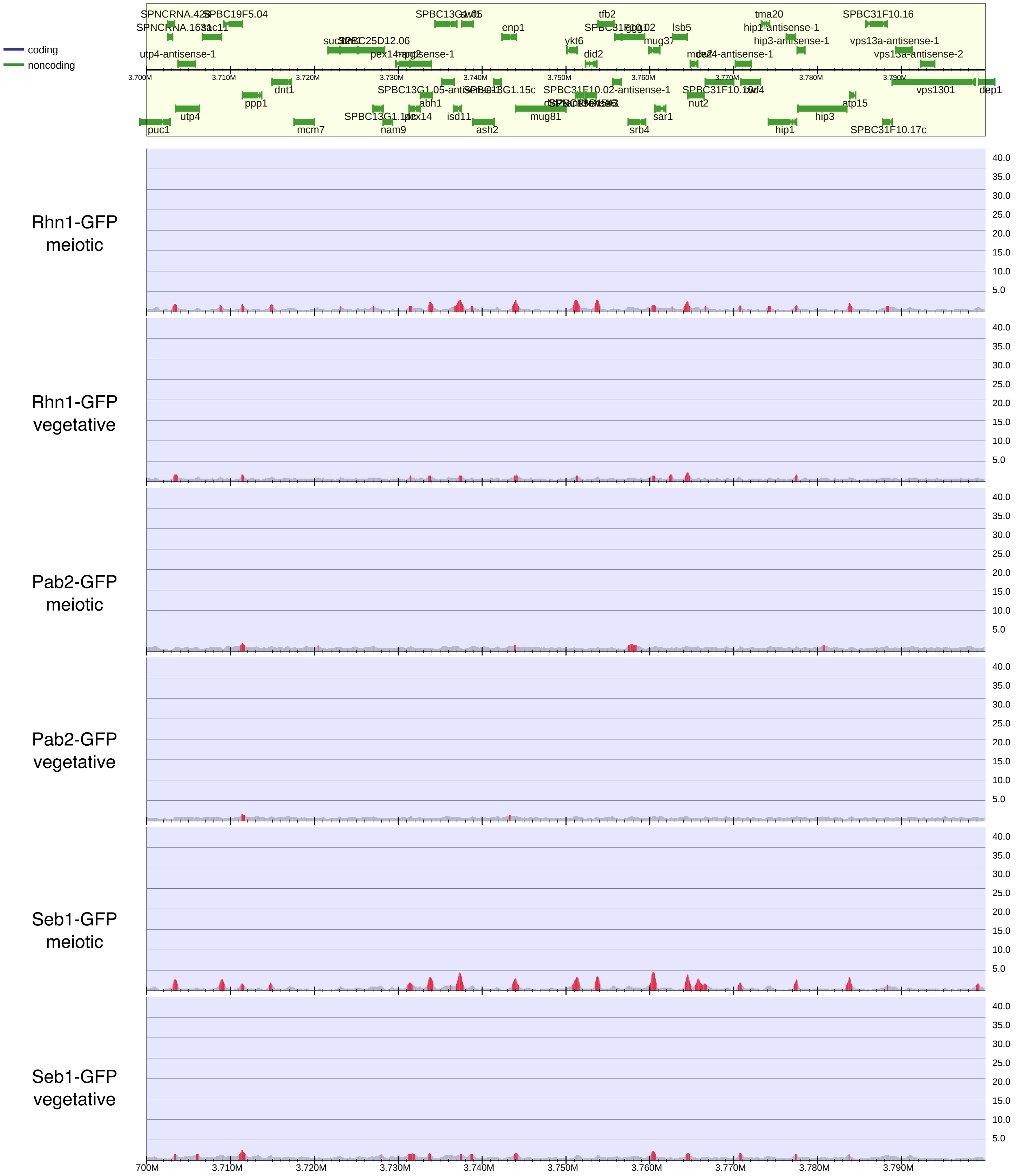
coding
noncoding
Rhn1-GFP meiotic
Rhn1-GFP vegetative
Pab2-GFP meiotic
Pab2-GFP vegetative
Seb1-GFP meiotic
Seb1-GFP vegetative
40.0
35.0
30.0
25.0
20.0
15.0
10.0
5.0
700M
3.710M
3.720M
3.730M
3.740M
3.750M
3.760M
3.770M
3.780M
3.790M

II_1_39

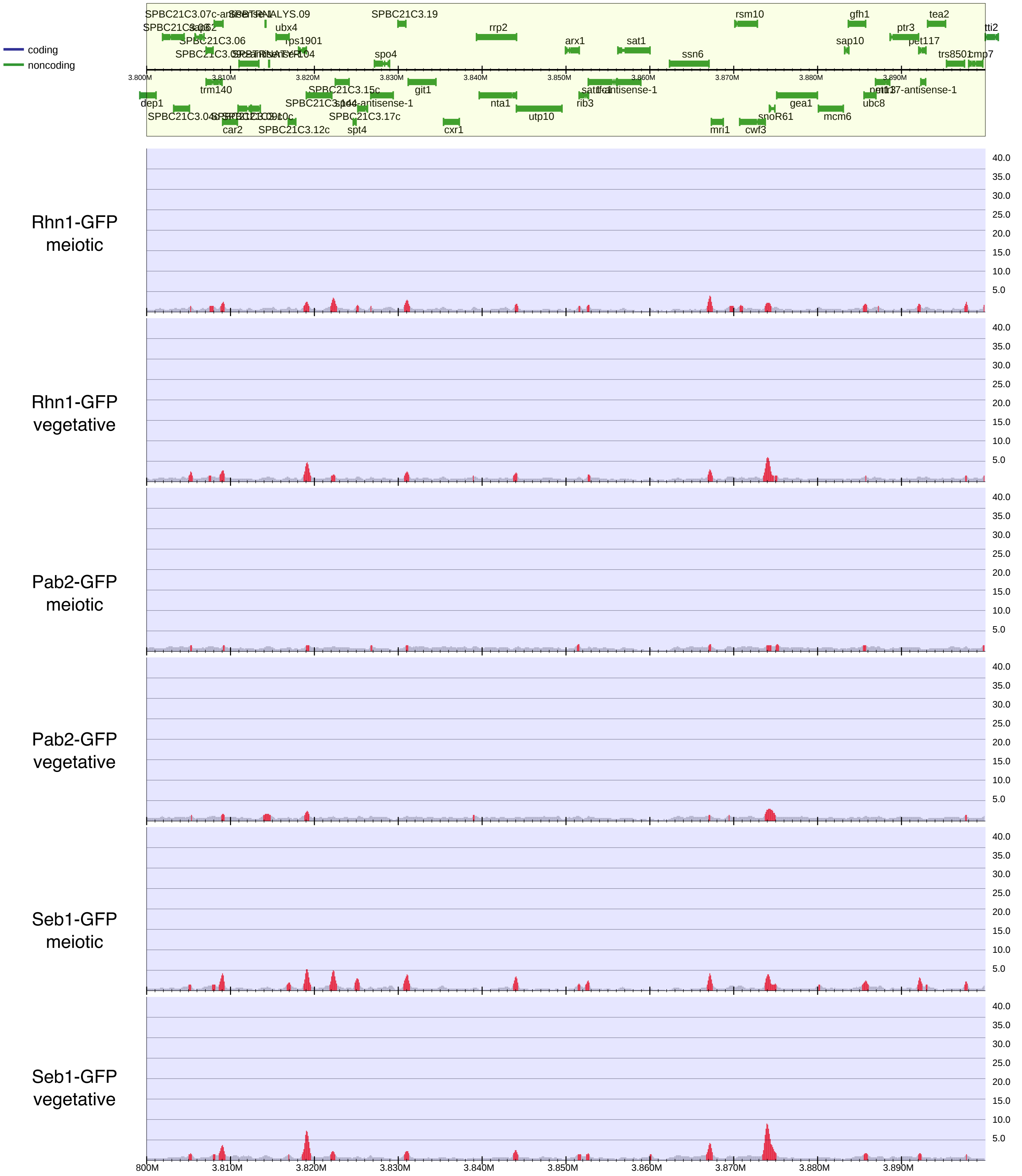
coding
noncoding
Rhn1-GFP meiotic
Rhn1-GFP vegetative
Pab2-GFP meiotic
Pab2-GFP vegetative
Seb1-GFP meiotic
Seb1-GFP vegetative
3.800M
3.810M
3.820M
3.830M
3.840M
3.850M
3.860M
3.870M
3.880M
3.890M
40.0
35.0
30.0
25.0
20.0
15.0
10.0
5.0
rsm10
gfh1
tea2
ptr3
tti2
sap10
pet117
rrp2
arx1
sat1
ssn6
spo4
ubx4
rps1901
trm140
git1
nta1
utp10
rib3
dep1
car2
spt4
cxr1
mri1
cwf3
snoR61
gea1
mcm6
ubc8
SPBC21C3.19
SPBC21C3.06
SPBC21C3.15c
SPBC21C3.17c
SPBC21C3.12c

II_1_40

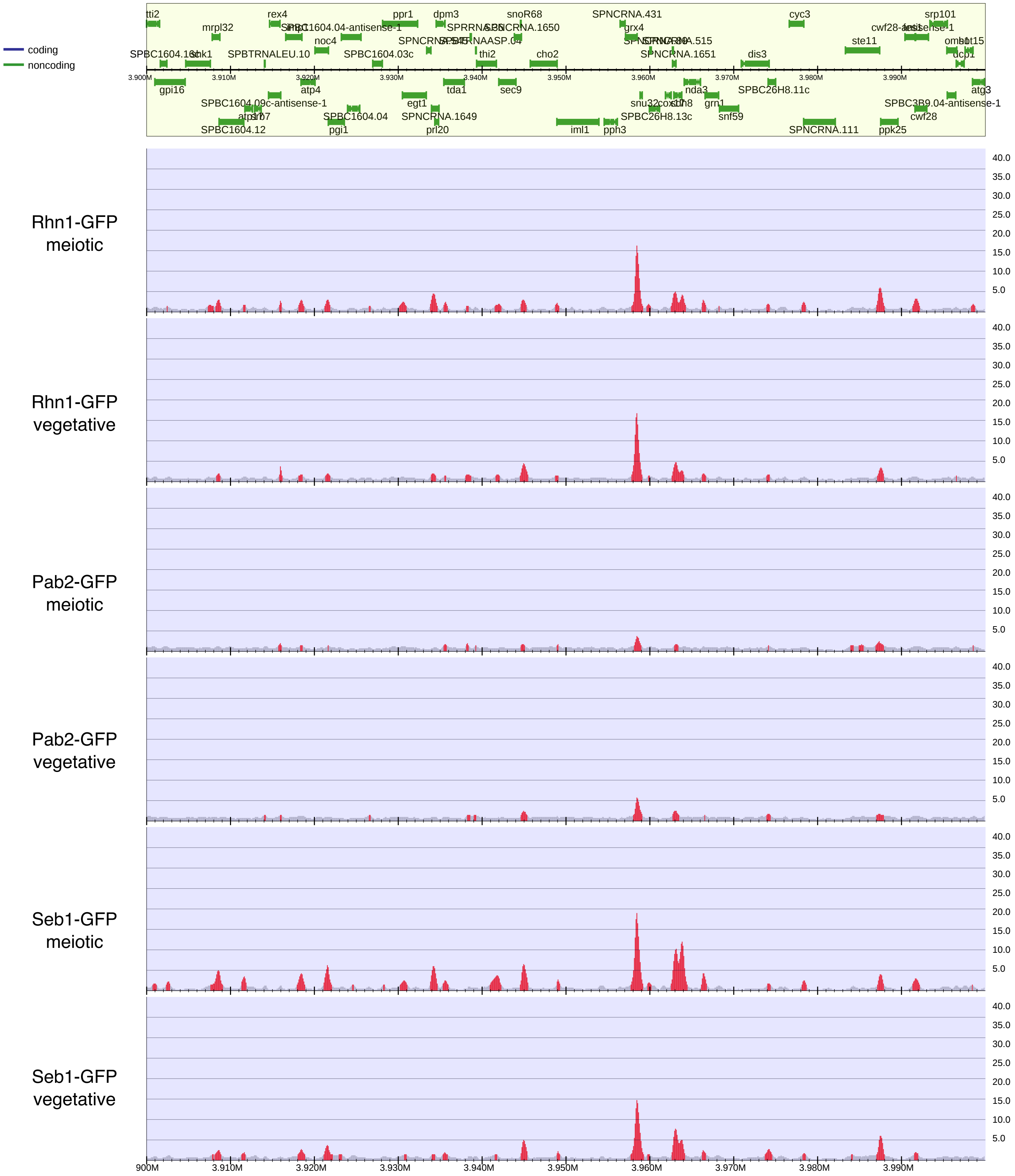
coding
noncoding
tti2
mrpl32
rex4
ppr1
dpm3
snoR68
SPNCRNA.431
cyc3
srp101
noc4
SPBC1604.03c
thi2
cho2
grx4
SPNCRNA.1651
dis3
ste11
SPBTRNALEU.10
gpi16
atp4
tda1
sec9
nda3
SPBC26H8.11c
atg3
SPBC1604.09c-antisense-1
egt1
grn1
SPBC3B9.04-antisense-1
SPBC1604.04
SPNCRNA.1649
SPBC26H8.13c
snf59
cwf28
SPBC1604.12
pgi1
prl20
iml1
pph3
SPNCRNA.111
ppk25
Rhn1-GFP meiotic
Rhn1-GFP vegetative
Pab2-GFP meiotic
Pab2-GFP vegetative
Seb1-GFP meiotic
Seb1-GFP vegetative
40.0
35.0
30.0
25.0
20.0
15.0
10.0
5.0
900M
3.910M
3.920M
3.930M
3.940M
3.950M
3.960M
3.970M
3.980M
3.990M

II_1_41

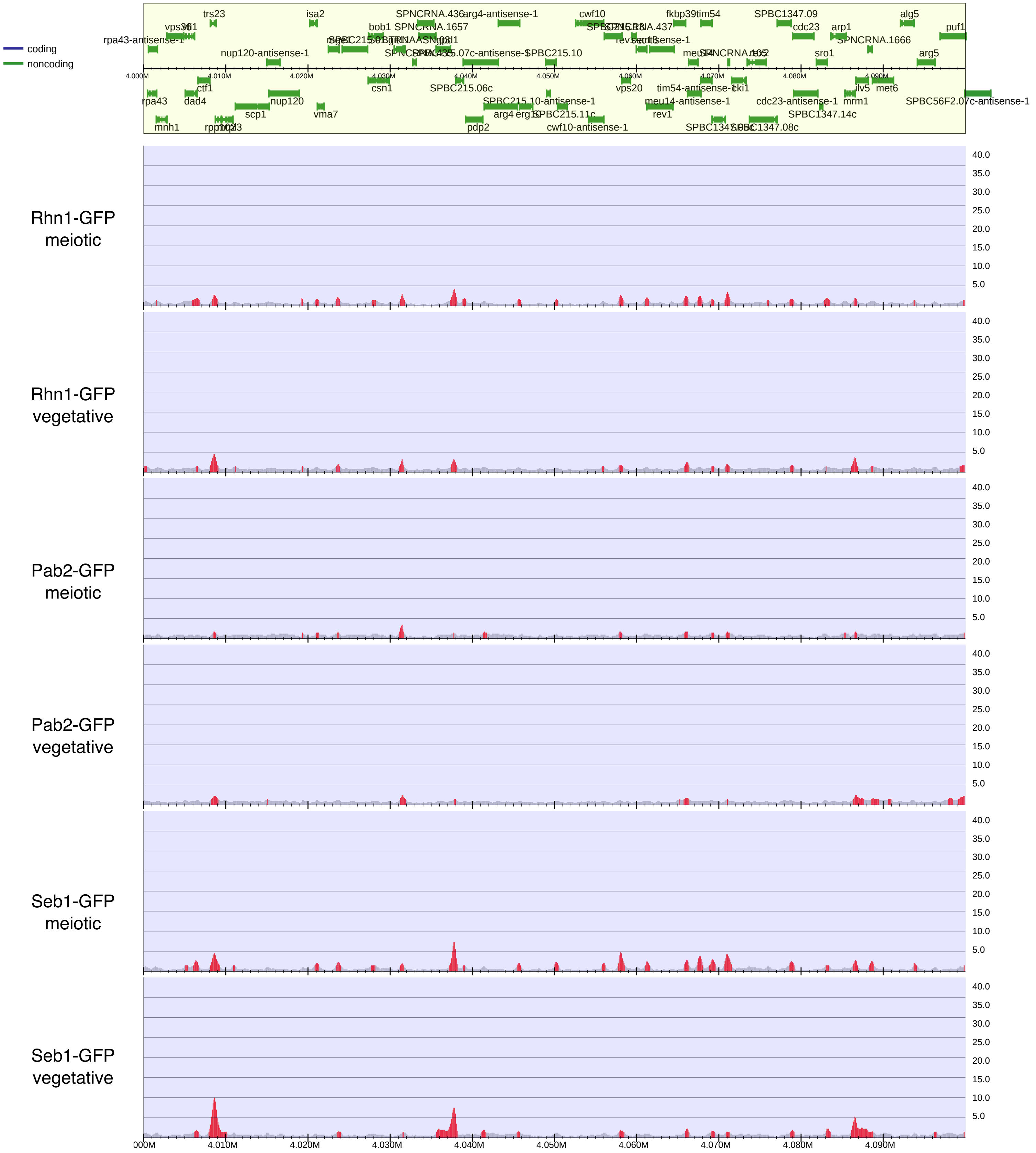
coding
noncoding
Rhn1-GFP meiotic
Rhn1-GFP vegetative
Pab2-GFP meiotic
Pab2-GFP vegetative
Seb1-GFP meiotic
Seb1-GFP vegetative
40.0
35.0
30.0
25.0
20.0
15.0
10.0
5.0
000M
4.010M
4.020M
4.030M
4.040M
4.050M
4.060M
4.070M
4.080M
4.090M

II_1_42

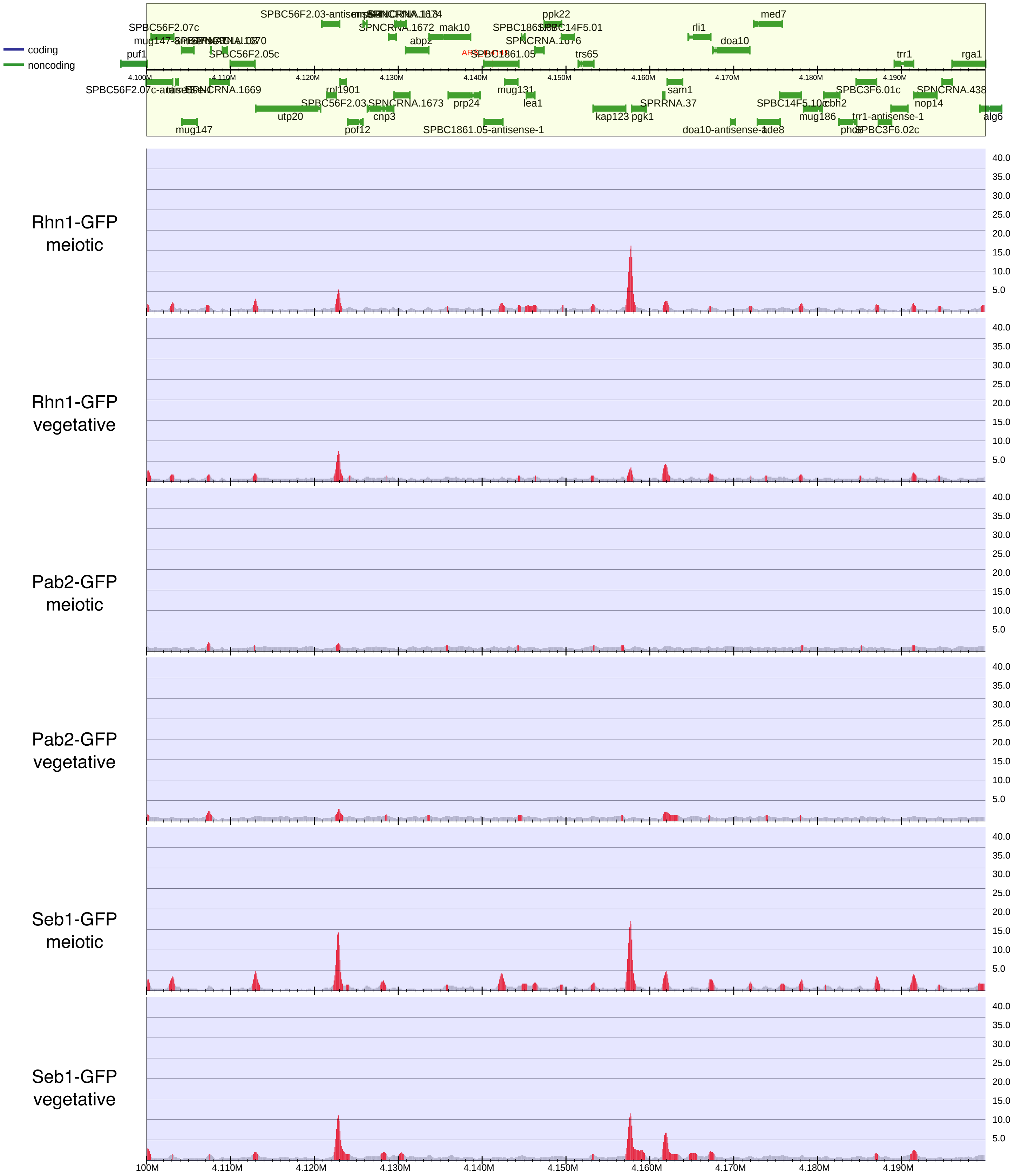
coding
noncoding
SPBC56F2.07c
puf1
SPBC56F2.05c
SPNCRNA.1672
mak10
abp2
ppk22
SPBC14F5.01
SPNCRNA.1676
SPBC1861.05
trs65
rli1
doa10
med7
trr1
rga1
rnl1901
SPNCRNA.1669
utp20
mug147
pof12
SPNCRNA.1673
cnp3
prp24
mug131
lea1
SPBC1861.05-antisense-1
kap123
pgk1
SPRRNA.37
sam1
doa10-antisense-1
ade8
cbh2
mug186
SPBC3F6.01c
trr1-antisense-1
SPBC3F6.02c
SPNCRNA.438
nop14
alg6
Rhn1-GFP meiotic
Rhn1-GFP vegetative
Pab2-GFP meiotic
Pab2-GFP vegetative
Seb1-GFP meiotic
Seb1-GFP vegetative
40.0
35.0
30.0
25.0
20.0
15.0
10.0
5.0
100M
4.110M
4.120M
4.130M
4.140M
4.150M
4.160M
4.170M
4.180M
4.190M

II_1_43

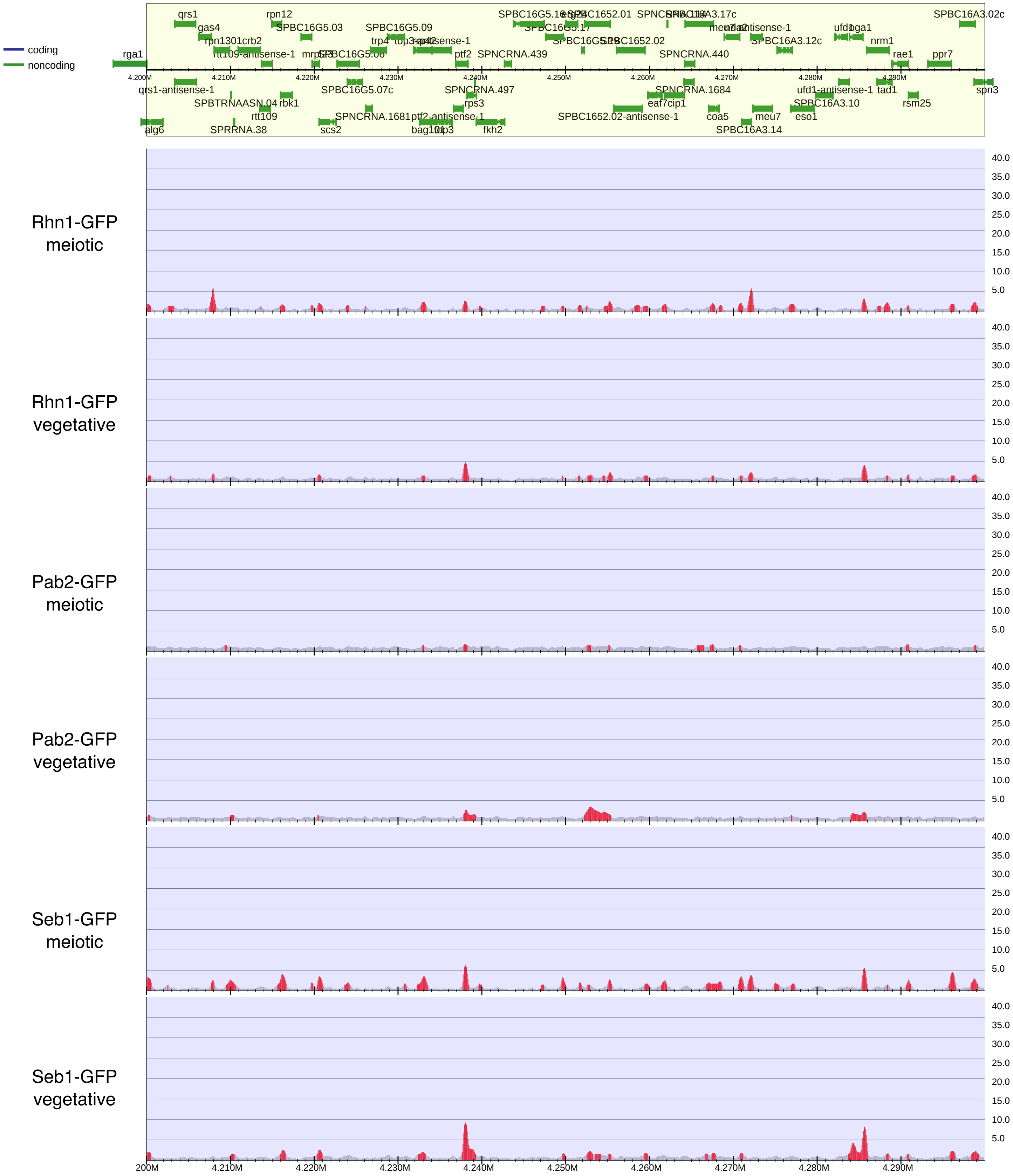

coding
noncoding
qrs1
gas4
rpn12
SPBC16G5.03
SPBC16G5.09
trp4
rga1
ptf2
SPNCRNA.439
SPNCRNA.440
SPBC1652.02
SPBC16A3.12c
nrm1
rae1
ppr7
SPBC16A3.02c
ufd1
4.200M
4.210M
4.220M
4.230M
4.240M
4.250M
4.260M
4.270M
4.280M
4.290M
qrs1-antisense-1
SPBC16G5.07c
SPNCRNA.497
SPNCRNA.1684
ufd1-antisense-1
tad1
spn3
rbk1
rps3
eaf7cip1
SPBC16A3.10
rsm25
rtt109
SPNCRNA.1681
SPBC1652.02-antisense-1
coa5
meu7
eso1
alg6
SPRRNA.38
scs2
fkh2
SPBC16A3.14
Rhn1-GFP meiotic
Rhn1-GFP vegetative
Pab2-GFP meiotic
Pab2-GFP vegetative
Seb1-GFP meiotic
Seb1-GFP vegetative
40.0
35.0
30.0
25.0
20.0
15.0
10.0
5.0
200M
4.210M
4.220M
4.230M
4.240M
4.250M
4.260M
4.270M
4.280M
4.290M

II_1_44

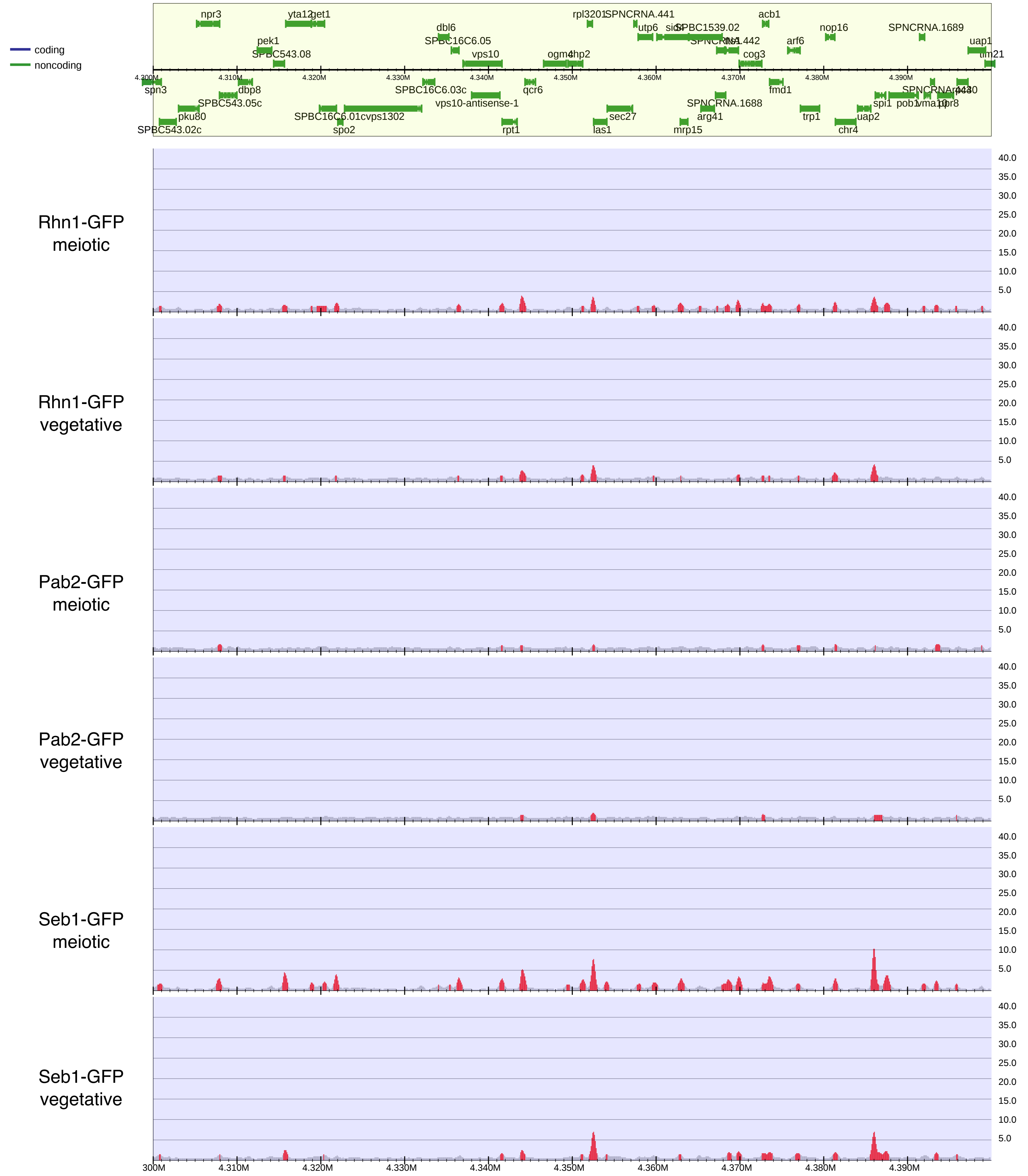
coding
noncoding
npr3
yta12
get1
dbl6
rpl3201
SPNCRNA.441
acb1
utp6
SPBC1539.02
nop16
SPNCRNA.1689
pek1
SPBC16C6.05
arf6
uap1
SPBC543.08
vps10
ogm4
hhp2
cog3
tim21
spn3
dbp8
SPBC16C6.03c
qcr6
fmd1
SPBC543.05c
vps10-antisense-1
SPNCRNA.1688
spi1
pob1
vma10
pku80
SPBC16C6.01c
vps1302
sec27
arg41
trp1
uap2
SPBC543.02c
spo2
rpt1
las1
mrp15
chr4
Rhn1-GFP meiotic
Rhn1-GFP vegetative
Pab2-GFP meiotic
Pab2-GFP vegetative
Seb1-GFP meiotic
Seb1-GFP vegetative
40.0
35.0
30.0
25.0
20.0
15.0
10.0
5.0
4.300M
4.310M
4.320M
4.330M
4.340M
4.350M
4.360M
4.370M
4.380M
4.390M

II_1_45

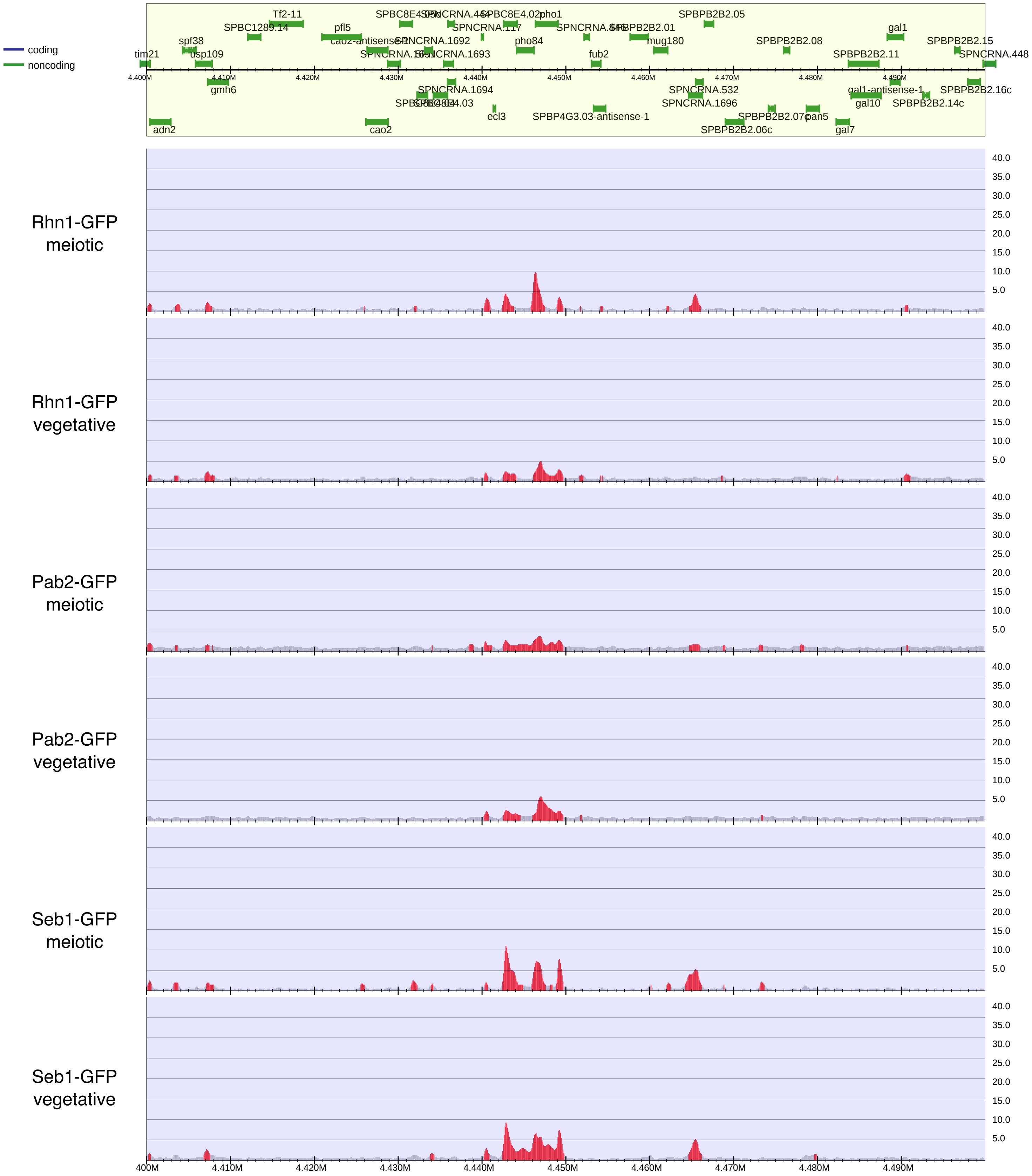

coding
noncoding
Rhn1-GFP meiotic
Rhn1-GFP vegetative
Pab2-GFP meiotic
Pab2-GFP vegetative
Seb1-GFP meiotic
Seb1-GFP vegetative
400M
4.410M
4.420M
4.430M
4.440M
4.450M
4.460M
4.470M
4.480M
4.490M

II_1_46

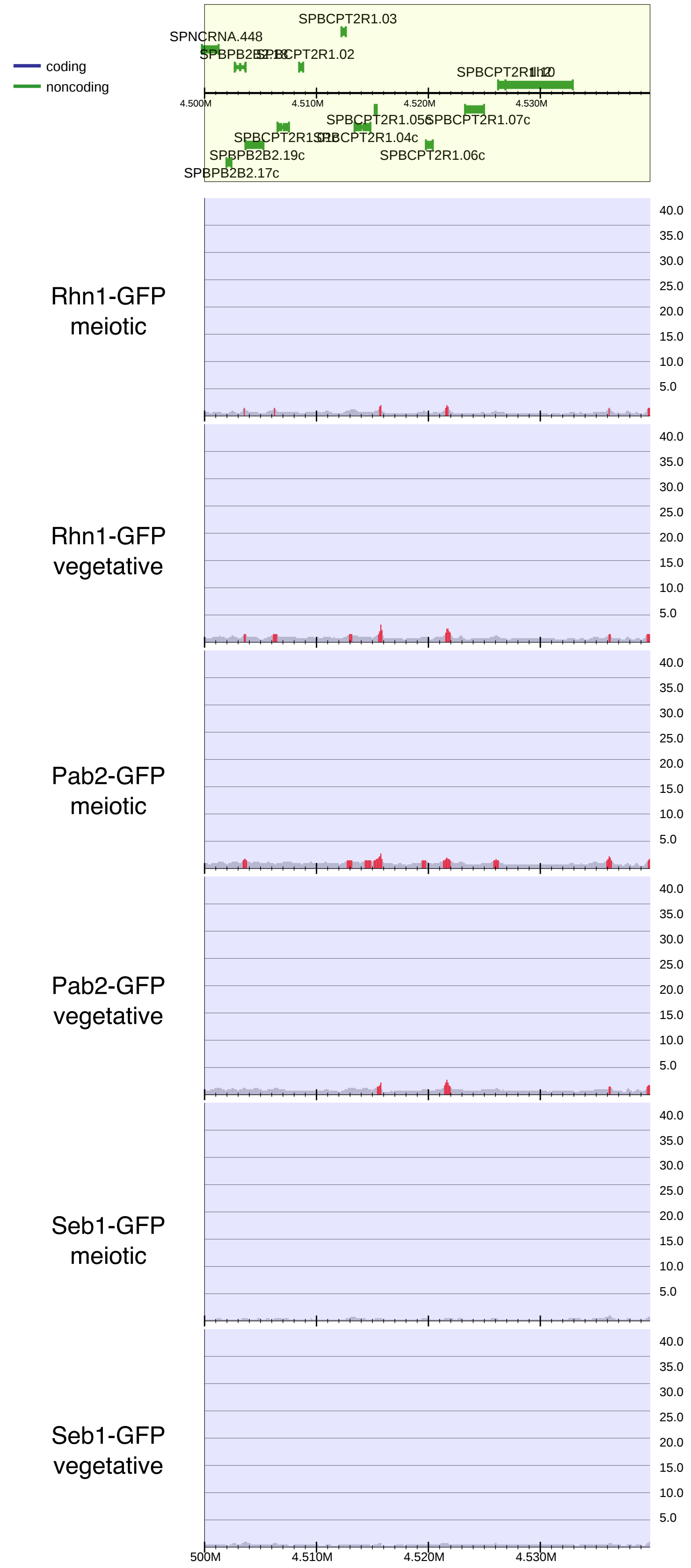
coding
noncoding
SPBCPT2R1.03
SPNCRNA.448
SPBCPT2R1.02
SPBCPT2R1.07c
SPBCPT2R1.04c
SPBCPT2R1.06c
SPBPB2B2.19c
SPBPB2B2.17c
4.500M
4.510M
4.520M
4.530M
Rhn1-GFP meiotic
Rhn1-GFP vegetative
Pab2-GFP meiotic
Pab2-GFP vegetative
Seb1-GFP meiotic
Seb1-GFP vegetative
40.0
35.0
30.0
25.0
20.0
15.0
10.0
5.0

III_1_1

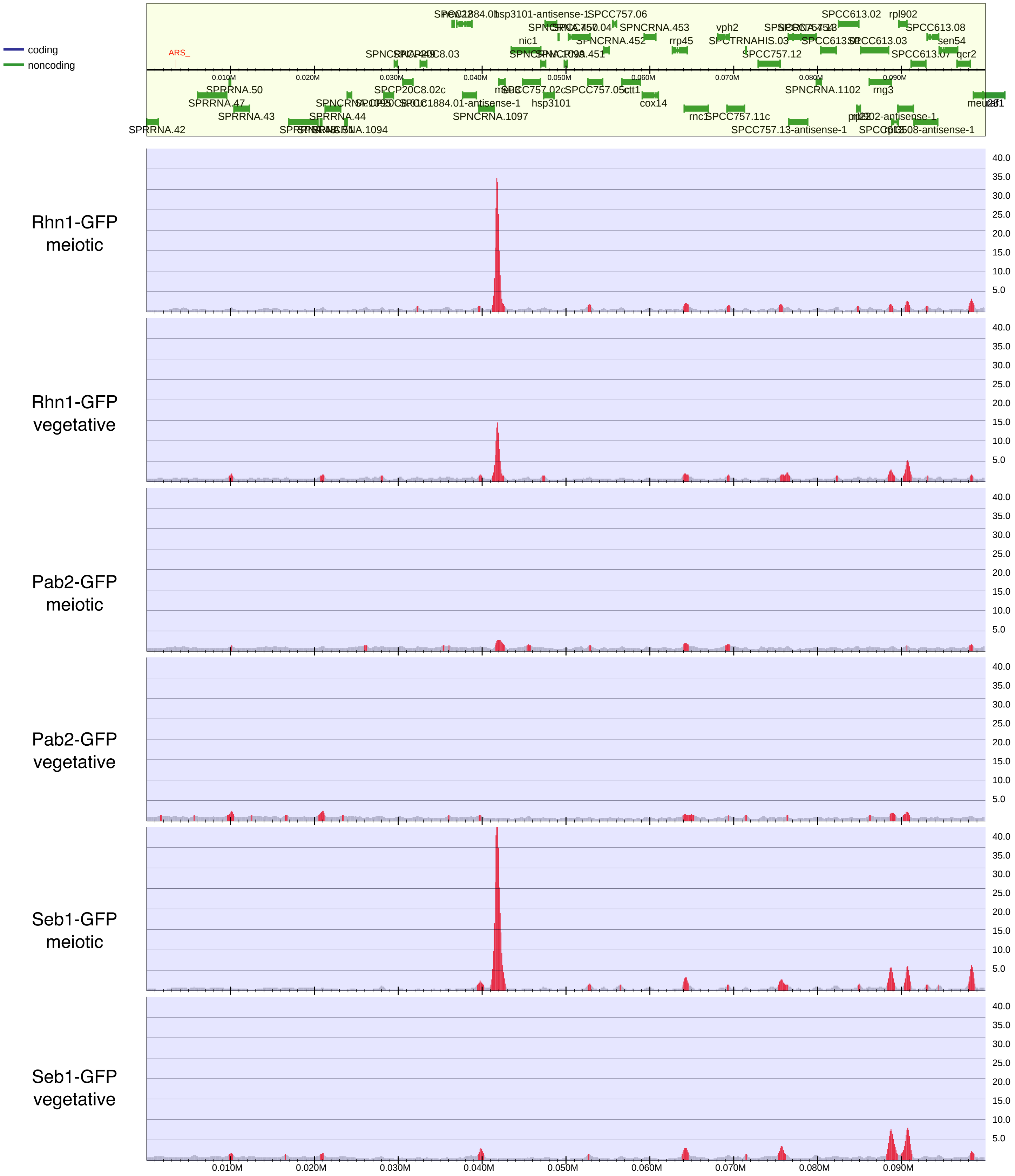
coding
noncoding
ARS_
SPRRNA.50
SPRRNA.47
SPRRNA.43
SPRRNA.42
SPRRNA.44
SPCP20C8.02c
SPCP20C8.03
SPNCRNA.1097
SPCC757.02c
SPCC757.05c
SPCC757.06
hsp3101-antisense-1
hsp3101
ctt1
nic1
SPNCRNA.452
SPNCRNA.453
SPNCRNA.451
cox14
rrp45
vph2
rnc1
SPCC757.11c
SPCC757.12
SPCC757.13-antisense-1
SPTRNAHIS.03
SPNCRNA.1102
SPCC613.02
SPCC613.03
SPCC613.07
SPCC613.08
rpl902
rng3
sen54
qcr2
0.010M
0.020M
0.030M
0.040M
0.050M
0.060M
0.070M
0.080M
0.090M
Rhn1-GFP meiotic
Rhn1-GFP vegetative
Pab2-GFP meiotic
Pab2-GFP vegetative
Seb1-GFP meiotic
Seb1-GFP vegetative
40.0
35.0
30.0
25.0
20.0
15.0
10.0
5.0

III_1_2

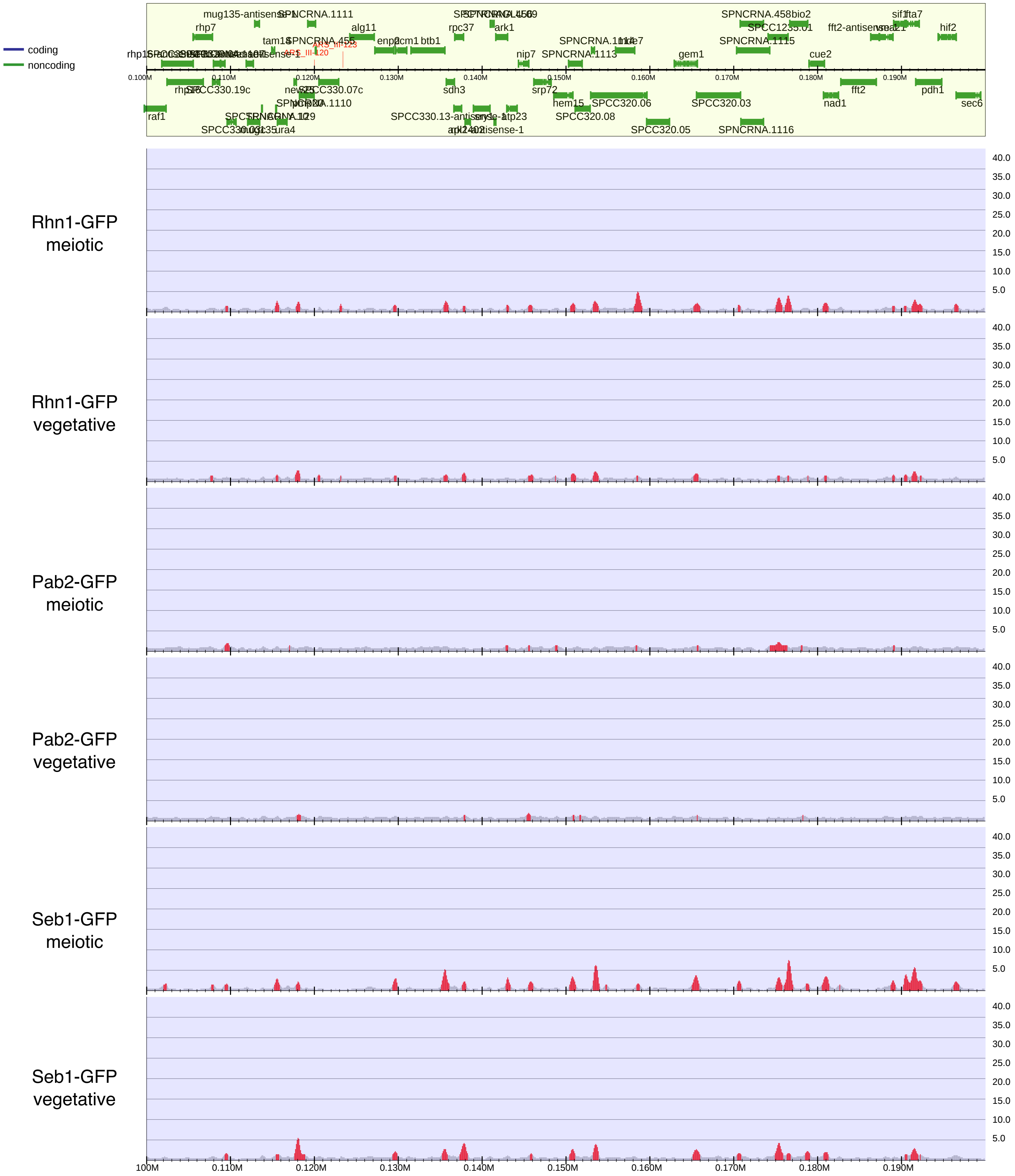
coding
noncoding
rhp7
alg11
tam14
SPNCRNA.455
enp2
cm1
btb1
rpc37
ark1
nip7
SPNCRNA.1113
gem1
SPNCRNA.1115
cue2
fft2-antisense-1
hif2
SPNCRNA.458
bio2
sif1
fta7
SPNCRNA.1111
SPCC330.19c
SPCC330.07c
SPNCRNA.1110
ura4
sdh3
srp72
hem15
SPCC320.06
SPCC320.08
SPCC320.05
SPCC320.03
SPNCRNA.1116
nad1
fft2
pdh1
sec6
raf1
atp23
0.100M
0.110M
0.120M
0.130M
0.140M
0.150M
0.160M
0.170M
0.180M
0.190M
Rhn1-GFP meiotic
Rhn1-GFP vegetative
Pab2-GFP meiotic
Pab2-GFP vegetative
Seb1-GFP meiotic
Seb1-GFP vegetative
40.0
35.0
30.0
25.0
20.0
15.0
10.0
5.0
100M
0.110M
0.120M
0.130M
0.140M
0.150M
0.160M
0.170M
0.180M
0.190M

III_1_3

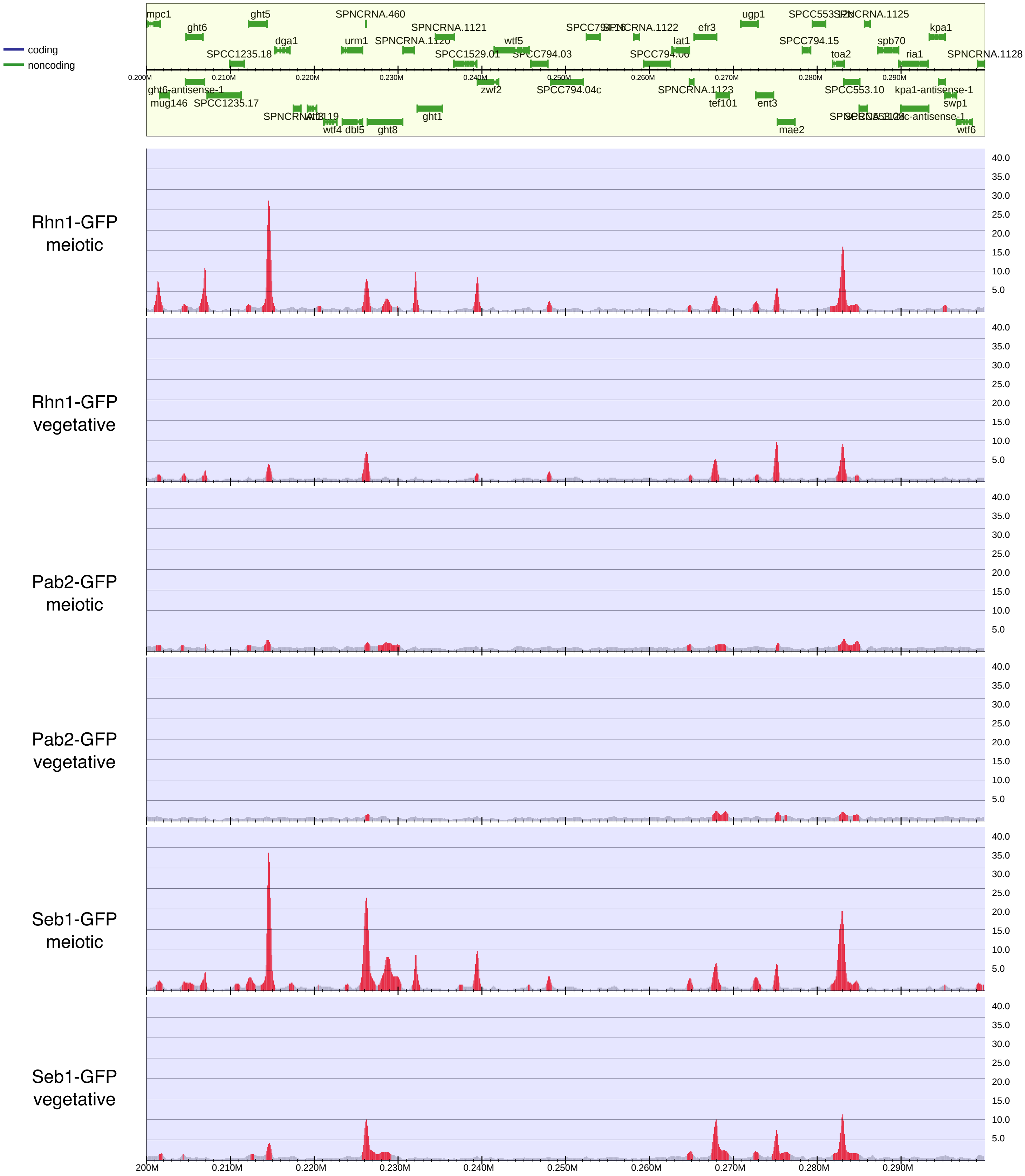
coding
noncoding
mpc1
ght6
ght5
SPNCRNA.460
SPNCRNA.1121
dga1
urm1
SPNCRNA.1120
wtf5
SPCC1235.18
SPCC1529.01
SPCC794.03
SPNCRNA.1122
efr3
lat1
ugp1
SPCC794.15
toa2
spb70
kpa1
ria1
SPNCRNA.1128
SPNCRNA.1125
ght6-antisense-1
mug146
SPCC1235.17
wtf4
dbl5
ght8
ght1
zwf2
SPCC794.04c
SPNCRNA.1123
tef101
ent3
mae2
SPCC553.10
kpa1-antisense-1
swp1
wtf6
0.200M
0.210M
0.220M
0.230M
0.240M
0.250M
0.260M
0.270M
0.280M
0.290M
Rhn1-GFP meiotic
Rhn1-GFP vegetative
Pab2-GFP meiotic
Pab2-GFP vegetative
Seb1-GFP meiotic
Seb1-GFP vegetative
40.0
35.0
30.0
25.0
20.0
15.0
10.0
5.0
200M
0.210M
0.220M
0.230M
0.240M
0.250M
0.260M
0.270M
0.280M
0.290M

III_1_4

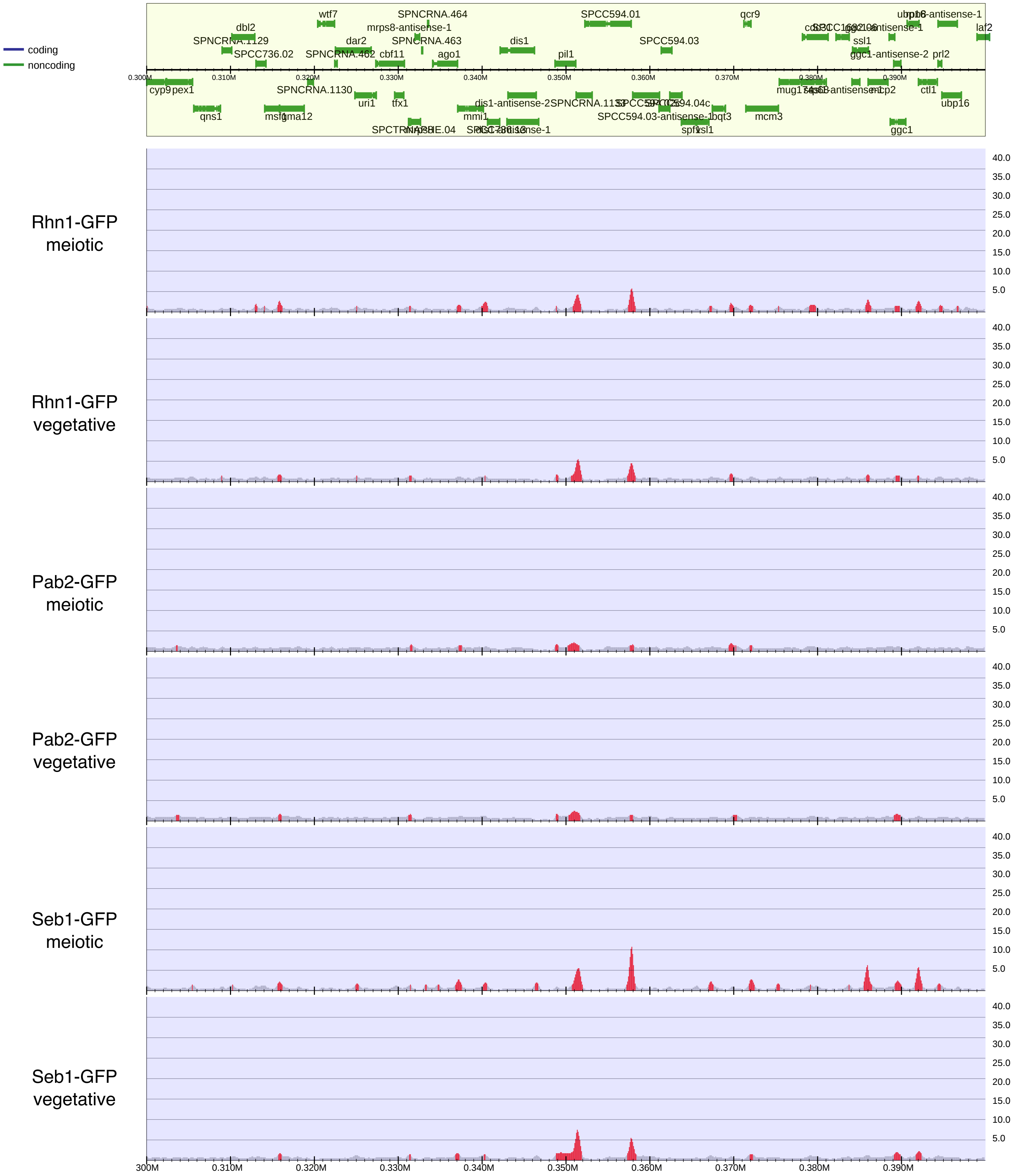
coding
noncoding
wtf7
SPNCRNA.464
SPCC594.01
qcr9
dbl2
mrps8-antisense-1
laf2
SPNCRNA.1129
dar2
SPNCRNA.463
dis1
SPCC594.03
ssl1
SPCC736.02
SPNCRNA.462
cbf11
ago1
pil1
ggc1-antisense-2
prl2
cyp9
pex1
SPNCRNA.1130
uri1
tfx1
ctl1
ubp16
qns1
mmi1
mcm3
ggc1
Rhn1-GFP meiotic
Rhn1-GFP vegetative
Pab2-GFP meiotic
Pab2-GFP vegetative
Seb1-GFP meiotic
Seb1-GFP vegetative
40.0
35.0
30.0
25.0
20.0
15.0
10.0
5.0
300M
0.310M
0.320M
0.330M
0.340M
0.350M
0.360M
0.370M
0.380M
0.390M

III_1_5

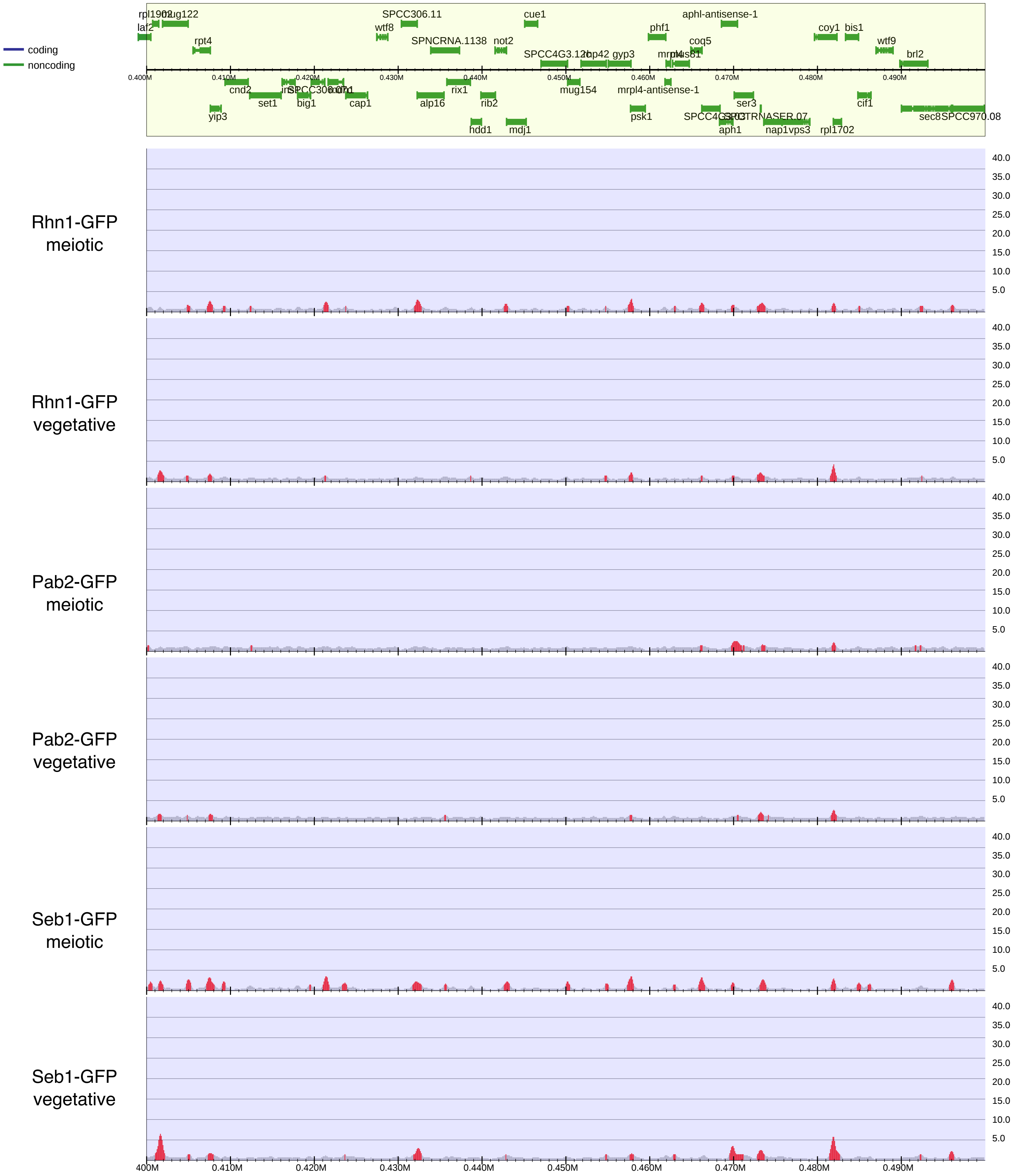
coding
noncoding
rpl1902
mug122
laf2
rpt4
SPCC306.11
wtf8
SPNCRNA.1138
not2
cue1
SPCC4G3.12c
gyp3
phf1
aphl-antisense-1
coq5
coy1
bis1
wtf9
brl2
0.400M
0.410M
0.420M
0.430M
0.440M
0.450M
0.460M
0.470M
0.480M
0.490M
cnd2
set1
big1
cap1
alp16
rix1
rib2
mug154
mrpl4-antisense-1
ser3
cif1
yip3
psk1
hdd1
mdj1
aph1
nap1
vps3
rpl1702
Rhn1-GFP meiotic
Rhn1-GFP vegetative
Pab2-GFP meiotic
Pab2-GFP vegetative
Seb1-GFP meiotic
Seb1-GFP vegetative
40.0
35.0
30.0
25.0
20.0
15.0
10.0
5.0
400M
0.410M
0.420M
0.430M
0.440M
0.450M
0.460M
0.470M
0.480M
0.490M

III_1_6

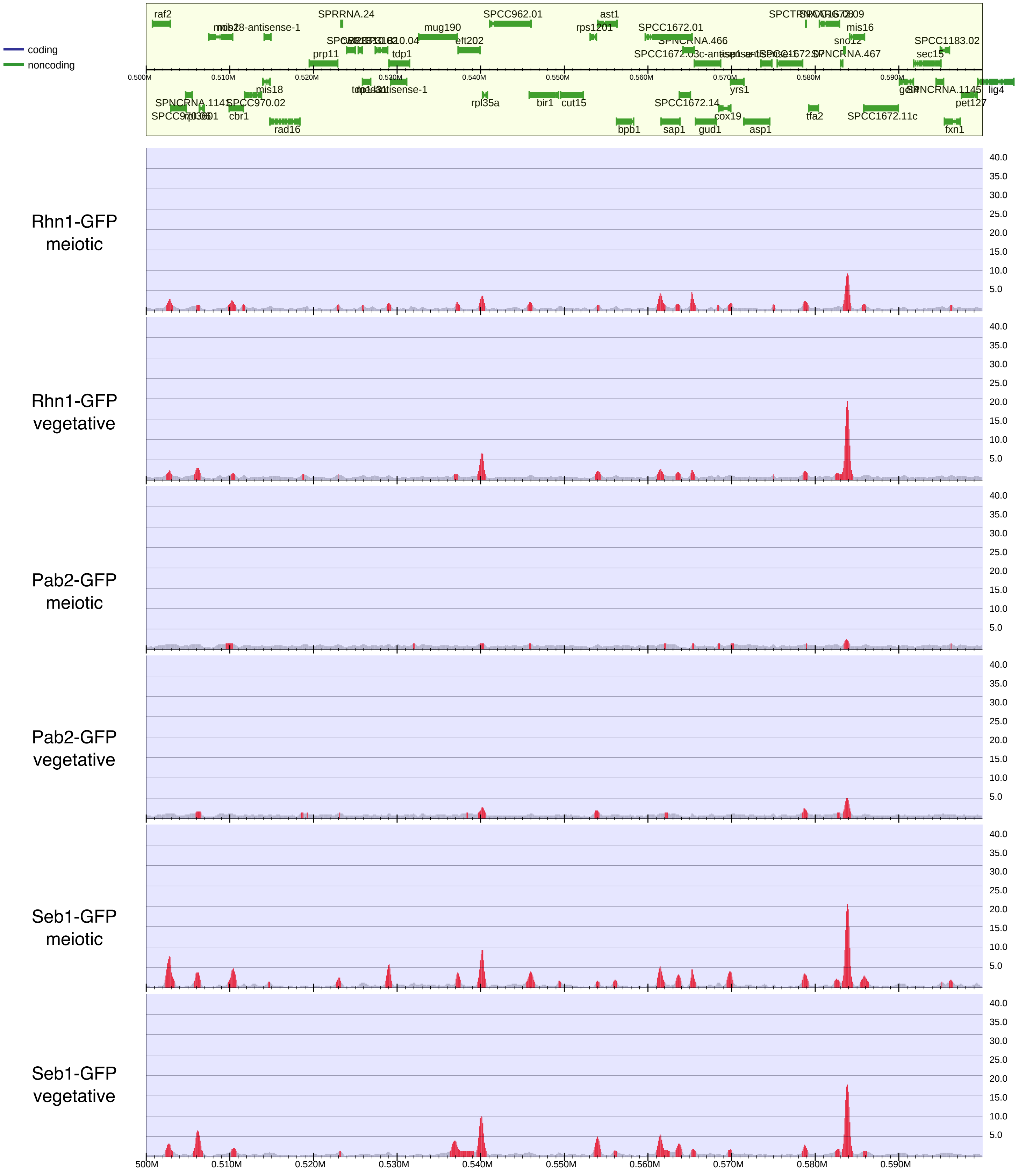

coding
noncoding
raf2
mis18
prp11
tdp1
mug190
eft202
SPCC962.01
ast1
rps1201
rpl35a
bir1
cut15
SPCC1672.01
SPNCRNA.466
SPCC1672.14
cox19
yrs1
bpb1
sap1
gud1
asp1
tfa2
snd12
mis16
SPCC1183.02
sec15
SPCC1672.11c
pet127
lig4
fxn1
cbr1
rad16
SPNCRNA.467
SPRRNA.24
Rhn1-GFP meiotic
Rhn1-GFP vegetative
Pab2-GFP meiotic
Pab2-GFP vegetative
Seb1-GFP meiotic
Seb1-GFP vegetative
40.0
35.0
30.0
25.0
20.0
15.0
10.0
5.0
500M
0.510M
0.520M
0.530M
0.540M
0.550M
0.560M
0.570M
0.580M
0.590M

III_1_7

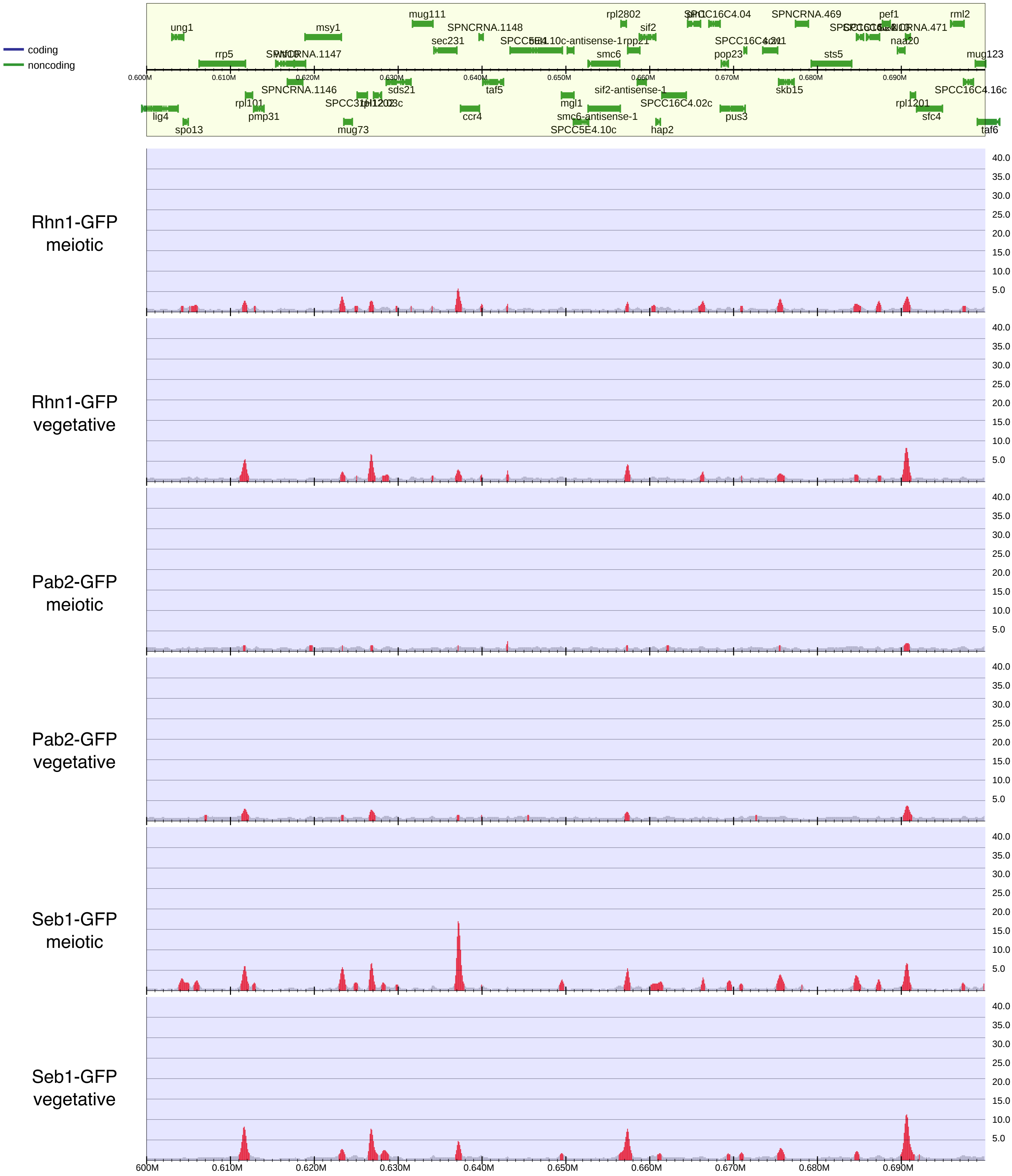

coding
noncoding
ung1
msy1
mug111
SPNCRNA.1148
sec231
rpl2802
sif2
SPCC16C4.04
SPNCRNA.469
pef1
rml2
naa20
smc6
pop23
sts5
mug123
rrp5
SPNCRNA.1147
0.600M
0.610M
0.620M
0.630M
0.640M
0.650M
0.660M
0.670M
0.680M
0.690M
SPNCRNA.1146
sds21
taf5
sif2-antisense-1
skb15
SPCC16C4.16c
rpl101
mgl1
SPCC16C4.02c
rpl1201
lig4
pmp31
ccr4
smc6-antisense-1
pus3
sfc4
spo13
mug73
SPCC5E4.10c
hap2
taf6
Rhn1-GFP meiotic
Rhn1-GFP vegetative
Pab2-GFP meiotic
Pab2-GFP vegetative
Seb1-GFP meiotic
Seb1-GFP vegetative
40.0
35.0
30.0
25.0
20.0
15.0
10.0
5.0
600M
0.610M
0.620M
0.630M
0.640M
0.650M
0.660M
0.670M
0.680M
0.690M

III_1_8

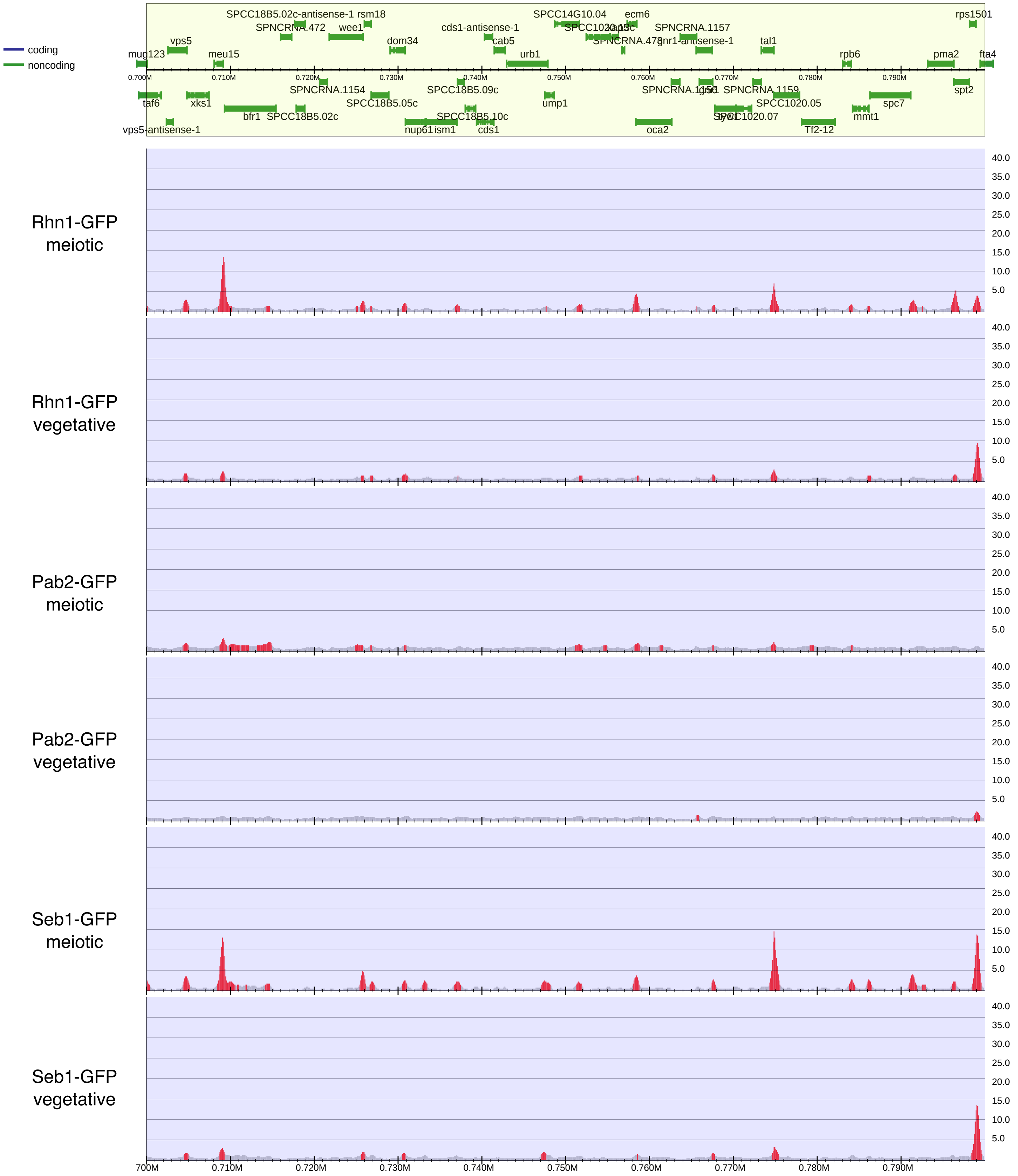

coding
noncoding
SPCC18B5.02c-antisense-1
rsm18
SPNCRNA.472
wee1
SPCC14G10.04
ecm6
cds1-antisense-1
SPNCRNA.1157
rps1501
vps5
dom34
cab5
tal1
mug123
meu15
urb1
rpb6
pma2
fta4
0.700M
0.710M
0.720M
0.730M
0.740M
0.750M
0.760M
0.770M
0.780M
0.790M
SPNCRNA.1154
SPCC18B5.09c
SPNCRNA.1159
spt2
taf6
xks1
SPCC18B5.05c
ump1
SPCC1020.05
spc7
bfr1
SPCC18B5.02c
SPCC18B5.10c
SPCC1020.07
mmt1
vps5-antisense-1
nup61
ism1
cds1
oca2
Tf2-12
Rhn1-GFP meiotic
Rhn1-GFP vegetative
Pab2-GFP meiotic
Pab2-GFP vegetative
Seb1-GFP meiotic
Seb1-GFP vegetative
40.0
35.0
30.0
25.0
20.0
15.0
10.0
5.0
700M
0.710M
0.720M
0.730M
0.740M
0.750M
0.760M
0.770M
0.780M
0.790M

III_1_9

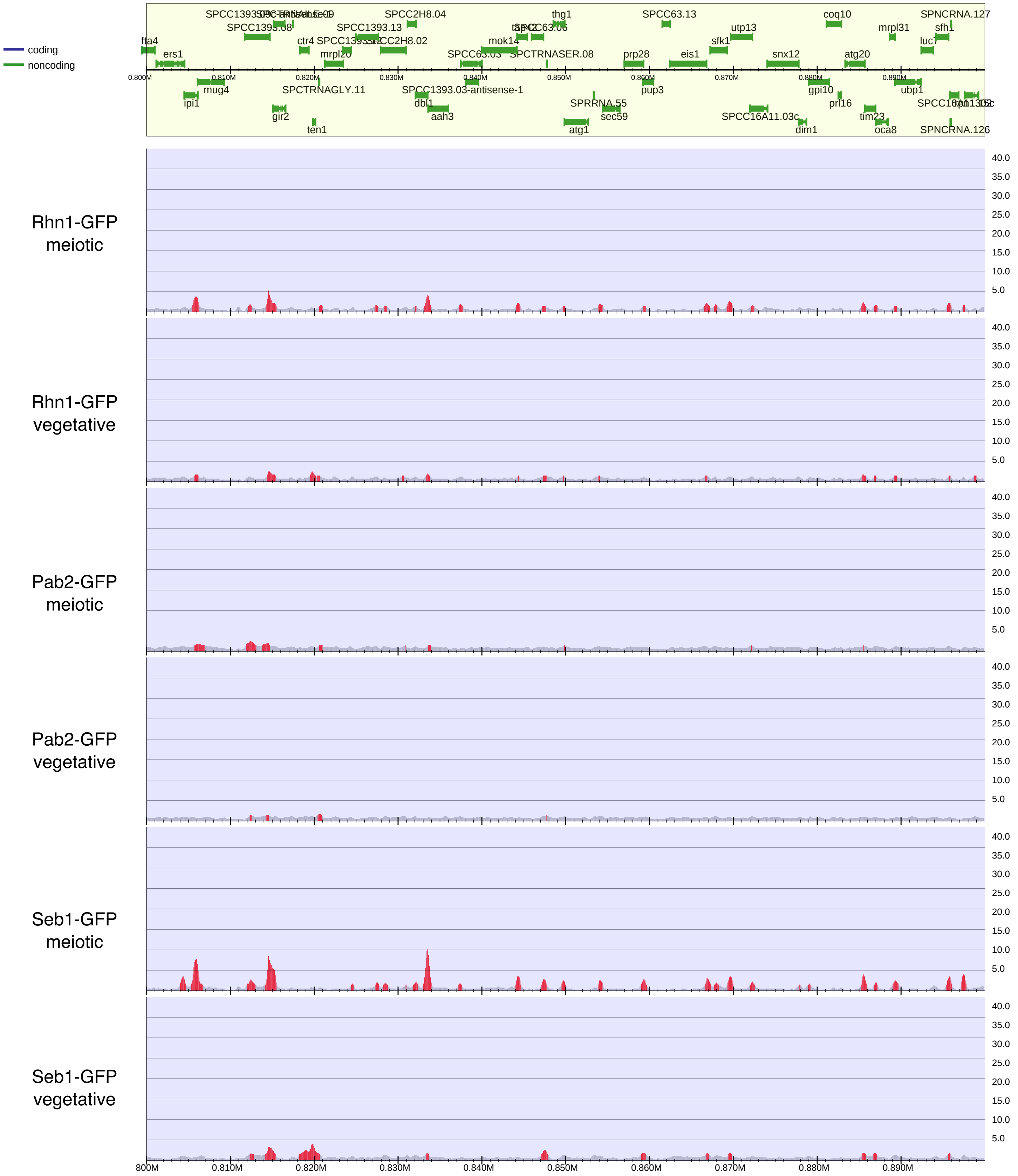

coding
noncoding
fta4
ers1
SPCC1393.08
ctr4
mrpl20
SPCC1393.13
SPCC2H8.04
SPCC2H8.02
SPCC63.03
mok14
SPCTRNASER.08
thg1
SPCC63.06
SPCC63.13
prp28
eis1
sfk1
utp13
snx12
coq10
atg20
mrpl31
luc7
sfh1
SPNCRNA.127
ipi1
mug4
gir2
ten1
SPCTRNAGLY.11
SPCC1393.03-antisense-1
dbl1
aah3
SPRRNA.55
sec59
atg1
pup3
SPCC16A11.03c
dim1
gpi10
prl16
tim23
oca8
ubp1
SPNCRNA.126
Rhn1-GFP meiotic
Rhn1-GFP vegetative
Pab2-GFP meiotic
Pab2-GFP vegetative
Seb1-GFP meiotic
Seb1-GFP vegetative
40.0
35.0
30.0
25.0
20.0
15.0
10.0
5.0
800M
0.810M
0.820M
0.830M
0.840M
0.850M
0.860M
0.870M
0.880M
0.890M

III_1_10

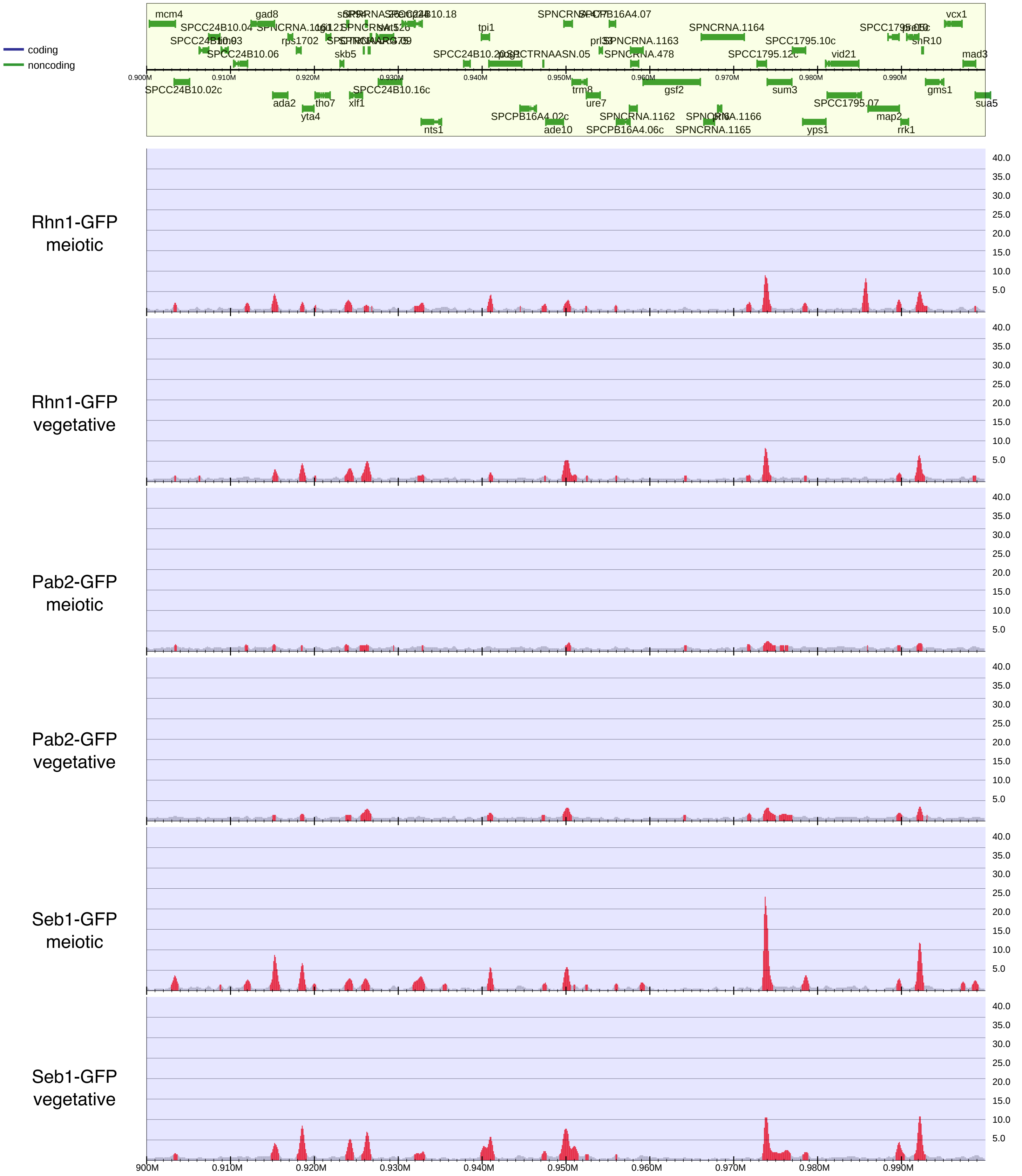

coding
noncoding
mcm4
gad8
SPCC24B10.04
SPCC24B10.06
SPCC24B10.02c
SPCC24B10.16c
SPCC24B10.18
ada2
tho7
xlf1
yta4
rps1702
skb5
nts1
tpi1
SPCPB16A4.02c
SPCPB16A4.06c
SPCPB16A4.07
ade10
trm8
ure7
prl33
SPNCRNA.1163
SPNCRNA.478
SPNCRNA.1162
SPNCRNA.1164
SPNCRNA.1165
gsf2
sum3
SPCC1795.10c
SPCC1795.12c
SPCC1795.07
vid21
yps1
map2
rrk1
snR10
gms1
vcx1
mad3
sua5
Rhn1-GFP
meiotic
Rhn1-GFP
vegetative
Pab2-GFP
meiotic
Pab2-GFP
vegetative
Seb1-GFP
meiotic
Seb1-GFP
vegetative
40.0
35.0
30.0
25.0
20.0
15.0
10.0
5.0
900M
0.910M
0.920M
0.930M
0.940M
0.950M
0.960M
0.970M
0.980M
0.990M

III_1_11

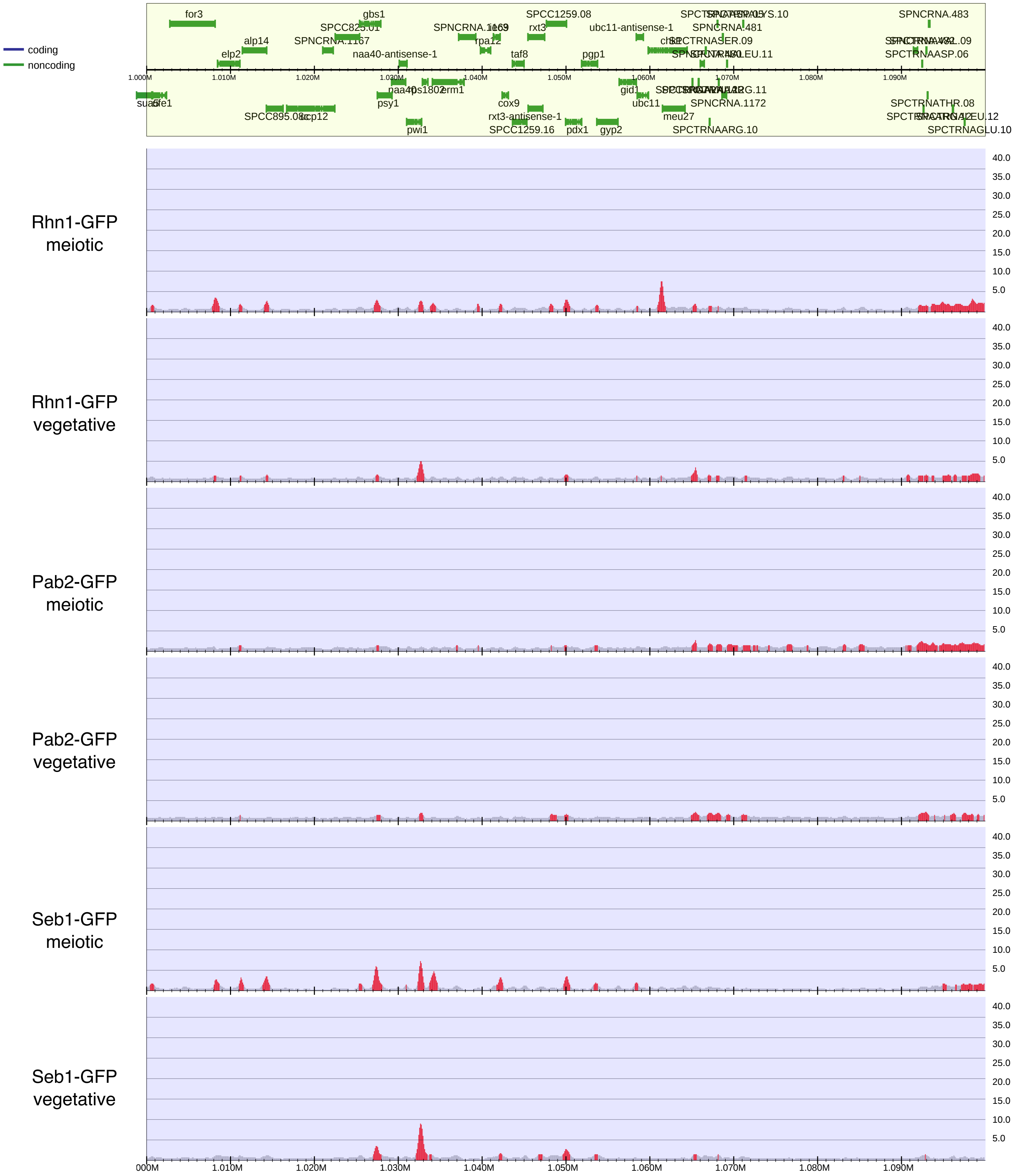
coding
noncoding
for3
gbs1
SPCC1259.08
SPCC825.01
SPNCRNA.1169
rxt3
ubc11-antisense-1
SPNCRNA.481
alp14
SPNCRNA.1167
rpa12
elp2
naa40-antisense-1
taf8
pgp1
SPNCRNA.483
SPCTRNAASP.06
1.000M
1.010M
1.020M
1.030M
1.040M
1.050M
1.060M
1.070M
1.080M
1.090M
naa40
psy1
cox9
rxt3-antisense-1
pwi1
SPCC1259.16
pdx1
gyp2
gid1
ubc11
meu27
SPNCRNA.1172
SPCTRNAARG.10
SPCTRNATHR.08
SPCTRNAGLU.10
SPCC895.08c
Rhn1-GFP meiotic
Rhn1-GFP vegetative
Pab2-GFP meiotic
Pab2-GFP vegetative
Seb1-GFP meiotic
Seb1-GFP vegetative
40.0
35.0
30.0
25.0
20.0
15.0
10.0
5.0
000M
1.010M
1.020M
1.030M
1.040M
1.050M
1.060M
1.070M
1.080M
1.090M

III_1_12

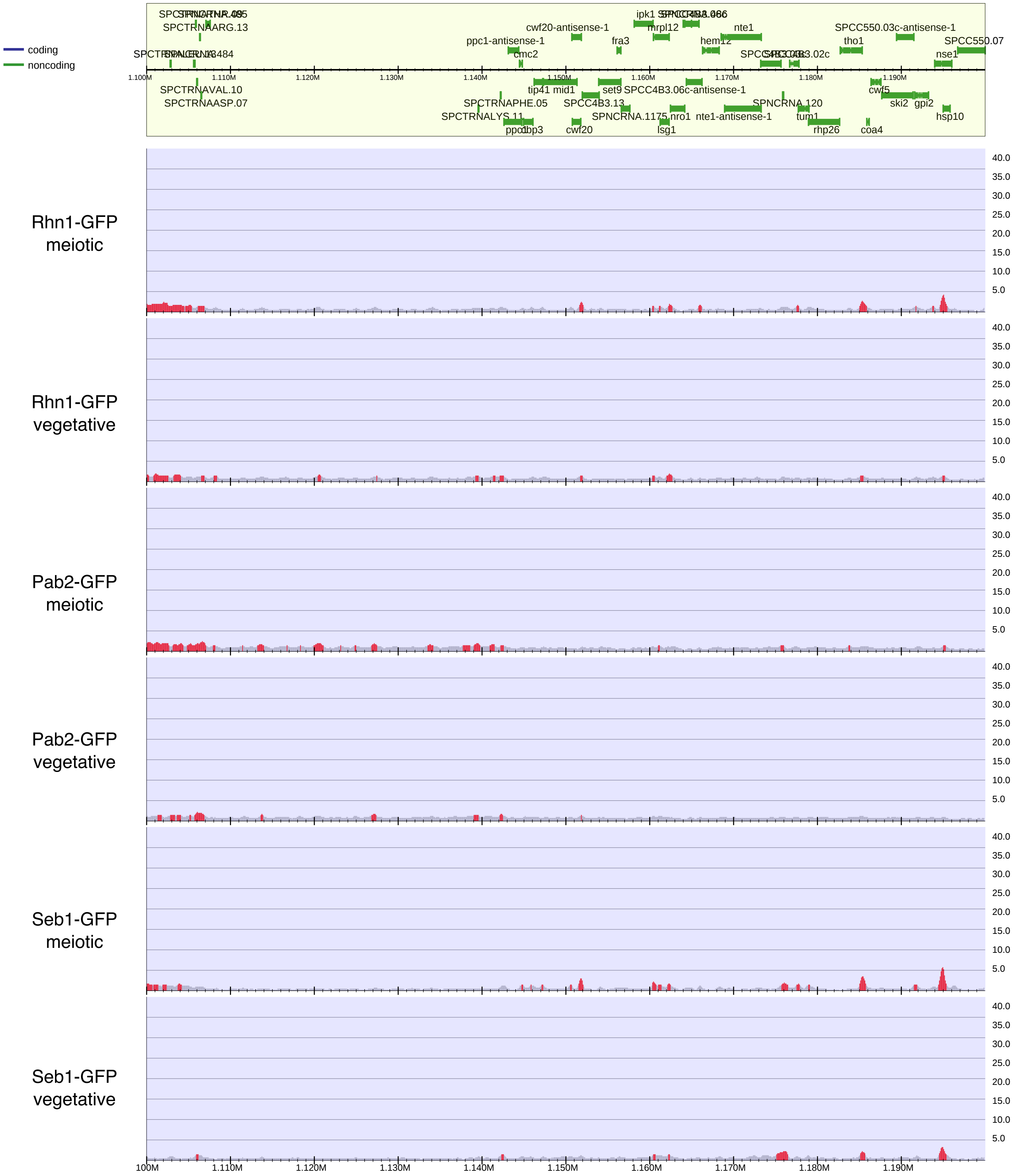
coding
noncoding
SPCTRNAARG.13
cwf20-antisense-1
ipk1
mrpl12
nte1
SPCC550.03c-antisense-1
ppc1-antisense-1
fra3
hem12
tho1
SPCC550.07
cmc2
nse1
1.100M
1.110M
1.120M
1.130M
1.140M
1.150M
1.160M
1.170M
1.180M
1.190M
SPCTRNAVAL.10
tip41
mid1
set9
SPCC4B3.06c-antisense-1
cwf5
SPCTRNAASP.07
SPCTRNAPHE.05
SPCC4B3.13
SPNCRNA.120
ski2
gpi2
SPNCRNA.1175
nro1
nte1-antisense-1
tum1
hsp10
cwf20
lsg1
rhp26
coa4
Rhn1-GFP meiotic
Rhn1-GFP vegetative
Pab2-GFP meiotic
Pab2-GFP vegetative
Seb1-GFP meiotic
Seb1-GFP vegetative
40.0
35.0
30.0
25.0
20.0
15.0
10.0
5.0
100M
1.110M
1.120M
1.130M
1.140M
1.150M
1.160M
1.170M
1.180M
1.190M

III_1_13

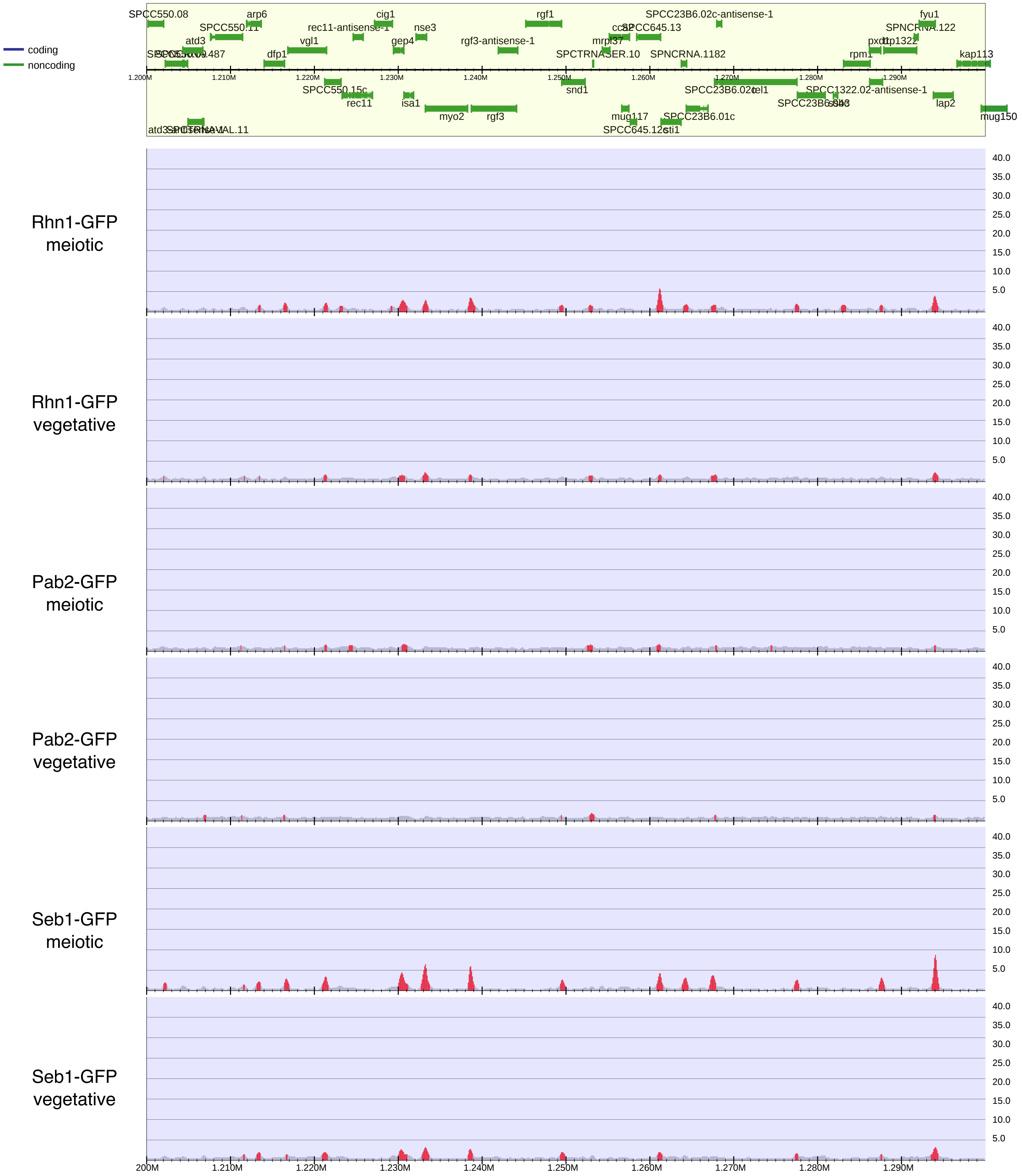
coding
noncoding
Rhn1-GFP meiotic
Rhn1-GFP vegetative
Pab2-GFP meiotic
Pab2-GFP vegetative
Seb1-GFP meiotic
Seb1-GFP vegetative
40.0
35.0
30.0
25.0
20.0
15.0
10.0
5.0
200M
1.210M
1.220M
1.230M
1.240M
1.250M
1.260M
1.270M
1.280M
1.290M

III_1_14

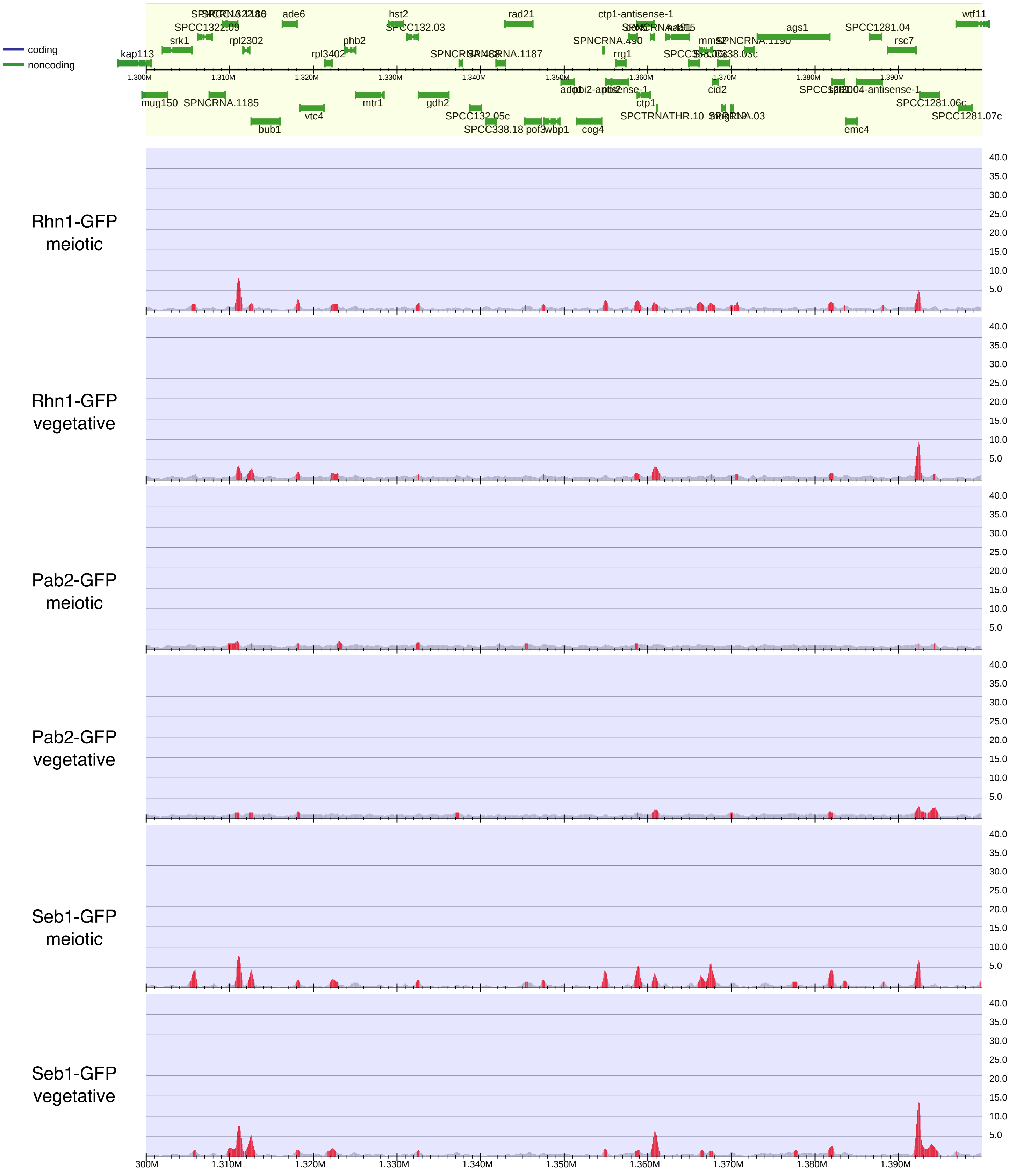
coding
noncoding
kap113
srk1
SPCC1322.09
ade6
rpl2302
rpl3402
phb2
hst2
SPCC132.03
rad21
SPNCRNA.1187
ctp1-antisense-1
SPNCRNA.490
rrg1
ags1
SPCC1281.04
rsc7
wtf11
mug150
SPNCRNA.1185
bub1
vtc4
mtr1
gdh2
SPCC132.05c
SPCC338.18
cog4
ctp1
SPCTRNATHR.10
cid2
emc4
SPCC1281.06c
SPCC1281.07c
Rhn1-GFP
meiotic
Rhn1-GFP
vegetative
Pab2-GFP
meiotic
Pab2-GFP
vegetative
Seb1-GFP
meiotic
Seb1-GFP
vegetative
40.0
35.0
30.0
25.0
20.0
15.0
10.0
5.0
300M
1.310M
1.320M
1.330M
1.340M
1.350M
1.360M
1.370M
1.380M
1.390M

III_1_15

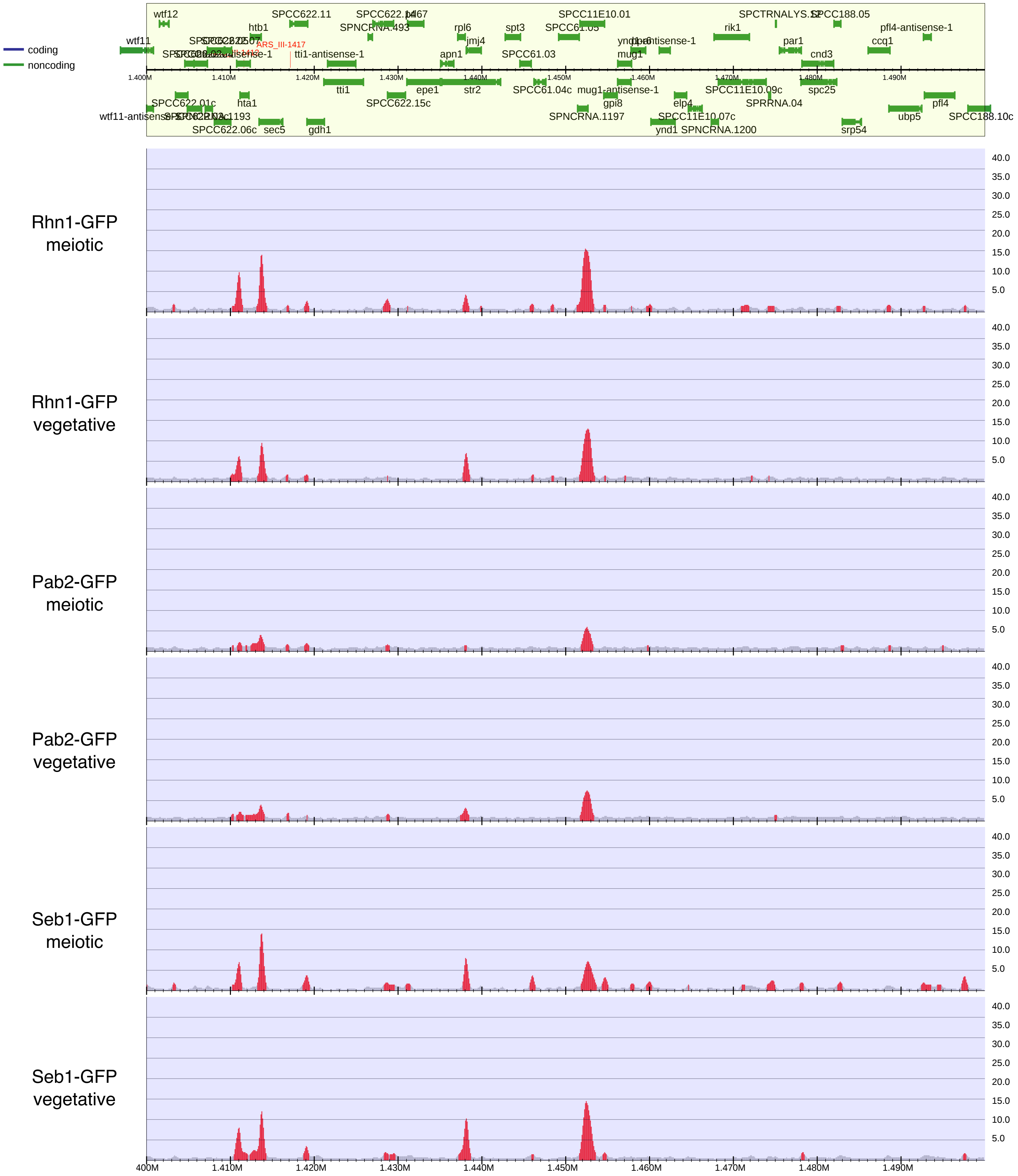

coding
noncoding
Rhn1-GFP meiotic
Rhn1-GFP vegetative
Pab2-GFP meiotic
Pab2-GFP vegetative
Seb1-GFP meiotic
Seb1-GFP vegetative
400M
1.410M
1.420M
1.430M
1.440M
1.450M
1.460M
1.470M
1.480M
1.490M

III_1_16

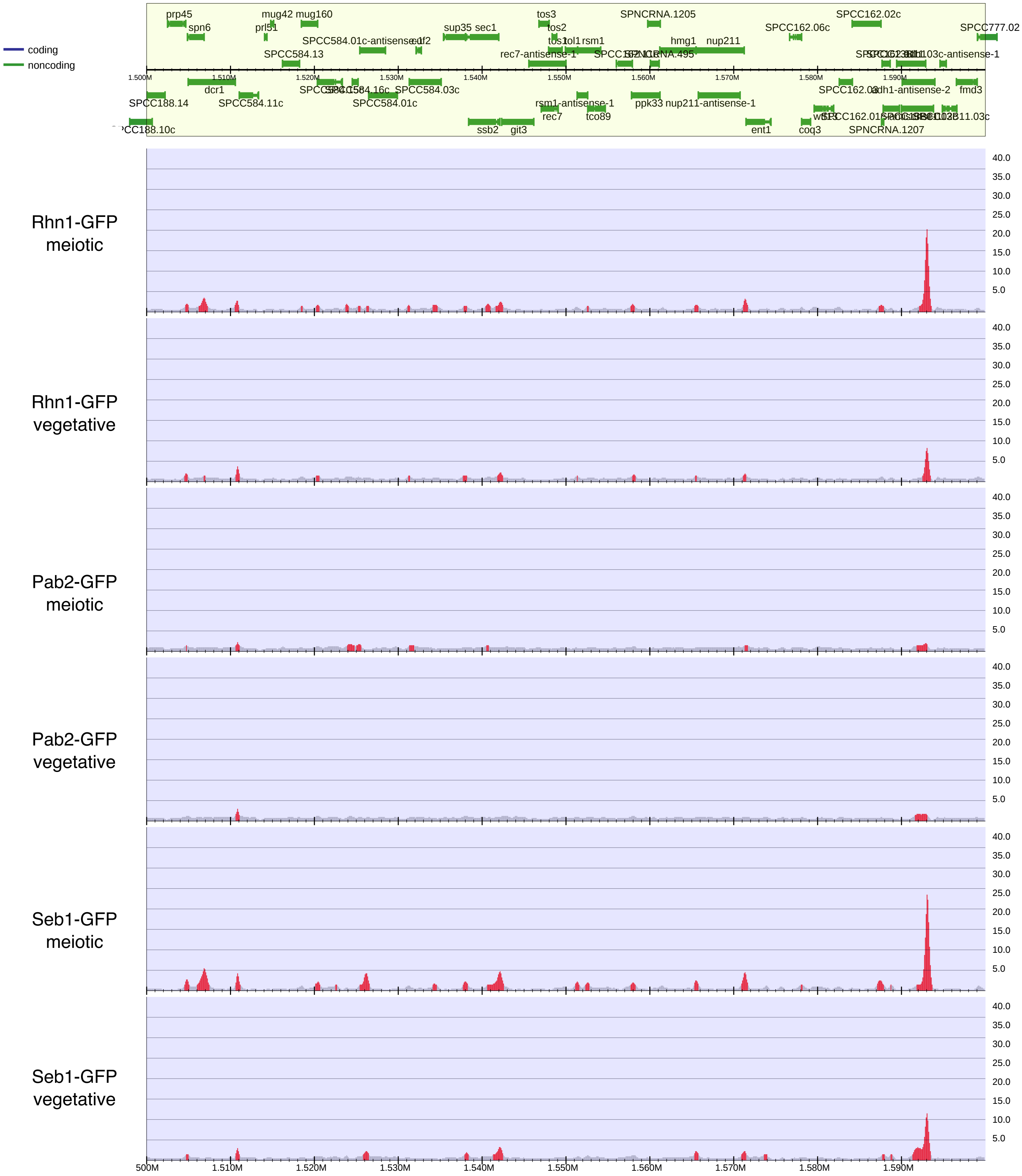
coding
noncoding
prp45
spn6
mug42 mug160
prl51
SPCC584.01c-antisense-1
eif2
SPCC584.13
sup35 sec1
tos3
tos2
tos1
tol1 rsm1
rec7-antisense-1
SPNCRNA.1205
SPNCRNA.495
hmg1
nup211
SPCC162.06c
SPCC162.02c
SPCC777.02
1.500M
1.510M
1.520M
1.530M
1.540M
1.550M
1.560M
1.570M
1.580M
1.590M
dcr1
SPCC584.16c
SPCC584.03c
SPCC188.14
SPCC584.11c
SPCC584.01c
rsm1-antisense-1
rec7
tco89
ppk33
nup211-antisense-1
SPCC162.03
adh1-antisense-2
fmd3
ssb2
git3
ent1
coq3
SPNCRNA.1207
Rhn1-GFP meiotic
Rhn1-GFP vegetative
Pab2-GFP meiotic
Pab2-GFP vegetative
Seb1-GFP meiotic
Seb1-GFP vegetative
40.0
35.0
30.0
25.0
20.0
15.0
10.0
5.0
500M
1.510M
1.520M
1.530M
1.540M
1.550M
1.560M
1.570M
1.580M
1.590M

III_1_17

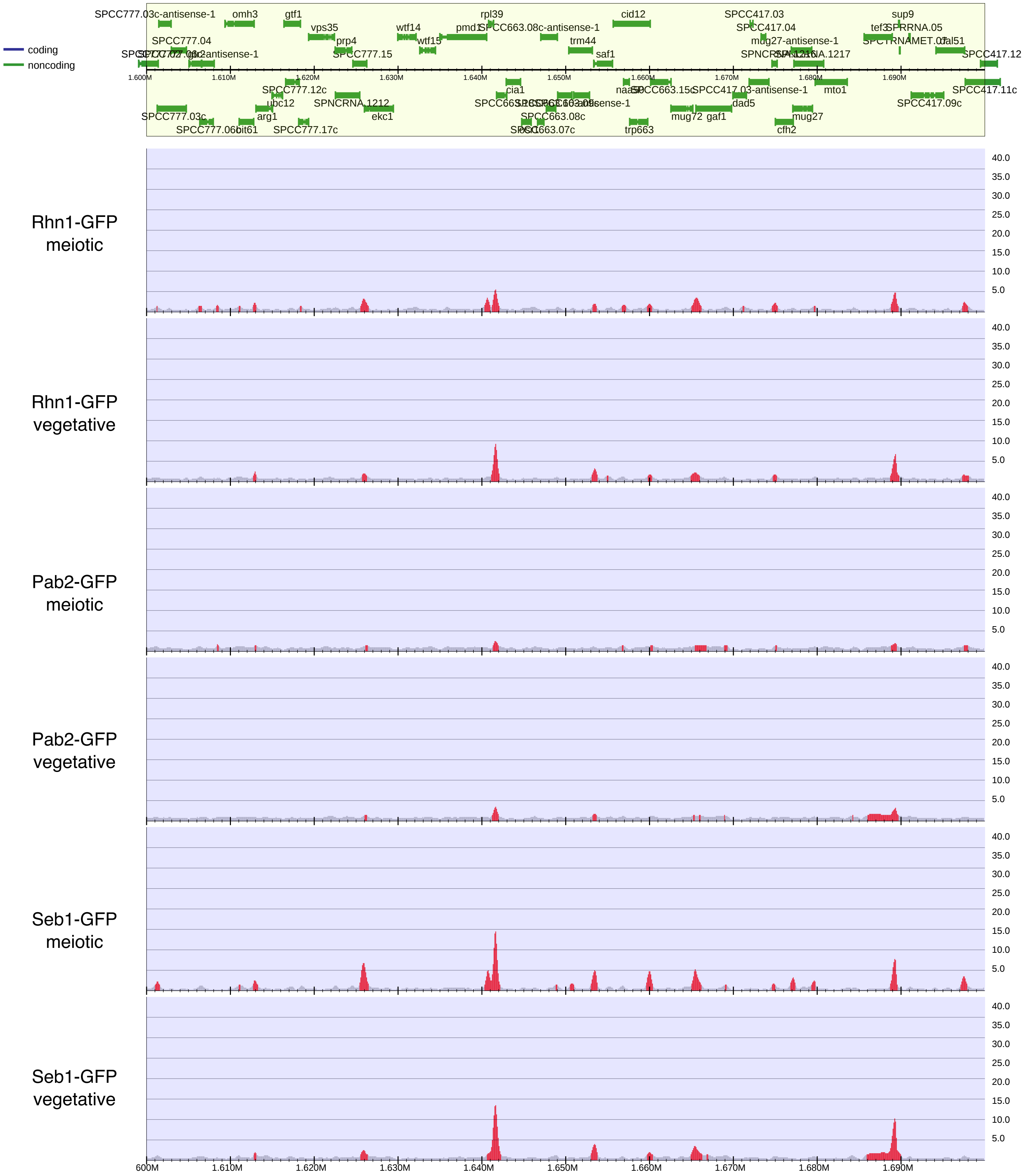

coding
noncoding
Rhn1-GFP meiotic
Rhn1-GFP vegetative
Pab2-GFP meiotic
Pab2-GFP vegetative
Seb1-GFP meiotic
Seb1-GFP vegetative
600M
1.610M
1.620M
1.630M
1.640M
1.650M
1.660M
1.670M
1.680M
1.690M

III_1_18

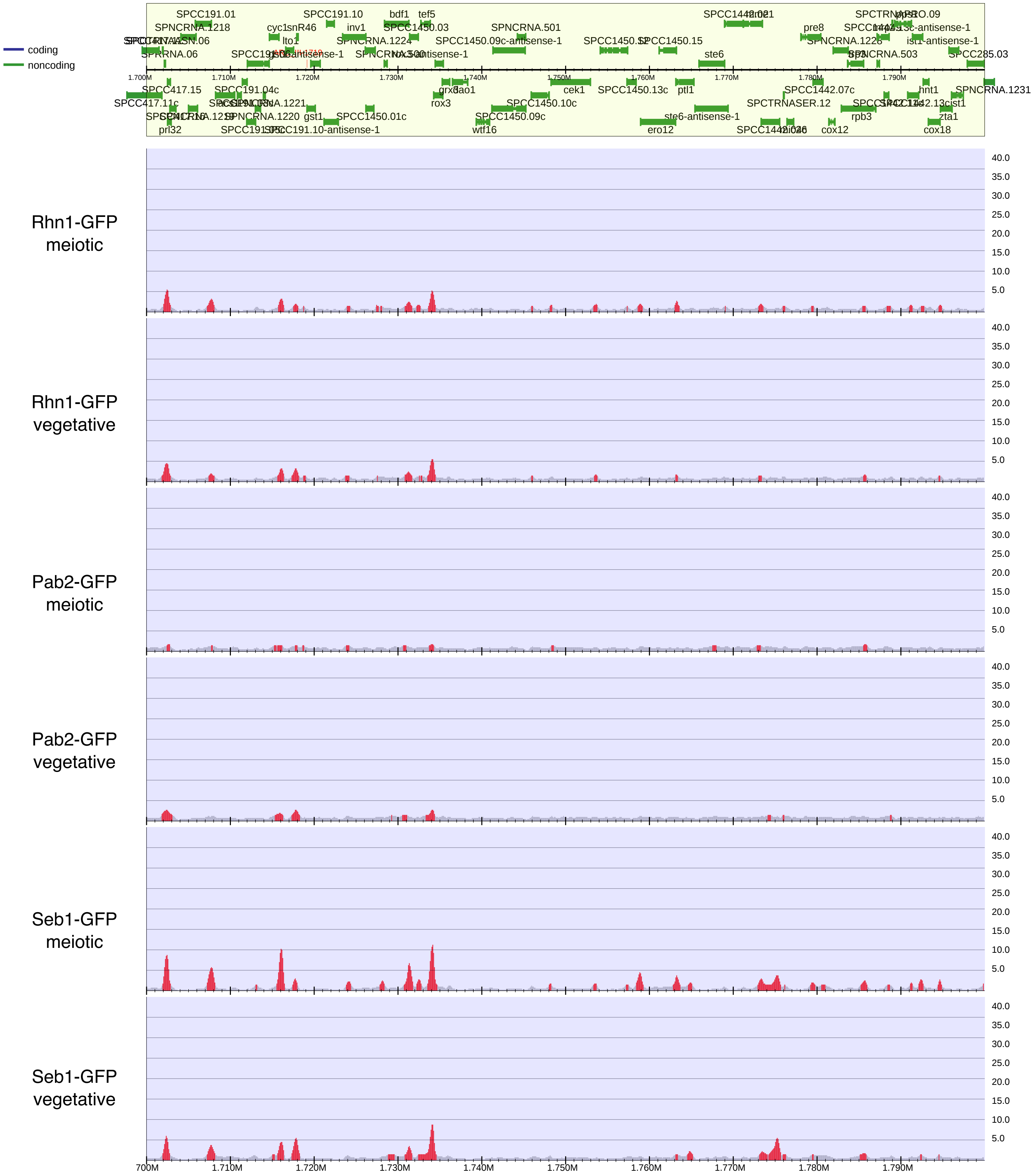

coding
noncoding
Rhn1-GFP meiotic
Rhn1-GFP vegetative
Pab2-GFP meiotic
Pab2-GFP vegetative
Seb1-GFP meiotic
Seb1-GFP vegetative
40.0
35.0
30.0
25.0
20.0
15.0
10.0
5.0
700M
1.710M
1.720M
1.730M
1.740M
1.750M
1.760M
1.770M
1.780M
1.790M

III_1_19

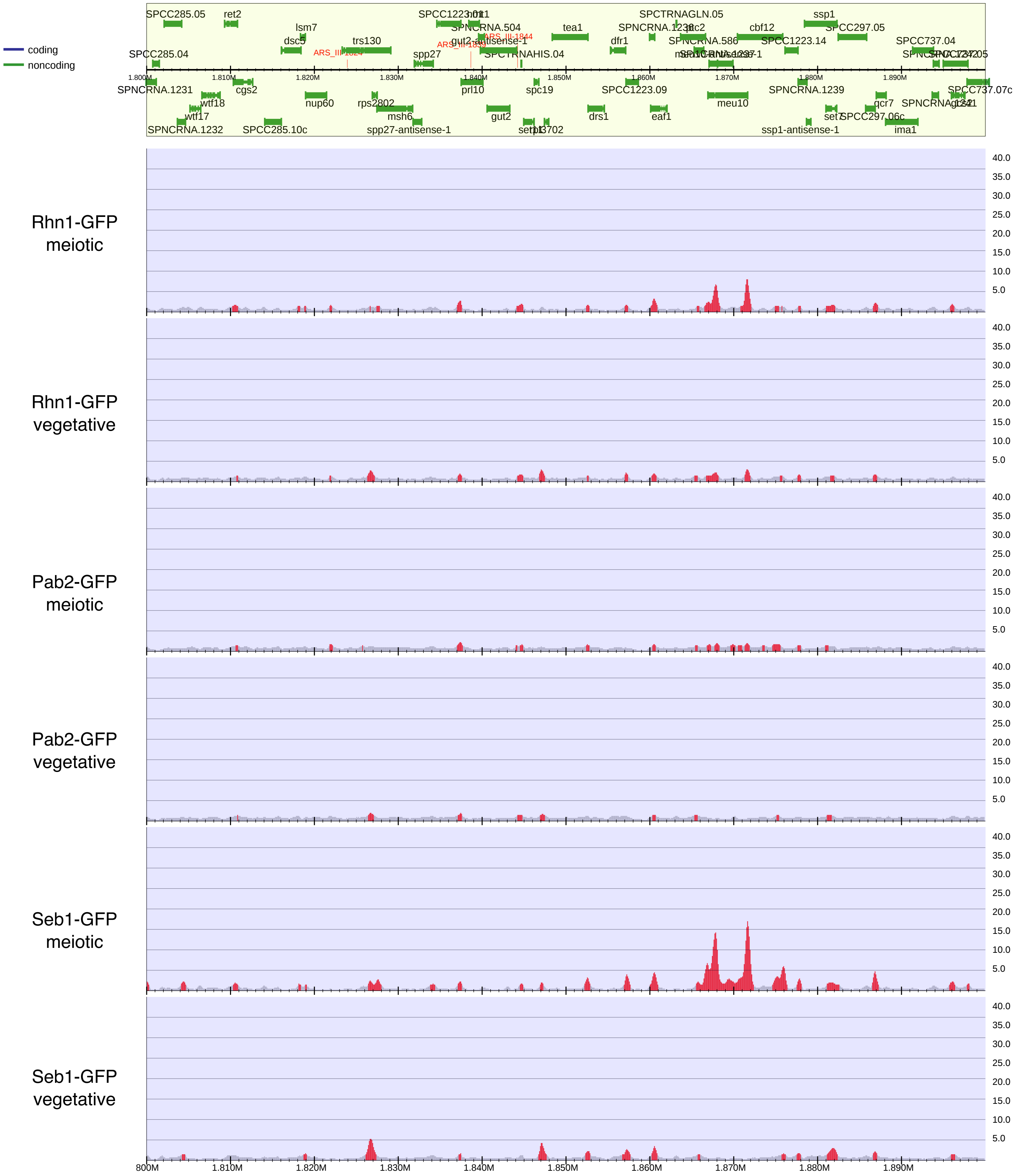

coding
noncoding
SPCC285.05
ret2
lsm7
dsc5
trs130
SPCC285.04
spp27
ARS_III-1824
tea1
dfr1
ssp1
SPCC297.05
cbf12
SPCC1223.14
SPCC737.04
SPCTRNAGLN.05
SPCTRNAHIS.04
1.800M
1.810M
1.820M
1.830M
1.840M
1.850M
1.860M
1.870M
1.880M
1.890M
SPNCRNA.1231
cgs2
wtf18
wtf17
SPNCRNA.1232
SPCC285.10c
nup60
rps2802
msh6
spp27-antisense-1
prl10
gut2
spc19
SPCC1223.09
drs1
eaf1
meu10
SPNCRNA.1239
ssp1-antisense-1
qcr7
ima1
SPCC737.07c
Rhn1-GFP meiotic
Rhn1-GFP vegetative
Pab2-GFP meiotic
Pab2-GFP vegetative
Seb1-GFP meiotic
Seb1-GFP vegetative
40.0
35.0
30.0
25.0
20.0
15.0
10.0
5.0
800M

III_1_20

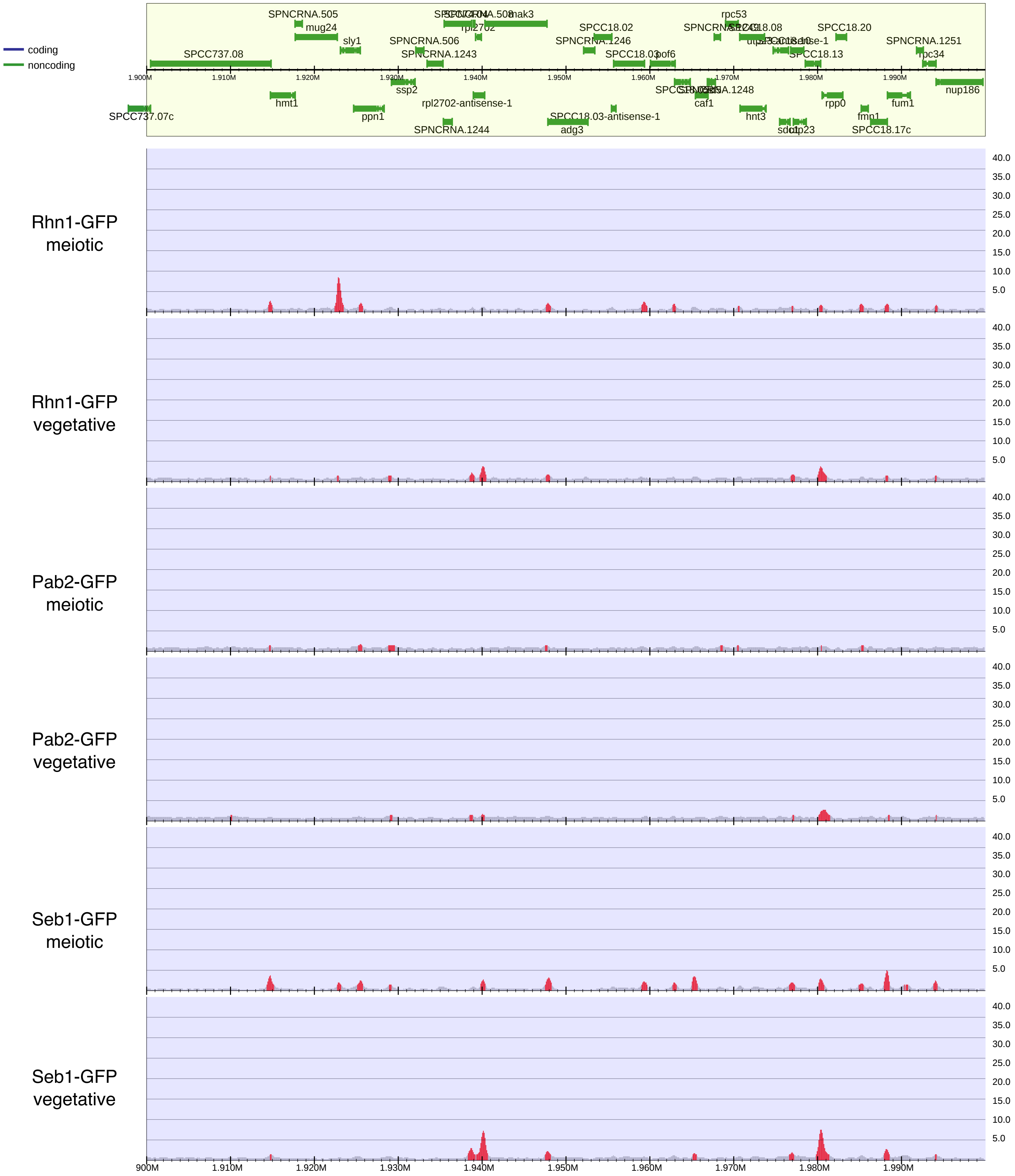
coding
noncoding
SPNCRNA.505
mug24
sly1
SPNCRNA.506
SPNCRNA.1243
rpl2702
mak3
SPCC18.02
SPNCRNA.1246
SPCC18.03
pof6
rpc53
SPCC18.08
SPCC18.20
SPCC18.13
SPNCRNA.1251
rpc34
SPCC737.08
SPCC737.07c
hmt1
ppn1
ssp2
rpl2702-antisense-1
SPNCRNA.1244
SPCC18.03-antisense-1
adg3
caf1
SPNCRNA.1248
hnt3
rpp0
fmn1
fum1
nup186
SPCC18.17c
Rhn1-GFP meiotic
Rhn1-GFP vegetative
Pab2-GFP meiotic
Pab2-GFP vegetative
Seb1-GFP meiotic
Seb1-GFP vegetative
40.0
35.0
30.0
25.0
20.0
15.0
10.0
5.0
900M
1.910M
1.920M
1.930M
1.940M
1.950M
1.960M
1.970M
1.980M
1.990M

III_1_21

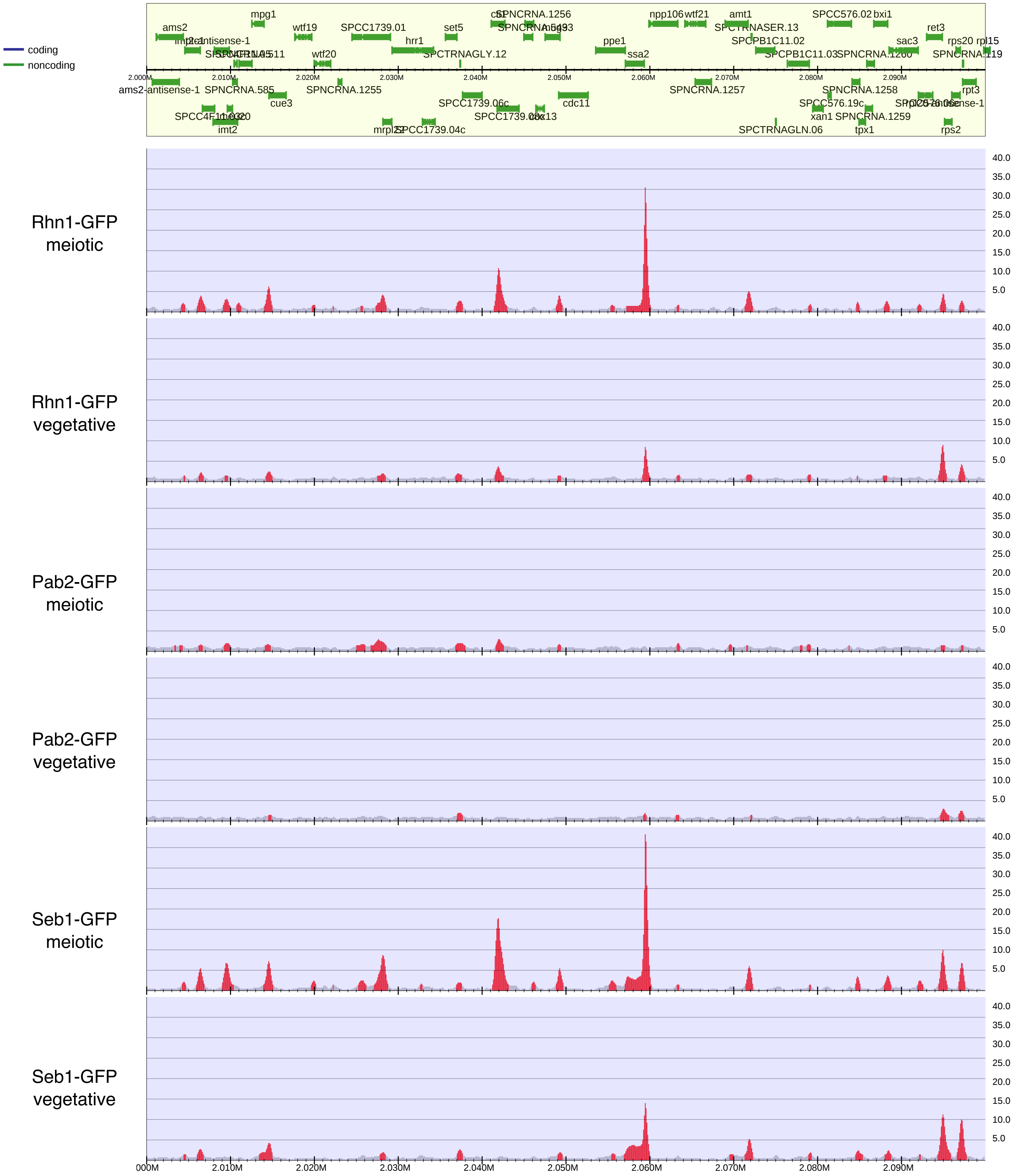
coding
noncoding
Rhn1-GFP meiotic
Rhn1-GFP vegetative
Pab2-GFP meiotic
Pab2-GFP vegetative
Seb1-GFP meiotic
Seb1-GFP vegetative
2.010M
2.020M
2.030M
2.040M
2.050M
2.060M
2.070M
2.080M
2.090M

III_1_22

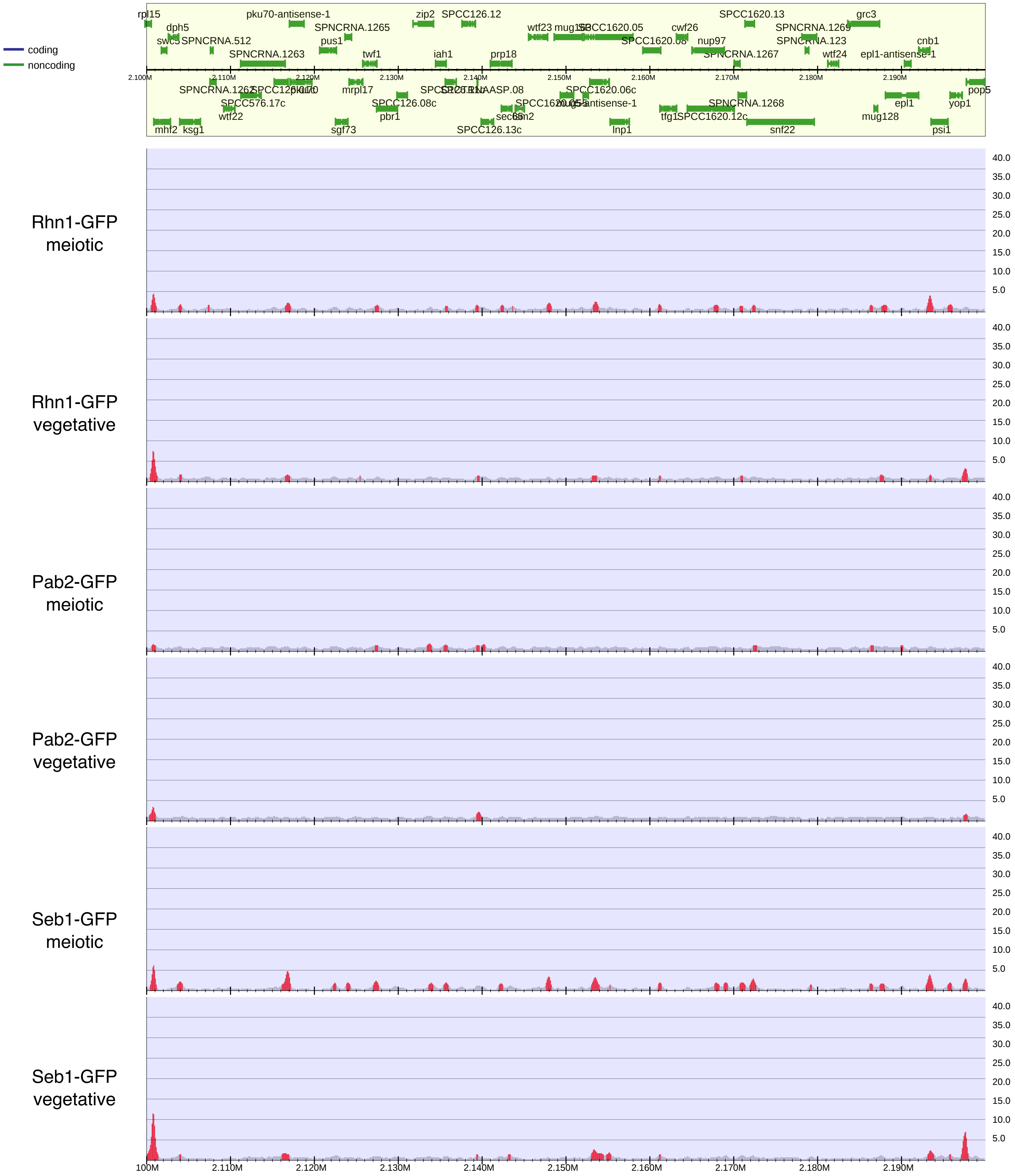
coding
noncoding
Rhn1-GFP meiotic
Rhn1-GFP vegetative
Pab2-GFP meiotic
Pab2-GFP vegetative
Seb1-GFP meiotic
Seb1-GFP vegetative
40.0
35.0
30.0
25.0
20.0
15.0
10.0
5.0
100M
2.110M
2.120M
2.130M
2.140M
2.150M
2.160M
2.170M
2.180M
2.190M

III_1_23

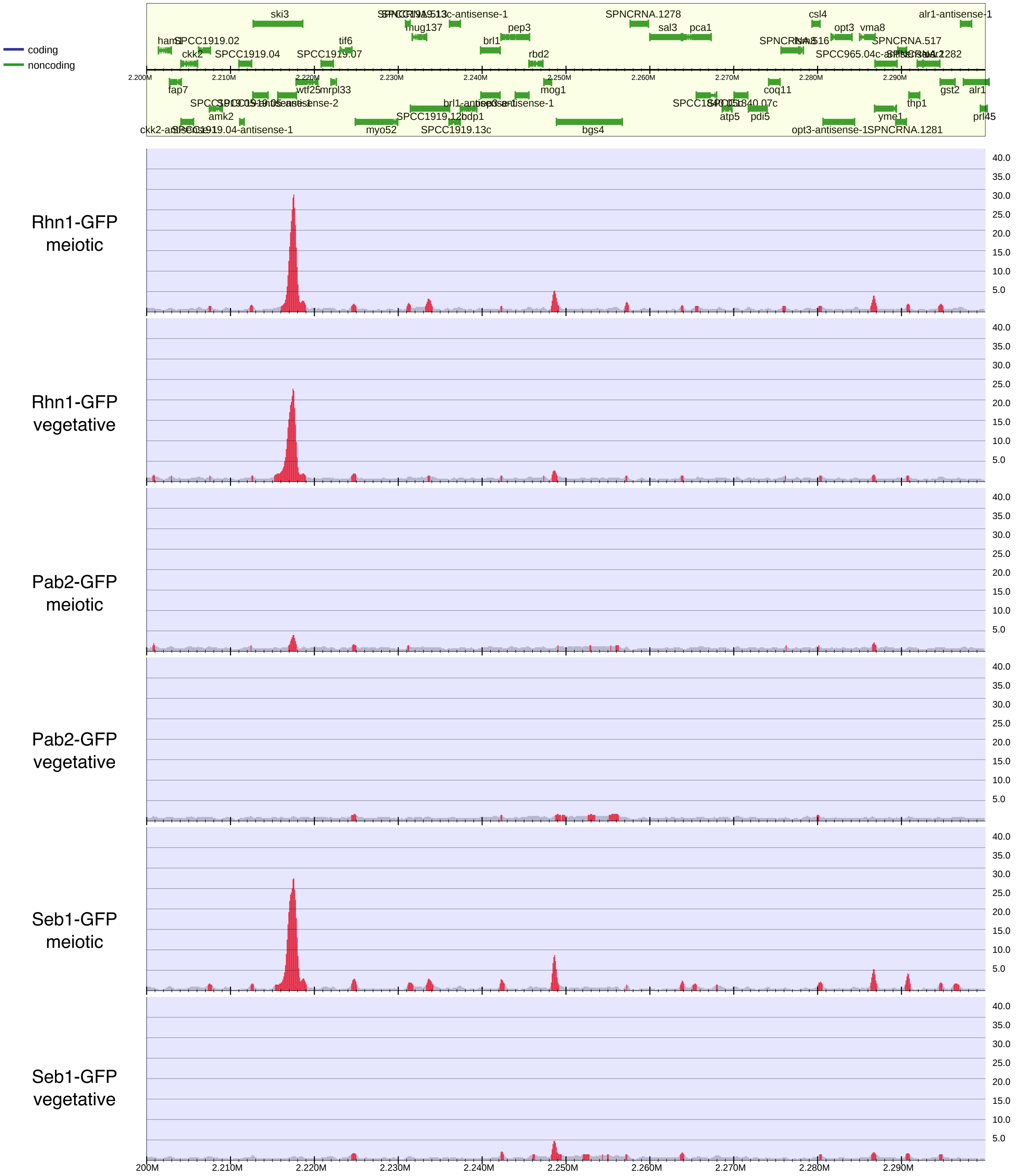

coding
noncoding
ski3
SPNCRNA.1278
csl4
alr1-antisense-1
mug137
pep3
sal3
pca1
opt3
vma8
ham1
SPCC1919.02
tif6
brl1
SPNCRNA.517
ckk2
SPCC1919.04
SPCC1919.07
rbd2
2.200M
2.210M
2.220M
2.230M
2.240M
2.250M
2.260M
2.270M
2.280M
2.290M
fap7
wtf25
mrpl33
mog1
coq11
gst2
alr1
thp1
amk2
bdp1
atp5
pdi5
yme1
prl45
myo52
SPCC1919.13c
bgs4
opt3-antisense-1
SPNCRNA.1281
Rhn1-GFP meiotic
Rhn1-GFP vegetative
Pab2-GFP meiotic
Pab2-GFP vegetative
Seb1-GFP meiotic
Seb1-GFP vegetative
40.0
35.0
30.0
25.0
20.0
15.0
10.0
5.0
200M
2.210M
2.220M
2.230M
2.240M
2.250M
2.260M
2.270M
2.280M
2.290M

III_1_24

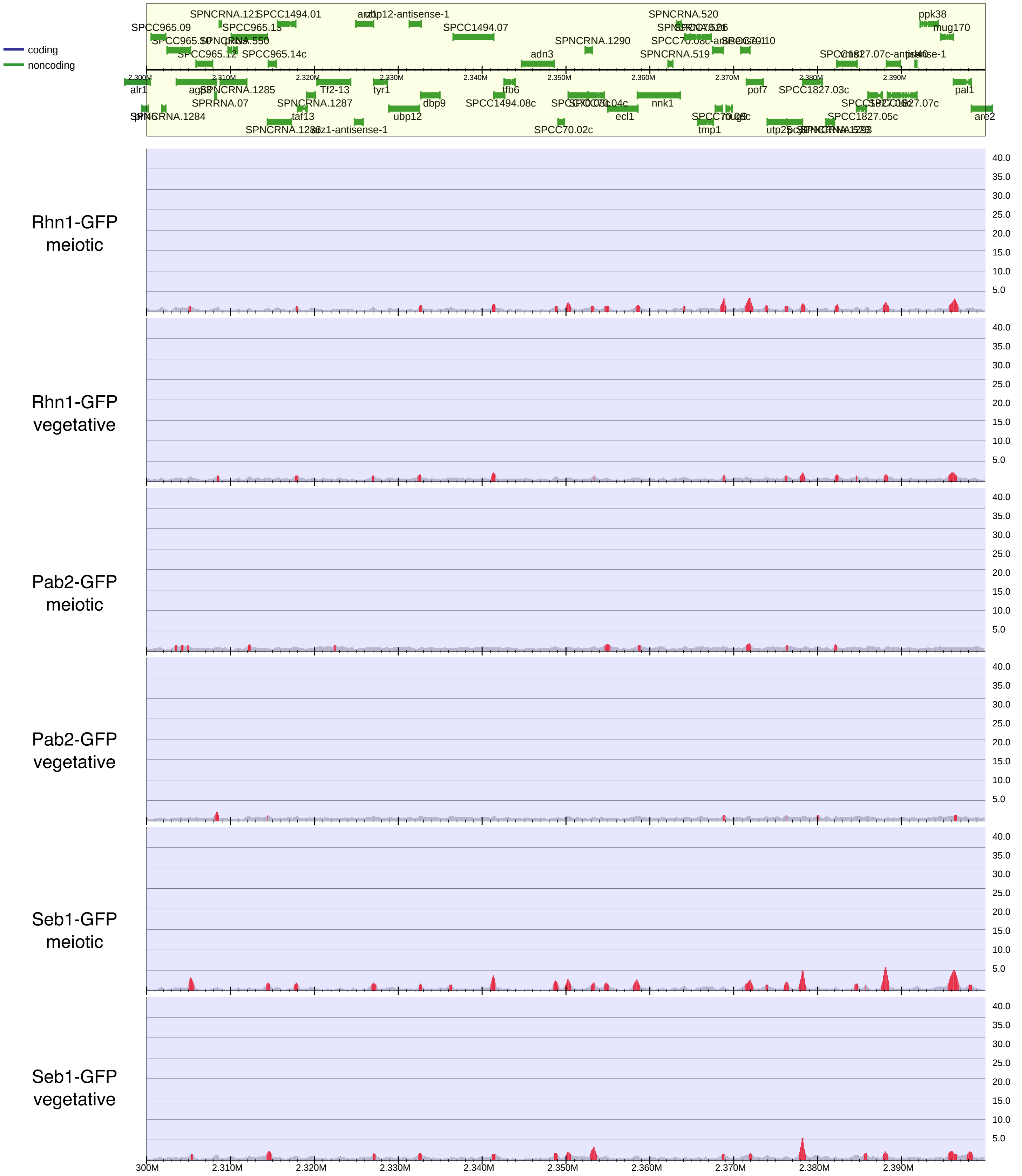
coding
noncoding
Rhn1-GFP meiotic
Rhn1-GFP vegetative
Pab2-GFP meiotic
Pab2-GFP vegetative
Seb1-GFP meiotic
Seb1-GFP vegetative
40.0
35.0
30.0
25.0
20.0
15.0
10.0
5.0
300M
2.310M
2.320M
2.330M
2.340M
2.350M
2.360M
2.370M
2.380M
2.390M

III_1_25

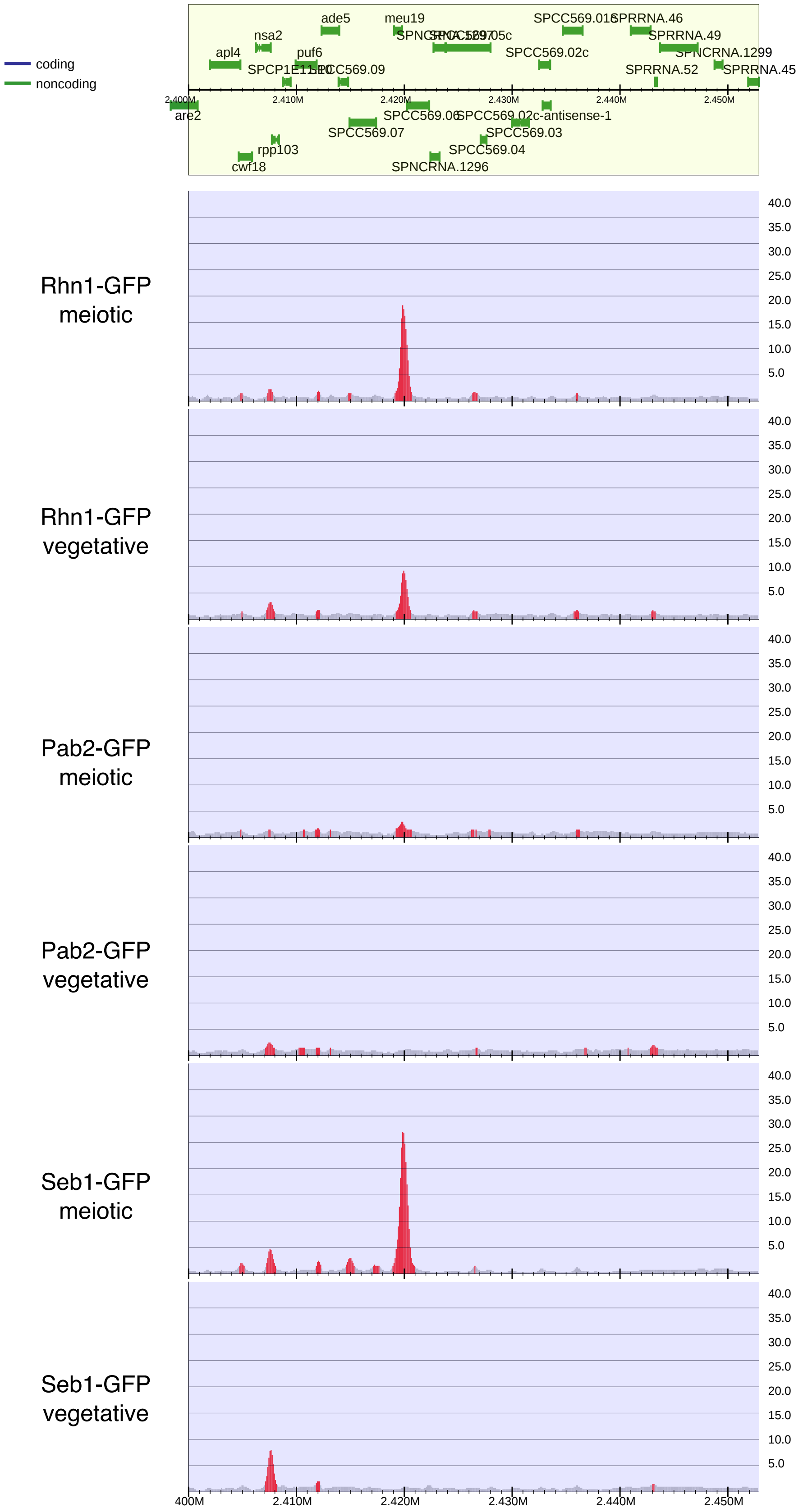
coding
noncoding
ade5
meu19
nsa2
apl4
puf6
SPCC569.02c
SPRRNA.46
SPRRNA.49
SPNCRNA.1299
SPRRNA.52
SPRRNA.45
are2
SPCC569.07
SPCC569.03
SPCC569.04
rpp103
cwf18
SPNCRNA.1296
SPCC569.02c-antisense-1
2.410M
2.420M
2.430M
2.440M
2.450M
Rhn1-GFP meiotic
Rhn1-GFP vegetative
Pab2-GFP meiotic
Pab2-GFP vegetative
Seb1-GFP meiotic
Seb1-GFP vegetative
40.0
35.0
30.0
25.0
20.0
15.0
10.0
5.0
400M

MT_1

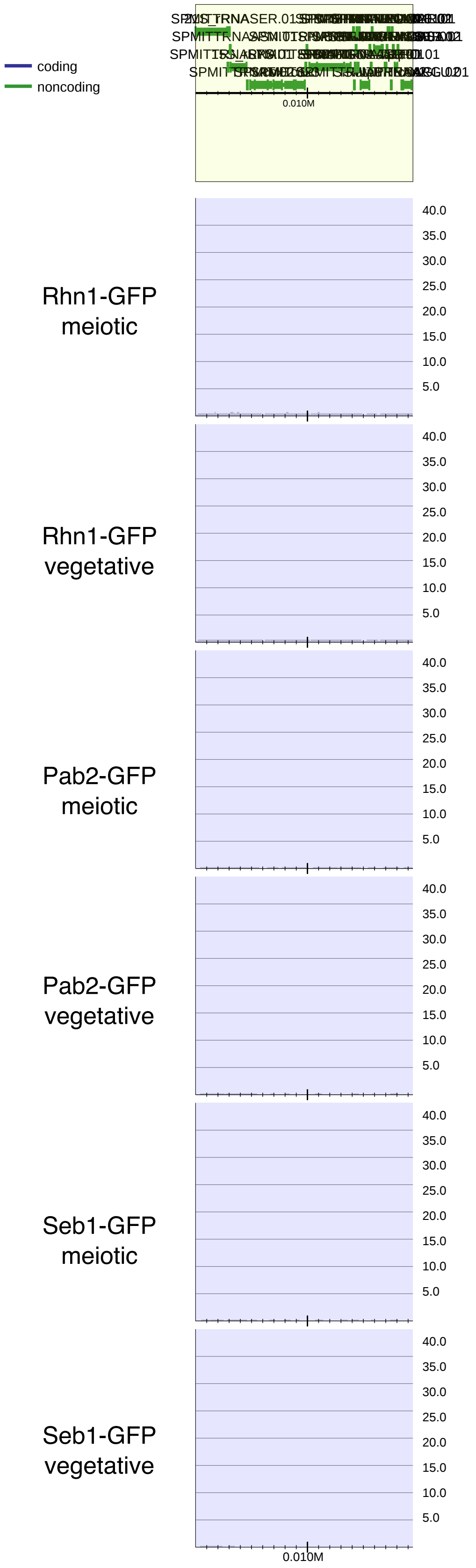
coding
noncoding
0.010M
Rhn1-GFP meiotic
Rhn1-GFP vegetative
Pab2-GFP meiotic
Pab2-GFP vegetative
Seb1-GFP meiotic
Seb1-GFP vegetative
40.0
35.0
30.0
25.0
20.0
15.0
10.0
5.0
0.010M

MTR_1

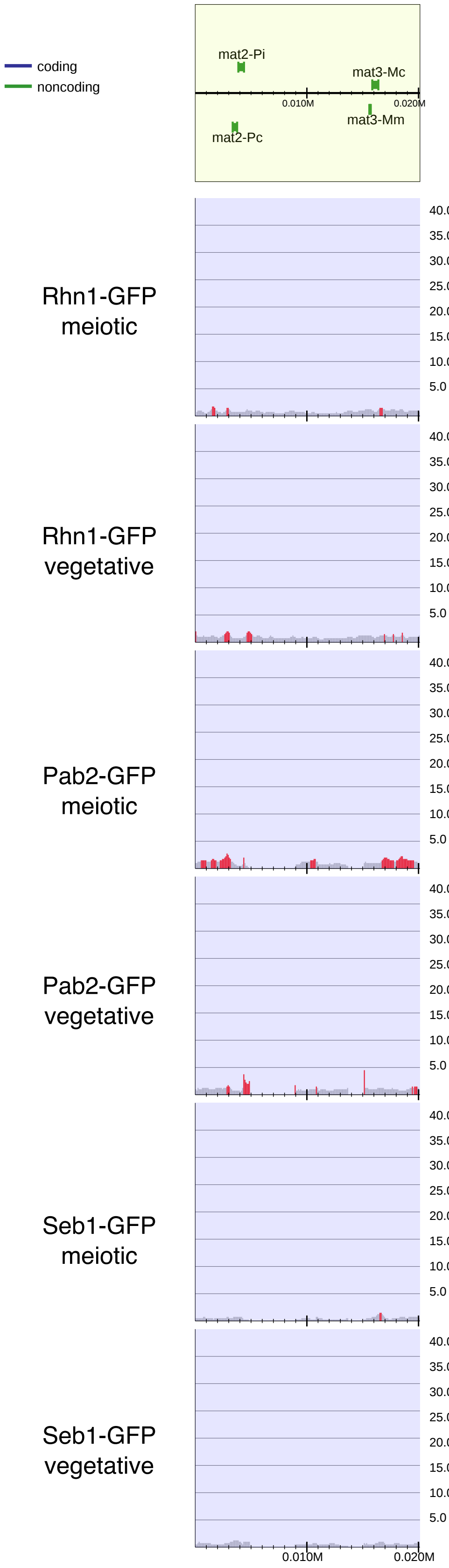

coding
noncoding
mat2-Pi
mat3-Mc
0.010M
0.020M
mat3-Mm
mat2-Pc
Rhn1-GFP meiotic
Rhn1-GFP vegetative
Pab2-GFP meiotic
Pab2-GFP vegetative
Seb1-GFP meiotic
Seb1-GFP vegetative
40.0
35.0
30.0
25.0
20.0
15.0
10.0
5.0

AB325691_1

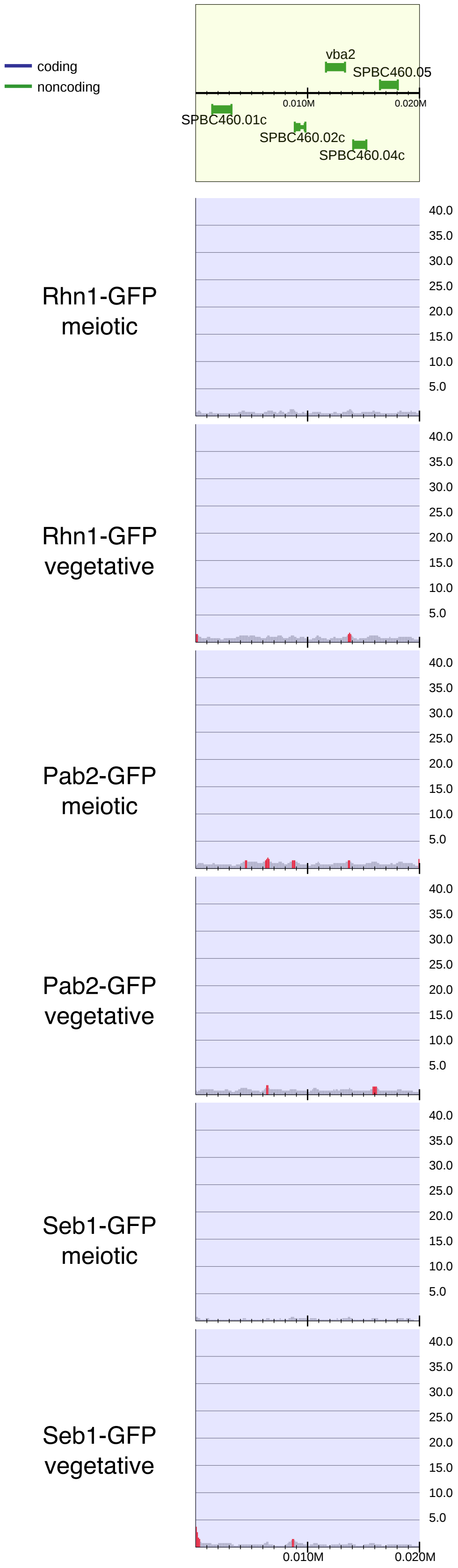

coding
noncoding
vba2
SPBC460.05
0.010M
0.020M
SPBC460.01c
SPBC460.02c
SPBC460.04c
Rhn1-GFP meiotic
Rhn1-GFP vegetative
Pab2-GFP meiotic
Pab2-GFP vegetative
Seb1-GFP meiotic
Seb1-GFP vegetative
40.0
35.0
30.0
25.0
20.0
15.0
10.0
5.0
0.010M
0.020M
