# Supplementary figures and images for: Chromosome-associated RNA–protein complexes promote pairing of homologous chromosomes during meiosis in Schizosaccharomyces pombe
Source: Nat Commun. 2019 Dec 6;10:5598. doi: 10.1038/s41467-019-13609-0 (PMC6898681; doi:10.1038/s41467-019-13609-0)

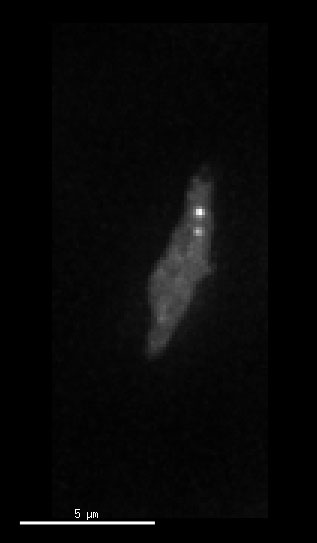

Supplement: Supplementary file 5 — Supplementary Movie 1 [file 41467_2019_13609_MOESM5_ESM.gif]

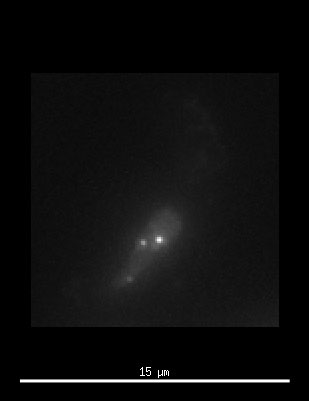

Supplement: Supplementary file 6 — Supplementary Movie 2 [file 41467_2019_13609_MOESM6_ESM.gif]

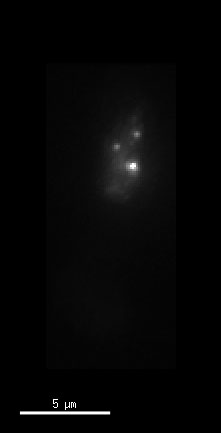

Supplement: Supplementary file 7 — Supplementary Movie 3 [file 41467_2019_13609_MOESM7_ESM.gif]

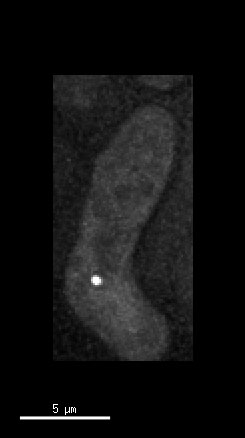

Supplement: Supplementary file 8 — Supplementary Movie 4 [file 41467_2019_13609_MOESM8_ESM.gif]

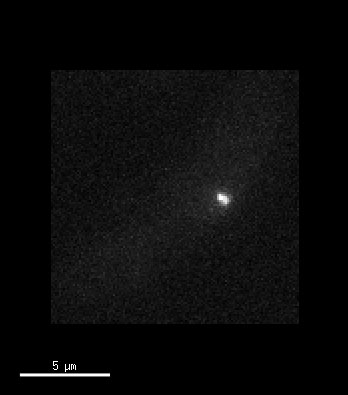

Supplement: Supplementary file 9 — Supplementary Movie 5 [file 41467_2019_13609_MOESM9_ESM.gif]

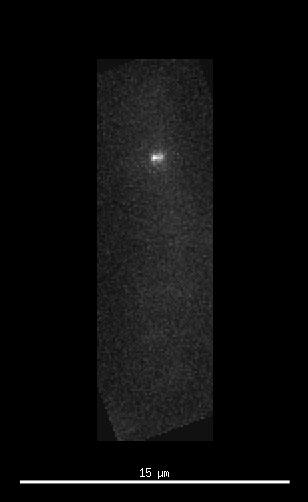

Supplement: Supplementary file 10 — Supplementary Movie 6 [file 41467_2019_13609_MOESM10_ESM.gif]
