# Supplementary material for: Chromosome-associated RNA–protein complexes promote pairing of homologous chromosomes during meiosis in Schizosaccharomyces pombe
Source: Nat Commun. 2019 Dec 6;10:5598. doi: 10.1038/s41467-019-13609-0 (PMC6898681; doi:10.1038/s41467-019-13609-0)
Supplement: Supplementary file 11 — Reporting Summary [file 41467_2019_13609_MOESM11_ESM.pdf]

nature research

Corresponding author(s): NCOMMS-19-09348

Last updated by author(s): Nov 8, 2019

Reporting Summary

Nature Research wishes to improve the reproducibility of the work that we publish. This form provides structure for consistency and transparency in reporting. For further information on Nature Research policies, see Authors & Referees and the Editorial Policy Checklist.

Statistics

For all statistical analyses, confirm that the following items are present in the figure legend, table legend, main text, or Methods section.

n/a	Confirmed	
☐	☒	The exact sample size (*n*) for each experimental group/condition, given as a discrete number and unit of measurement
☐	☒	A statement on whether measurements were taken from distinct samples or whether the same sample was measured repeatedly
☒	☐	The statistical test(s) used AND whether they are one- or two-sided<br>*Only common tests should be described solely by name; describe more complex techniques in the Methods section.*
☒	☐	A description of all covariates tested
☒	☐	A description of any assumptions or corrections, such as tests of normality and adjustment for multiple comparisons
☐	☒	A full description of the statistical parameters including central tendency (e.g. means) or other basic estimates (e.g. regression coefficient) AND variation (e.g. standard deviation) or associated estimates of uncertainty (e.g. confidence intervals)
☒	☐	For null hypothesis testing, the test statistic (e.g. *F*, *t*, *r*) with confidence intervals, effect sizes, degrees of freedom and *P* value noted<br>*Give P values as exact values whenever suitable.*
☒	☐	For Bayesian analysis, information on the choice of priors and Markov chain Monte Carlo settings
☒	☐	For hierarchical and complex designs, identification of the appropriate level for tests and full reporting of outcomes
☒	☐	Estimates of effect sizes (e.g. Cohen's *d*, Pearson's *r*), indicating how they were calculated

Our web collection on statistics for biologists contains articles on many of the points above.

Software and code

Policy information about availability of computer code

Data collection	Imaging data were collected using SoftWorx (GE)
Data analysis	Image data analysis was using SoftWorx (GE). Chromatic shifts were corrected by using the “Chromagnion” software (https://github.com/macronucleus/Chromagnon)

For manuscripts utilizing custom algorithms or software that are central to the research but not yet described in published literature, software must be made available to editors/reviewers. We strongly encourage code deposition in a community repository (e.g. GitHub). See the Nature Research guidelines for submitting code & software for further information.

Data

Policy information about availability of data

All manuscripts must include a data availability statement. This statement should provide the following information, where applicable:

- Accession codes, unique identifiers, or web links for publicly available datasets
- A list of figures that have associated raw data
- A description of any restrictions on data availability

The authors declare that all the other data supporting the findings of this study are available within the paper and its supplementary information files, and from the corresponding author upon reasonable request.

Field-specific reporting

Please select the one below that is the best fit for your research. If you are not sure, read the appropriate sections before making your selection.

☒ Life sciences ☐ Behavioural & social sciences ☐ Ecological, evolutionary & environmental sciences

For a reference copy of the document with all sections, see nature.com/documents/nr-reporting-summary-flat.pdf

Life sciences study design

All studies must disclose on these points even when the disclosure is negative.

Sample size	The sample size is provided in Source Data files.
Data exclusions	No data were excluded from analyses.
Replication	For following pairing of homologous chromosome in live cells, experiments were repeated until data were successfully collected from at least 18 cells. For counting fluorescent signals in live or fixed cells, independent experiments were repeated for three times, and all replication were successful. The precise number of cells examined is provided in Source Data files.
Randomization	NA
Blinding	NA

Reporting for specific materials, systems and methods

We require information from authors about some types of materials, experimental systems and methods used in many studies. Here, indicate whether each material, system or method listed is relevant to your study. If you are not sure if a list item applies to your research, read the appropriate section before selecting a response.

Materials & experimental systems

n/a	Involved in the study	
☐	☒	Antibodies
☒	☐	Eukaryotic cell lines
☒	☐	Palaeontology
☒	☐	Animals and other organisms
☒	☐	Human research participants
☒	☐	Clinical data

Methods

n/a	Involved in the study	
☐	☒	ChIP-seq
☒	☐	Flow cytometry
☒	☐	MRI-based neuroimaging

Antibodies

Antibodies used	Full-length A.v. polyclonal GFP antibody (632460; Clontech, Mountain View, CA, USA) ,anti-Flag M2 antibody (A8592, Merck), anti-α-tubulin (T5168, Merck), peroxidase-conjugated AffiPure anti-mouse IgG (515-035-072, Jackson)
Validation	https://www.takarabio.com/assets/documents/Certificate%20of%20Analysis/632592-632593-072412.pdf

ChIP-seq

Data deposition

☒ Confirm that both raw and final processed data have been deposited in a public database such as GEO.

☒ Confirm that you have deposited or provided access to graph files (e.g. BED files) for the called peaks.

Data access links<br>*May remain private before publication.*	https://www.ncbi.nlm.nih.gov/sra/?term=SRP129475
Files in database submission	SRR6472706~SRR6472717
Genome browser session<br>(e.g. UCSC)	https://www.pombase.org/

Methodology

Replicates	One replicate for each ChIP-input sample pair
Sequencing depth	Average reads 6892222, mapped unique 98.45%, unmapped 1.59%, average read length=51
Antibodies	Full-length A.v. polyclonal GFP antibody (632460; Clontech, Mountain View, CA, USA)
Peak calling parameters	Fold enrichments (ChIP/WCE) of more than 1.5 for each 100-bp window
Data quality	Peak numbers:<br>Rhn1-mito 1901, Rhn1-meio, 2794; Pab2-mito 1042, Pab2-meio 1446; Seb1-mito 1817, Seb1-meio 2714
Software	Mapping: bowtie (http://bowtie-bio.sourceforge.net/index.shtml), visualization and peak calling: DROMPA (https://github.com/rnakato/DROMPA3).
